# Total synthesis and antiviral activity of indolosesquiterpenoids from the xiamycin and oridamycin families

**DOI:** 10.1038/ncomms7096

**Published:** 2015-02-04

**Authors:** Zhanchao Meng, Haixin Yu, Li Li, Wanyin Tao, Hao Chen, Ming Wan, Peng Yang, David J. Edmonds, Jin Zhong, Ang Li

**Affiliations:** 1 State Key Laboratory of Bioorganic and Natural Products Chemistry, Shanghai Institute of Organic Chemistry, Chinese Academy of Sciences, 345 Lingling Road, Shanghai 200032, China; 2 Unit of Viral Hepatitis, Key Laboratory of Molecular Virology and Immunology, Institut Pasteur of Shanghai, Shanghai Institutes for Biological Sciences, Chinese Academy of Sciences, Shanghai 200032, China; 3 Pfizer Worldwide Research and Development, Cambridge, Massachusetts 02139, USA; 4 Collaborative Innovation Center of Chemistry for Life Sciences, 22 Hankou Road, Nanjing, Jiangsu 210093, China

## Abstract

Indolosesquiterpenoids are a growing class of natural products that exhibit a wide range of biological activities. Here, we report the total syntheses of xiamycin A and oridamycins A and B, indolosesquiterpenoids isolated from *Streptomyces*. Two parallel strategies were exploited to forge the carbazole core: 6π-electrocyclization/aromatization and indole C2–H bond activation/Heck annulation. The construction of their *trans*-decalin motifs relied on two diastereochemically complementary radical cyclization reactions mediated by Ti(III) and Mn(III), respectively. The C23 hydroxyl of oridamycin B was introduced by an sp^3^ C–H bond oxidation at a late stage. On the basis of the chemistry developed, the dimeric congener dixiamycin C has been synthesized for the first time. Evaluation of the antiviral activity of these compounds revealed that xiamycin A is a potent agent against herpes simplex virus–1 (HSV-1) *in vitro*.

## Abstract (web summary)

Using a single strategy to make a number of related intermediates is a useful strategy in the total synthesis. Here, the authors report the synthesis of a number of natural products, employing two diastereomerically complementary metal-catalyzed cyclizations as the key step to access a number of frameworks.

## Graphical abstract

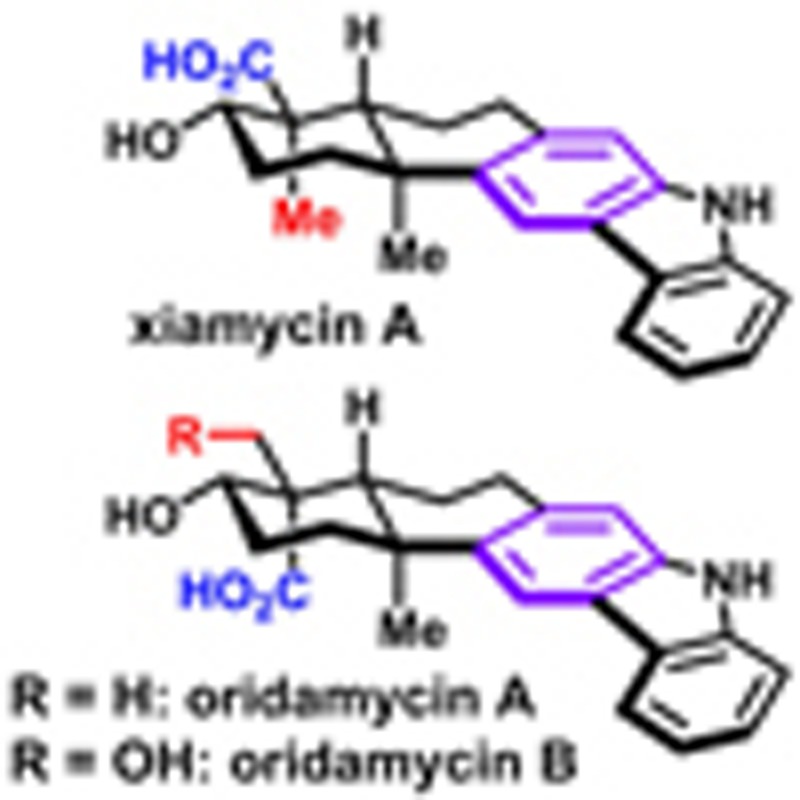

Indolosesquiterpenoids have attracted increasing attention from chemical and biosynthetic perspectives^1^,^2^,^3^,^4^,^5^,^7^; however, their biological activities are yet to be systematically explored through chemical synthesis^8^,^9^,^10^,^11^,^12^,^13^,^14^. Xiamycin A (**1**, Fig. 1), isolated independently from *Streptomyces* species by Hertweck^15^,^16^,^17^,^18^ and Zhang^19^,^20^,^21^, displays selective anti-HIV activity. The naturally occurring antibiotics oridamycins A and B (**2** and **3**) share a similar scaffold to that of **1**, though with the opposite stereochemistry at quaternary C16 (ref. 22). Several congeners of **1** were also identified from the same species, including the C6–N1′ dimer dixiamycin C (**4**), the C11–C12 bond oxidized and ring-expanded oxiamycin (**5**) and a putative biosynthetic precursor, indosespene (**6**)^17^,^18^,^19^,^20^,^21^. Considerable efforts have been made towards revealing the biogenesis of this class of natural products, including the elucidation of the relevant gene clusters and enzymes^17^,^18^,^19^,^20^. The chemical synthesis of these compounds^13^,^14^ may help further the understanding of the biosynthetic relationships within this indolosesquiterpenoid family, as well as the discovery of their unknown biological activities. Very recently, Baran *et al*.^13^ disclosed the first total synthesis of xiamycin A (**1**) and dixiamycin B.

In this paper, we report the total syntheses of xiamycin A, oridamycins A and B, dixiamycin C (**1**–**4**) and indosespene (**6**). The syntheses of **2**–**4** were accomplished for the first time. A 6π-electrocyclization strategy and an oxidative Heck annulation strategy were developed for assembling the carbazole scaffold. The antiviral properties of the synthetic natural products were evaluated *in vitro*, and **1** exhibits potent inhibitory activity against herpes simplex virus–1 (HSV-1).

## Results

### Retrosynthetic analysis

The synthetic challenge of the xiamycin and oridamycin families may be separated into two distinct problems, namely the stereocontrolled assembly of the *trans*-decalin motif bearing the C16 quaternary centre and the construction of the carbazole core. Inspired by the structural correlation between xiamycin A (**1**) and indosespene (**6**), our retrosynthetic analysis of **1** (Fig. 2) began with a disconnection at the C2–C21 bond. A 6π-electrocyclization^23^ (path a) and a Heck-type annulation^24^,^25^,^26^,^27^,^28^,^29^ (path b) were envisioned as two alternatives to forge the carbazole motif. The former draws on our recent syntheses of tubingensin A and daphenylline^30^,^31^, while the latter relies on activation of the indole C2–H bond by palladium. Correspondingly, triene (**7**) and indosespene ester (**8**) are considered as the key intermediates for the two strategies. These functionalized decalin systems are expected to arise from a cascade cyclization of dienylepoxide precursors, such as **9** and **10**. This type of radical cyclization may be initiated by reduction of the epoxide by a Ti(III) species^32^,^33^,^34^,^35^,^36^,^37^,^38^,^39^,^40^,^41^,^42^,^43^,^44^,^45^,^46^,^47^, although, to our knowledge, α,β-epoxy esters/ketones have not been fully explored as the substrates for such cyclization^14^,^48^. Notably, Cuerva and coworkers^49^ disclosed that enol radicals derived from α,β-unsaturated ketones inhibit the cyclization reaction. The stereochemical outcome of the cyclization cascade (including the stereochemistry at C16) would be determined by the chirality and geometry of the precursor, which may allow a convenient access to oridamycins A and B (**2** and **3**) from an alternative precursor. Successful execution of the route outlined above would also be anticipated to provide access to indosespene (**6**) and dixiamycin C (**4**) through minor modifications.

### The 6π-electrocyclization/aromatization approach

Fig. 3 illustrates the 6π-electrocyclization/aromatization approach for the synthesis of xiamycin A (**1**). Starting from optically active epoxide **11** (96% ee)^50^, a sequence of 2-azaadamantane *N*-oxyl (AZADO) oxidation^51^,^52^ and esterification led to compound **12** in 76% overall yield. On the basis of seminal work on the Ti(III)-catalysed bioinspired epoxypolyene cyclization by Cuerva and Oltra^38^,^39^,^40^,^41^, we investigated the cyclization of the α,β-epoxy ester **12**. Under the optimized conditions [Cp_2_TiCl_2_ (20 mol%), Mn, *i*Pr_2_NEt, TMSCl], the desired *trans*-decalin **13** was obtained as a single diastereomer. TBS protection followed by acetyl deprotection gave **14** (44% yield for the three steps), which was subjected to a sequence of DMP oxidation, Grignard addition (with reagent **15**)^30^, and dehydration (MsCl/*i*Pr_2_NEt) to furnish triene **16** in 75% overall yield. On microwave irradiation (120 °C, air), **16** underwent a thermal 6π-electrocyclization and a subsequent aromatization to generate carbazole **17** (80% yield). Similarly, when using Grignard reagent **18** with a bromine substituent, we readily obtained compound **19** via the corresponding triene **20**. This bromocarbazole served as an appropriate substrate for the crucial C–N bond formation in our dixiamycin C synthesis (*vide infra*). Desulfonylation of **17** followed by TMSE deprotection of the resulting compound **21** afforded xiamycin A (**1**) in a good overall yield, the structure of which was confirmed by X-ray crystallographic analysis (Fig. 4).

The mechanism of the cascade cyclization is postulated in Fig. 5. The epoxide C16–O bond is reductively cleaved by the Ti(III) species to generate **22**, which triggers two sequential cyclization reactions, and the resulting radical **23** could undergo a mixed disproportionation^53^ to give the intermediate **24** with an exocyclic C=C bond. In fact, two side reactions occurred: 1, over reduction of **22** to the corresponding enolate^48^ followed by β-elimination to form an α,β-unsaturated ester; 2, hydrogen transfer to **23** to form the saturated derivative of **24**. Organic bases had subtle influence on these undesired reactions^54^, and *i*Pr_2_NEt was found to suppress the latter effectively in our case. Considering the challenges for the acid-promoted epoxy polyene cyclization, such as the preference to the formation of products with tri-/tetrasubstituted olefins and difficulty generating carbocations with electron-withdrawing substituents, this radical chemistry offers a useful and complementary route for preparing functionalized sesquiterpenoid synthons.

We then focused on the synthesis of oridamycin A bearing an axial carboxylic substituent on C16 (Fig. 6). The *cis*-epoxy analogue of **12** was first prepared and subjected to the Ti(III) conditions. However, the compound **13** with an equatorial carboxylic substituent was detected as the major product, presumably due to the fast rotation of the C15–C16 bond to adopt the sterically favoured conformation (Fig. 5) for the cyclization. Thus, we made recourse to a mechanistically different oxidative radical cyclization mediated by Mn(III)^55^,^56^,^57^,^58^,^59^ using β-ketoester **25** (ref. 56) as a substrate. Treatment with Mn(OAc)_3_/Cu(OAc)_2_ in DMSO effected the radical cyclization with an acceptable yield of decalin **26** (48%, a single diastereomer detected). This compound was subjected to a sequence similar to that described above to afford triene **27** with good overall efficiency, via the intermediacy of aldehyde **28**. Under thermal conditions (120 °C) and in presence of air, **27** was converted to carbazole **29** (83% yield) in one pot. Sequential ketone reduction (NaBH_4_) and desulfonylation (Mg/MeOH) provided compound **30** in 83% overall yield. Final TMSE deprotection led to the completion of the first synthesis of (±)-oridamycin A (**2**).

We accomplished the synthesis of oridamycin B for the first time by adjusting the C23 oxidation state of **29** (Fig. 6), taking advantage of the neighbouring ketone carbonyl group. *O*-Methyloxime formation afforded the substrate suitable for a directed C–H bond oxidation, and Sanford’s conditions [Pd(OAc)_2_ (20 mol%), PhI(OAc)_2_]^60^,^61^ were effective to give the desired acetate **31** with good overall efficiency. It should be noted that stoichiometric Pd-mediated Csp^3^–H bond oxidation reactions have been exploited in terpenoid synthesis^41^,^62^,^63^. Deprotection of the oxime and acetate, followed by reduction and desulfonylation, afforded diol **32** with acceptable overall efficiency. Removal of TMSE with TASF provided (±)-oridamycin B (**3**) in 91% yield. The spectroscopic data of the synthetic samples of oridamycins A and B matched those reported for the natural products.

### The C–H bond activation/Heck annulation approach

The second approach to xiamycin A (**1**) fulfilled the strategy of indole C2–H activation/Heck-type annulation (Fig. 7). Stille coupling^64^ of **12** with *N*-Boc-3-(tributylstannyl)indole **33**^65^ followed by deprotection of Boc^66^ provided a fully functionalized precursor **34** (66% overall yield), which underwent a Ti(III)-catalysed radical cyclization under the same conditions as above to give decalin **35** as a single diastereomer in 60% yield^39^. In the presence of Pd(OAc)_2_ and *p*-benzoquinone at 50 °C (refs 28, 29), **35** was converted to carbazole **21** in 81% yield, presumably through the devised oxidative Heck/aromatization sequence. TMSE deprotection of **35** and **21** with TASF then afforded indosespene (**6**) and xiamycin A (**1**), respectively. The structure of **6** was verified by X-ray crystallographic analysis (Fig. 4).

The above strategy was further applied to alternative syntheses of oridamycins A and B (Fig. 8). In this case, an indole-containing β-ketoester **36** was employed as the substrate for the Mn(III) mediated cyclization. *N*-Sulfonylation of 3-geranylindole **37 (**ref. 67; NaOH, PhSO_2_Cl) and subsequent allylic oxidation (SeO_2_, *t*BuOOH) gave alcohol **38** in 78% overall yield, which underwent bromination (Et_3_N, MsCl; LiBr) followed by alkylation with the dianion generated from β-ketoester **39** (KH, then BuLi) to furnish **36** (80% yield for the two steps). Under the similar conditions used for the cyclization of compound **25** (Fig. 6), decalin **40** was obtained in 52% yield as a single detectable diastereomer. Its C16 stereochemistry was secured by X-ray crystallographic analysis (Fig. 4). Desulfonylation (Mg/MeOH, 96% yield) and subsequent oxidative Heck annulation (*vide supra*) afforded carbazole **41** (65% yield), which was subjected to a two-step sequence of ketone reduction and TMSE deprotection to give (±)-oridamycin A (**2**). Sanford Csp^3^–H bond oxidation was exploited again for the synthesis of oridamycin B from **41**; deactivation of the carbazole motif with an electron-withdrawing *N*-protecting group was required. Thus, **41** was subjected to NH_2_OMe condensation and Boc protection to afford the oxidation precursor. The same conditions [Pd(OAc)_2_ (20 mol%), PhI(OAc)_2_] as used before delivered acetate **42** smoothly (67% yield for the three steps). Global deprotection of the oxime, acetyl and Boc groups under acidic conditions followed by reduction of the exposed carbonyl group furnished the late intermediate **32** in 54% overall yield, which was converted to (±)-oridamycin B (**3**) through the removal of TMSE group.

### The synthesis of dixiamycin C

Having synthesized the monomeric natural products, we directed our attention to the synthesis of dixiamycin C (Fig. 9). Starting from coupling partners **19** and **21**, we investigated a variety of conditions for the transition metal promoted C–N bond formation. Buchwald’s protocol^68^ [CuI, (±)-**43**, K_3_PO_4_, 110 °C] proved to be optimal to provide dimer **44**. Deprotection of the benzenesulfonyl and TMSE groups furnished dixiamycin C (**4**) in a good overall yield, via the intermediacy of **45**. The spectra and physical properties of the synthetic dixiamycin C were consistent with those reported for the natural products.

### The antiviral activity

**Figure 1:**
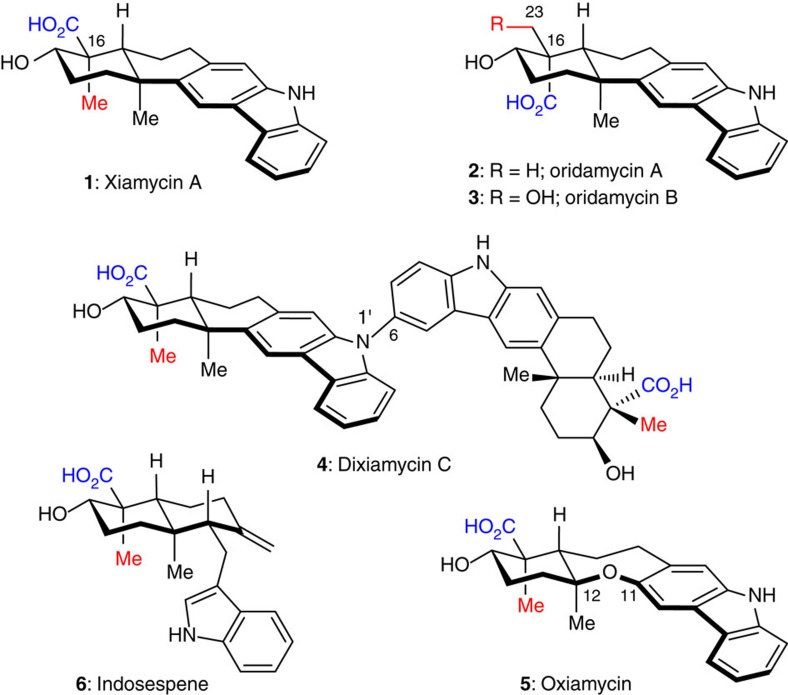
The structures of representative natural products from the xiamycin and oridamycin families. The two families share a similar scaffold but differ in the C16 stereochemistry. Indosespene (**6**) is a putative biosynthetic precursor of xiamycin A (**1**).

**Figure 2:**
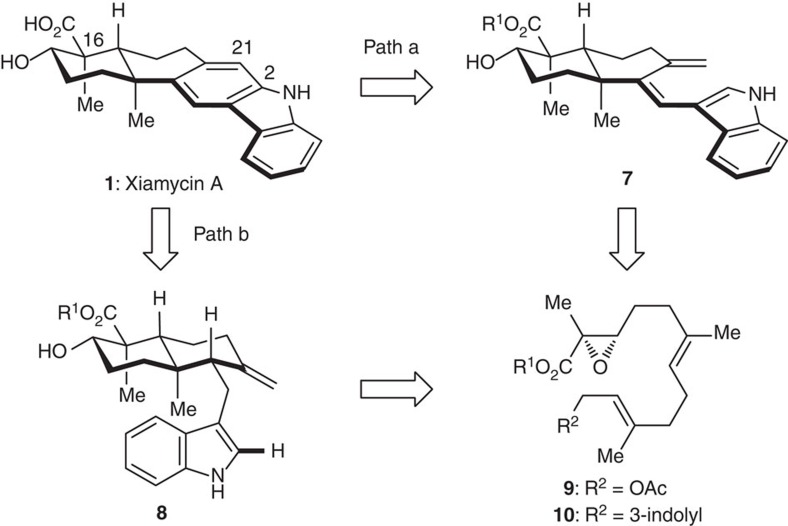
Two retrosynthetic analyses of xiamycin A. One (path a) features a 6π-electrocyclization/aromatization strategy, and the other (path b) involves an indole C2–H bond activation/Heck annulation strategy. The devised strategies are anticipated to be applicable to the synthesis of other members from the xiamycin and oridamycin families.

**Figure 3:**
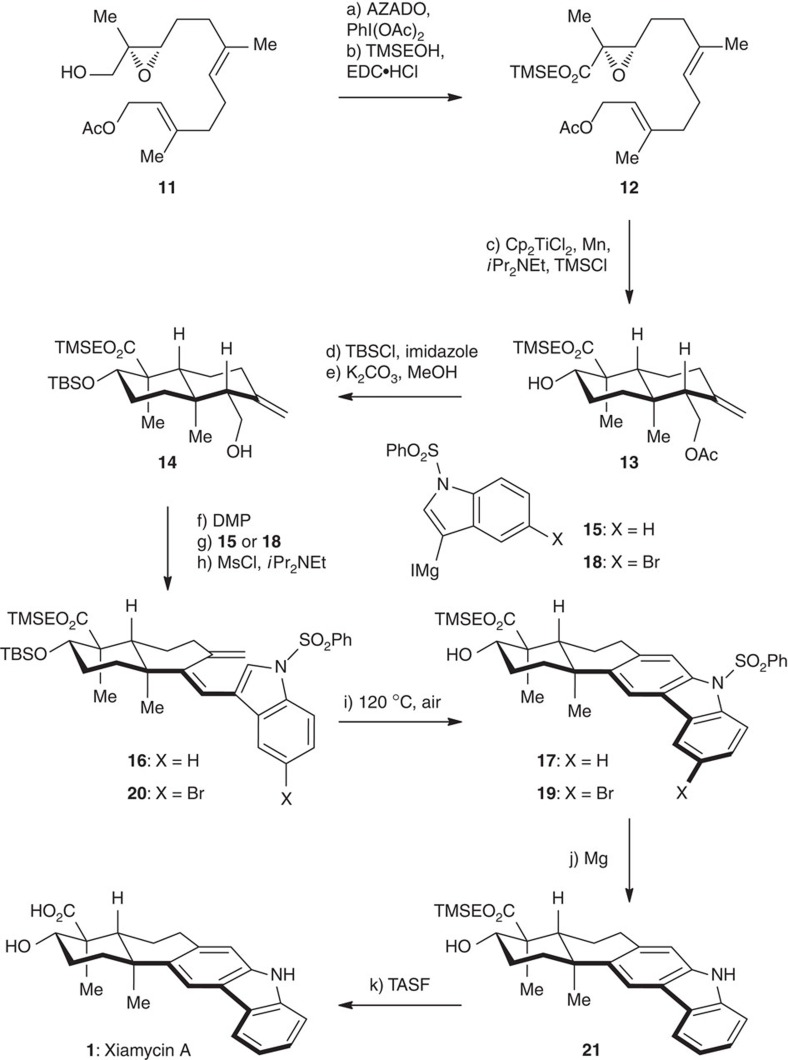
The 6π-electrocyclization/aromatization approach towards the total synthesis of xiamycin A. Reagents and conditions: (a) AZADO (5 mol%), PhI(OAc)_2_ (3.0 eq), CH_2_Cl_2_, 22 °C, 6 h; (b) TMSEOH (1.2 eq), EDC·HCl (1.1 eq), 4-DMAP (1.0 eq), CH_2_Cl_2_, 22 °C, 5 h, 76% (two steps); (c) Cp_2_TiCl_2_ (20 mol%), Mn (8.0 eq), *i*Pr_2_NEt (6.0 eq), TMSCl (5.0 eq), THF, 22 °C, 4 h; (d) TBSCl (1.2 eq), imidazole (1.5 eq), DMF, 22 °C, 10 h; (e) K_2_CO_3_ (1.0 eq), MeOH, 22 °C, 3 h, 44% (3 steps); (f) DMP (1.5 eq), CH_2_Cl_2_, 22 °C, 30 min; (g) **15** or **18** (3.0 eq), THF, 22 °C, 40 min; (h) MsCl (1.2 eq), *i*Pr_2_NEt (1.5 eq), CH_2_Cl_2_, 0 °C, 1 h, 75% for **16** (three steps), 71% for **20** (three steps); (**i**) air, DMSO, 120 °C, 2 h, 80% for **17**, 76% for **19**; (**j**) Mg (2.0 eq), MeOH, 22 °C, 1 h, 98%; (k) TASF (2.0 eq), DMF, 50 °C, 95%.

**Figure 4:**
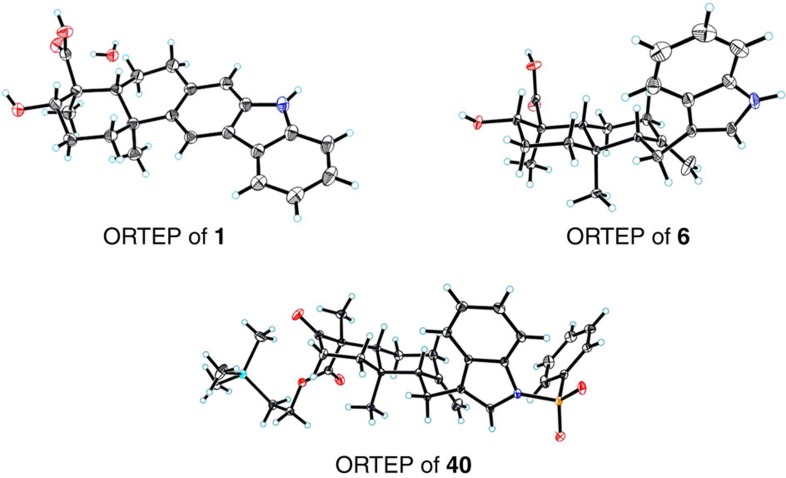
ORTEP drawings of compounds **1**, **6** and **40**. Non-hydrogen atoms are shown as 30% ellipsoids. The stereochemical outcomes of the radical cyclization cascades are unambiguously confirmed.

**Figure 5:**
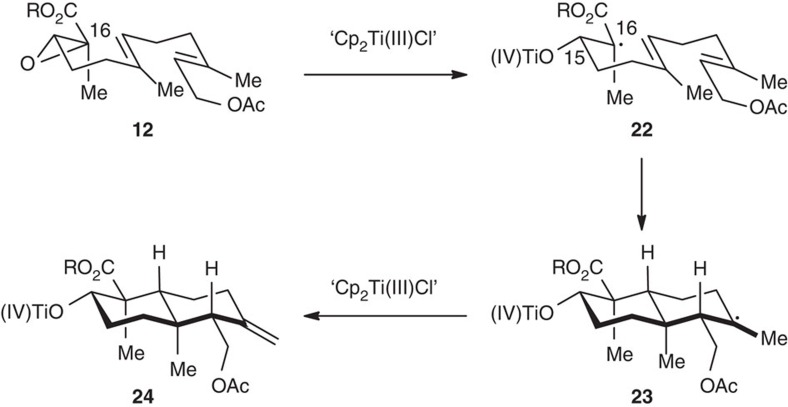
A postulated mechanism of the Ti(III)-mediated radical cyclization. The epoxy ester serves as a suitable substrate for this reaction. The *trans*-decalin system with an equatorial alkoxycarbonyl and an exocyclic methylene groups is obtained.

**Figure 6:**
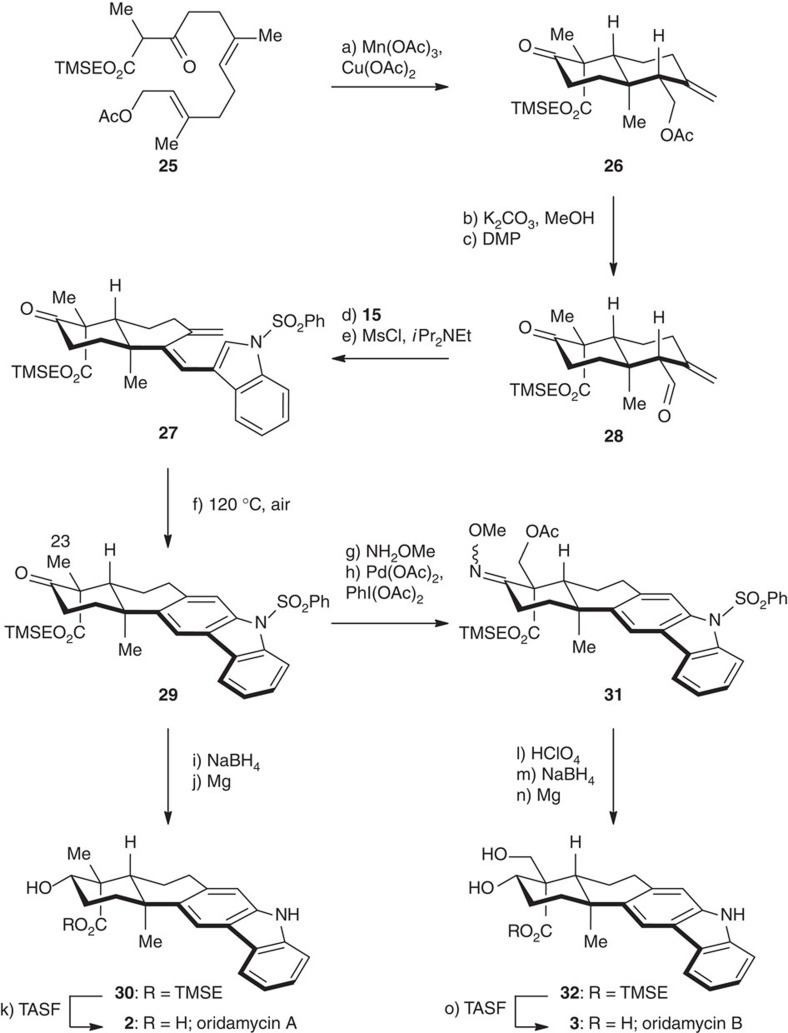
The 6π-electrocyclization/aromatization approach towards the total syntheses of oridamycins A and B. Reagents and conditions: (a) Mn(OAc)_3_·2H_2_O (2.0 eq), Cu(OAc)_2_·H_2_O (1.0 eq), DMSO, 22 °C, 12 h, 48%; (b) K_2_CO_3_ (1.5 eq), MeOH, 22 °C, 3 h; (c) DMP (2.0 eq), CH_2_Cl_2_, 22 °C, 30 min, 92% (two steps); (d) **15** (3.0 eq), THF, 22 °C, 40 min; (e) MsCl (1.2 eq), *i*Pr_2_NEt (1.5 eq), CH_2_Cl_2_, 0 °C, 1 h, 74% (2 steps); (f) air, DMSO, 120 °C, 2 h, 83%; (g) NH_2_OMe·HCl (2.0 eq), pyridine, 22 °C, 2 h, 94%; (h) Pd(OAc)_2_ (20 mol%), PhI(OAc)_2_ (1.0 eq), Ac_2_O/AcOH (1:1), 110 °C, 1 h, 82%; (i) NaBH_4_ (3.0 eq), MeOH, 0 °C, 30 min; (j) Mg (8.0 eq), MeOH, 22 °C, 1 h, 83% (two steps); (k) TASF (2.0 eq), DMF, 50 °C, 92%; (l) aq. HClO_4_ (6.0 M)/acetone (1:3), 22 °C, 12 h; (m) NaBH_4_ (3.0 eq), MeOH, 0 °C, 30 min, 62% (2 steps); (n) Mg (8.0 eq), MeOH, 22 °C, 1 h, 90%; (o) TASF (2.0 eq), DMF, 50 °C, 91%.

**Figure 7:**
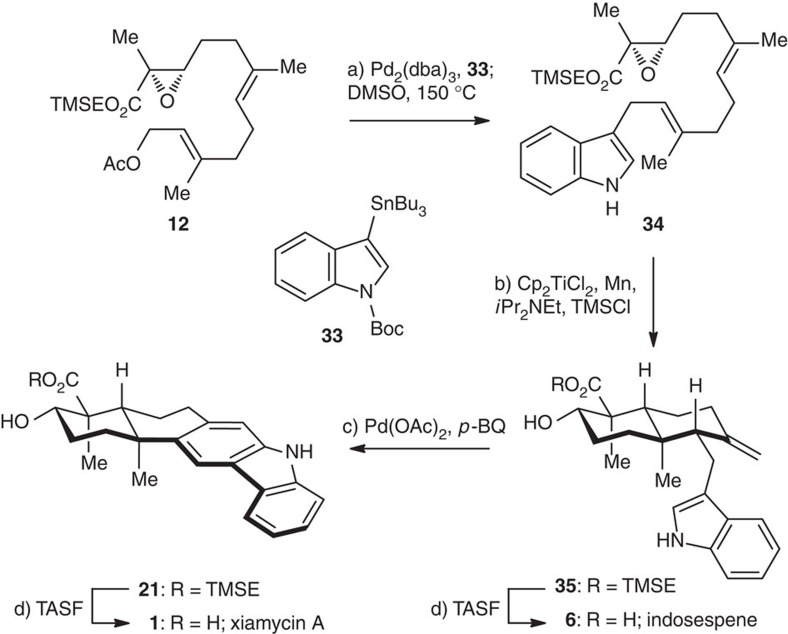
The oxidative Heck annulation approach towards the total synthesis of xiamycin A. Reagents and conditions: (a) Pd_2_(dba)_3_ (5 mol%), LiCl (3.0 eq), **33** (2.0 eq), DMF, 80 °C, 2 h, then DMSO, 150 °C, 30 min, 66%; (b) Cp_2_TiCl_2_ (20 mol%), Mn (8.0 eq), *i*Pr_2_NEt (6.0 eq), TMSCl (5.0 eq), THF, 22 °C, 4 h, 60%; (c) Pd(OAc)_2_ (20 mol%), *p*-benzoquinone (2.0 eq), AcOH/toluene (1:4), 50 °C, 2 h, 81%; (d) TASF (2.0 eq), DMF, 50 °C, 95% for **1**, 91% for **6**.

**Figure 8:**
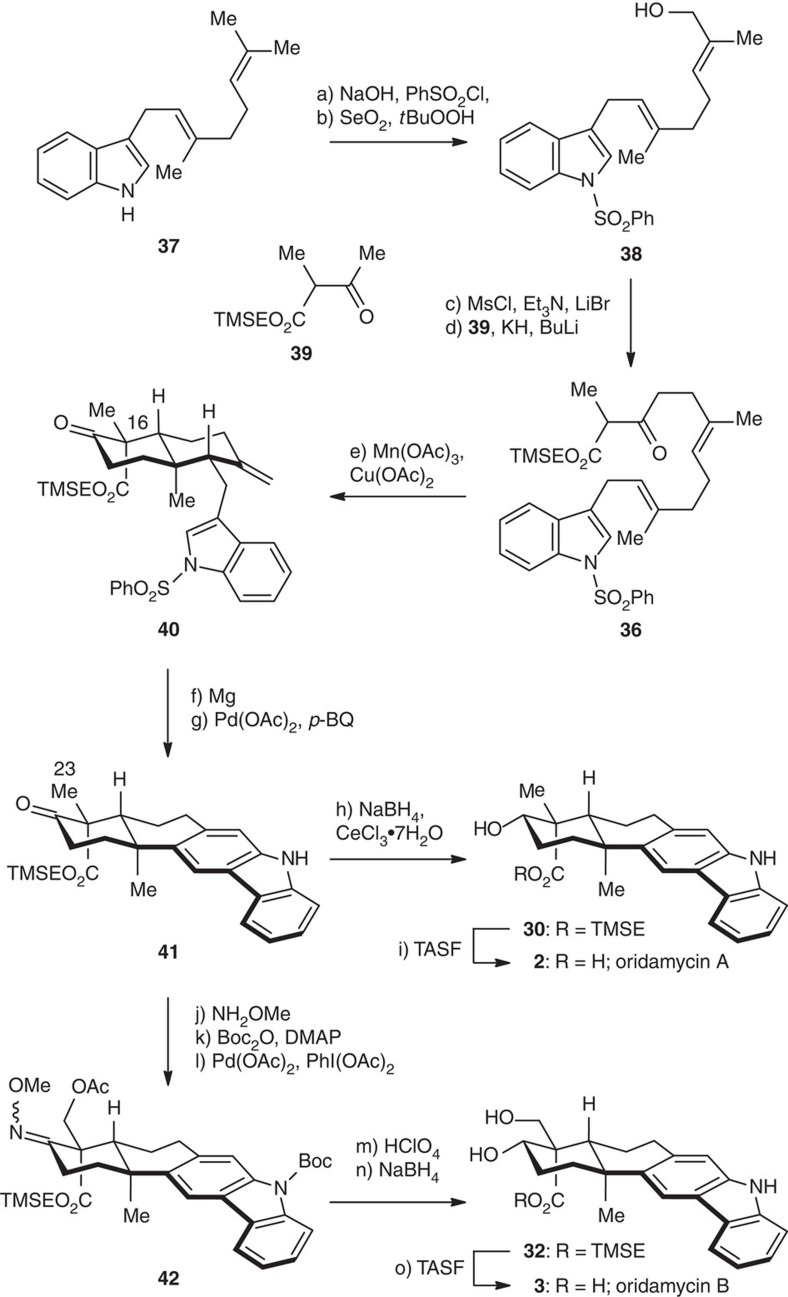
The oxidative Heck annulation approach towards the total syntheses of oridamycins A and B. Reagents and conditions: (a) PhSO_2_Cl (2.0 eq), Bu_4_NBr (10 mol%), aq. NaOH (50 wt%)/toluene (1:2), 22 °C, 3 h; (b) SeO_2_ (20 mol%), *t*BuOOH (2.0 eq), CH_2_Cl_2_, 0 °C, 10 h, 78% (two steps); (c) Et_3_N (1.5 eq), MsCl (1.2 eq), LiBr (5.0 eq), THF, 0 °C, 30 min; (d) **39**, (1.0 eq), KH (1.2 eq), BuLi (1.2 eq), THF/HMPA (5:1), 0 °C, 3 h, 80% (two steps); (e) Mn(OAc)_3_·2H_2_O (2.0 eq), Cu(OAc)_2_·H_2_O (1.0 eq), DMSO, 22 °C, 12 h, 52%; (f) Mg (3.0 eq), NH_4_Cl (1.2 eq), MeOH, 22 °C, 1 h, 96%; (g) Pd(OAc)_2_ (20 mol%), *p*-benzoquinone (2.5 eq), AcOH/toluene (1:4), 50 °C, 2 h, 65%; (h) NaBH_4_ (3.0 eq), CeCl_3_·7H_2_O (3.0 eq), MeOH, 0 °C, 30 min; (i) TASF (2.0 eq), DMF, 50 °C, 2 h, 85% (two steps); (j) NH_2_OMe·HCl (2.0 eq), pyridine, 22 °C, 2 h, 93%; (k) Boc_2_O (2.0 eq), 4-DMAP (2.0 eq), THF, 22 °C, 1 h, 89%; (l) Pd(OAc)_2_ (20 mol%), PhI(OAc)_2_ (1.1 eq), Ac_2_O/AcOH (1:1), 110 °C, 1 h, 81%; (m) aq. HClO_4_ (6.0 M)/acetone (1:3), 22 °C, 8 h; (n) NaBH_4_ (3.0 eq), MeOH, 0 °C, 30 min, 54% (two steps); (o) TASF (2.0 eq), DMF, 50 °C, 2 h, 91%.

**Figure 9:**
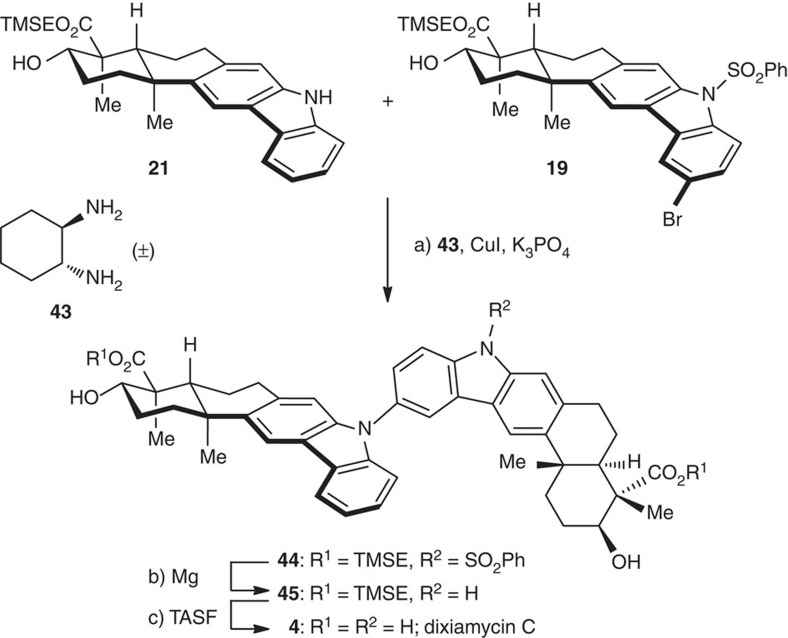
Total synthesis of dixiamycin C. Reagents and conditions: (a) CuI (1.5 eq), **21** (1.0 eq), **19** (1.5 eq), (±)-**43** (3.0 eq), K_3_PO_4_ (7.5 eq), 1,4-dioxane, 110 °C, 12 h; (b) Mg (6.0 eq), MeOH, 22 °C, 1 h, 51% for the two steps; (c) TASF (4.0 eq), DMF, 50 °C, 5 h, 96%.

**Figure 10:**
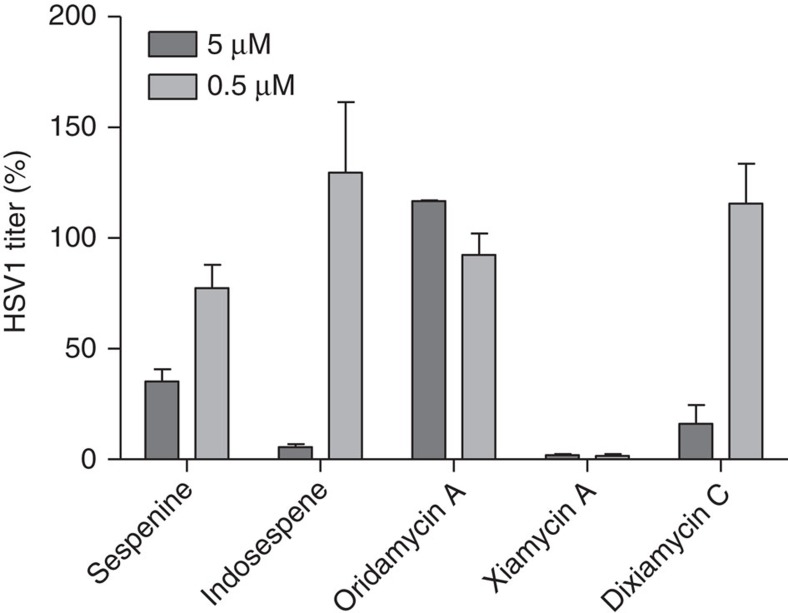
Effects of the synthetic compounds on HSV-1 propagation. Vero cells were infected with HSV1 (strain KOS-ATCC VR1493) for 3 h and then treated with different concentrations of compounds for 20 h. HSV-1 in the culture supernatants were then quantified by using TCID_50_ assay. Virus titres were presented as the percentages of the mock DMSO treatment. Data represent means±s.e.m. of six individual infections.

The activities of synthetic xiamycin A (**1**), oridamycins A (**2**), dixiamycin C (**4**), indosespene (**6**) and sespenine^14^,^16^ against HSV-1 and hepatitis C virus (HCV) were evaluated. Notably, xiamycin A was reported to possess moderate anti-HIV activity but no significant cytotoxicity against a number of human tumour cell lines^16^, and a structurally relevant carbazole natural product, named tubingensin A, was found to exhibit anti-HSV-1 activity (without details disclosed)^69^. We treated HSV-1 (KOS strain)-infected vero cells with 5.0 and 0.50 μM solutions of the compounds for 20 h and then quantified the infectious HSV-1 titres in the supernatants by using TCID_50_ assay^70^. As shown in Fig. 10, **1**, **4**, **6** and sespenine can suppress HSV-1 propagation in a dose-dependent manner. Notably, xiamycin A (**1**) is the most potent compound that reduces >95% of virus propagation at a concentration of 0.50 μM. Indosespene (**6**) significantly inhibits HSV-1 propagation at the concentration of 5.0 μM and displays essentially no antiviral activity at the concentration of 0.50 μM. Dixiamycin C (**4**) and sespenine show moderate inhibitory activity against HSV-1 at 5.0 μM but no activity at 0.50 μM. For the anti-HCV test (using a replicon assay), only dixiamycin C (**6**) exhibited inhibitory activity at a high concentration (50 μM, see Supplementary Fig. 81). It should be mentioned that these compounds did not exhibit cytotoxicity against the cells used for both anti-HSV-1 and HCV tests (see Supplementary Fig. 82).

## Discussion

We have accomplished the total synthesis of indolosesquiterpenoids xiamycin A, oridamycins A and B, dixiamycin C and indosespene. Two unified strategies for the framework construction and a series of transition-metal-mediated reactions formed the basis of the synthesis. Antiviral tests of the synthetic natural products revealed that xiamycin A exhibits a potent inhibitory activity against HSV-1.

## Methods

### General

All reactions were carried out under an argon atmosphere with dry solvents under anhydrous conditions, unless otherwise noted. Tetrahydrofuran (THF), toluene and 1,4-dioxane were distilled immediately before use from sodium-benzophenone ketyl. Methylene chloride (CH_2_Cl_2_), *N*,*N*-dimethylformamide (DMF), dimethyl sulfoxide (DMSO), hexamethylphosphoramide (HMPA), triethylamine (Et_3_N), *N*,*N*-diisopropylethylamine (*i*Pr_2_NEt) and pyridine were distilled from calcium hydride and stored under an argon atmosphere. Methanol (MeOH) was distilled from magnesium and stored under an argon atmosphere. Acetone was dried over drierite and distilled before use. Reagents were purchased at the highest commercial quality and used without further purification, unless otherwise stated. Solvents for chromatography were used as supplied by Titan Chemical. Reactions were monitored by thin layer chromatography (TLC) carried out on S-2 0.25 mm E. Merck silica gel plates (60F-254) using UV light as a visualizing agent and aqueous ammonium cerium nitrate/ammonium molybdate or basic aqueous potassium permanganate as a developing agent. E. Merck silica gel (60, particle size 0.040–0.063 mm) was used for flash column chromatography. Preparative thin layer chromatography separations were carried out on 0.25 or 0.50 mm E. Merck silica gel plates (60F-254). NMR spectra were recorded on Bruker AV-400, DRX-600 or Agilent 500/54/ASP instrument and calibrated by using residual undeuterated chloroform (*δ*_H_=7.26 p.p.m.) and CDCl_3_ (*δ*_C_=77.16 p.p.m.), or undeuterated methanol (*δ*_H_=3.31 p.p.m.) and methanol-d_4_ (*δ*_C_=49.00 p.p.m.), as internal references. IR spectra were recorded on a Thermo Scientific Nicolet 380 FT-IR spectrometer. Melting points (m.p.) are uncorrected and were recorded on a SGW X-4 apparatus. High-resolution mass spectra (HRMS) were recorded on a Bruker APEXIII 7.0 Tesla ESI-FT mass spectrometer.

For ^1^H and ^13^C NMR spectra of compounds, see Supplementary Figs 1–70. For the comparisons of ^1^H and ^13^C NMR spectra of the natural and synthetic natural products, see Supplementary Figs 71–80. For the comparisons of ^1^H and ^13^C NMR spectroscopic data of the natural and synthetic natural products, see Supplementary Tables 1–10. For the experimental procedures, and spectroscopic and physical data of compounds, the antiviral assay and cytotoxicity assays, and the crystallographic data of compounds **1**, **6** and **40**, see Supplementary Methods.

## Author contributions

A.L. directed the project. A.L., Z.M., H.Y. and D.J.E. conceived the synthetic route. Z.M., H.Y., H.C. and M.W. conducted the synthetic work. L.L. and W.T. conducted the biological work. A.L., Z.M., H.Y., H.C., P.Y., D.J.E., L.L., W.T. and J.Z. analysed the results. A.L., D.J.E., L.L. and J.Z. wrote the manuscript.

## Additional information

**How to cite this article:** Meng, Z. *et al*. Total synthesis and antiviral activity of indolosesquiterpenoids from the xiamycin and oridamycin families. *Nat. Commun.* 6:6096 doi: 10.1038/ncomms7096 (2015).

**Accession codes:** The X-ray crystallographic coordinates for structures (**1**, **6** and **40**) reported in this Article have been deposited at the Cambridge Crystallographic Data Centre (CCDC), under deposition number CCDC 948203, 949054 and 948503. These data can be obtained free of charge from The Cambridge Crystallographic Data Centre via www.ccdc.cam.ac.uk/data_request/cif.

## Supplementary Material

Supplementary Information Supplementary Figures 1-82, Supplementary Tables 1-10, Supplementary Methods and Supplementary References
