# Supplementary material for: Total synthesis and antiviral activity of indolosesquiterpenoids from the xiamycin and oridamycin families
Source: Nat Commun. 2015 Feb 4;6:6096. doi: 10.1038/ncomms7096 (PMC4347019; doi:10.1038/ncomms7096)
Supplement: Supplementary Information — Supplementary Figures 1-82, Supplementary Tables 1-10, Supplementary Methods and Supplementary References [file ncomms7096-s1.pdf]

**Supplementary Figure 1. $^{1}$H NMR Spectrum of 12 (400 MHz, $CDCl_3$)**

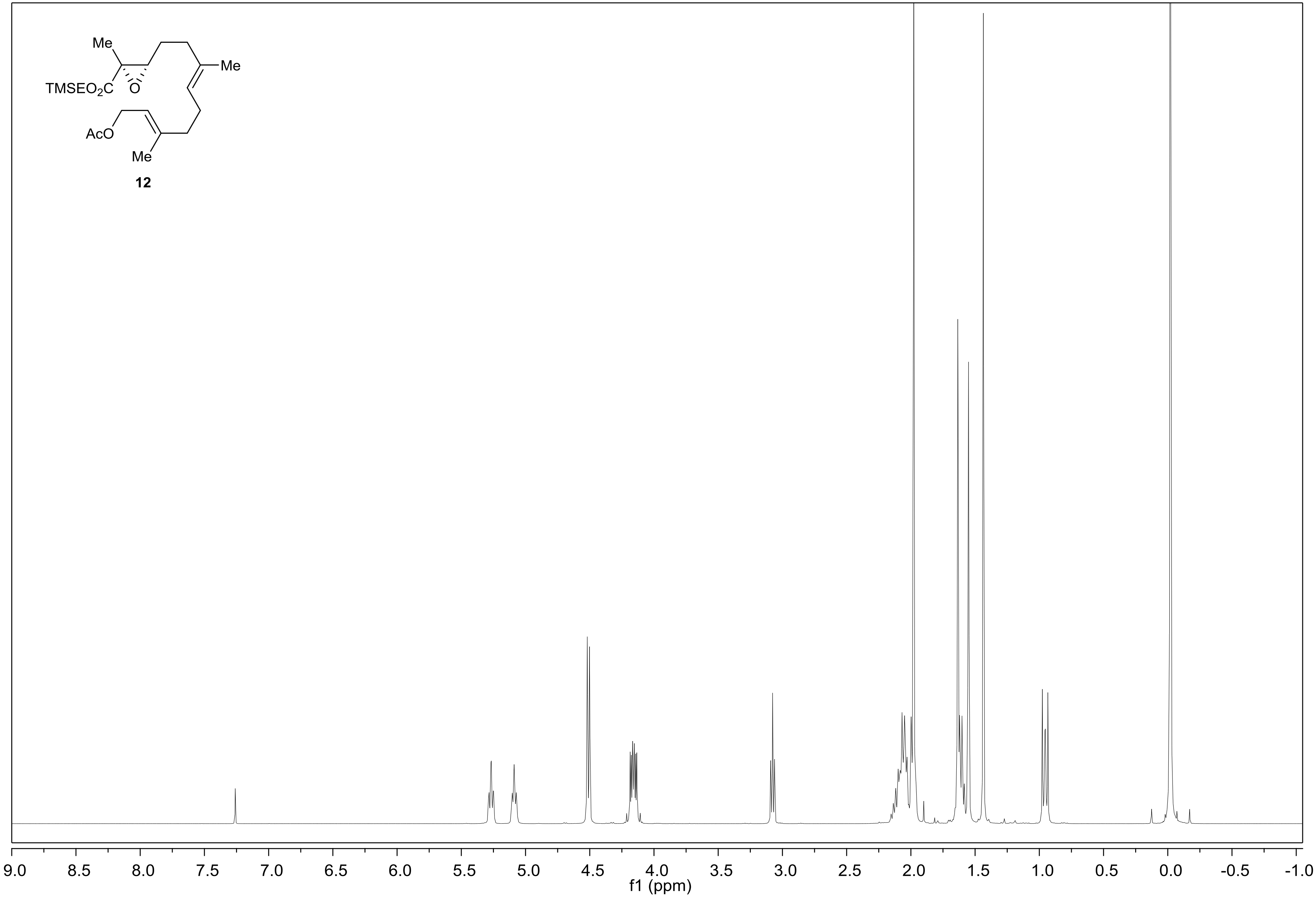

**Supplementary Figure 2. $^{13}$C NMR Spectrum of 12 (101 MHz, $CDCl_3$)**

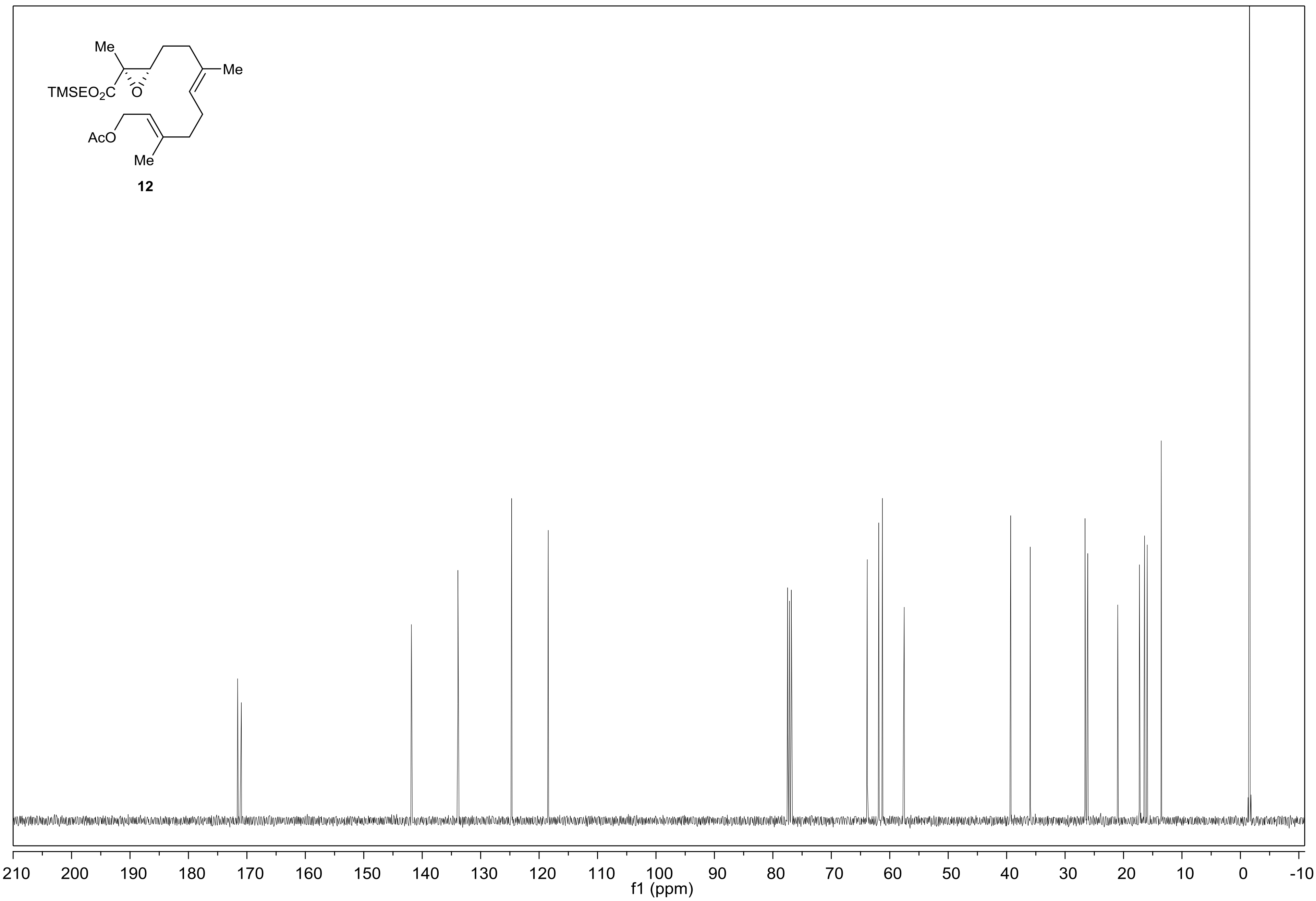

**Supplementary Figure 3. $^{1}$H NMR Spectrum of 14 (500 MHz, $CDCl_3$)**

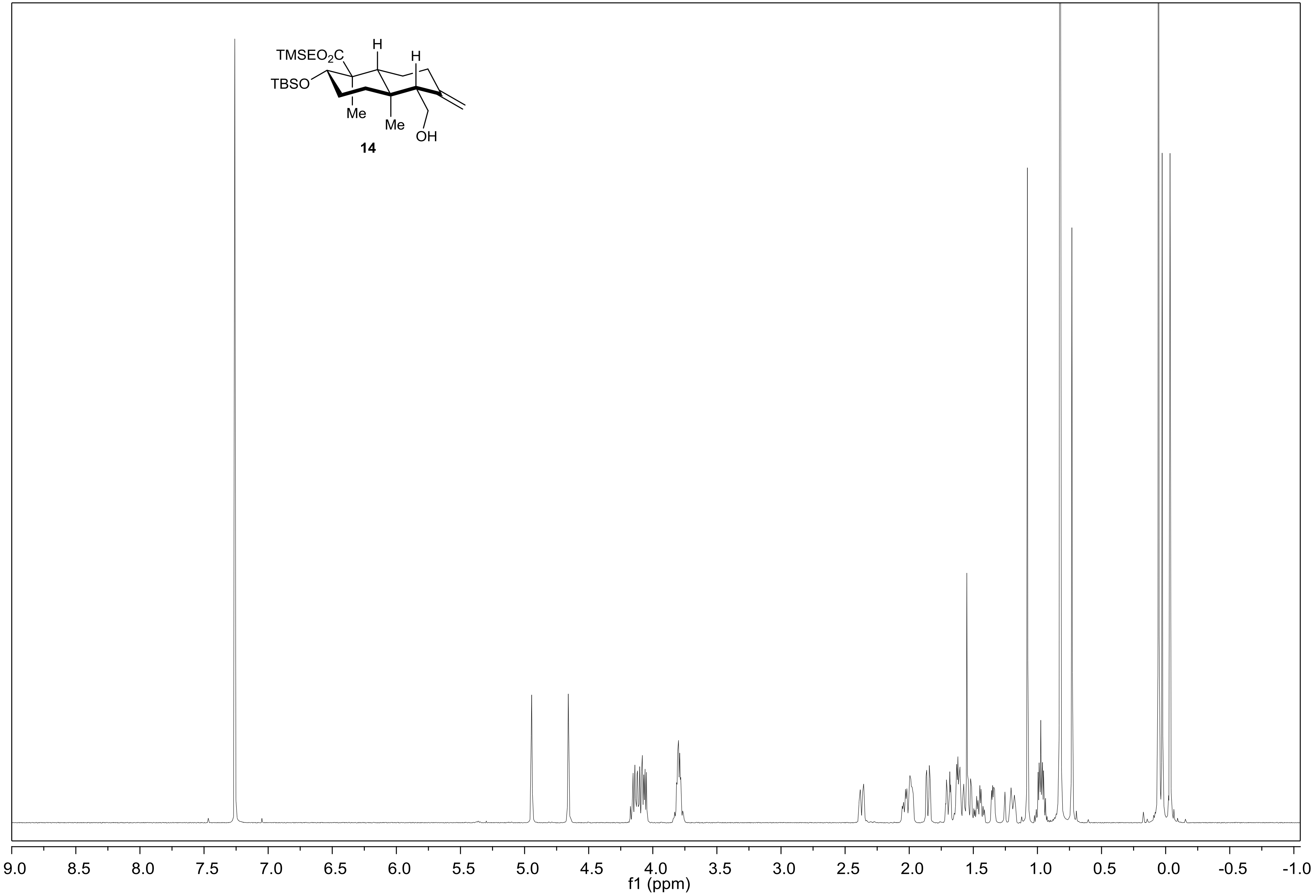

**Supplementary Figure 4. $^{13}$C NMR Spectrum of 14 (126 MHz, $CDCl_3$)**

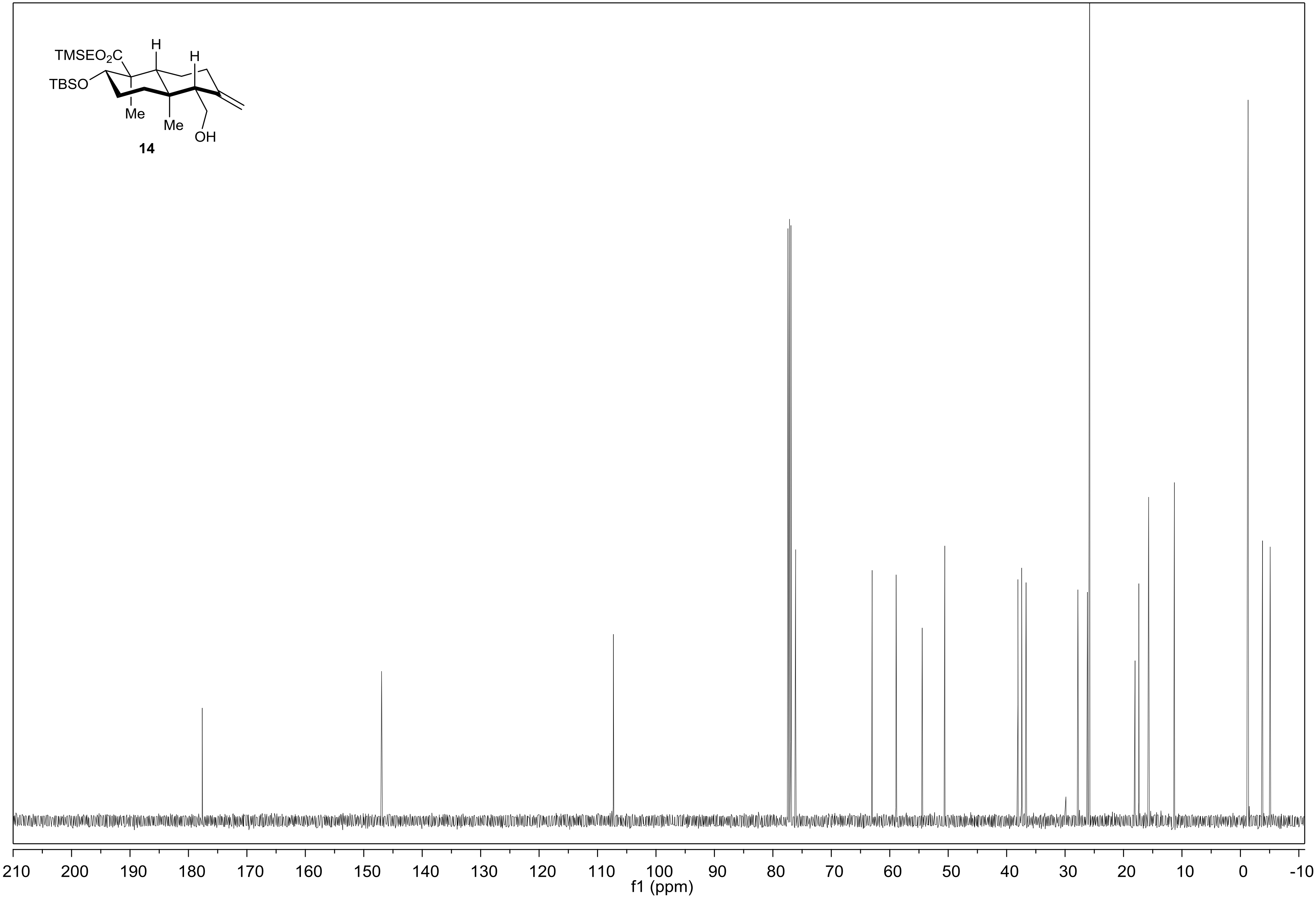

**Supplementary Figure 5. $^1$H NMR Spectrum of 14a (400 MHz, $CDCl_3$)**

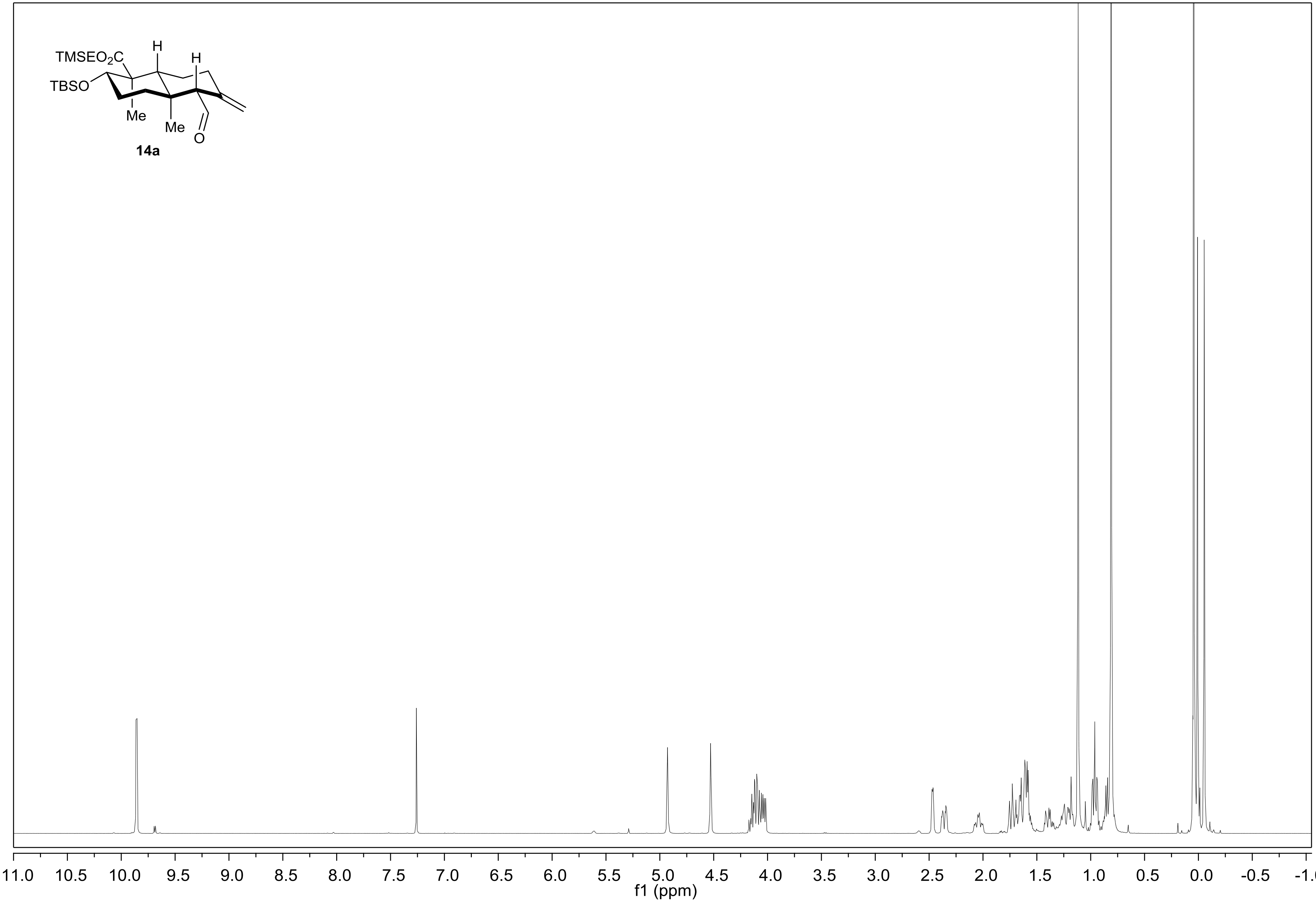

**Supplementary Figure 6. $^{13}$C NMR Spectrum of 14a (101 MHz, $CDCl_3$)**

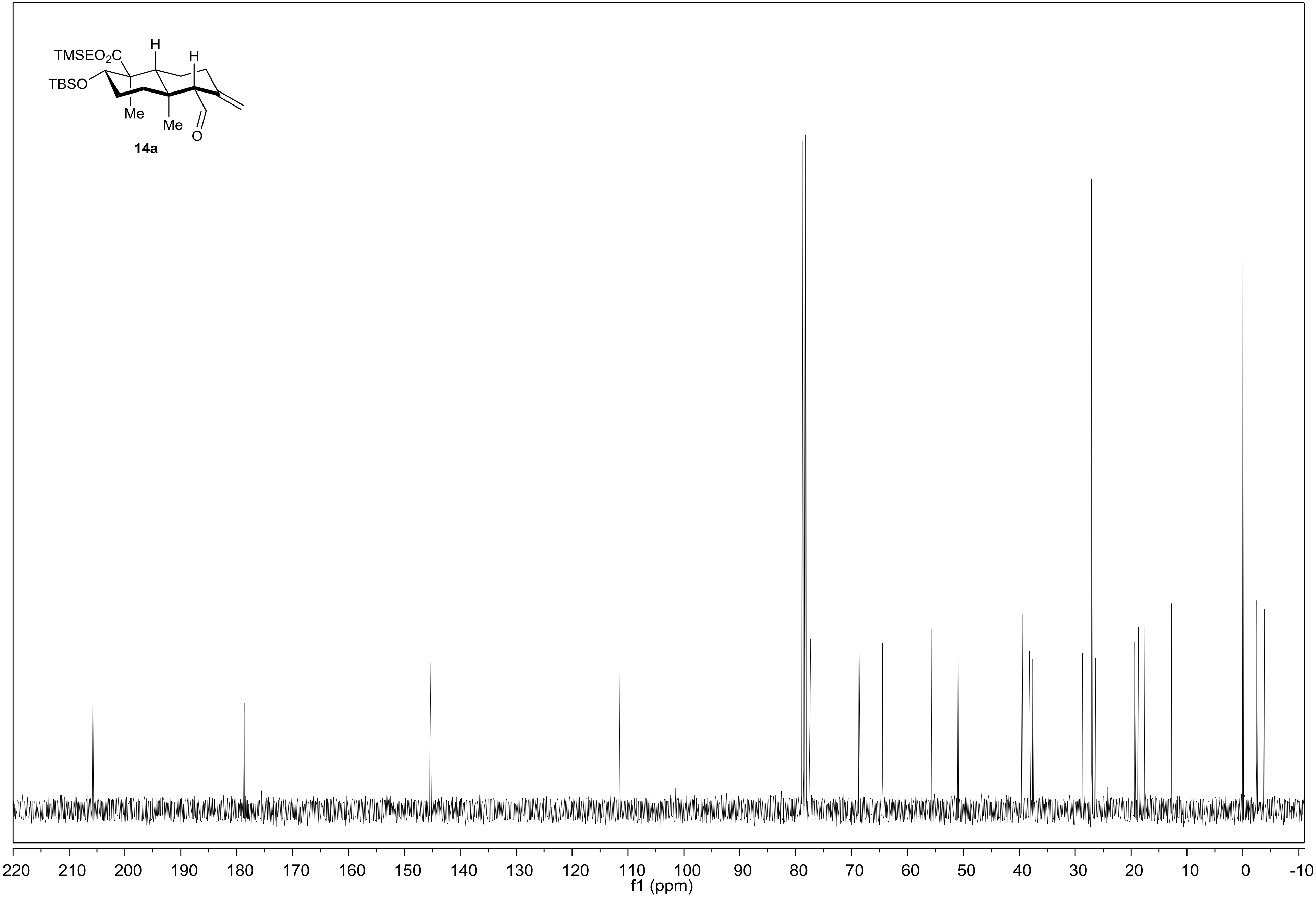

**Supplementary Figure 7. $^1H$ NMR Spectrum of 16 (400 MHz, $CDCl_3$)**

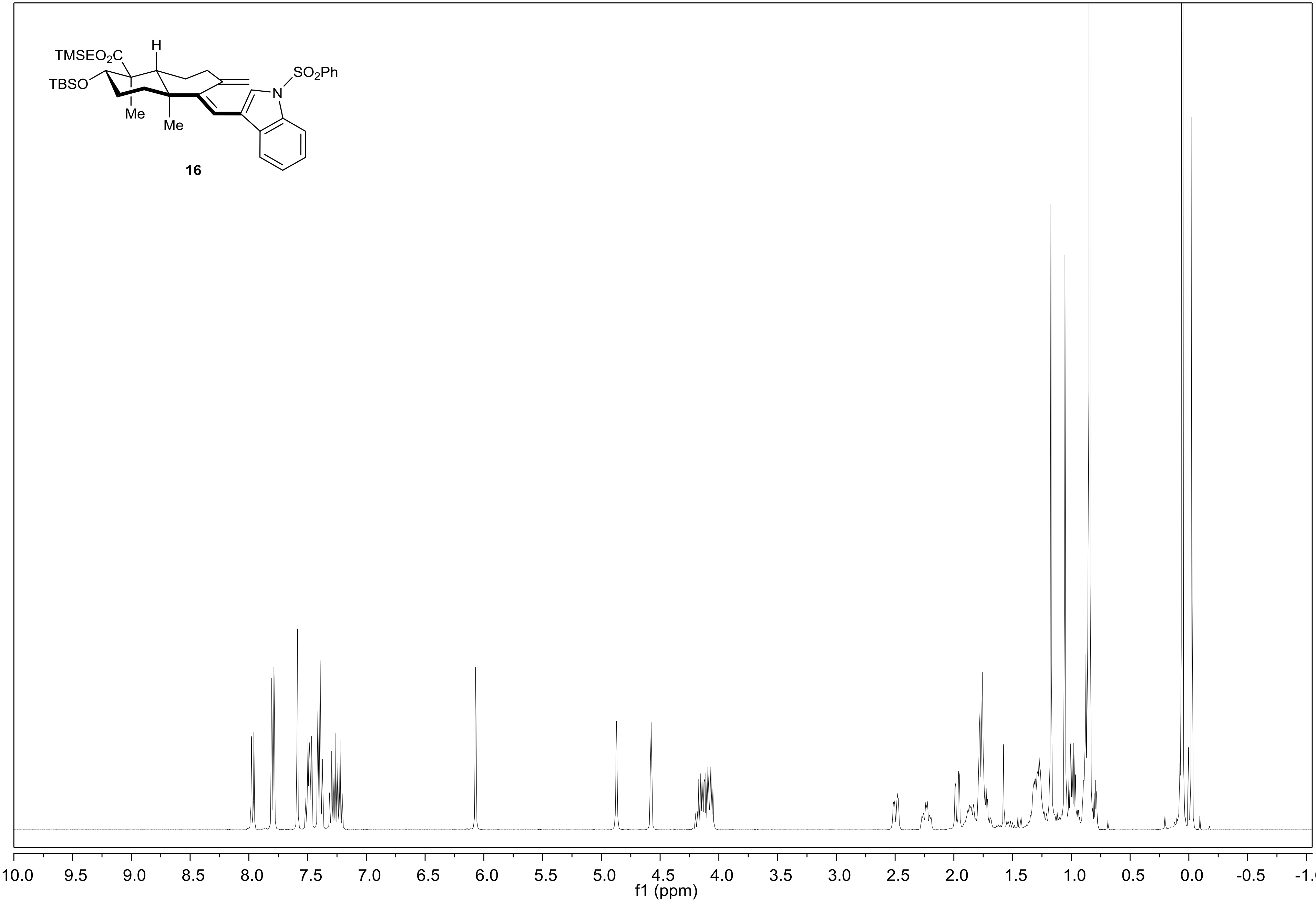

**Supplementary Figure 8. $^{13}$C NMR Spectrum of 16 (126 MHz, $CDCl_3$)**

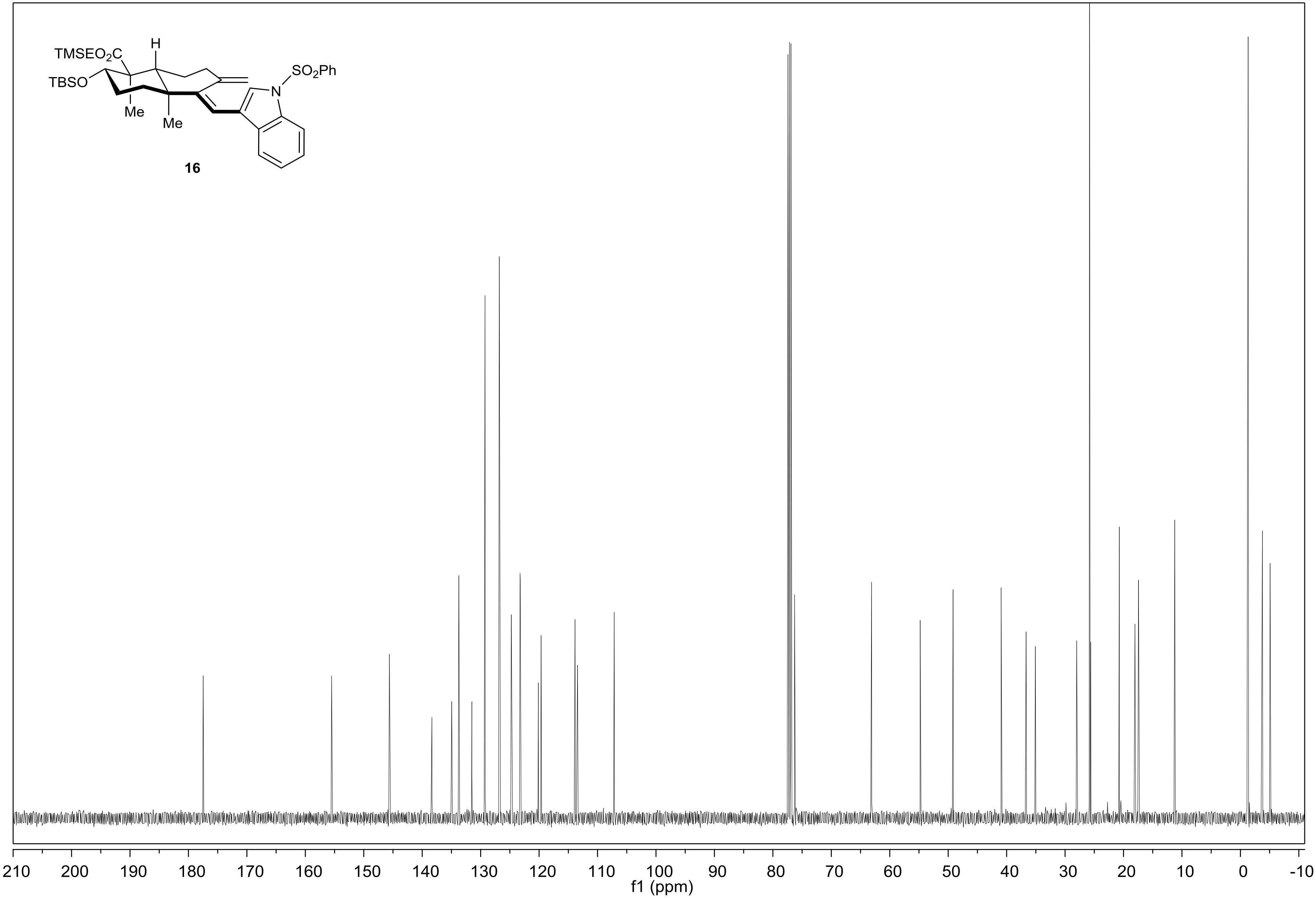

**Supplementary Figure 9. $^1$H NMR Spectrum of 17 (400 MHz, $CDCl_3$)**

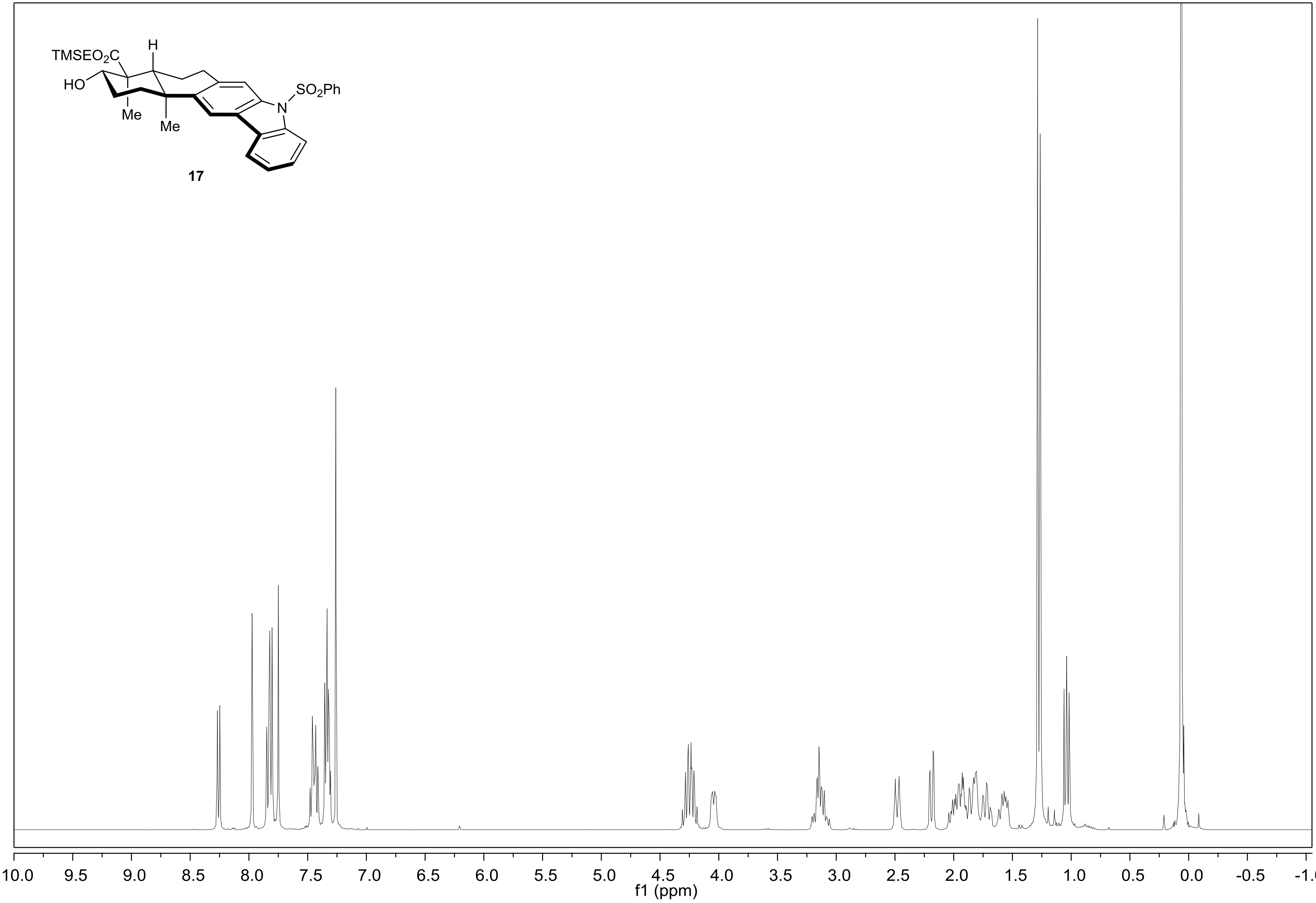

**Supplementary Figure 10. $^{13}$C NMR Spectrum of 17 (126 MHz, $CDCl_3$)**

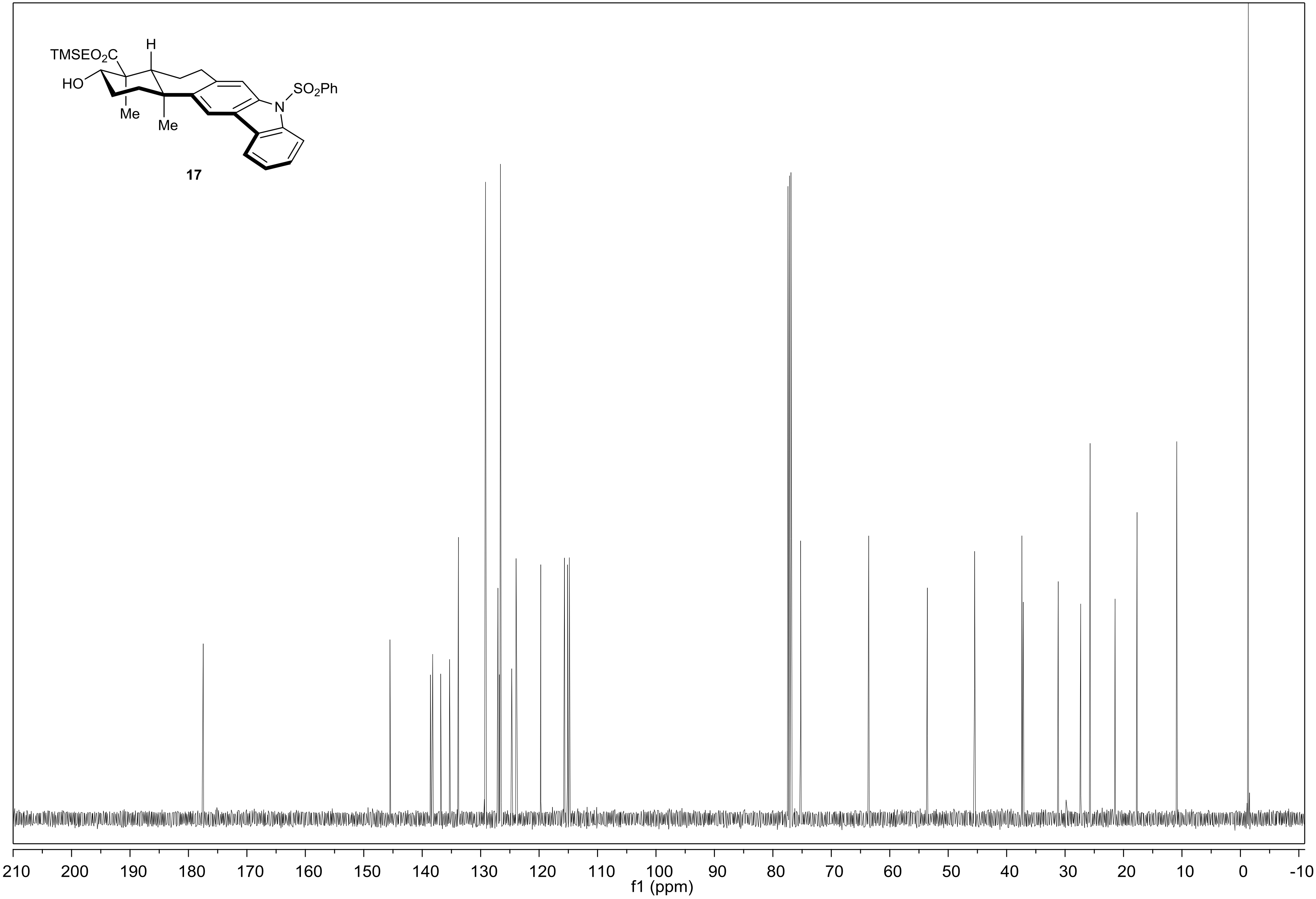

Supplementary Figure 11. $^{1}$H NMR Spectrum of 20 (500 MHz, $CDCl_3$)

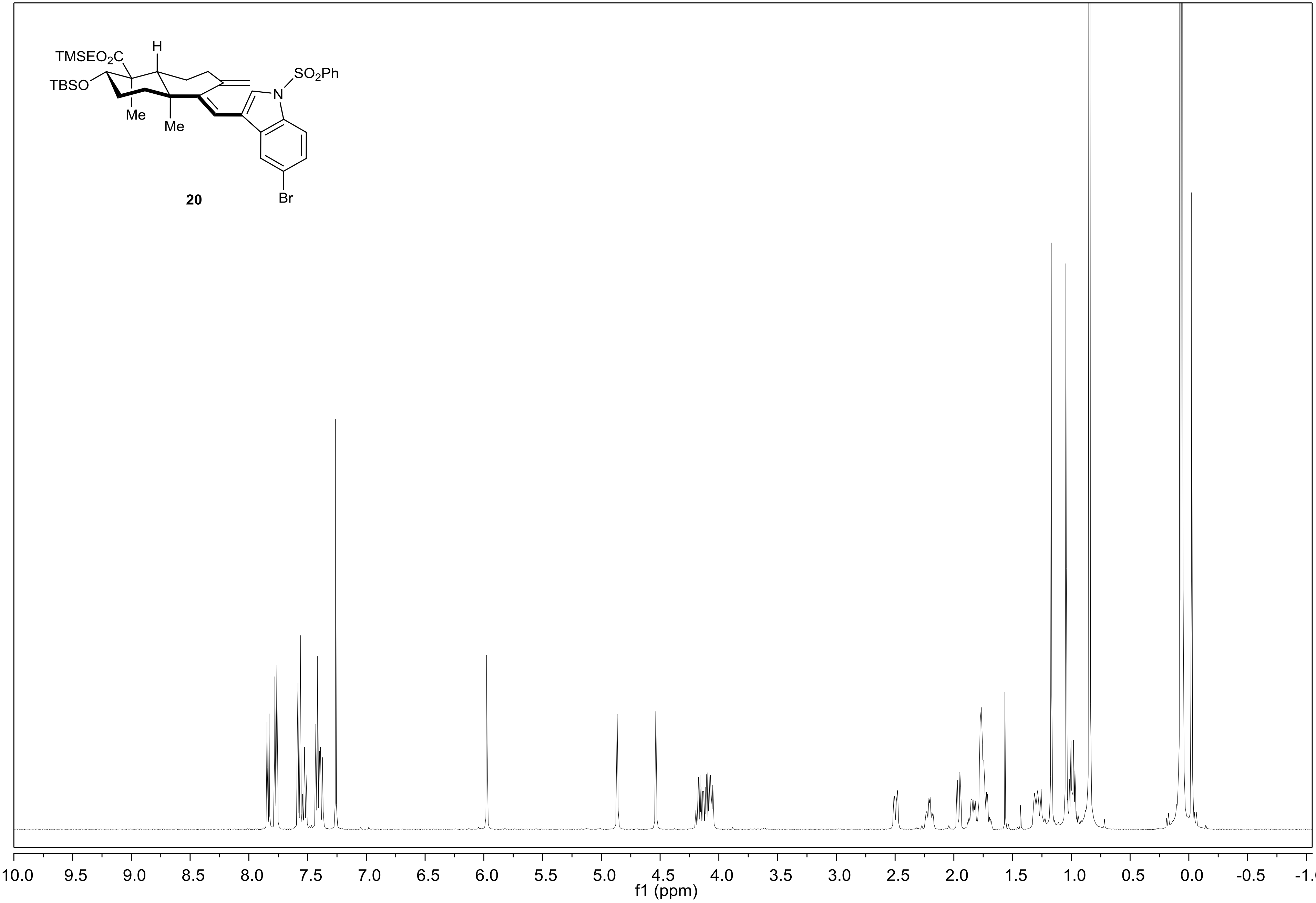

**Supplementary Figure 12. $^{13}$C NMR Spectrum of 20 (126 MHz, $CDCl_3$)**

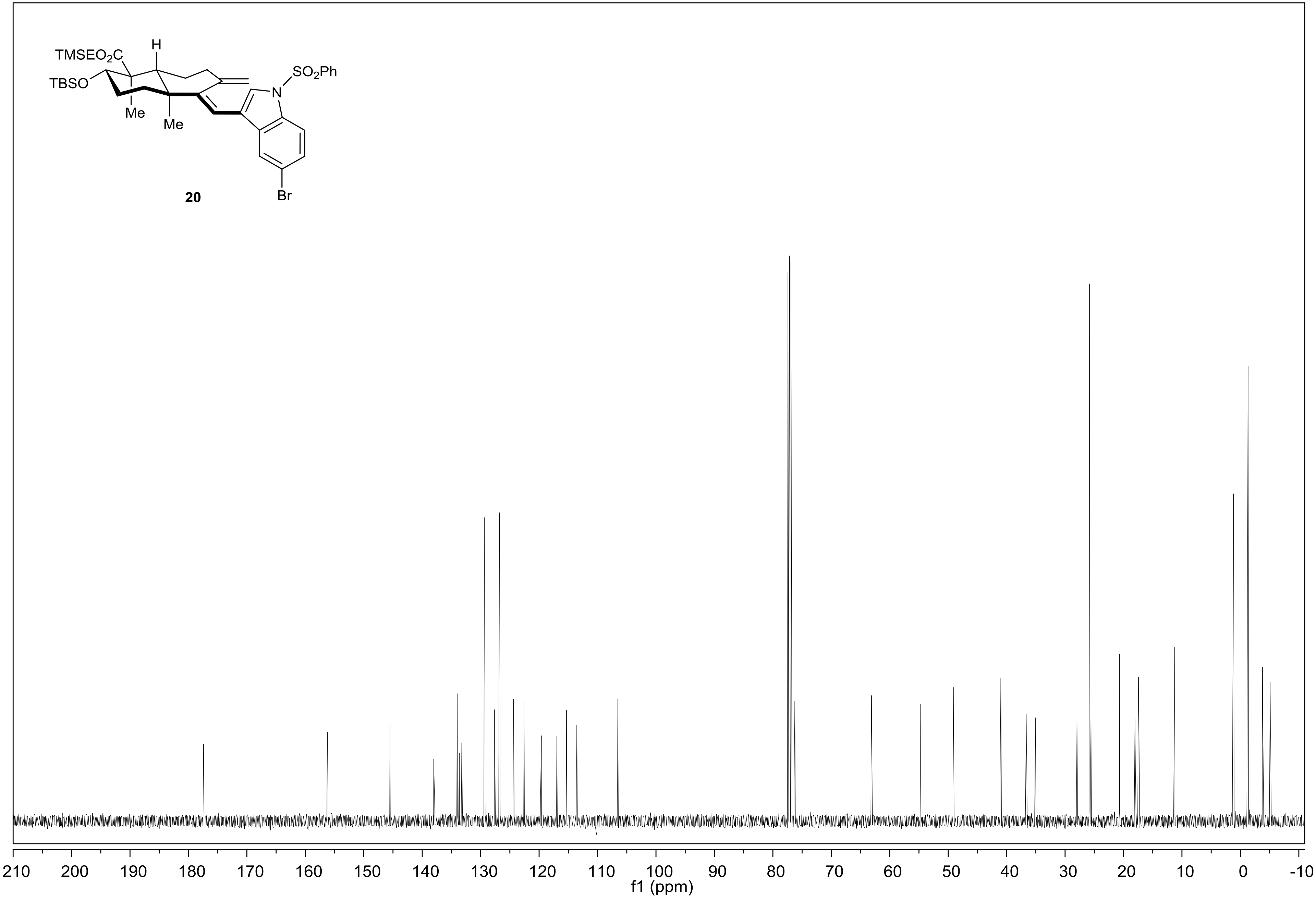

**Supplementary Figure 13.** $^{1}$**H NMR Spectrum of 19 (400 MHz, CDCl$_3$)**

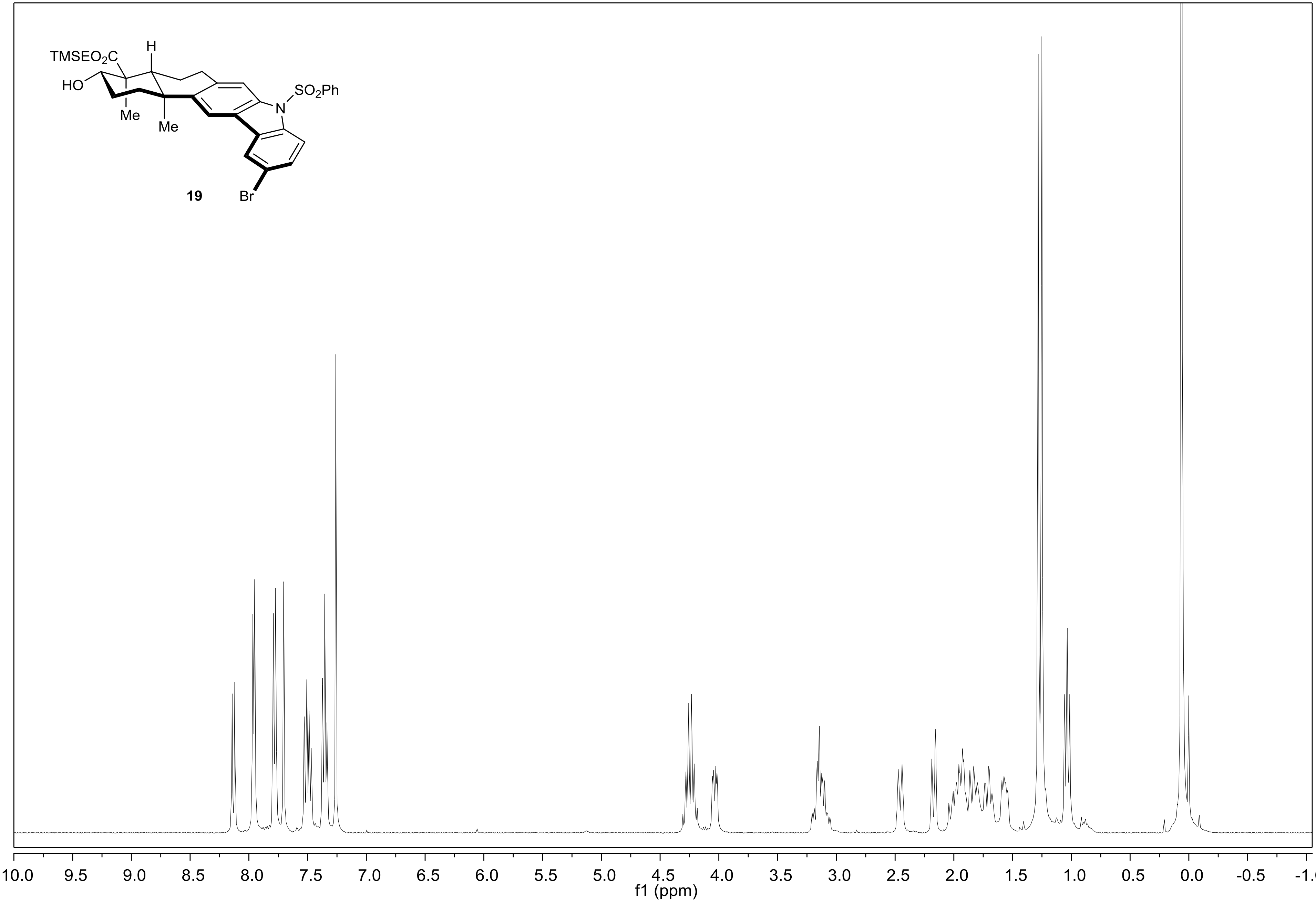

**Supplementary Figure 14.** $^{13}$**C NMR Spectrum of 19 (126 MHz, CDCl$_3$)**

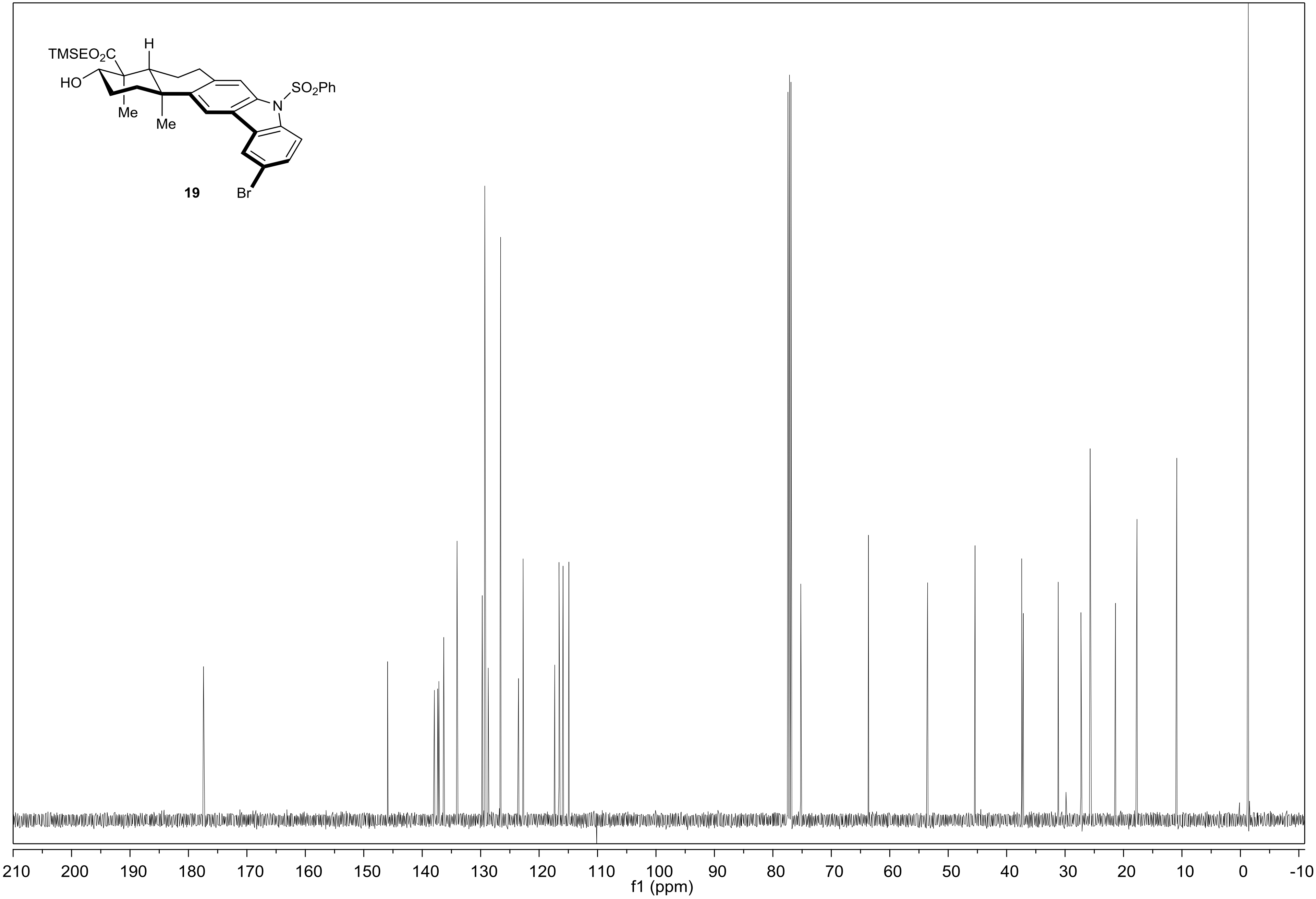

**Supplementary Figure 15. $^1$H NMR Spectrum of 21 (400 MHz, $CDCl_3$)**

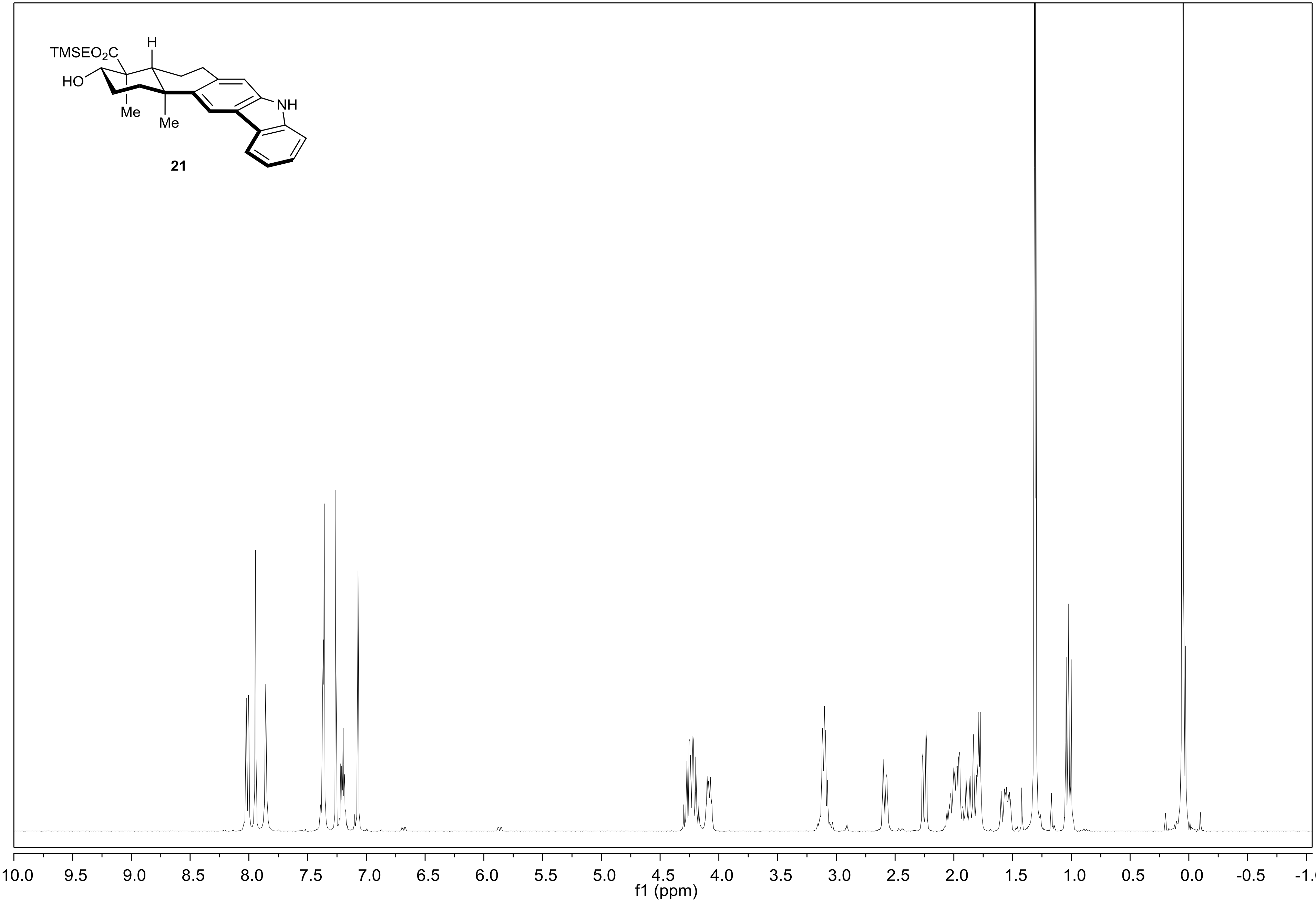

**Supplementary Figure 16. $^{13}$C NMR Spectrum of 21 (126 MHz, $CDCl_3$)**

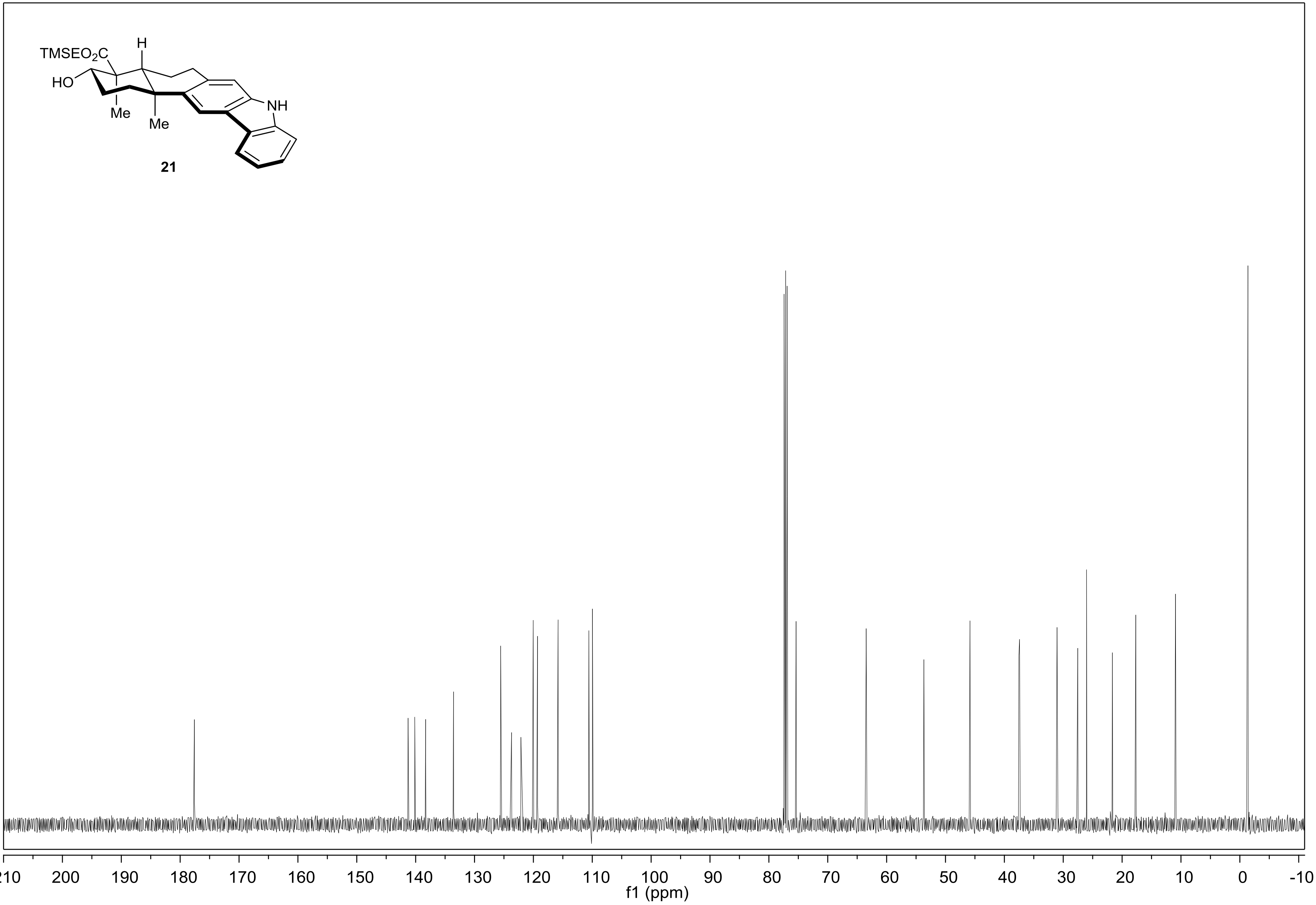

**Supplementary Figure 17. $^{1}$H NMR Spectrum of 1 (400 MHz, methanol-$d_4$)**

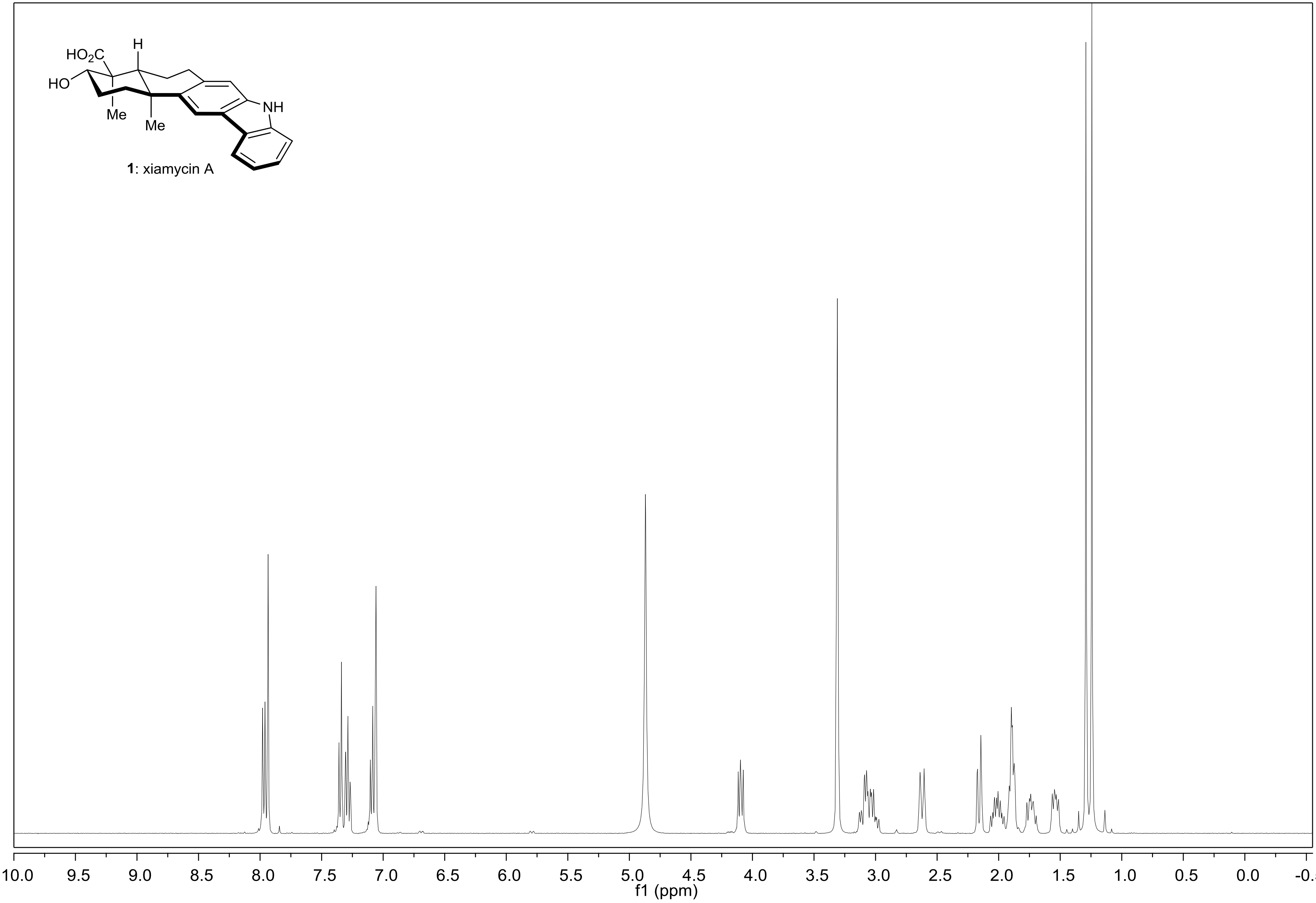

**Supplementary Figure 18. $^{13}$C NMR Spectrum of 1 (101 MHz, methanol-d$_4$)**

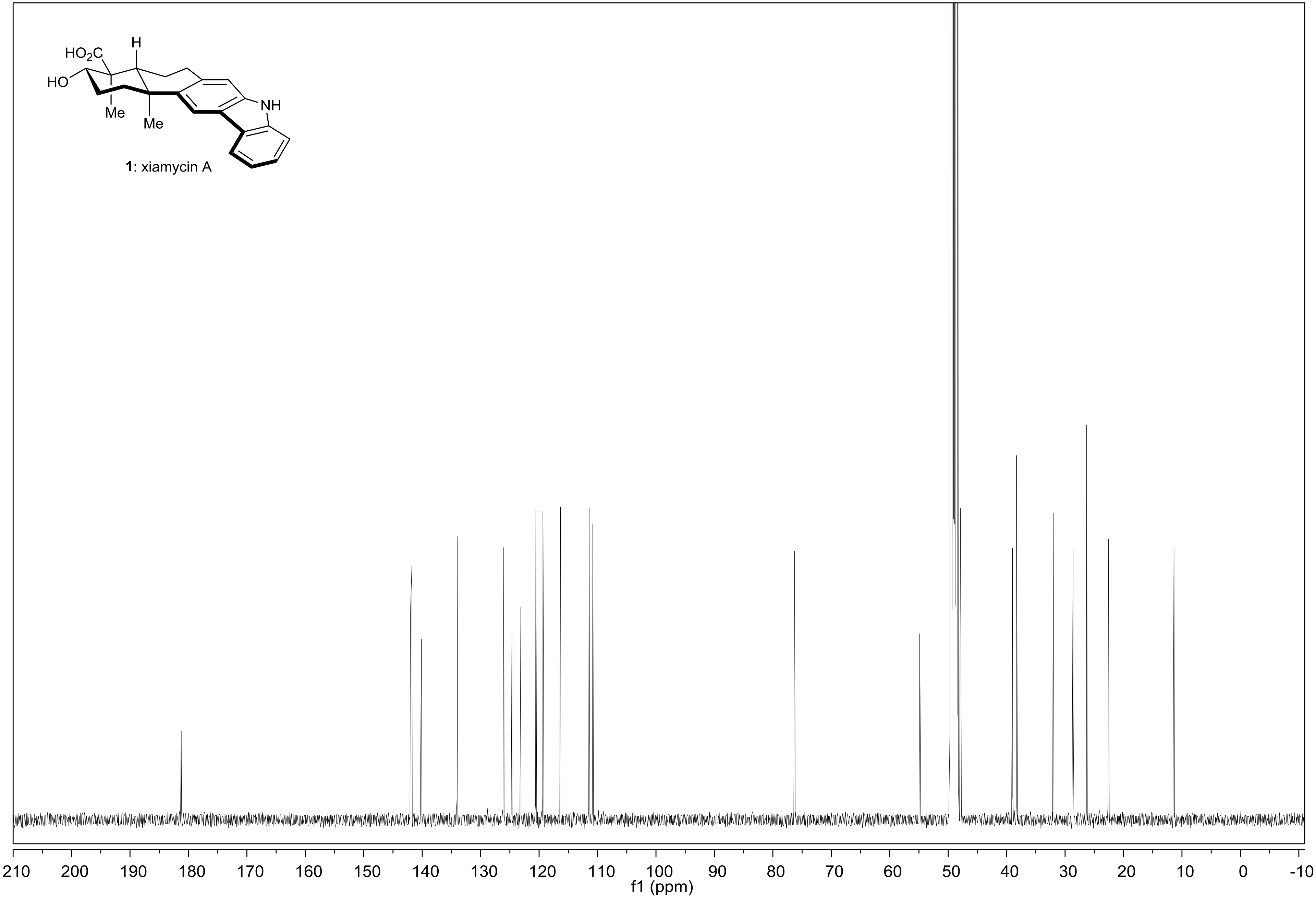

**Supplementary Figure 19. $^{1}H$ NMR Spectrum of 25 (400 MHz, $CDCl_3$)**

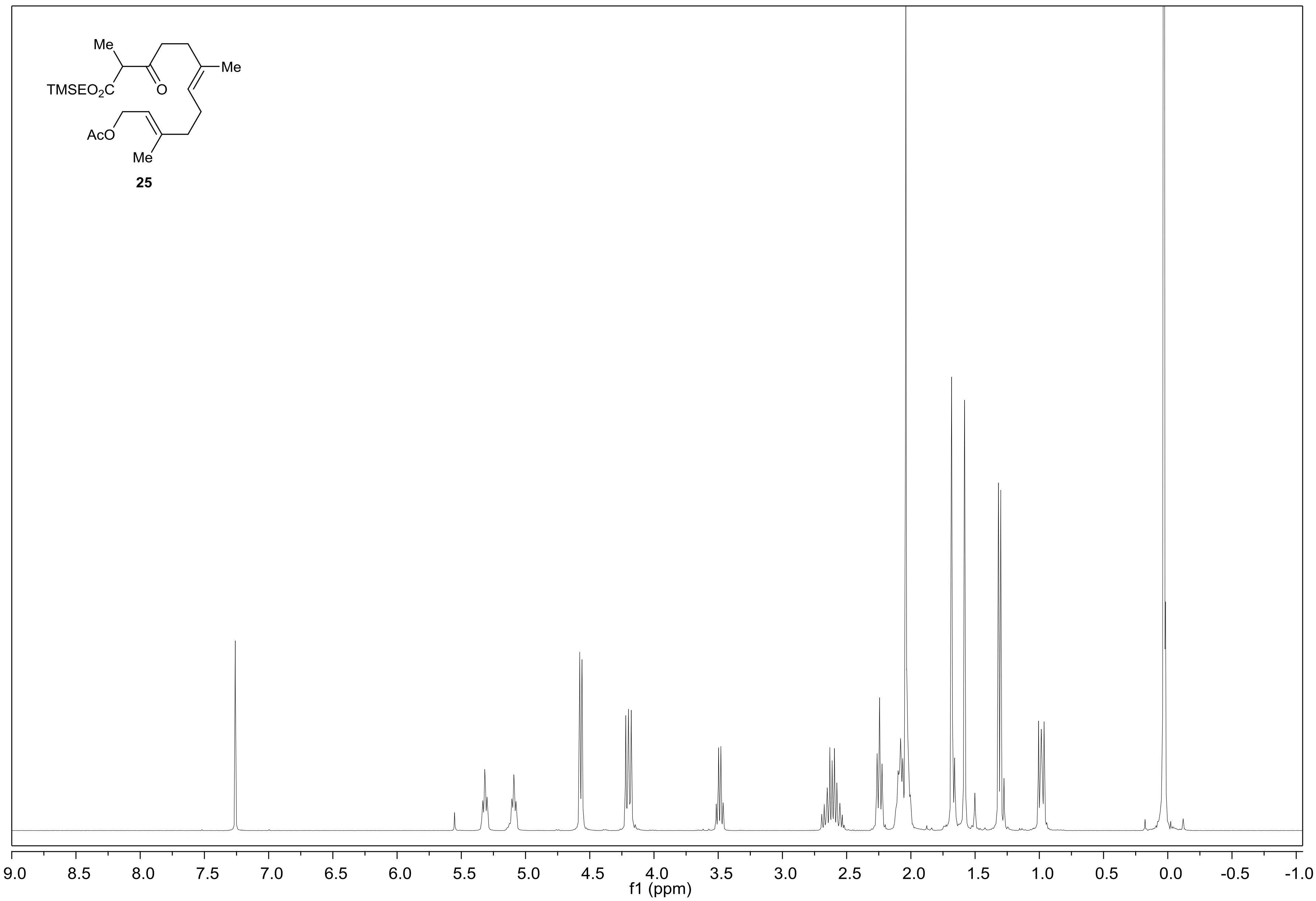

**Supplementary Figure 20. $^{13}$C NMR Spectrum of 25 (101 MHz, $CDCl_3$)**

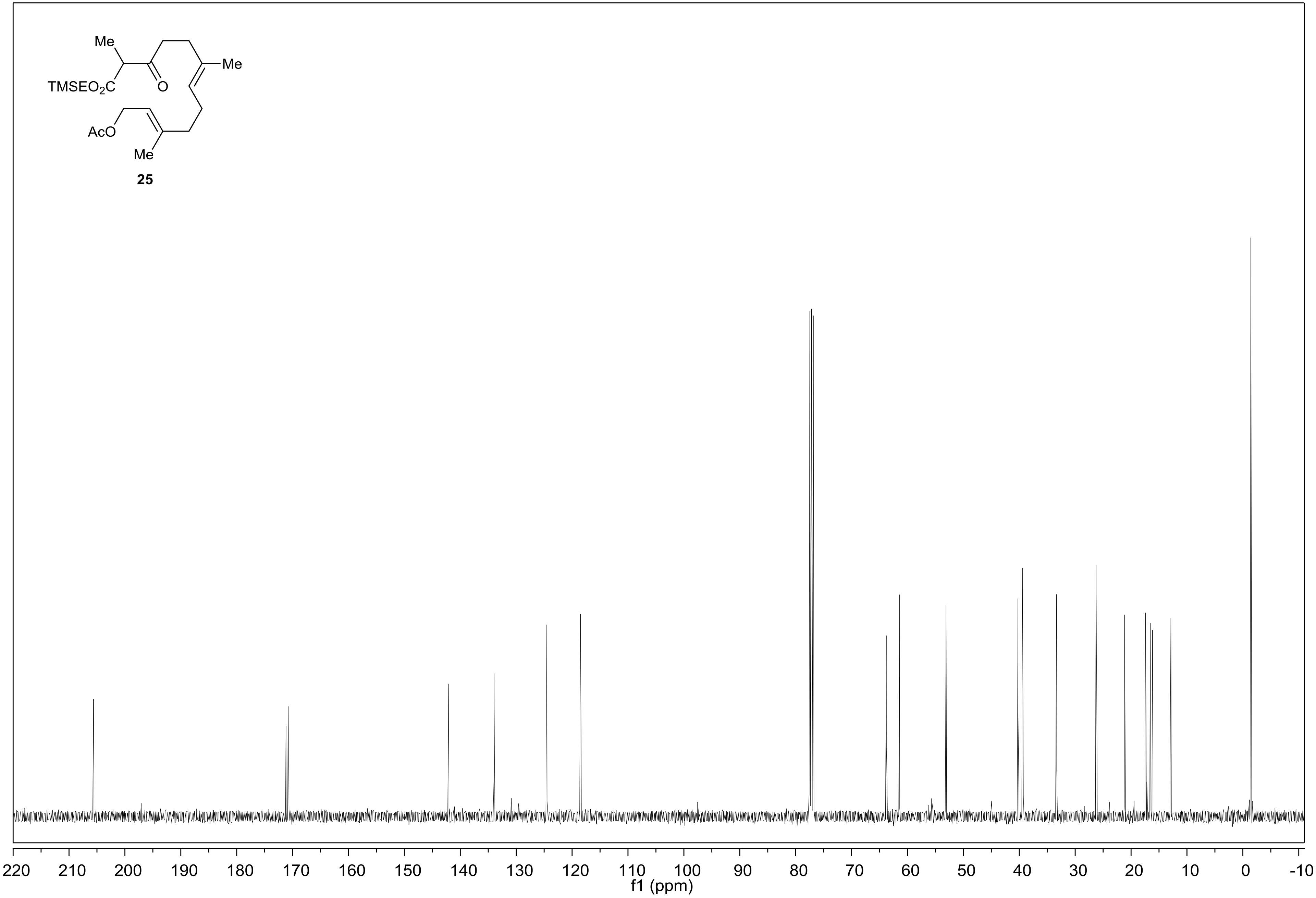

**Supplementary Figure 21. $^{1}$H NMR Spectrum of 26 (400 MHz, $CDCl_3$)**

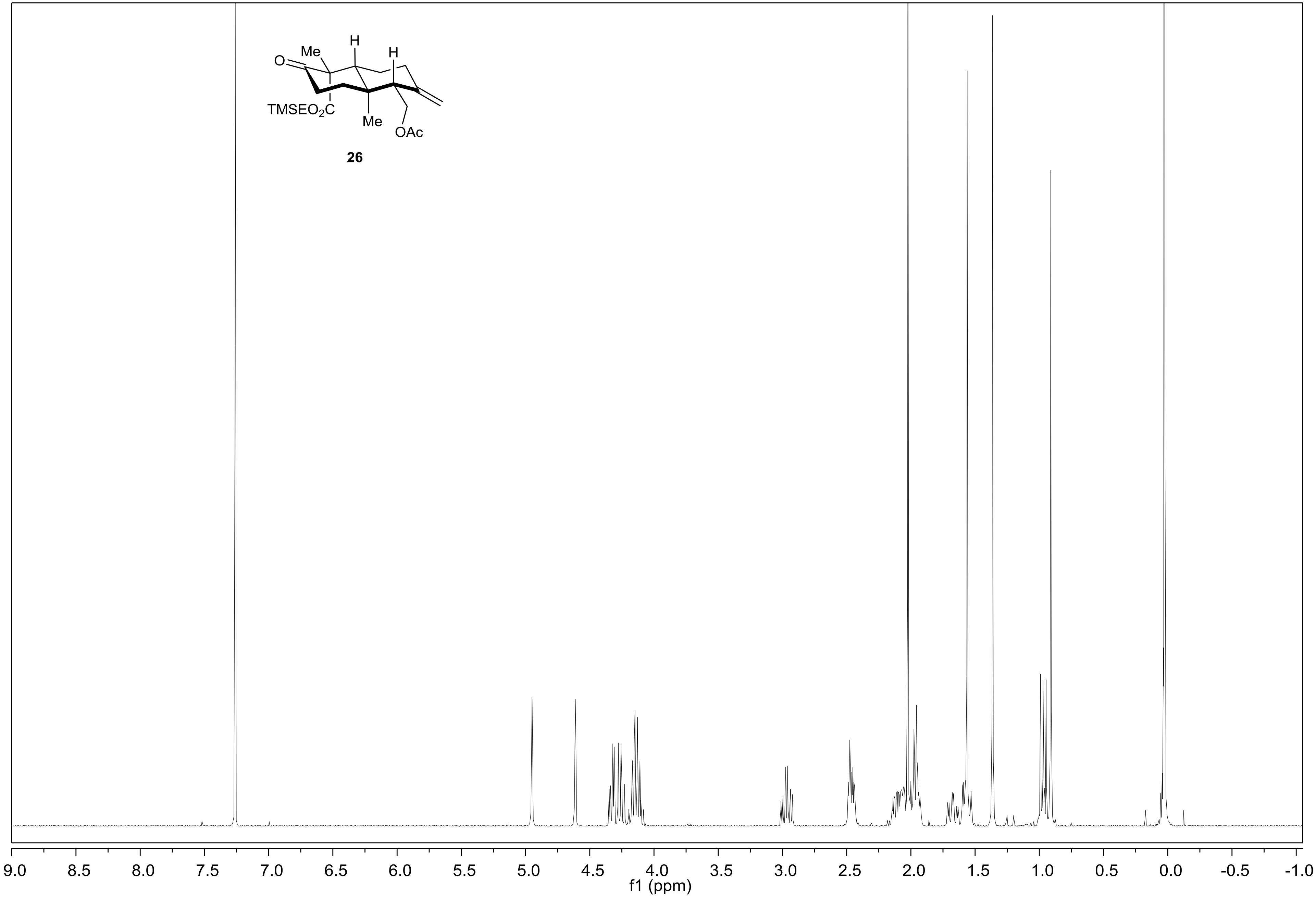

**Supplementary Figure 22. $^{13}$C NMR Spectrum of 26 (101 MHz, $CDCl_3$)**

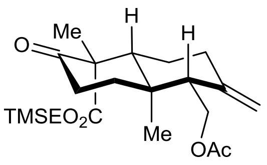


**26**

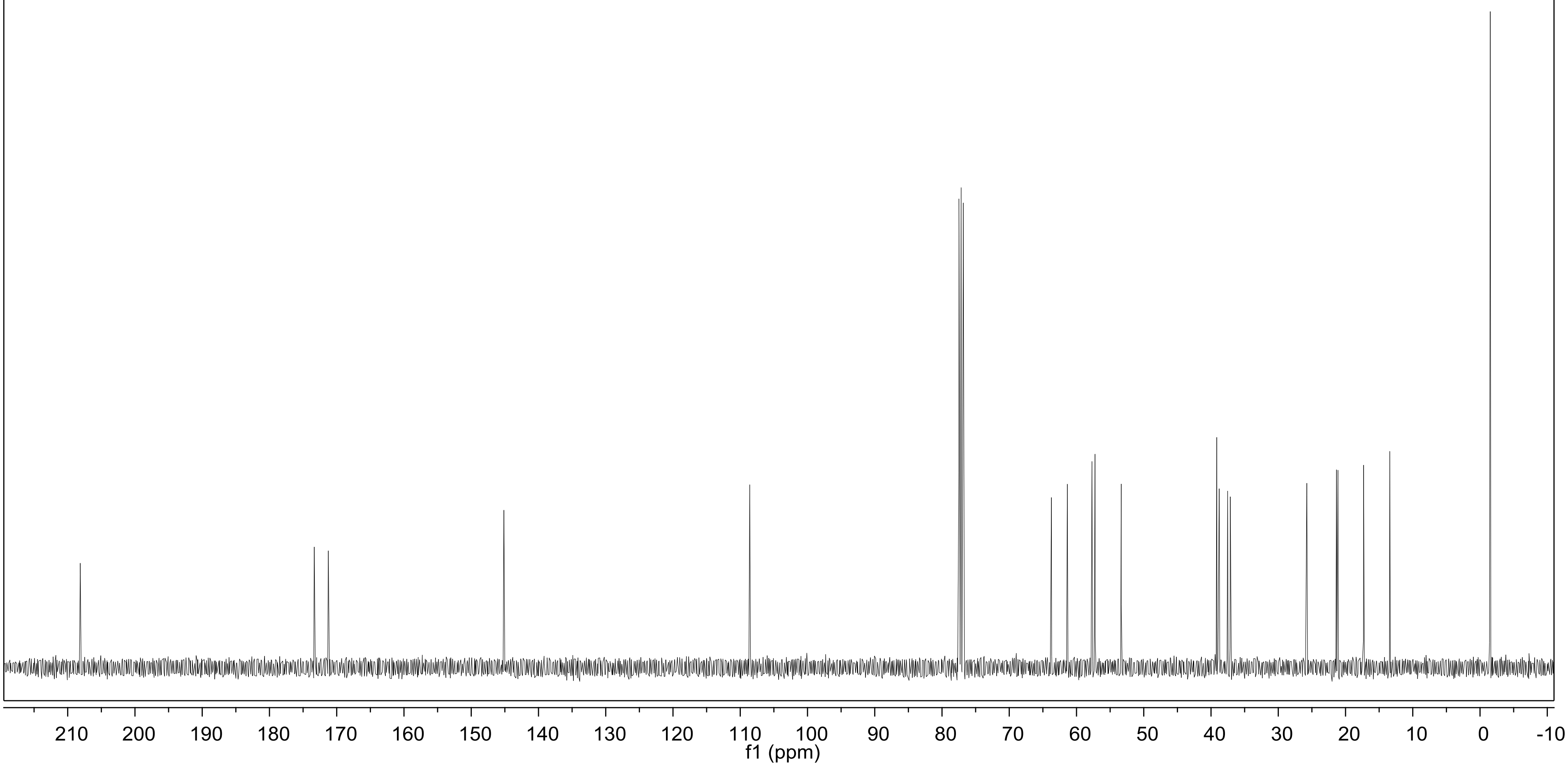

**Supplementary Figure 23. $^{1}$H NMR Spectrum of 28 (400 MHz, $CDCl_3$)**

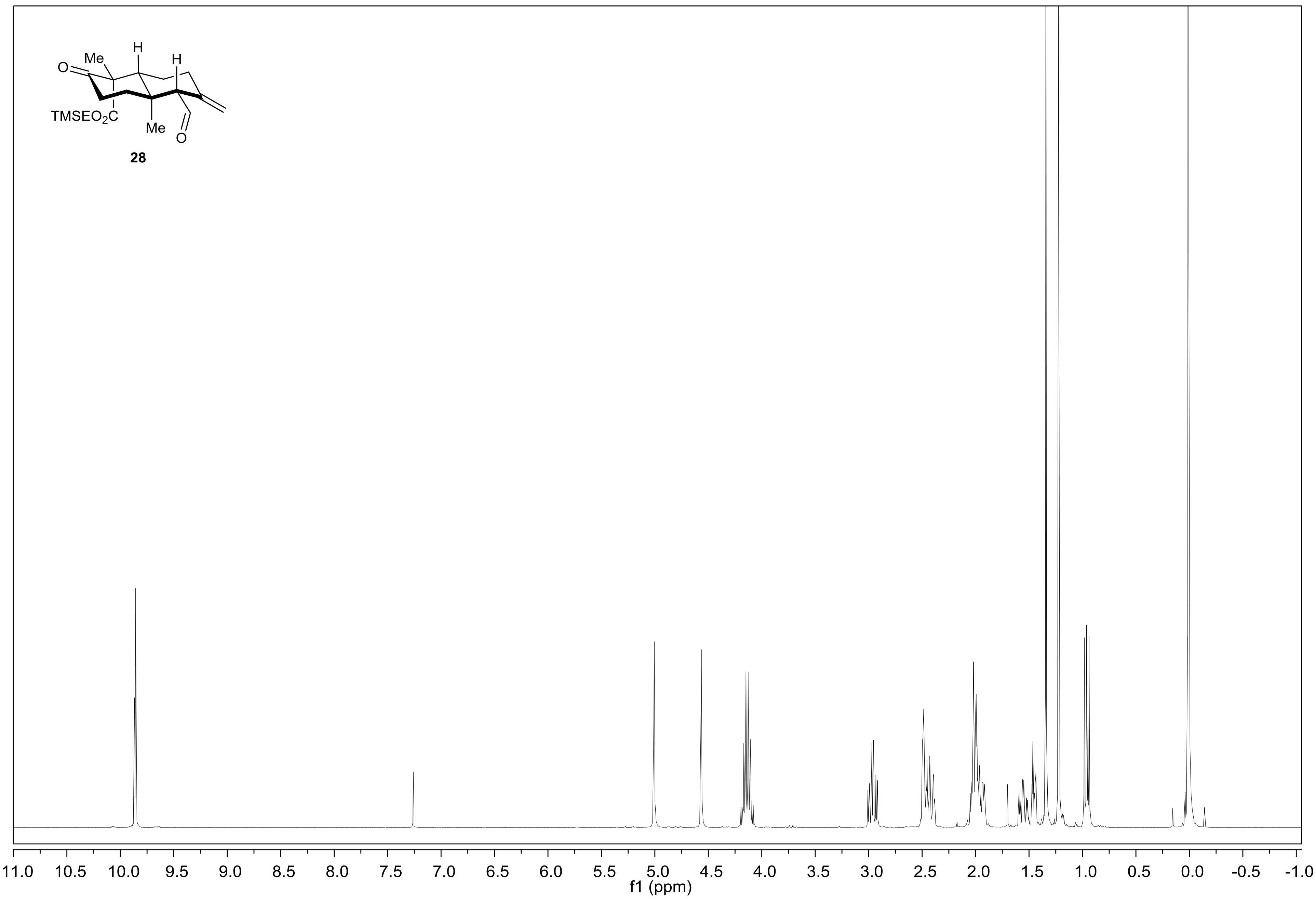

**Supplementary Figure 24. $^{13}$C NMR Spectrum of 28 (101 MHz, CDCl$_3$)**

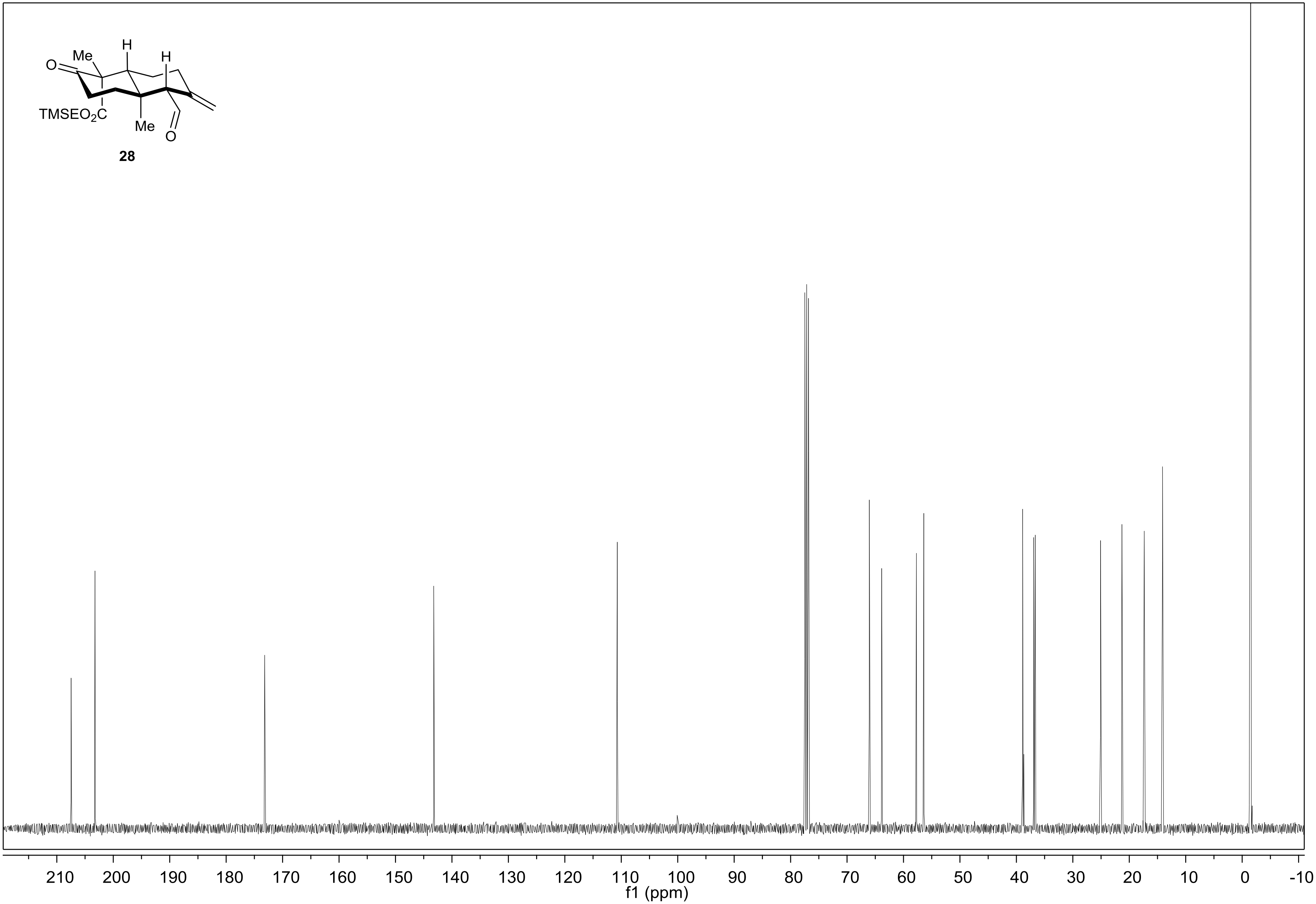

**Supplementary Figure 25. $^{1}H$ NMR Spectrum of 27 (400 MHz, $CDCl_3$)**

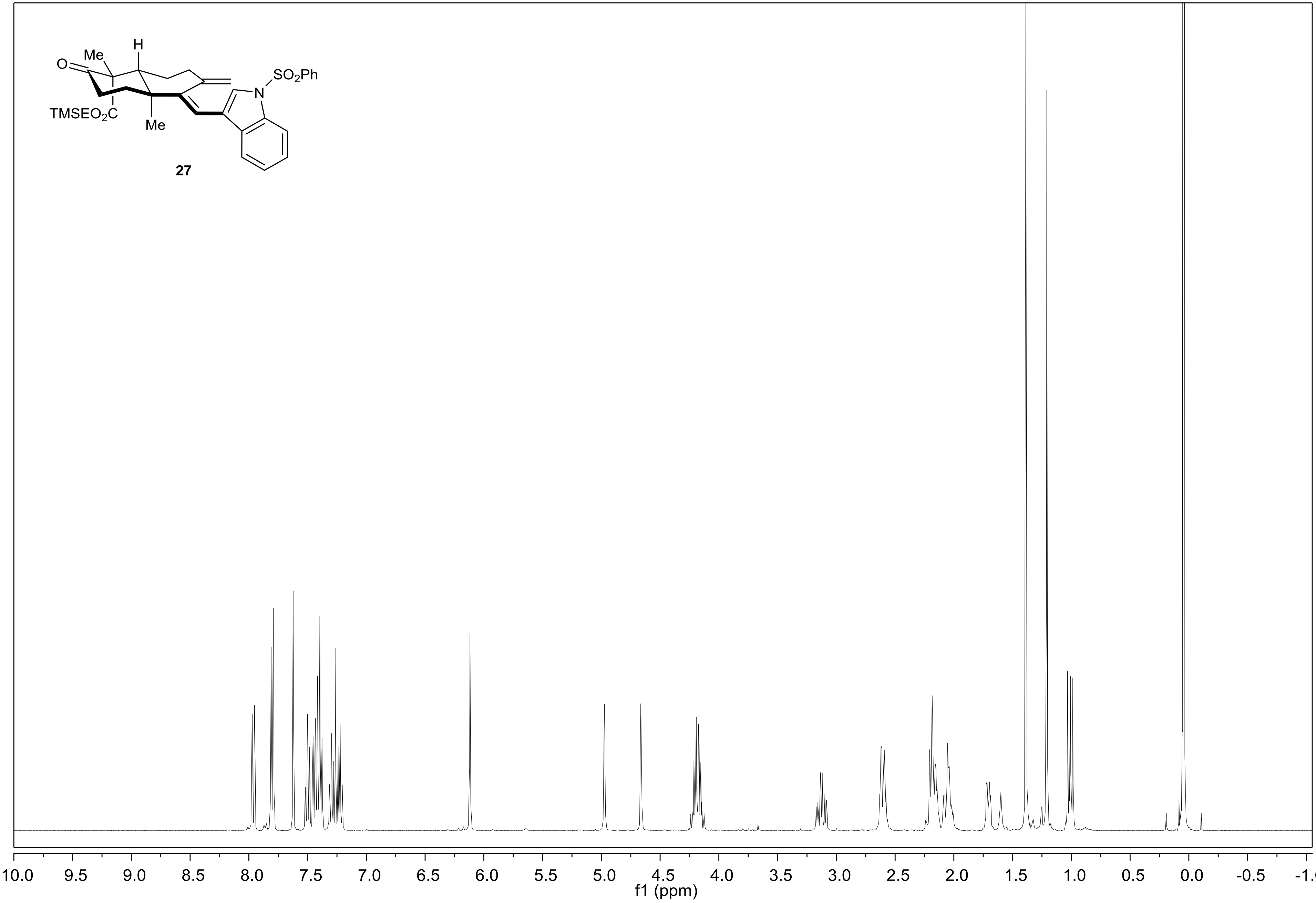

**Supplementary Figure 26. $^{13}$C NMR Spectrum of 27 (126 MHz, $CDCl_3$)**

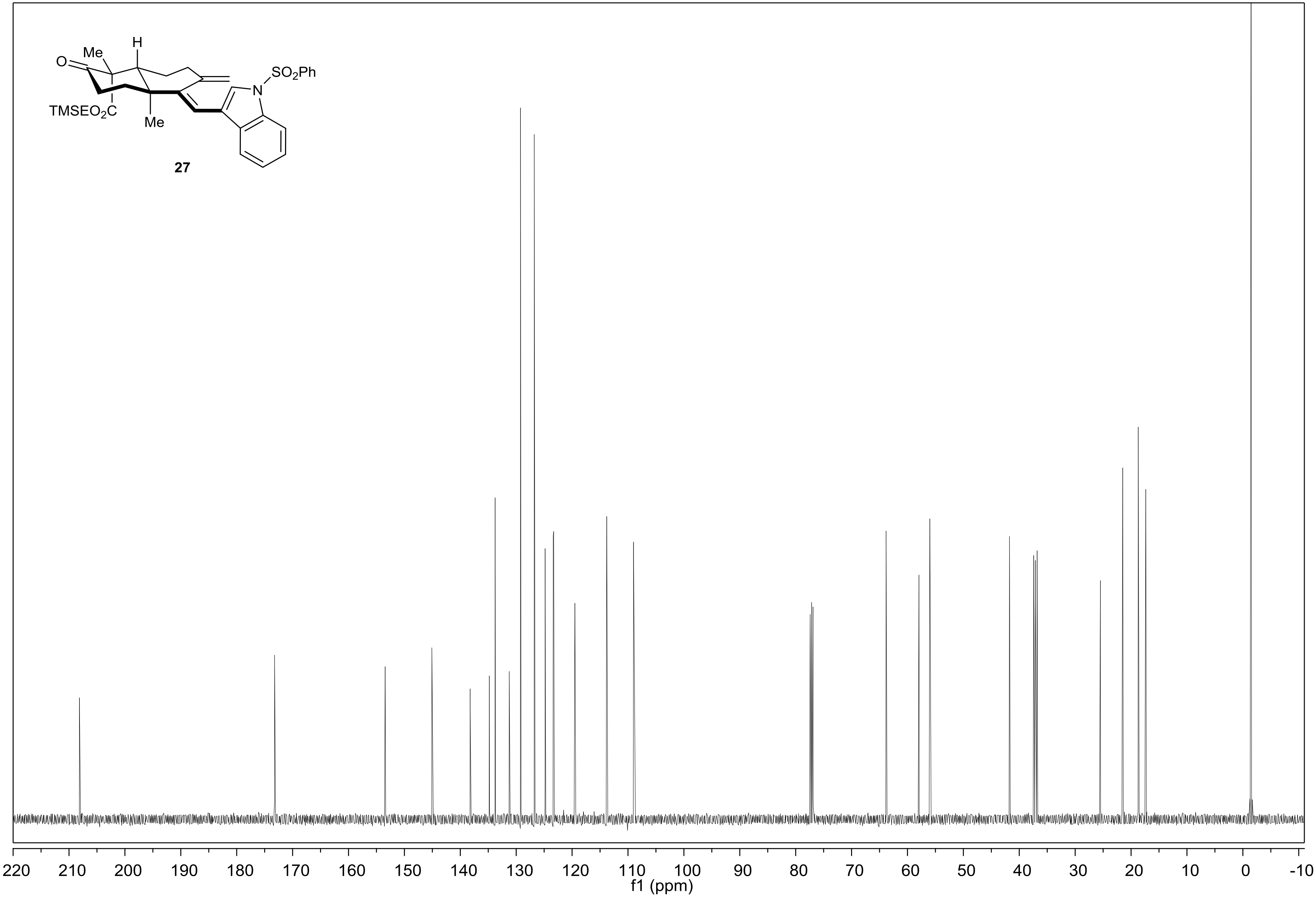

**Supplementary Figure 27. $^{1}$H NMR Spectrum of 29 (500 MHz, $CDCl_3$)**

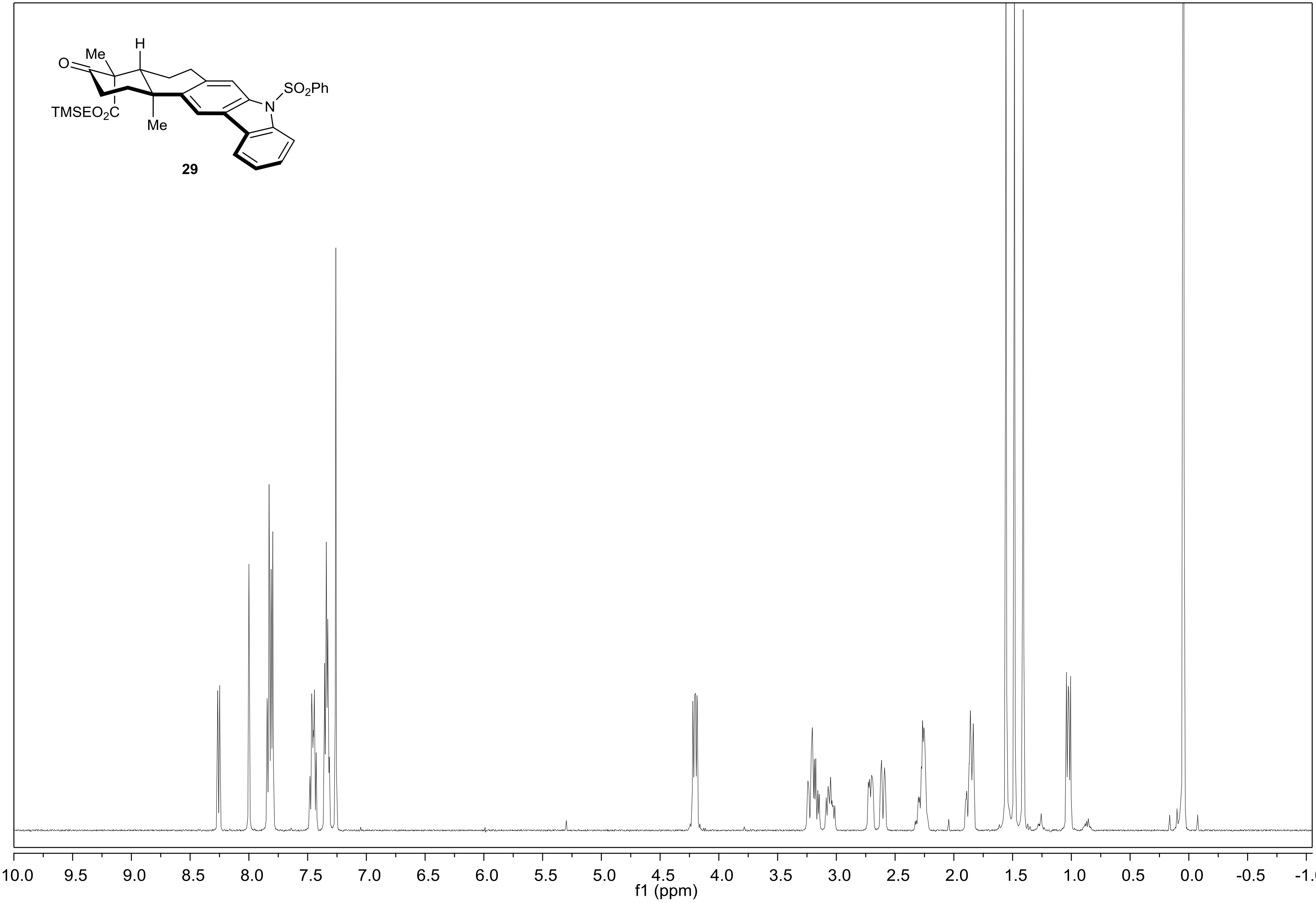

**Supplementary Figure 28. $^{13}$C NMR Spectrum of 29 (101 MHz, $CDCl_3$)**

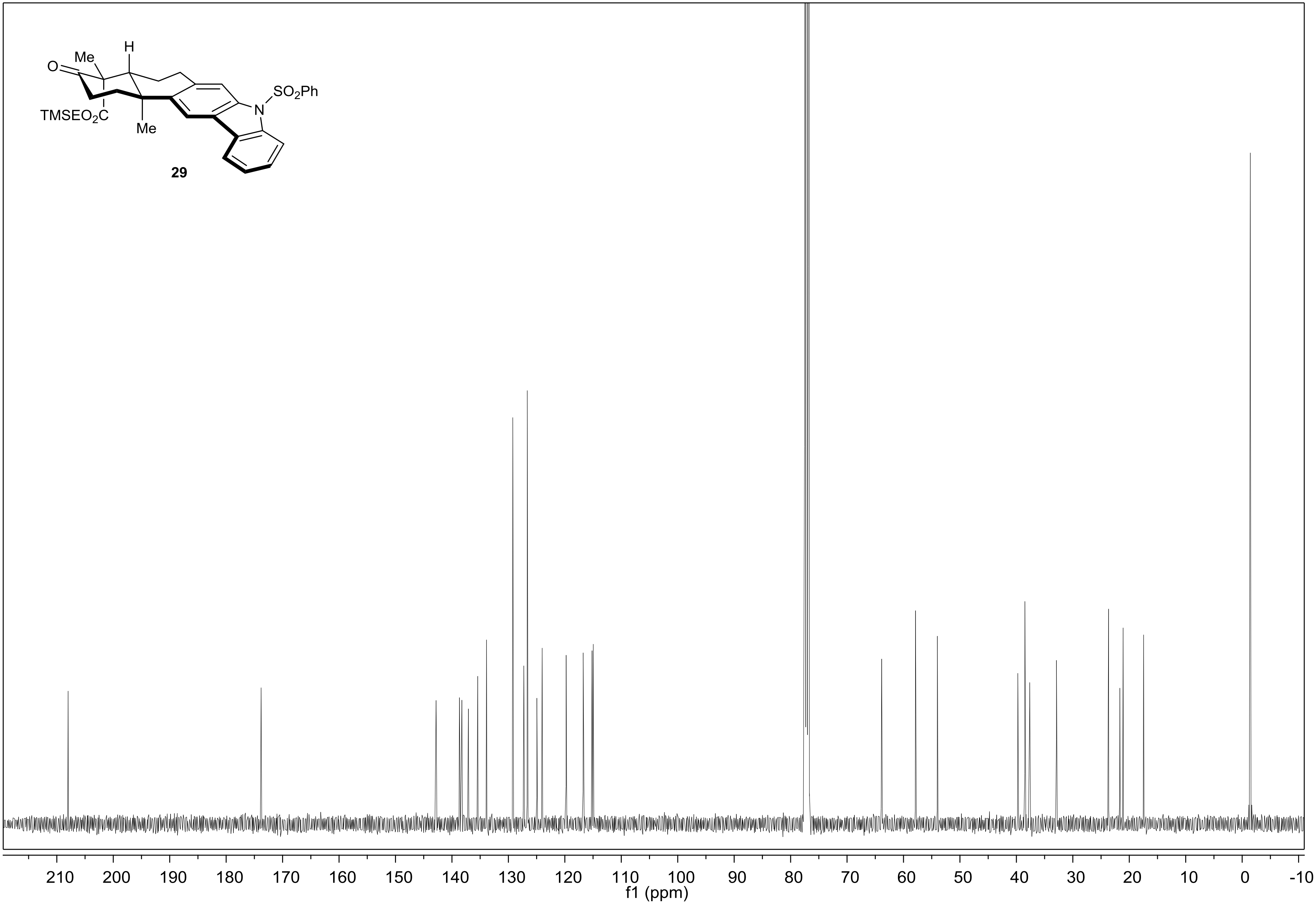

**Supplementary Figure 29. $^{1}$H NMR Spectrum of 30 (500 MHz, $CDCl_3$)**

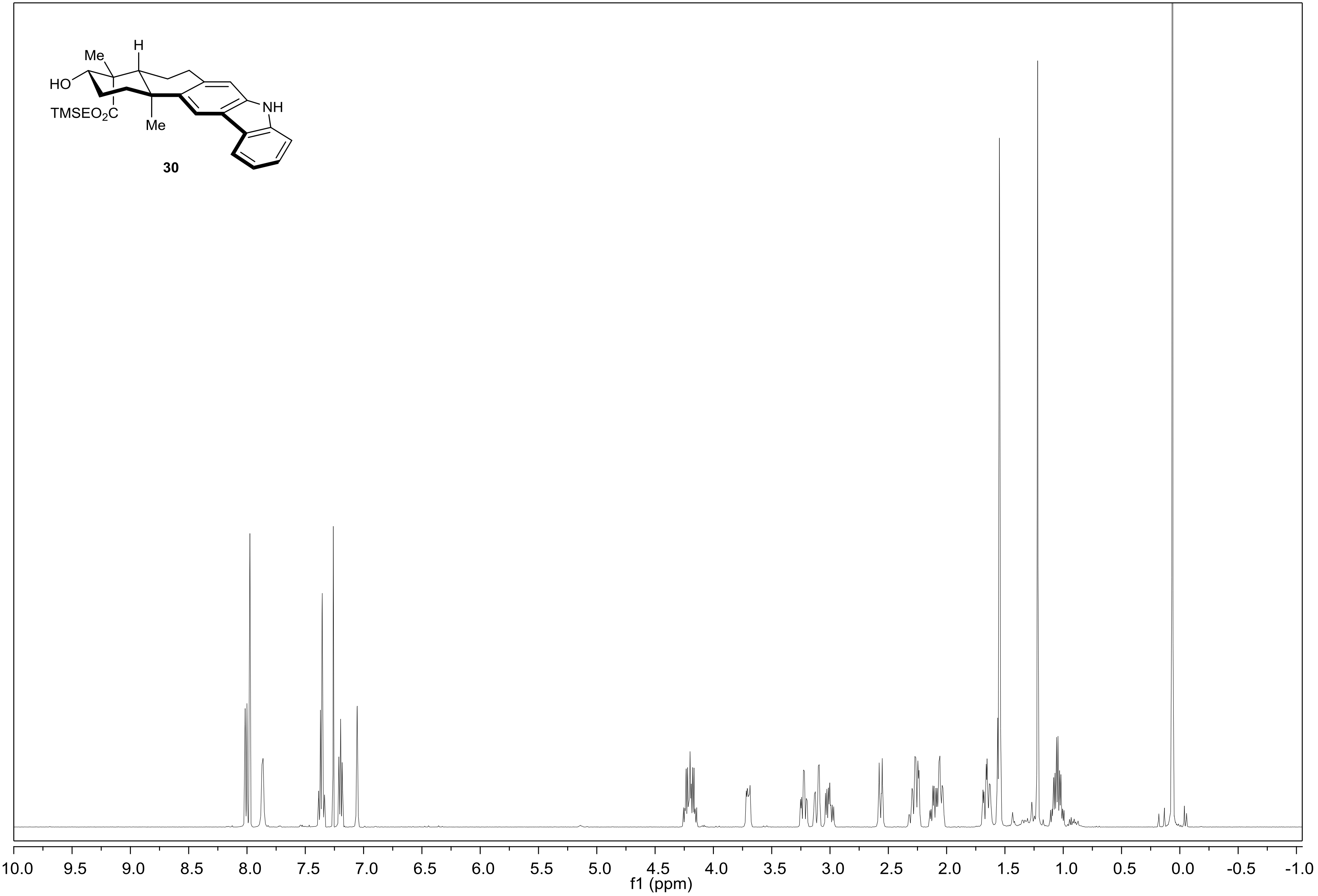

**Supplementary Figure 30.** $^{13}$**C NMR Spectrum of 30 (126 MHz, CDCl$_3$)**

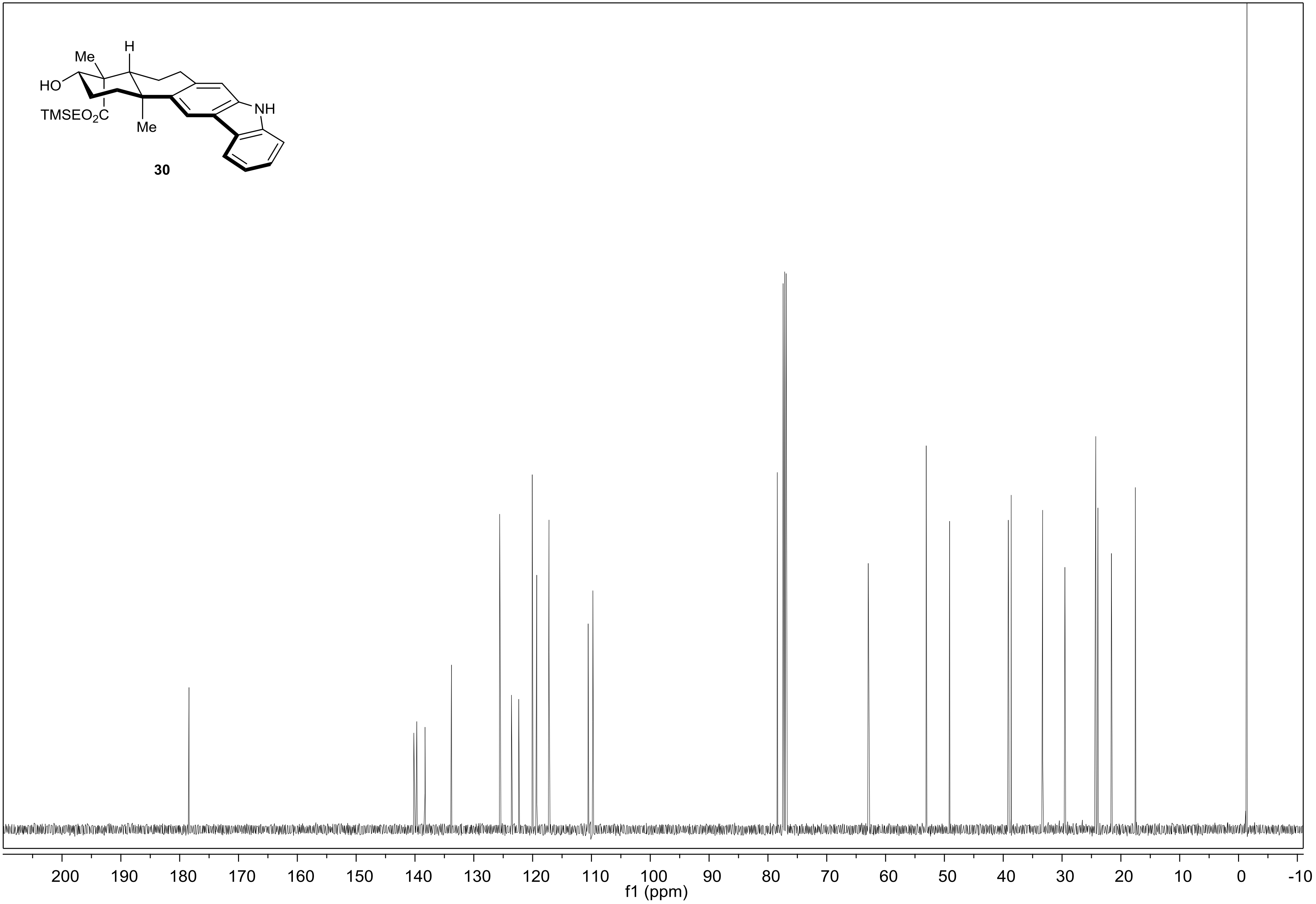

**Supplementary Figure 31. $^1$H NMR Spectrum of 2 (400 MHz, methanol-$d_4$)**

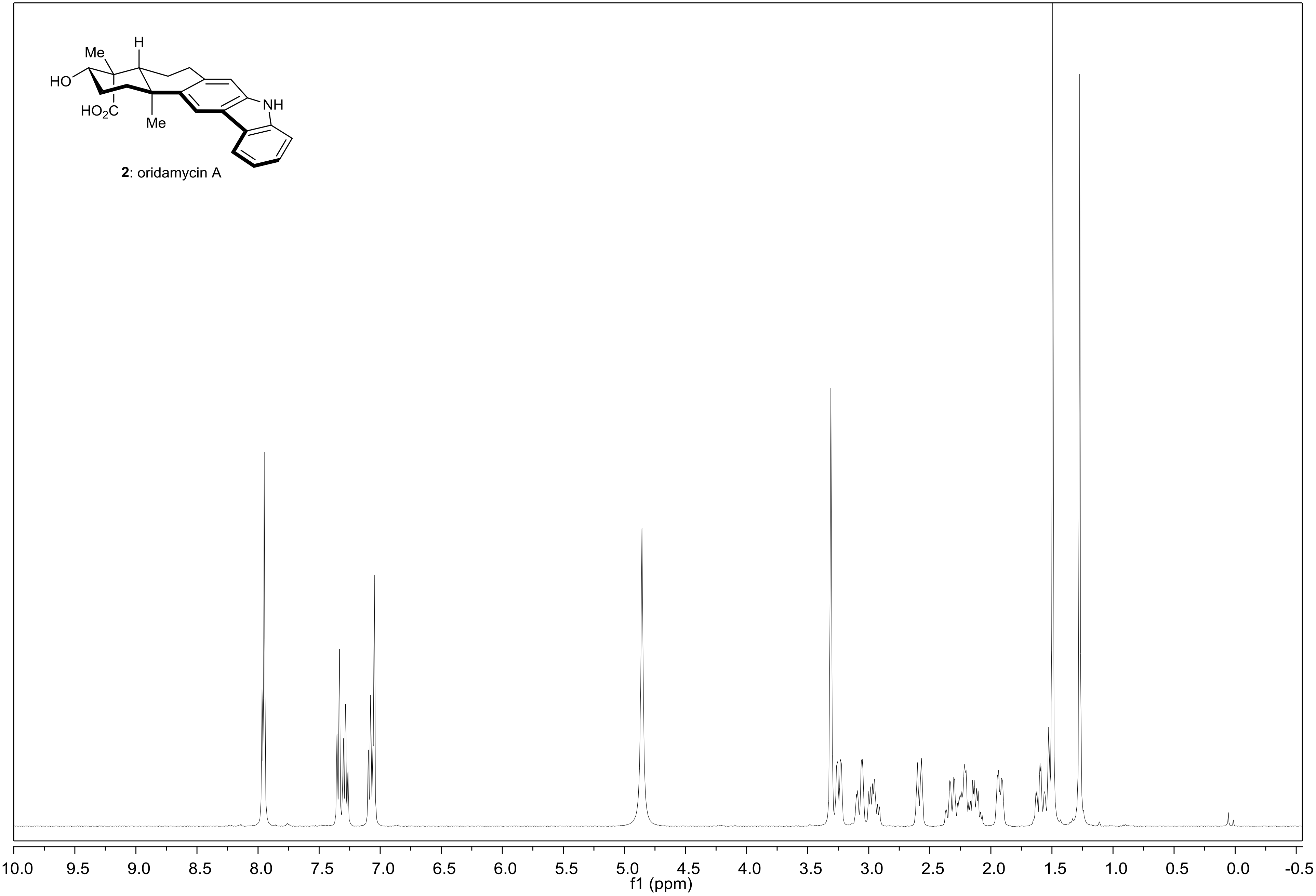

**Supplementary Figure 32. $^{13}$C NMR Spectrum of 2 (101 MHz, methanol-$d_4$)**

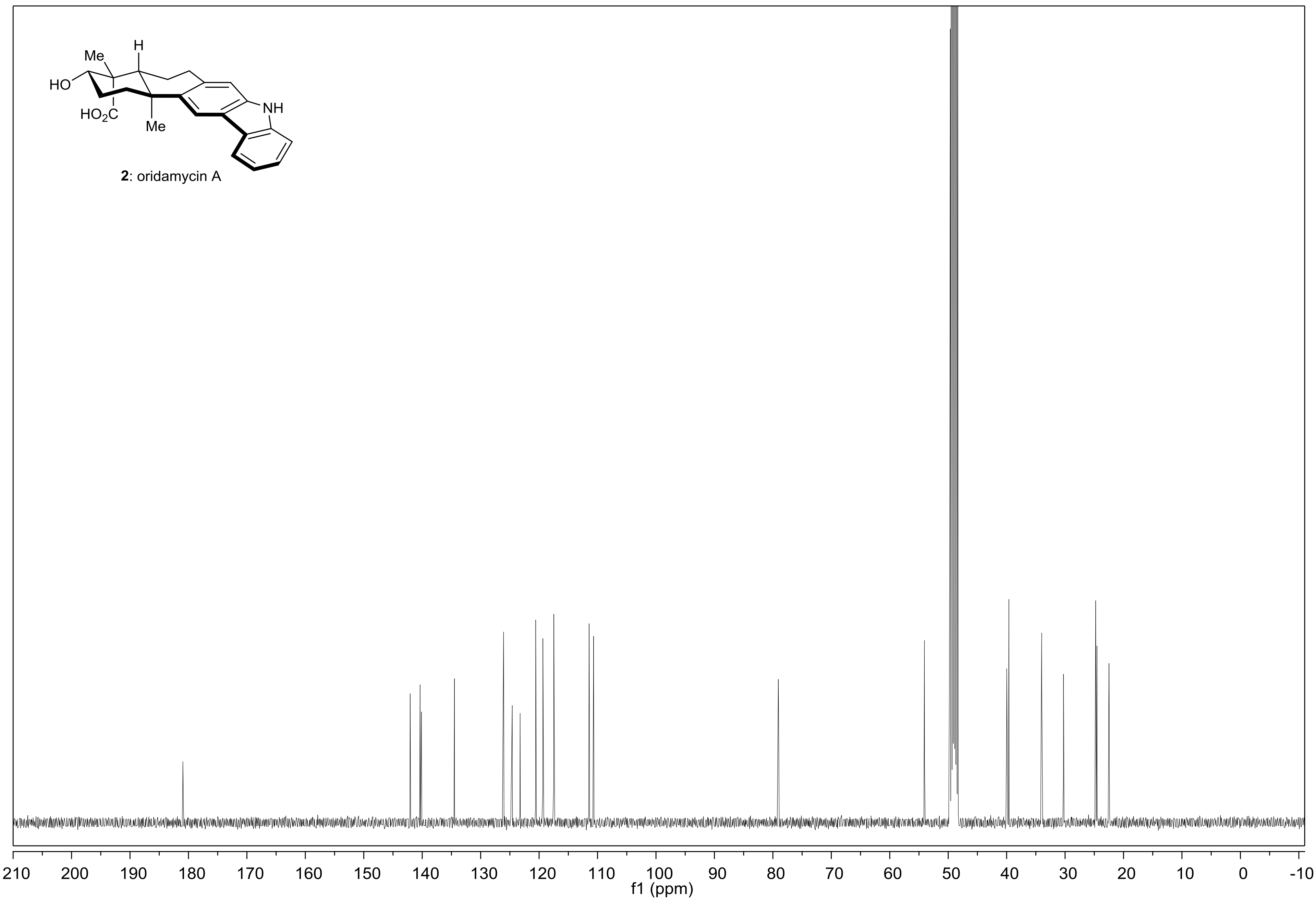

**Supplementary Figure 33. $^1$H NMR Spectrum of 31a (400 MHz, $CDCl_3$)**

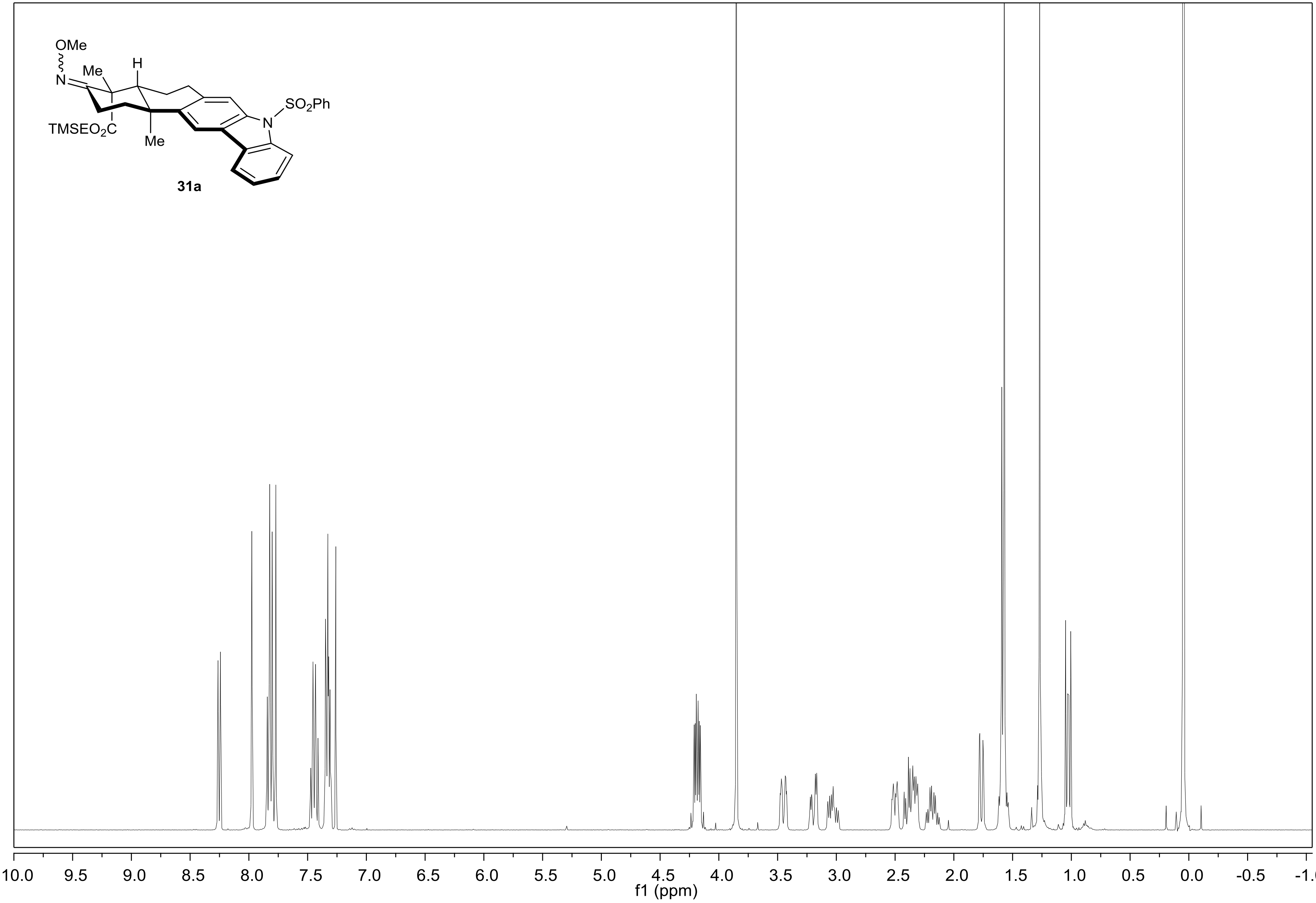

**Supplementary Figure 34. $^{13}C$ NMR Spectrum of 31a (126 MHz, $CDCl_3$)**

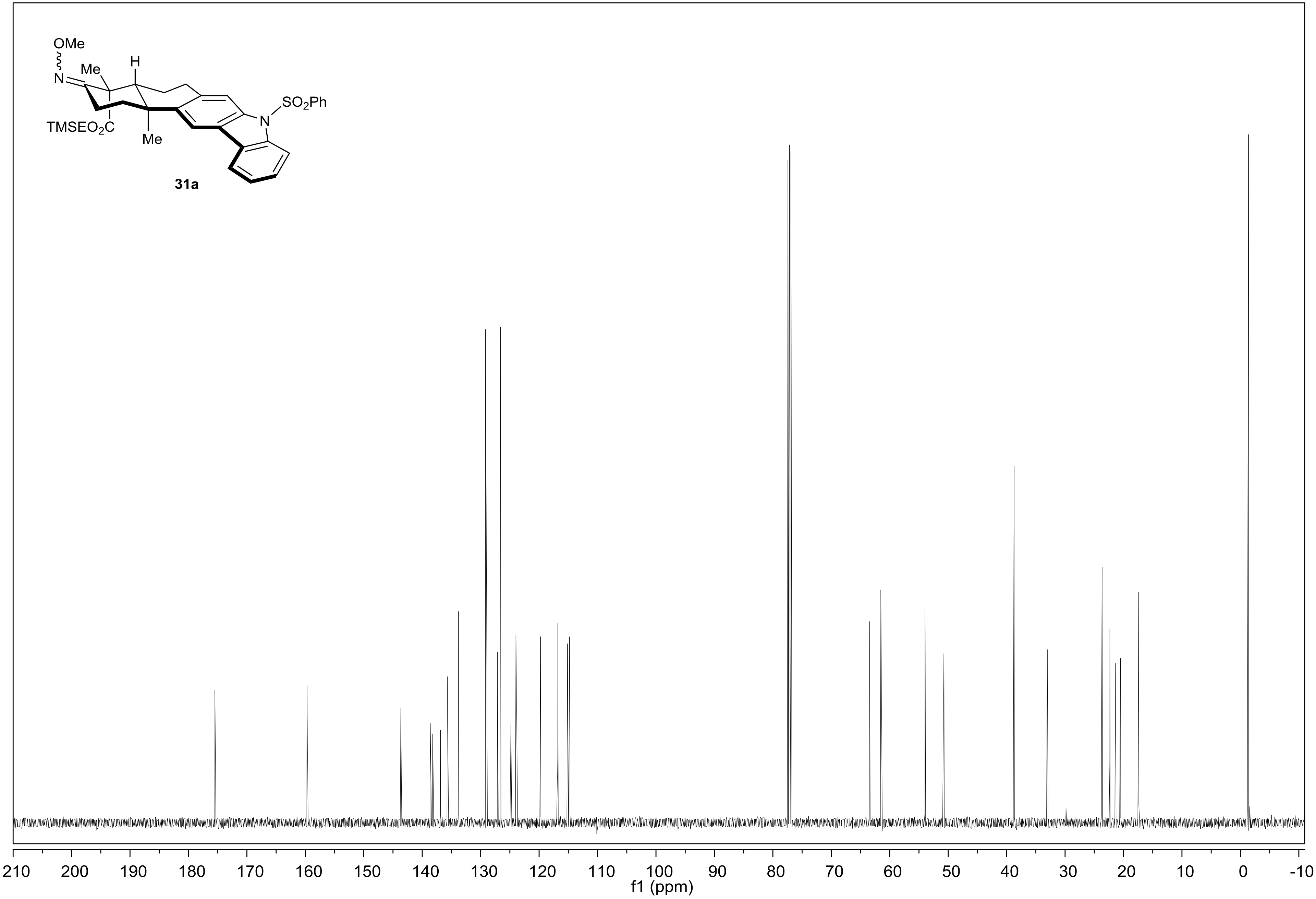

**Supplementary Figure 35. $^{1}$H NMR Spectrum of 31 (400 MHz, $CDCl_3$)**

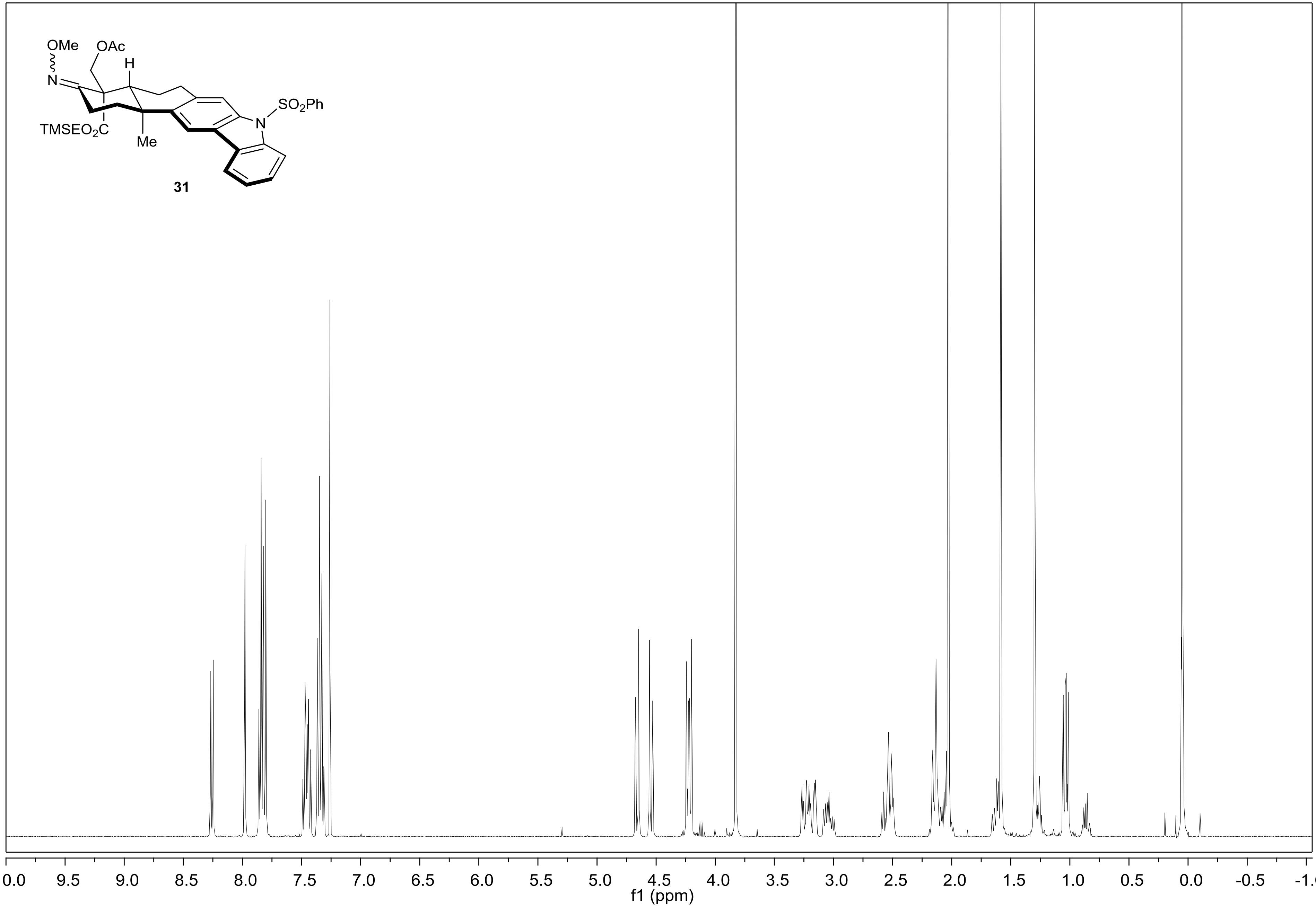

**Supplementary Figure 36. $^{13}C$ NMR Spectrum of 31 (126 MHz, $CDCl_3$)**

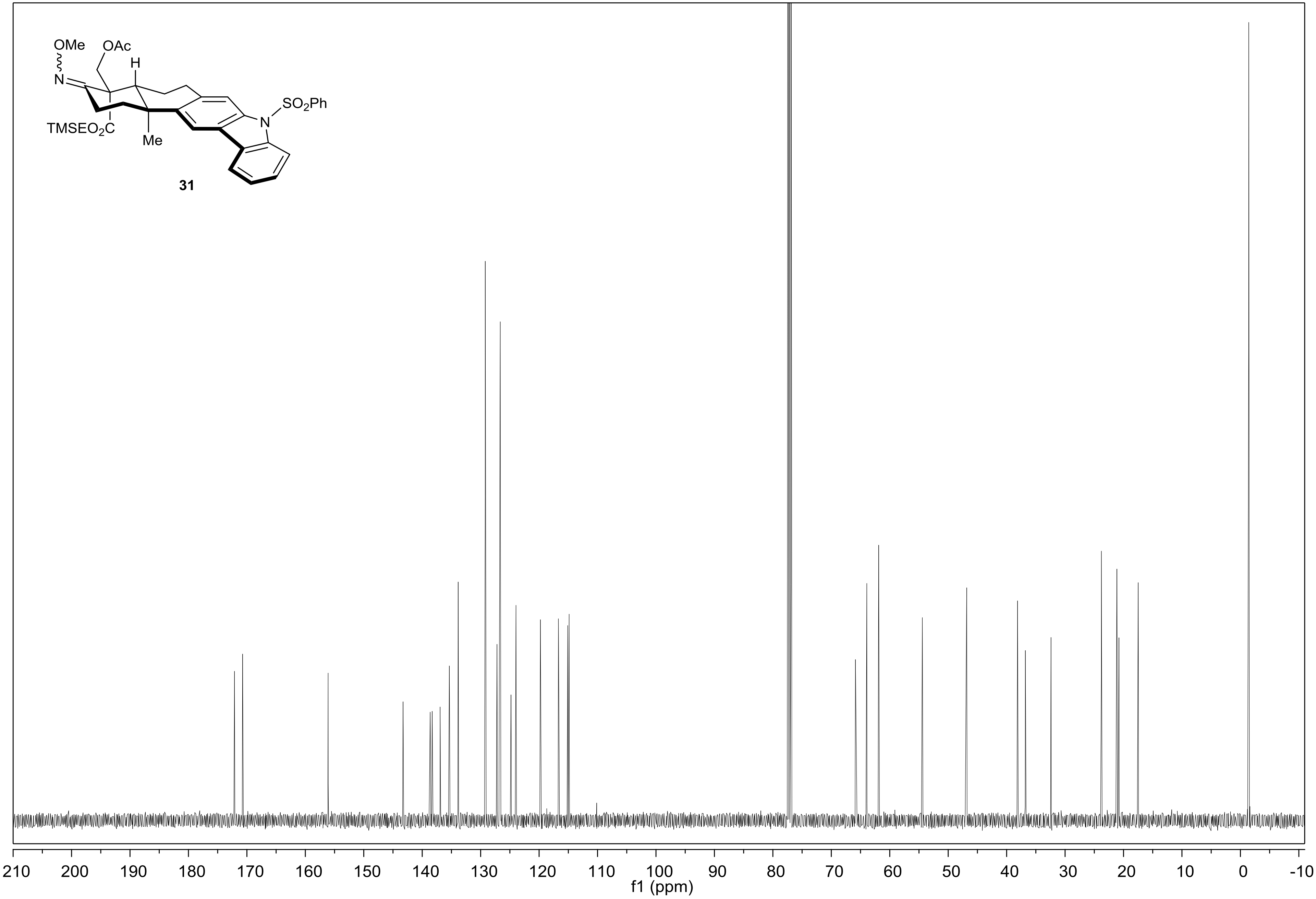

**Supplementary Figure 37. $^{1}H$ NMR Spectrum of 32a (400 MHz, $CDCl_3$)**

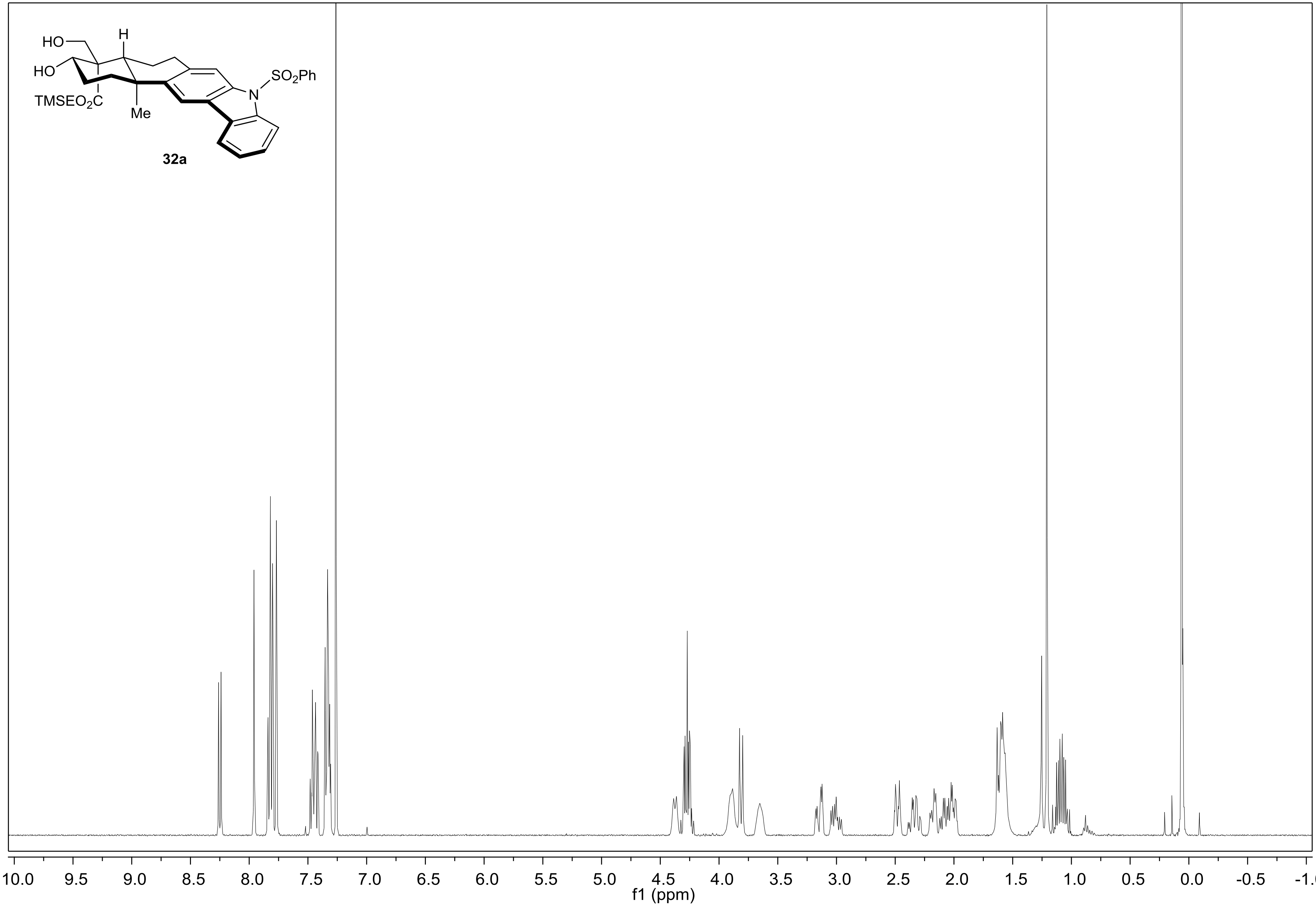

**Supplementary Figure 38. $^{13}$C NMR Spectrum of 32a (126 MHz, $CDCl_3$)**

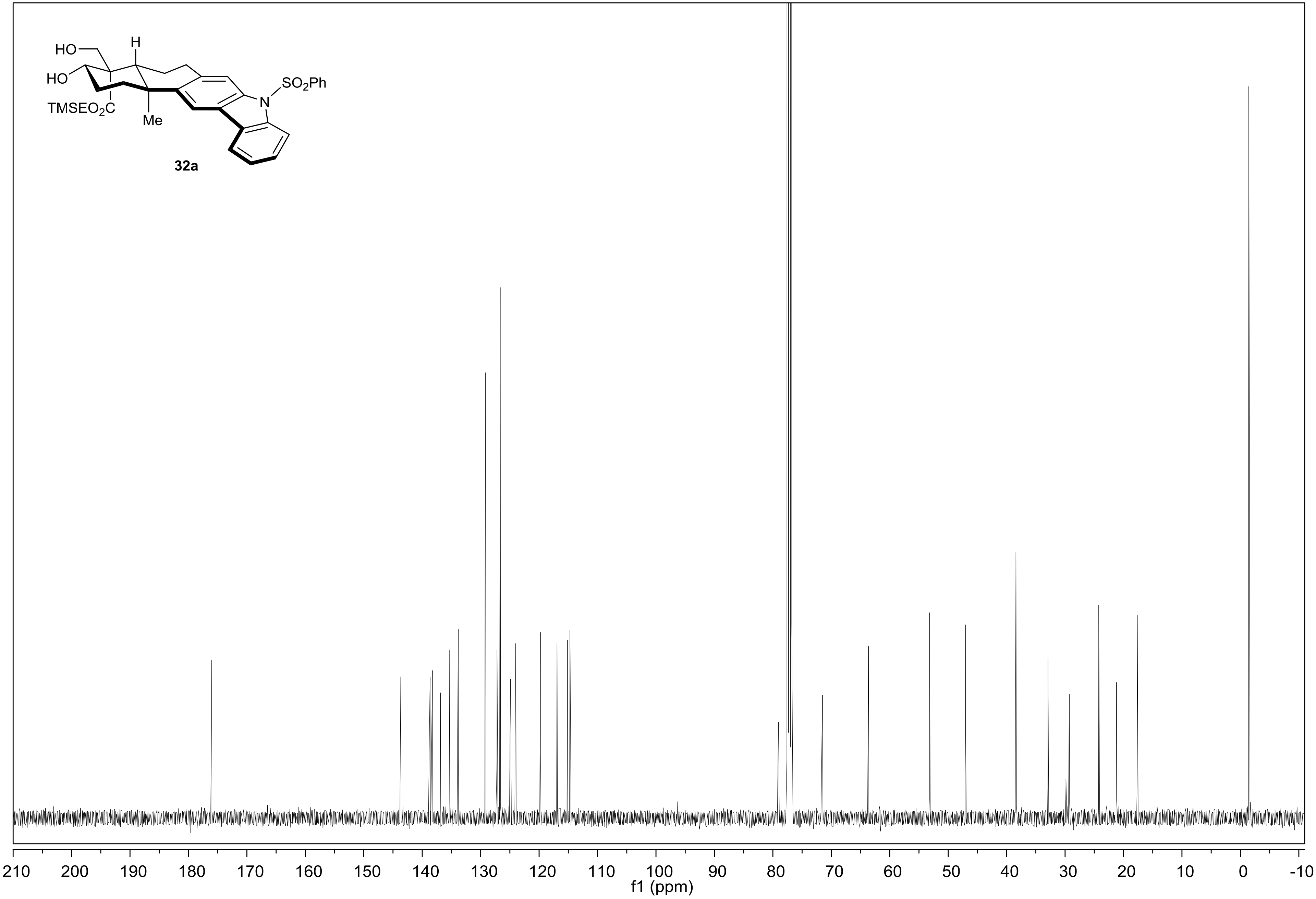

**Supplementary Figure 39. $^1$H NMR Spectrum of 32 (400 MHz, $CDCl_3$)**

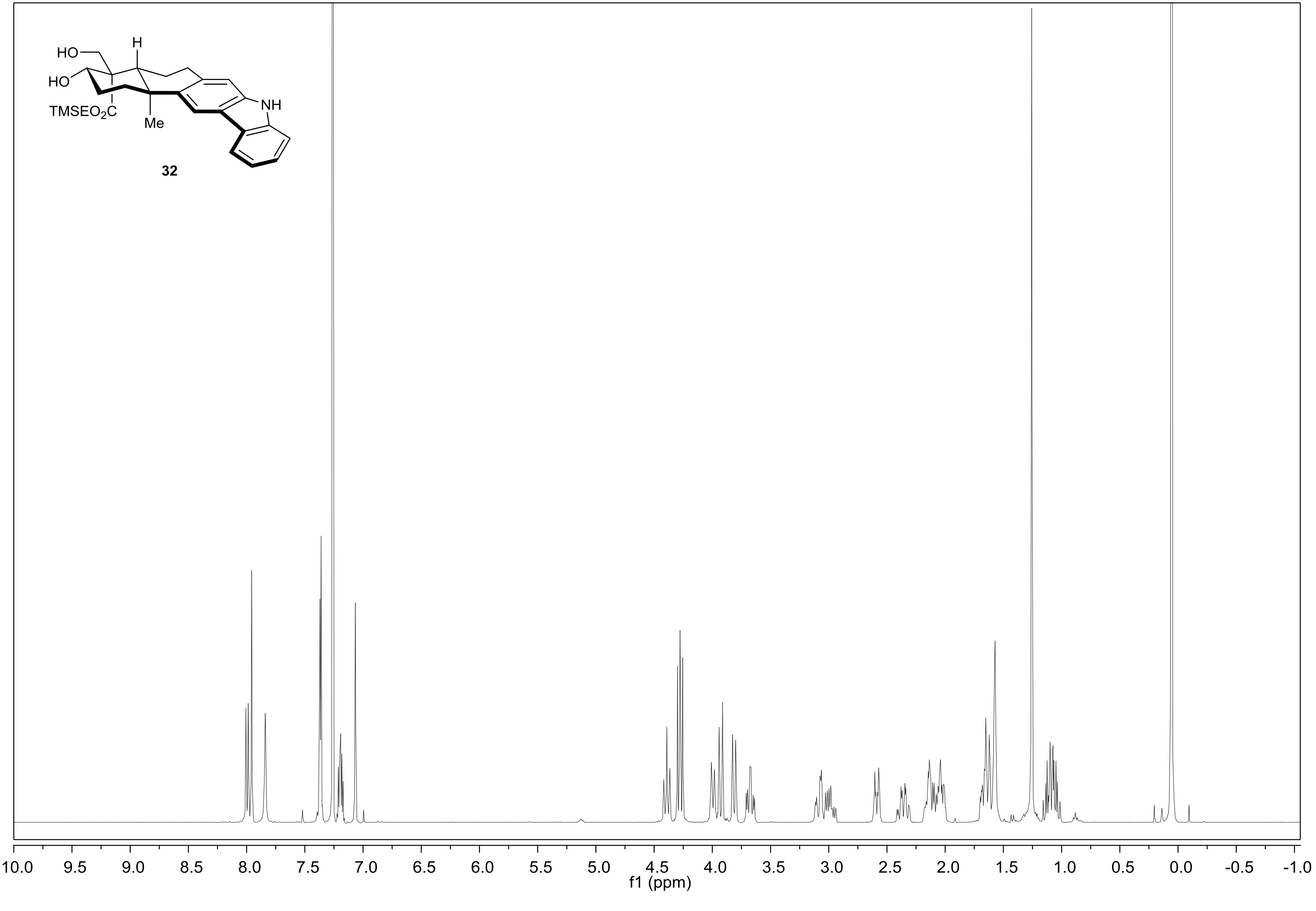

**Supplementary Figure 40. $^{13}$C NMR Spectrum of 32 (126 MHz, $CDCl_3$)**

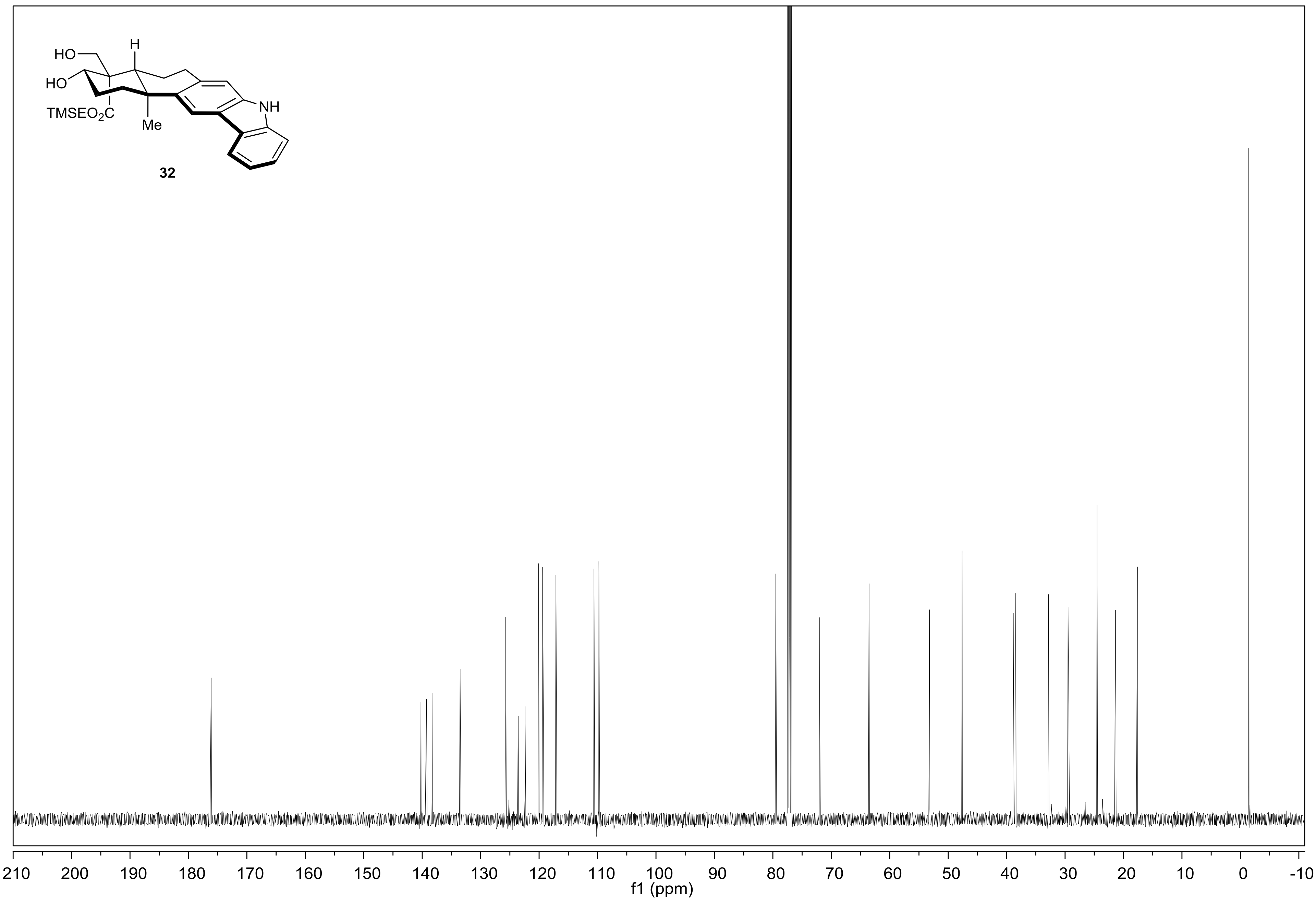

**Supplementary Figure 41. $^{1}$H NMR Spectrum of 3 (600 MHz, methanol-$d_4$)**

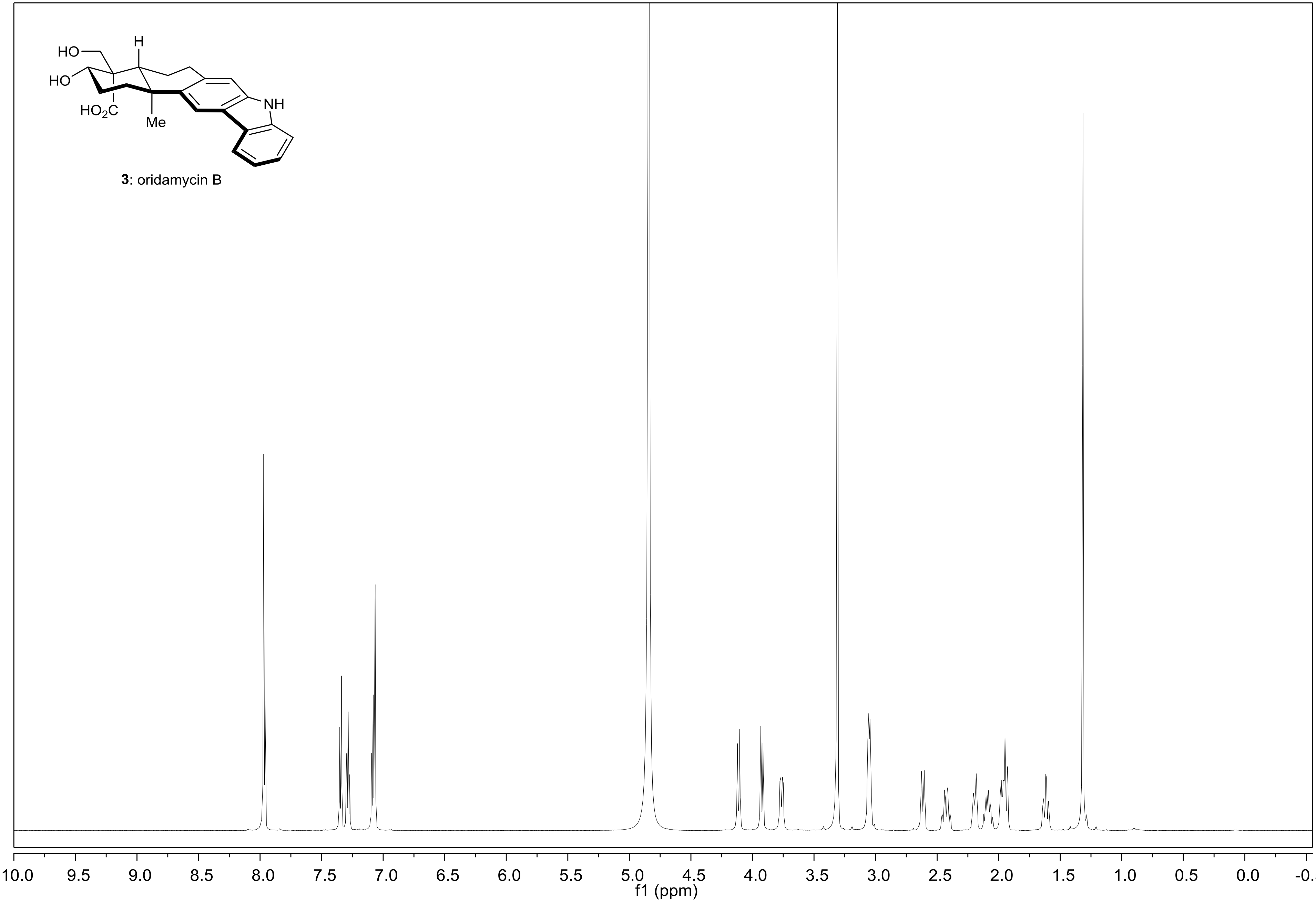

**Supplementary Figure 42. $^{13}C$ NMR Spectrum of 3 (151 MHz, methanol-$d_4$)**

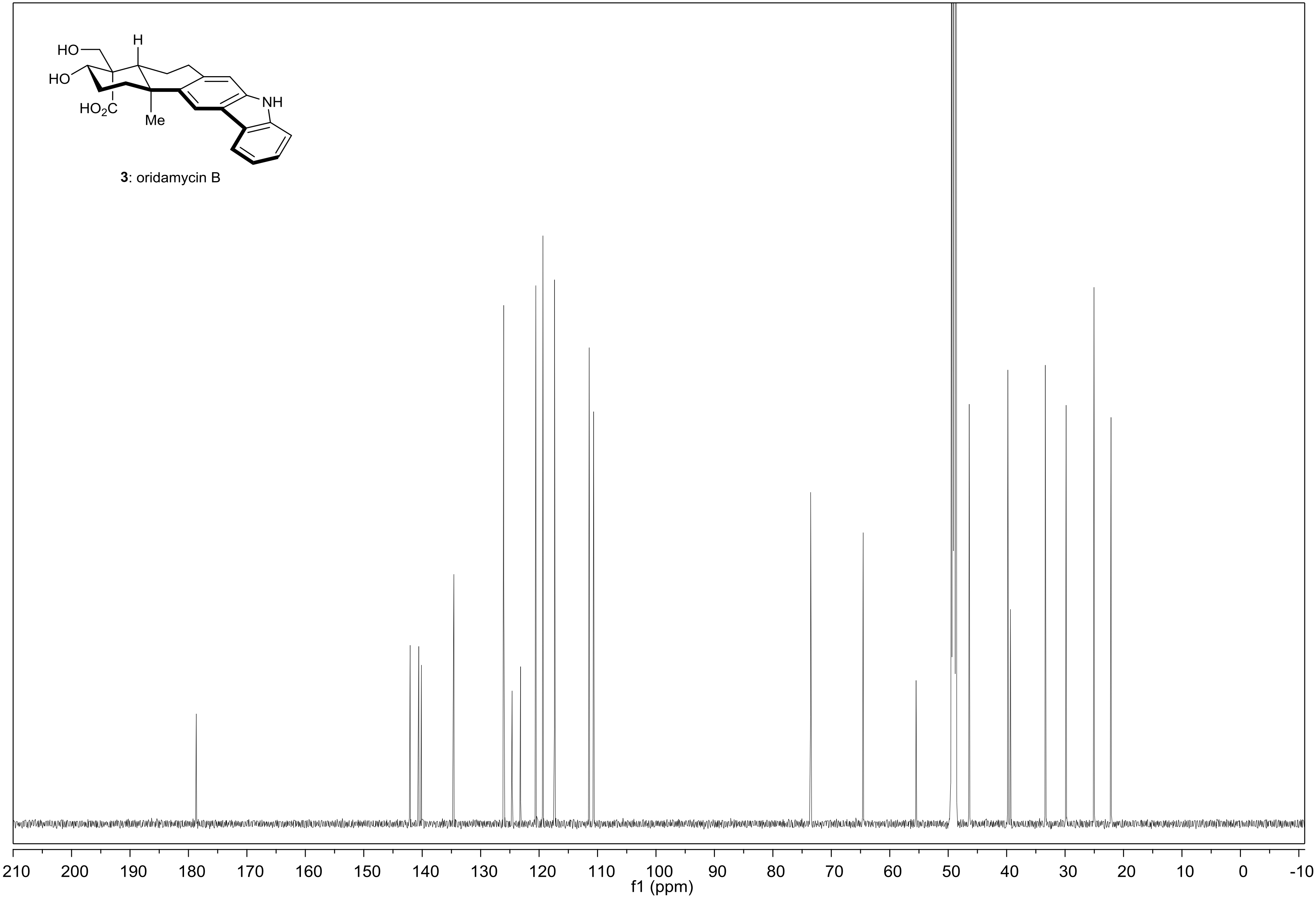

**Supplementary Figure 43. $^{1}$H NMR Spectrum of 34 (400 MHz, $CDCl_3$)**

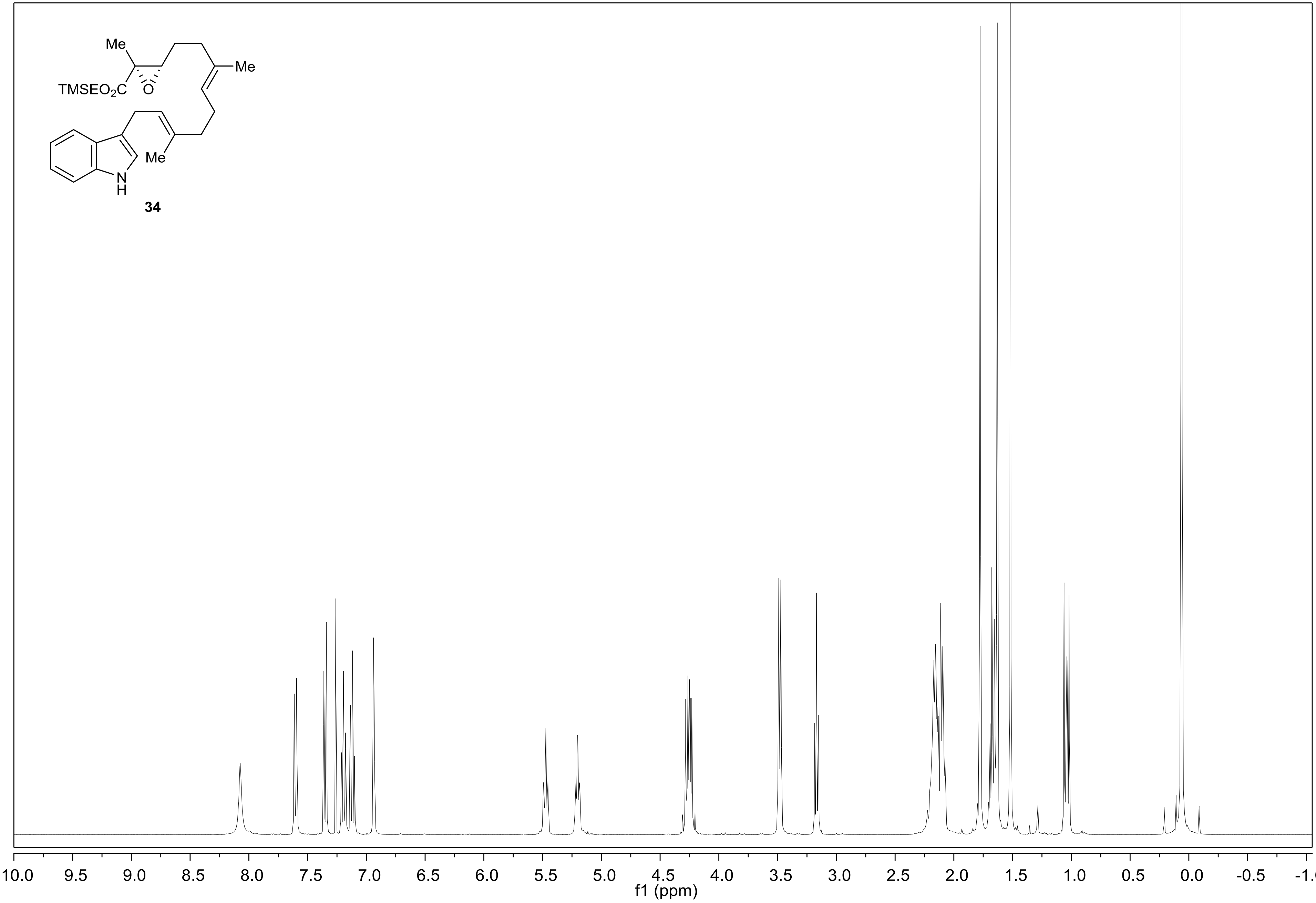

**Supplementary Figure 44. $^{13}$C NMR Spectrum of 34 (126 MHz, $CDCl_3$)**

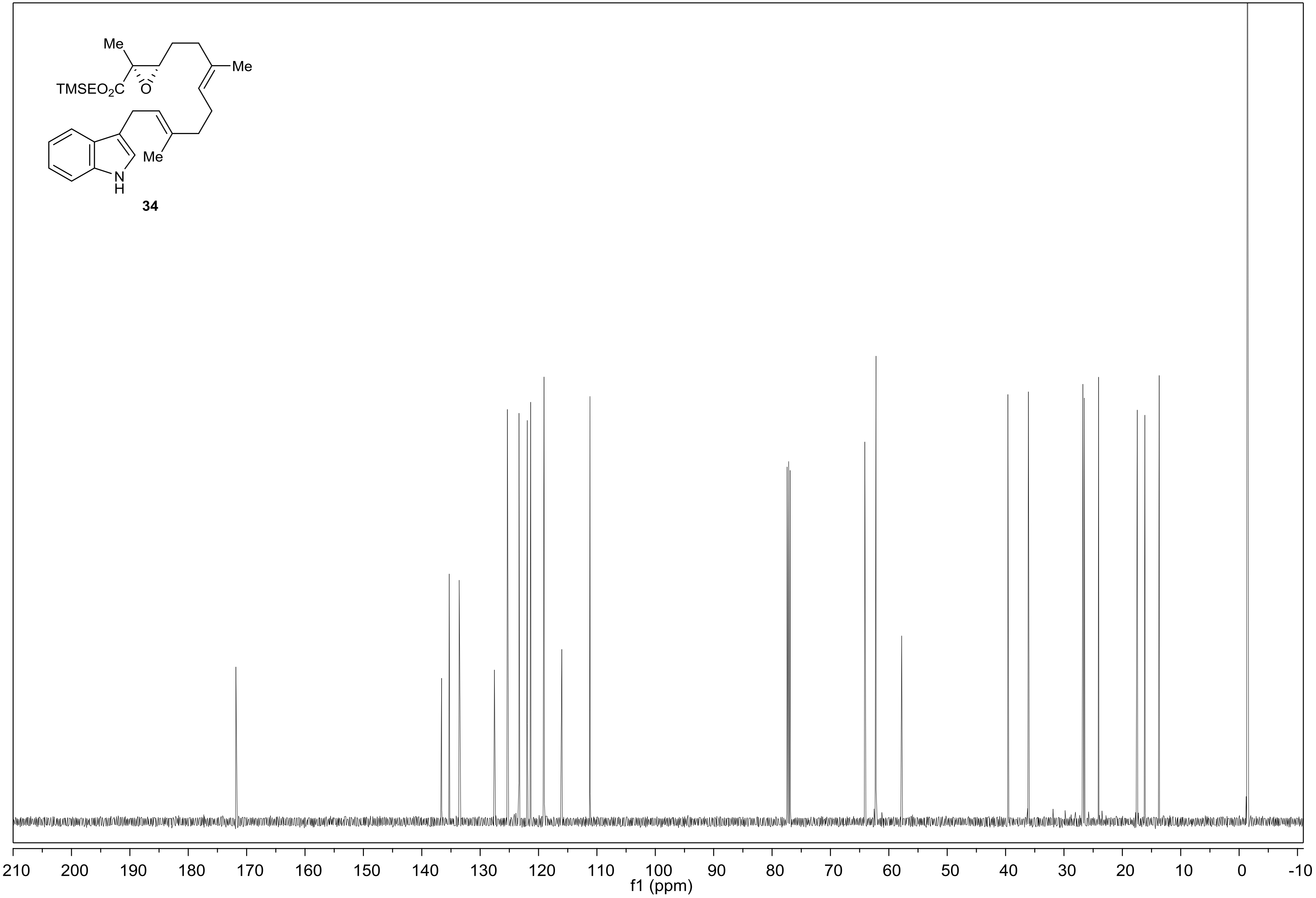

**Supplementary Figure 45. $^{1}$H NMR Spectrum of 35 (400 MHz, $CDCl_3$)**

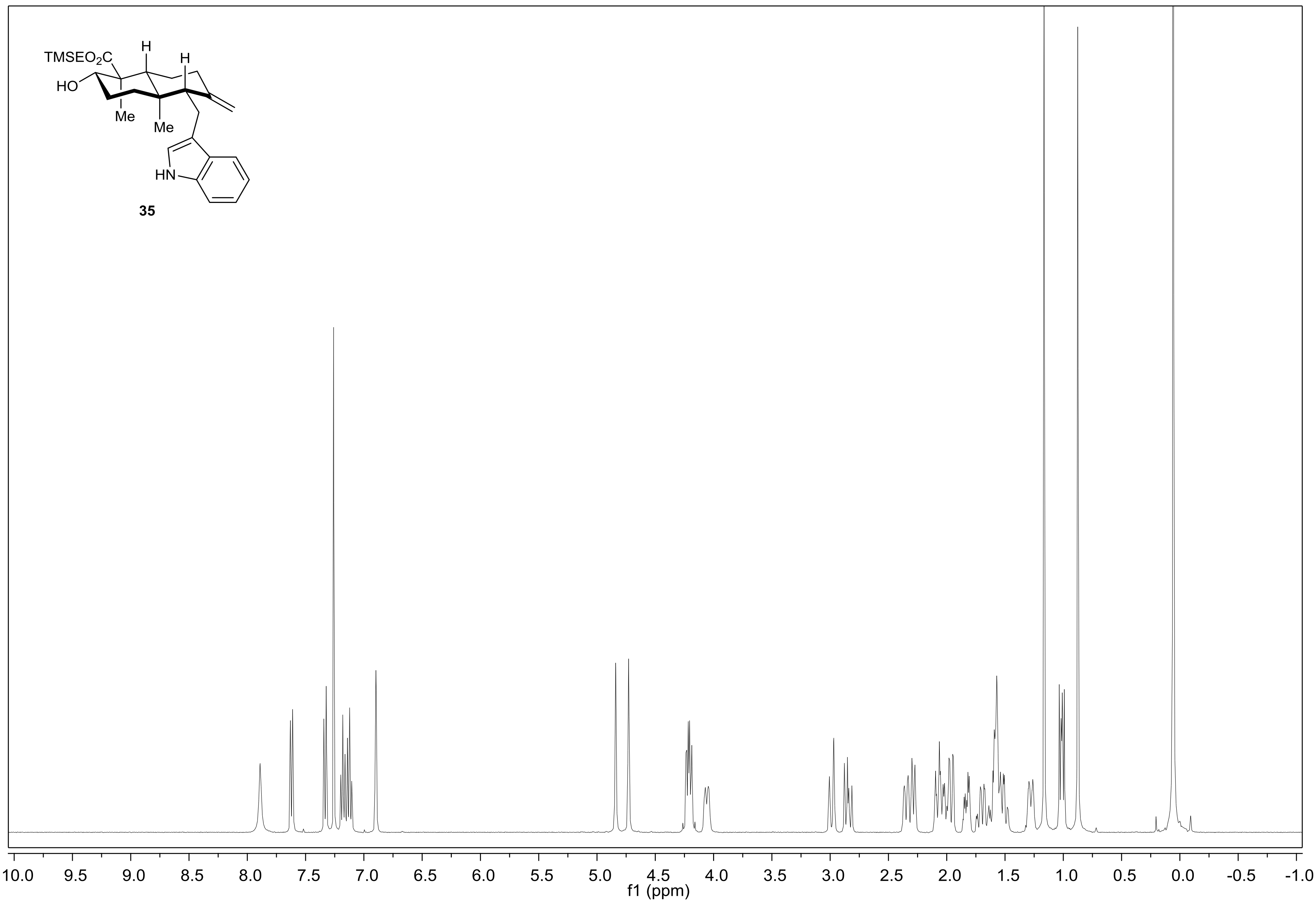

**Supplementary Figure 46. $^{13}C$ NMR Spectrum of 35 (126 MHz, $CDCl_3$)**

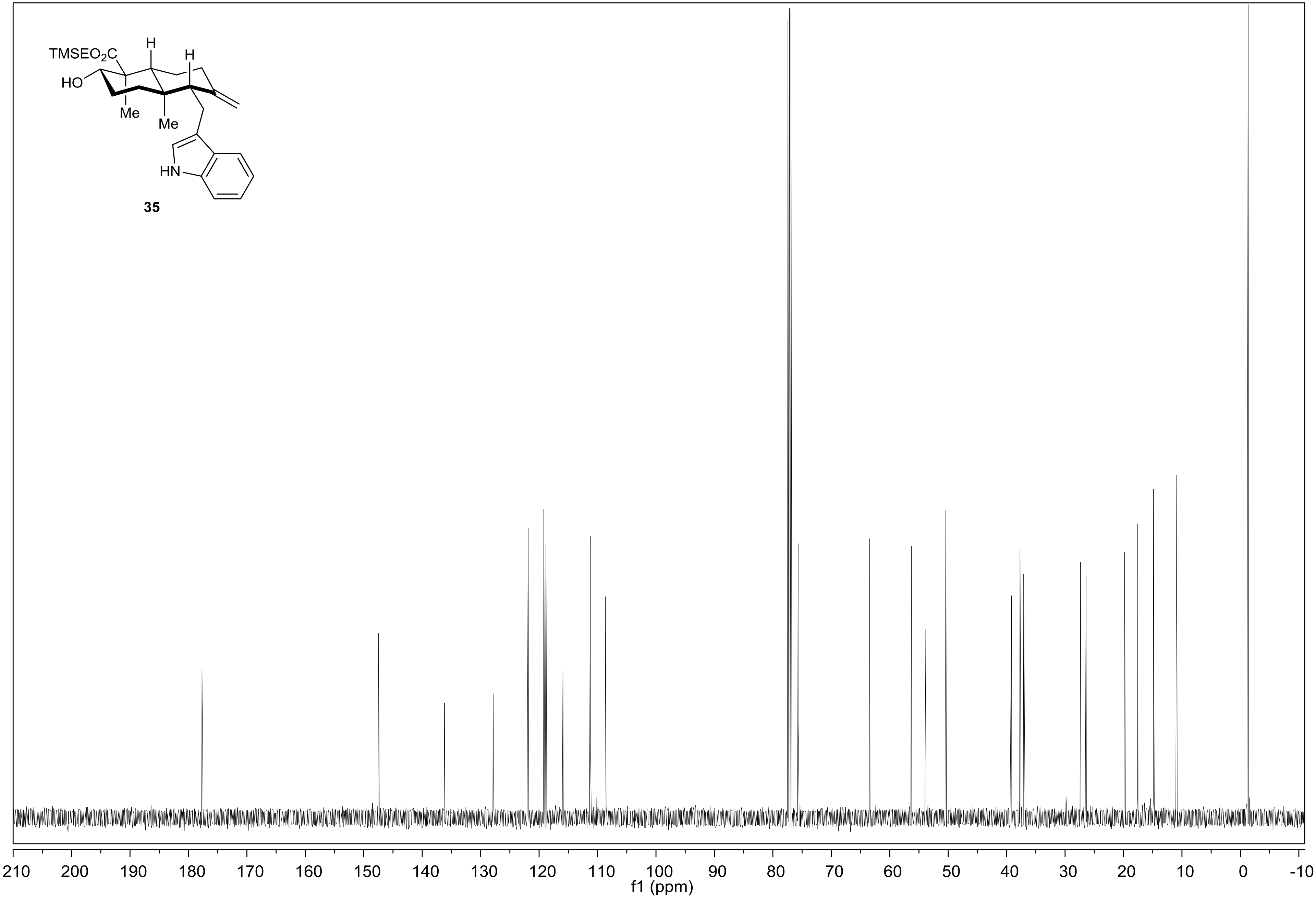

**Supplementary Figure 47. $^1$H NMR Spectrum of 6 (400 MHz, methanol-$d_4$)**

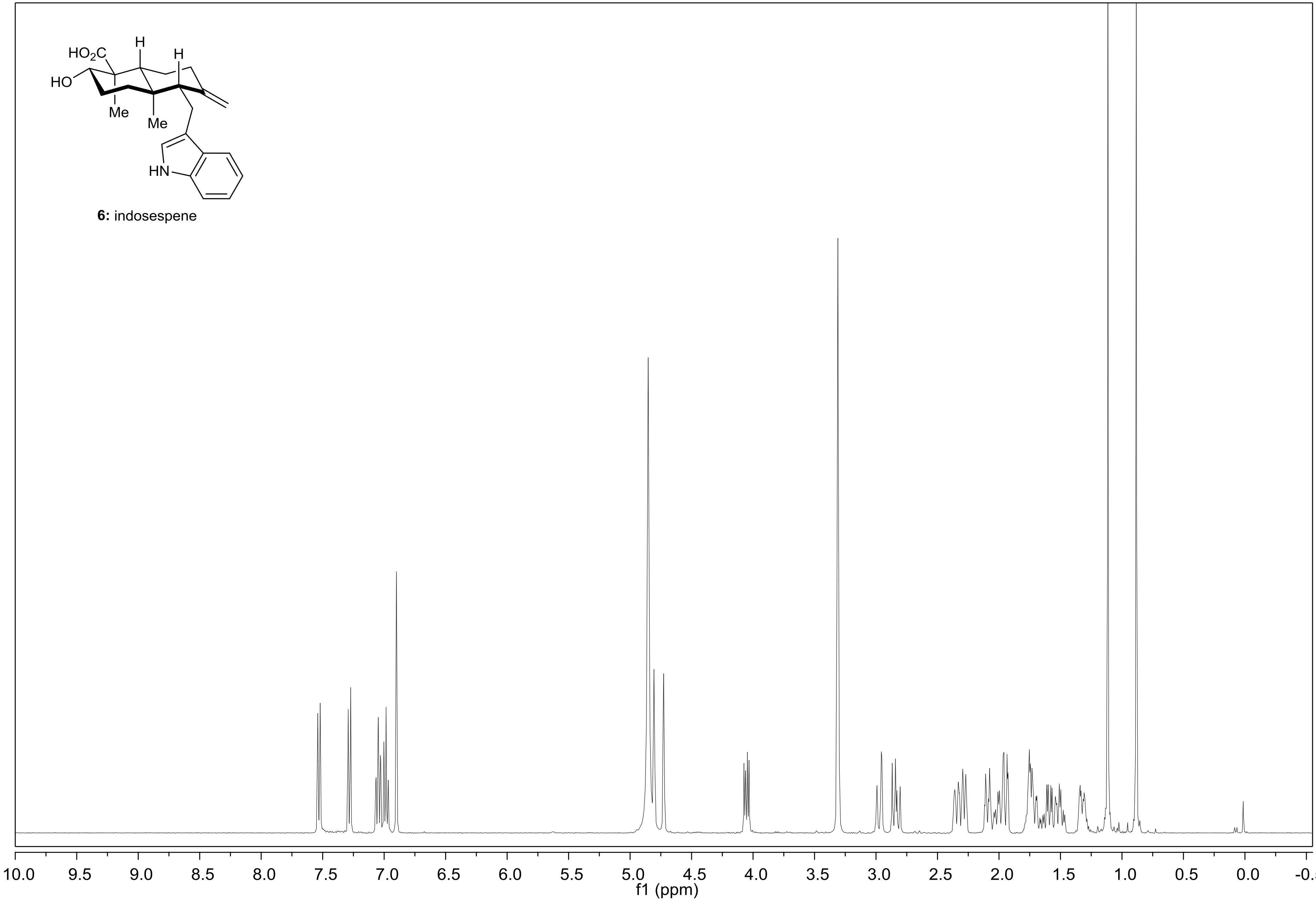

**Supplementary Figure 48. $^{13}$C NMR Spectrum of 6 (126 MHz, methanol-$d_4$)**

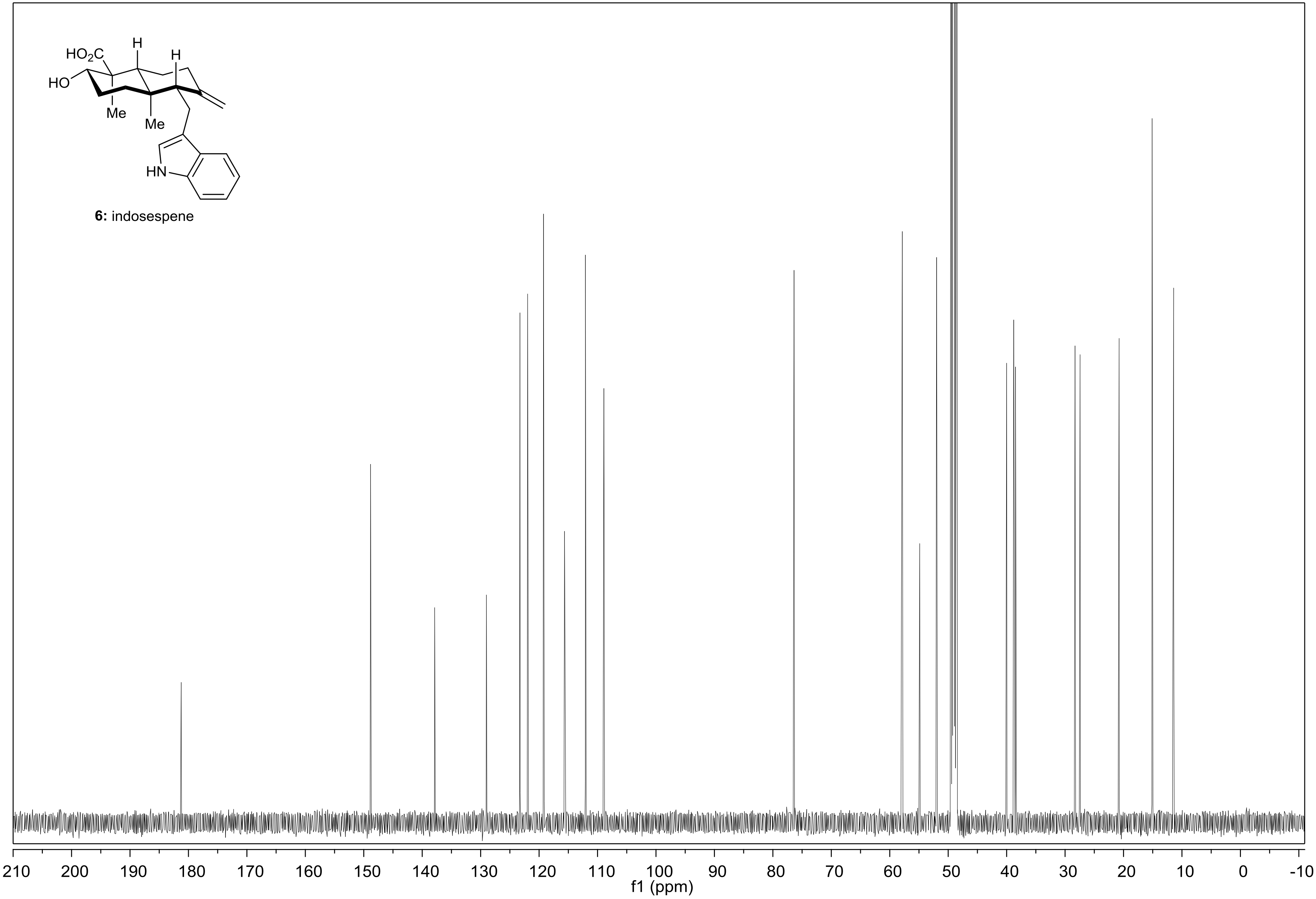

**Supplementary Figure 49. $^{1}$H NMR Spectrum of 37a (400 MHz, $CDCl_3$)**

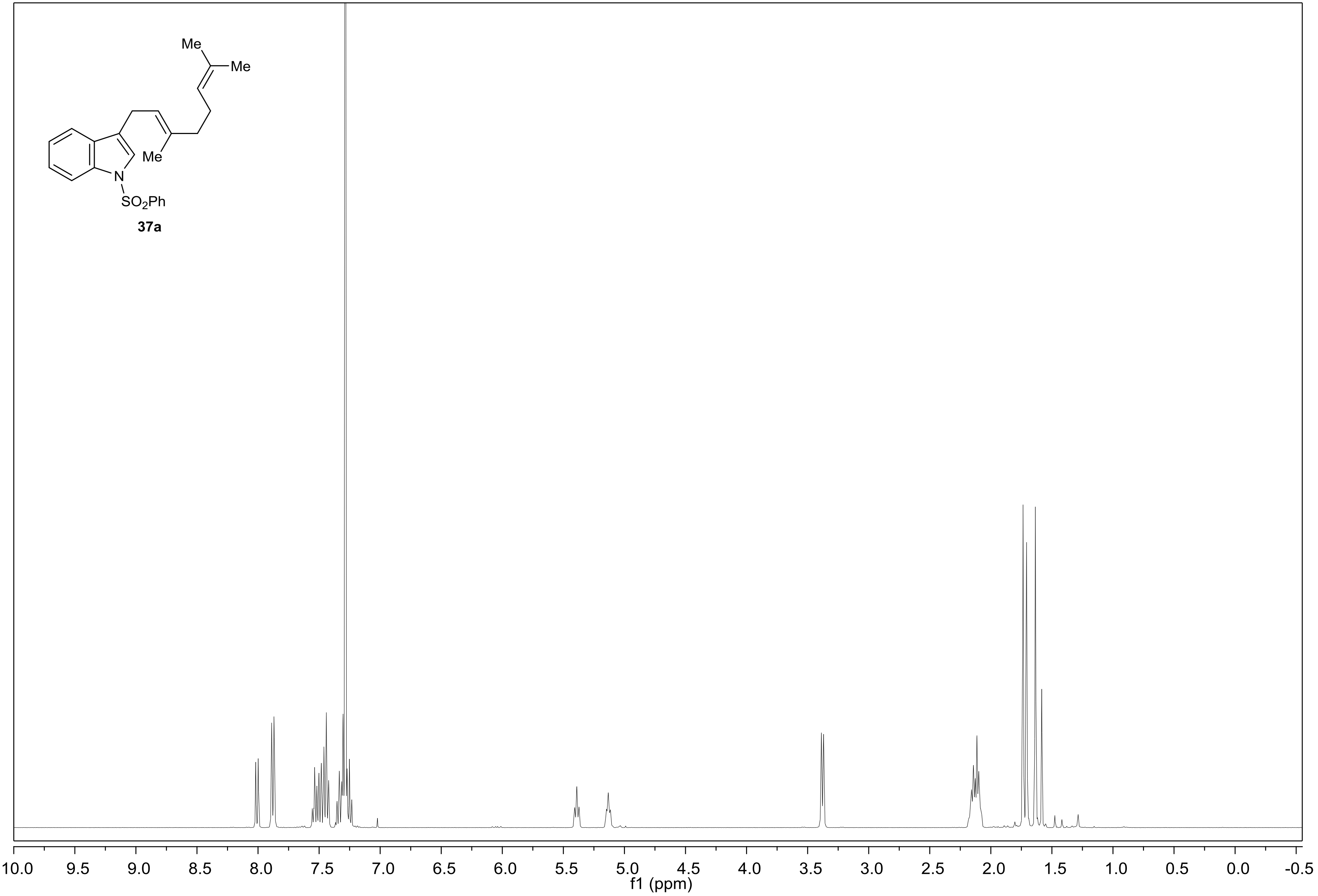

**Supplementary Figure 50. $^{13}C$ NMR Spectrum of 37a (101 MHz, $CDCl_3$)**

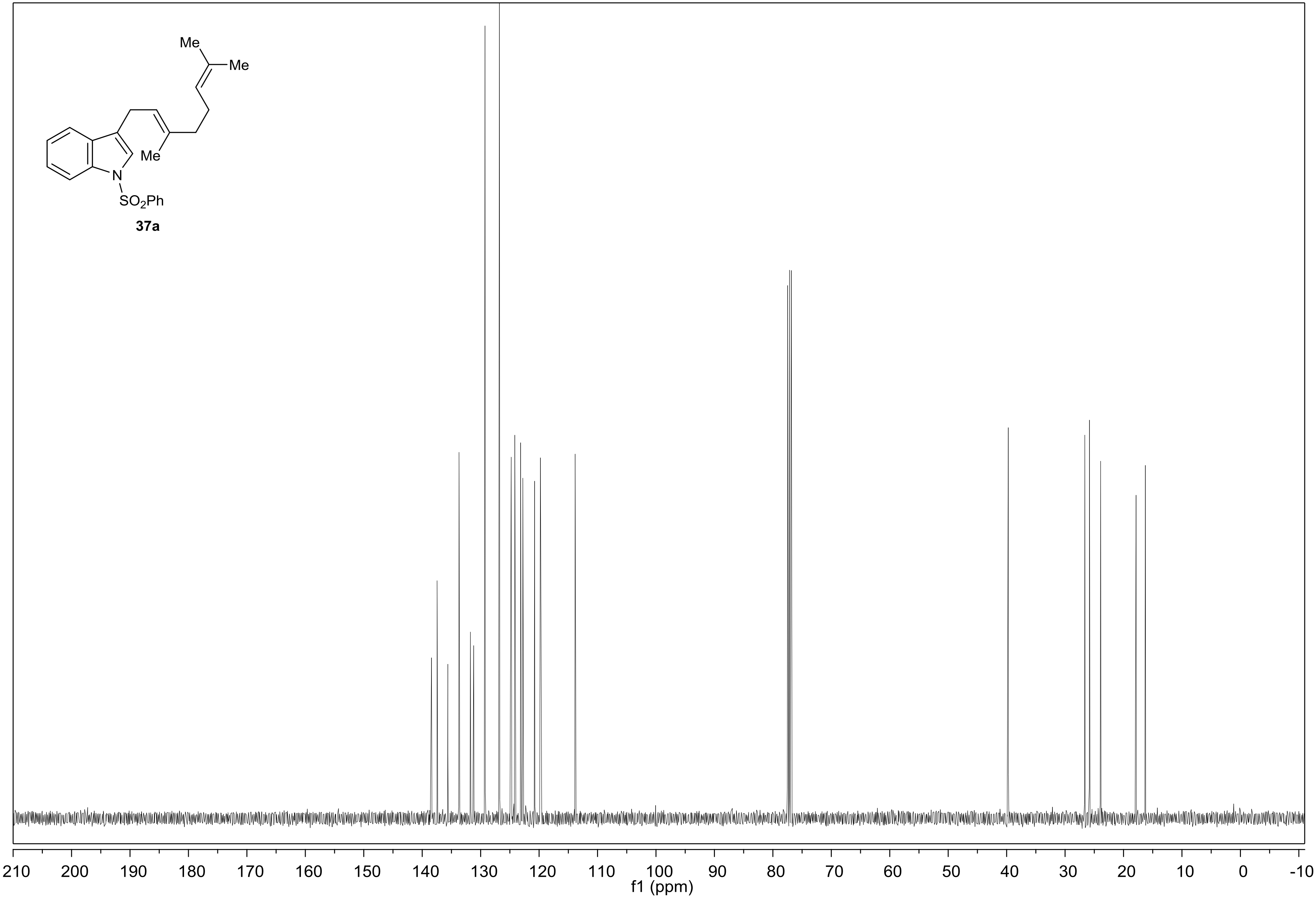

**Supplementary Figure 51. $^{1}$H NMR Spectrum of 38 (500 MHz, $CDCl_3$)**

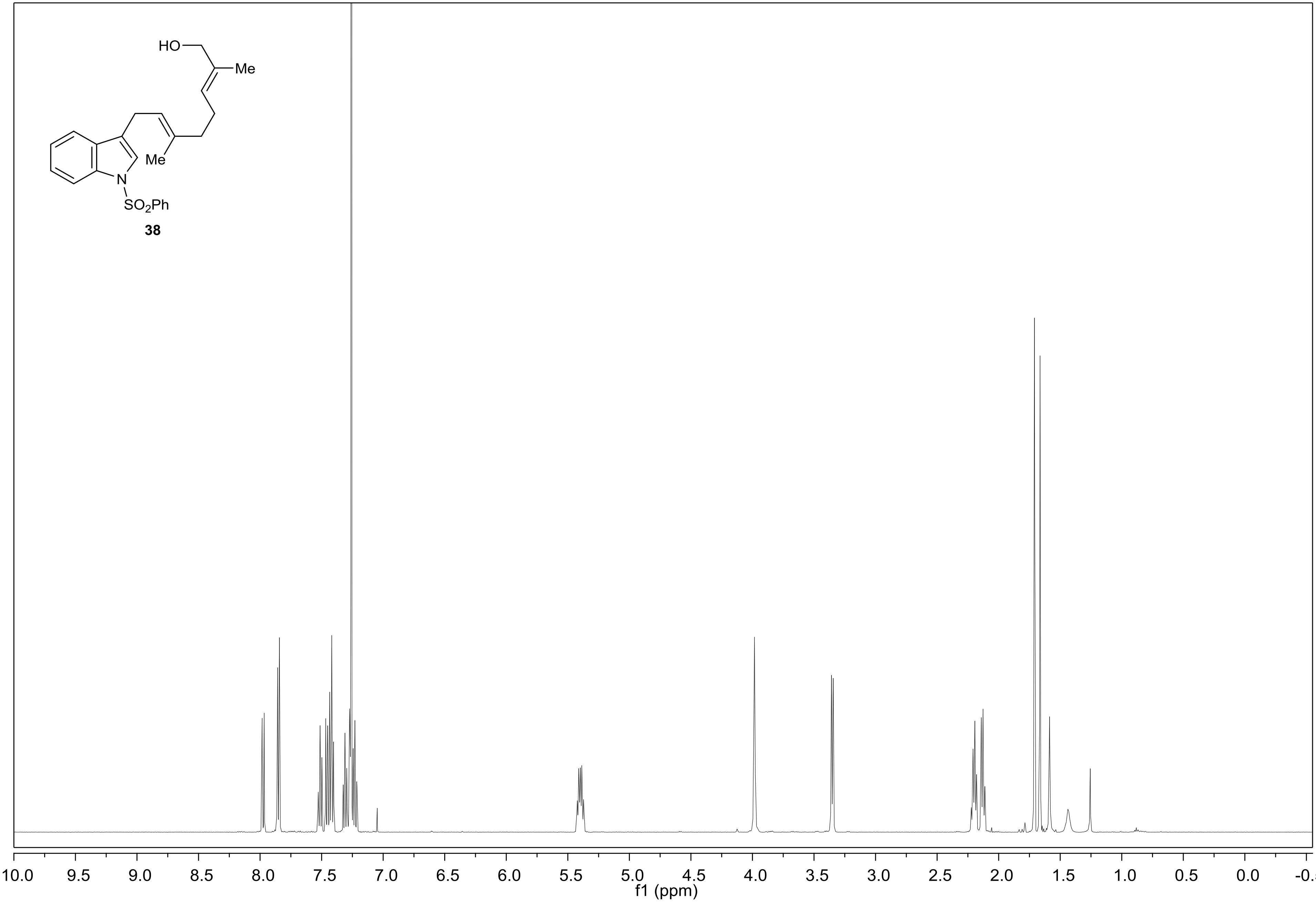

**Supplementary Figure 52. $^{13}$C NMR Spectrum of 38 (126 MHz, $CDCl_3$)**

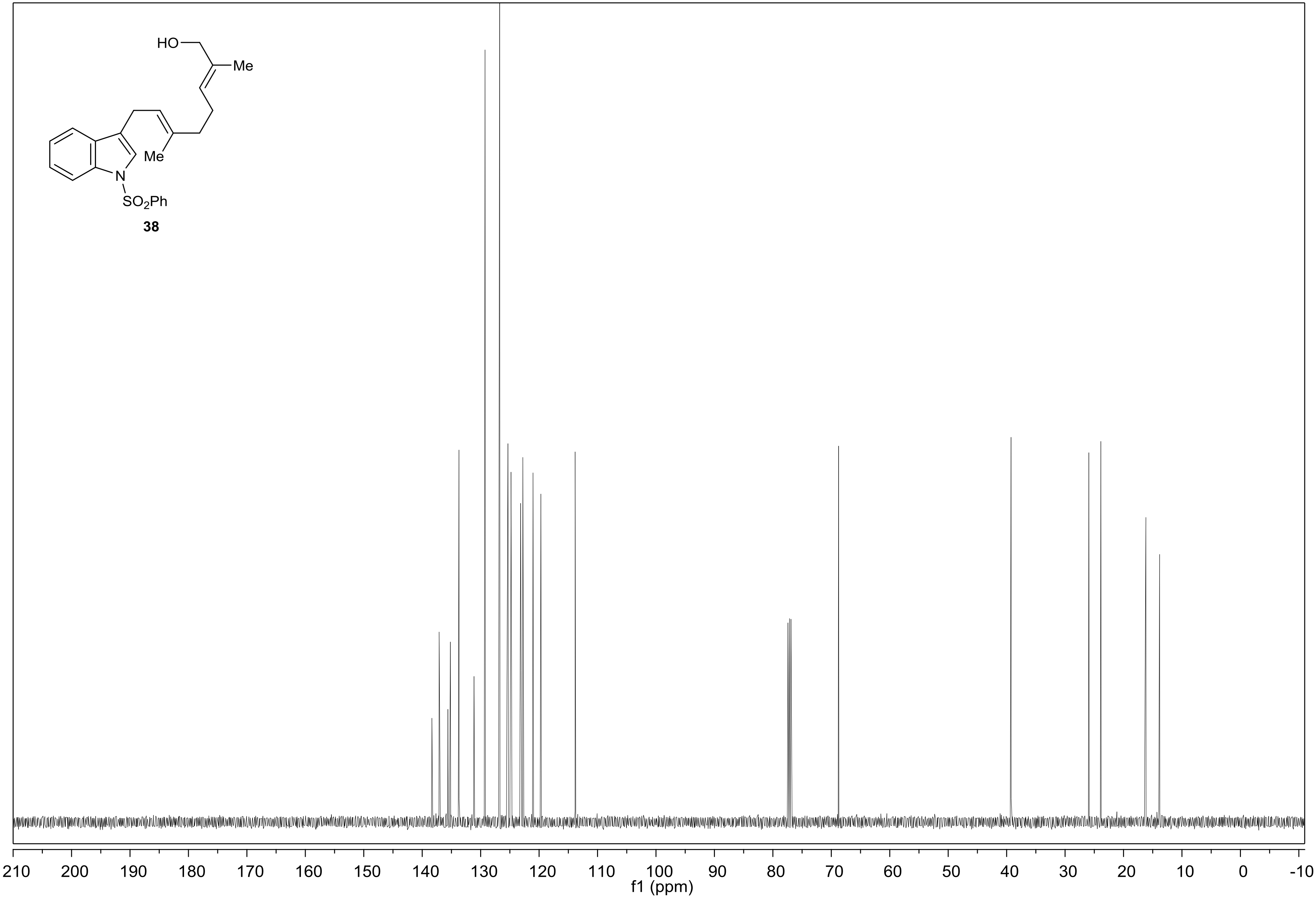

**Supplementary Figure 53. $^{1}$H NMR Spectrum of 39 (400 MHz, $CDCl_3$)**

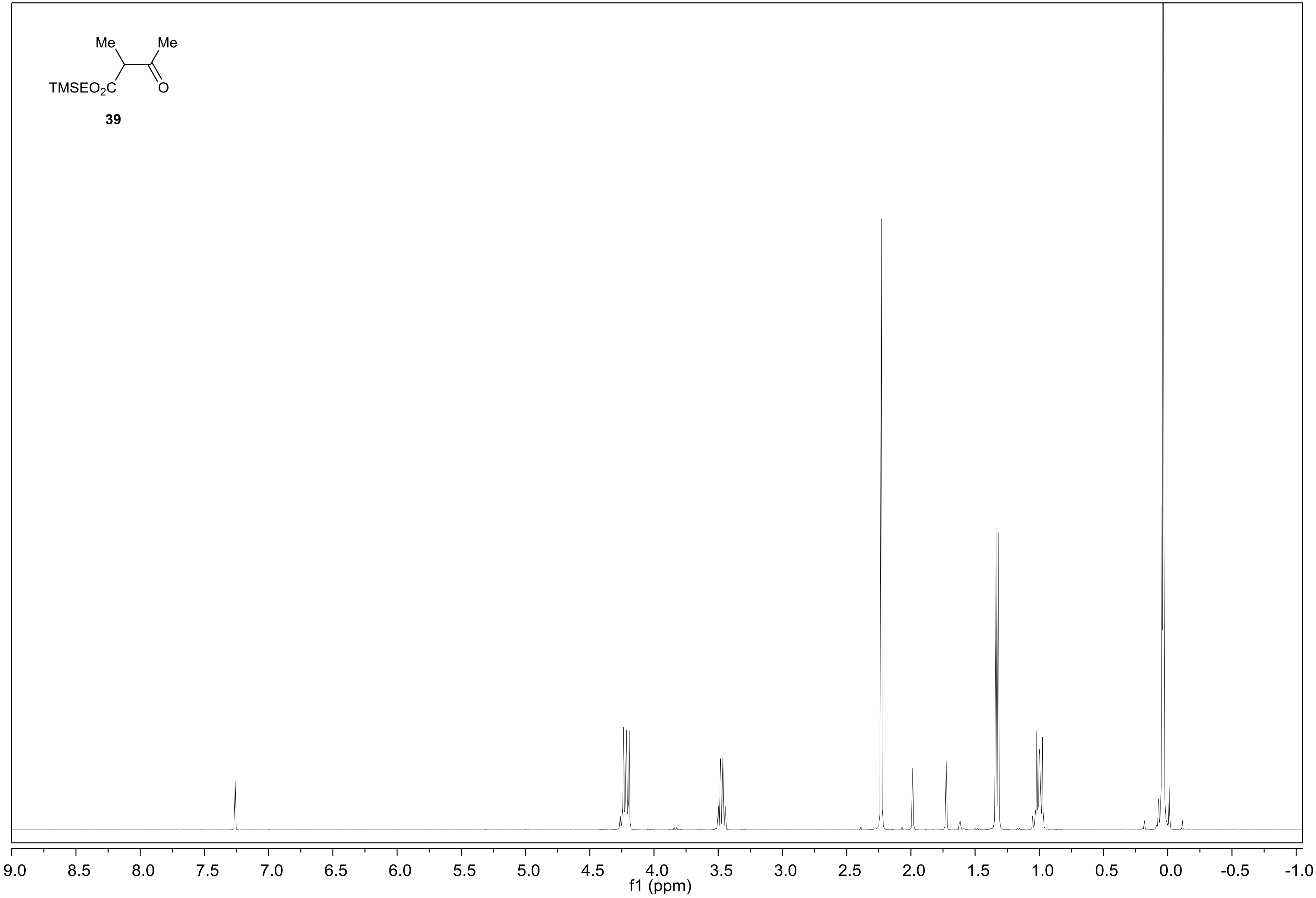

**Supplementary Figure 54.** $^{13}$**C NMR Spectrum of 39 (101 MHz,** $CDCl_3$**)**

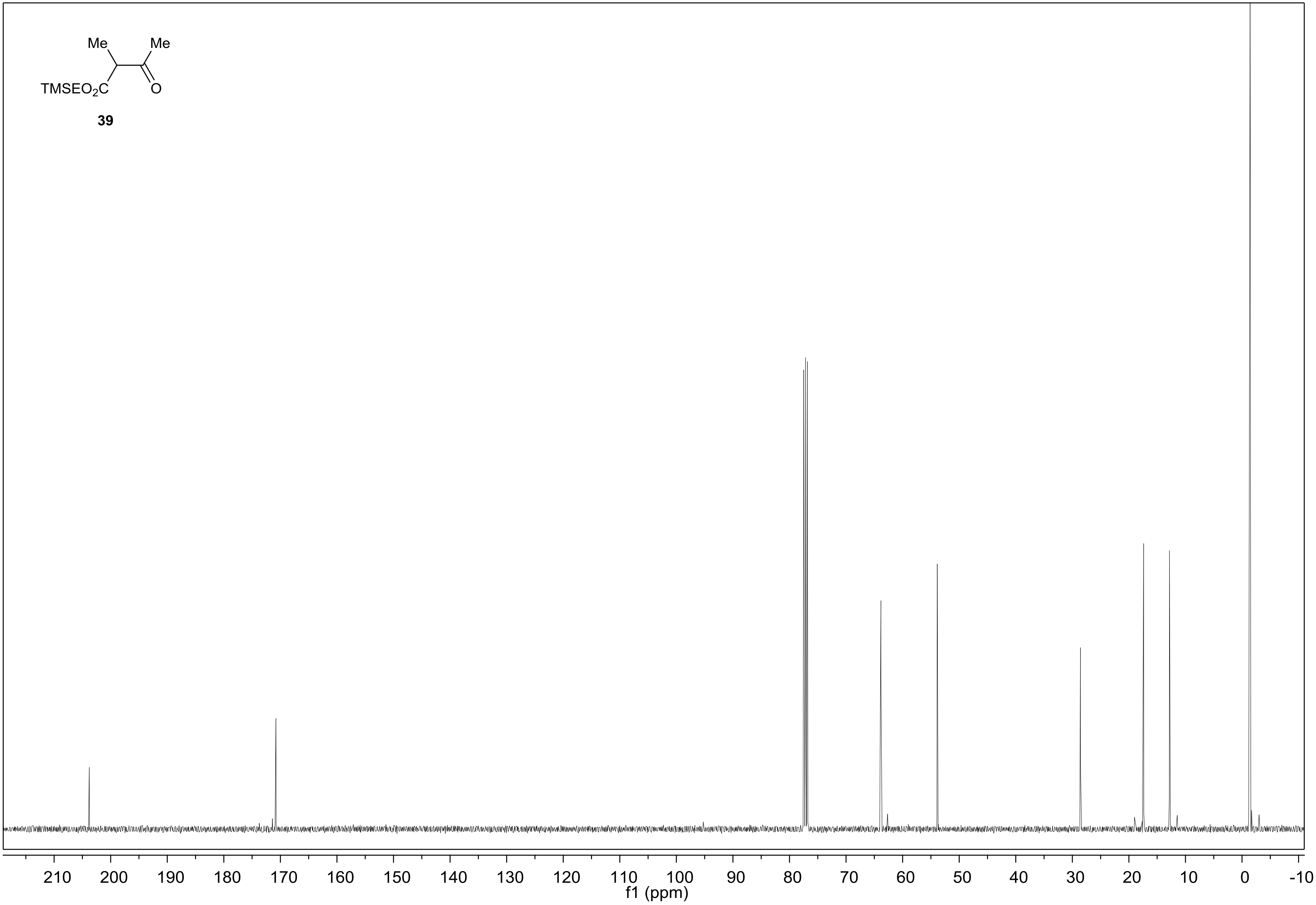

**Supplementary Figure 55. $^1$H NMR Spectrum of 36 (400 MHz, $CDCl_3$)**

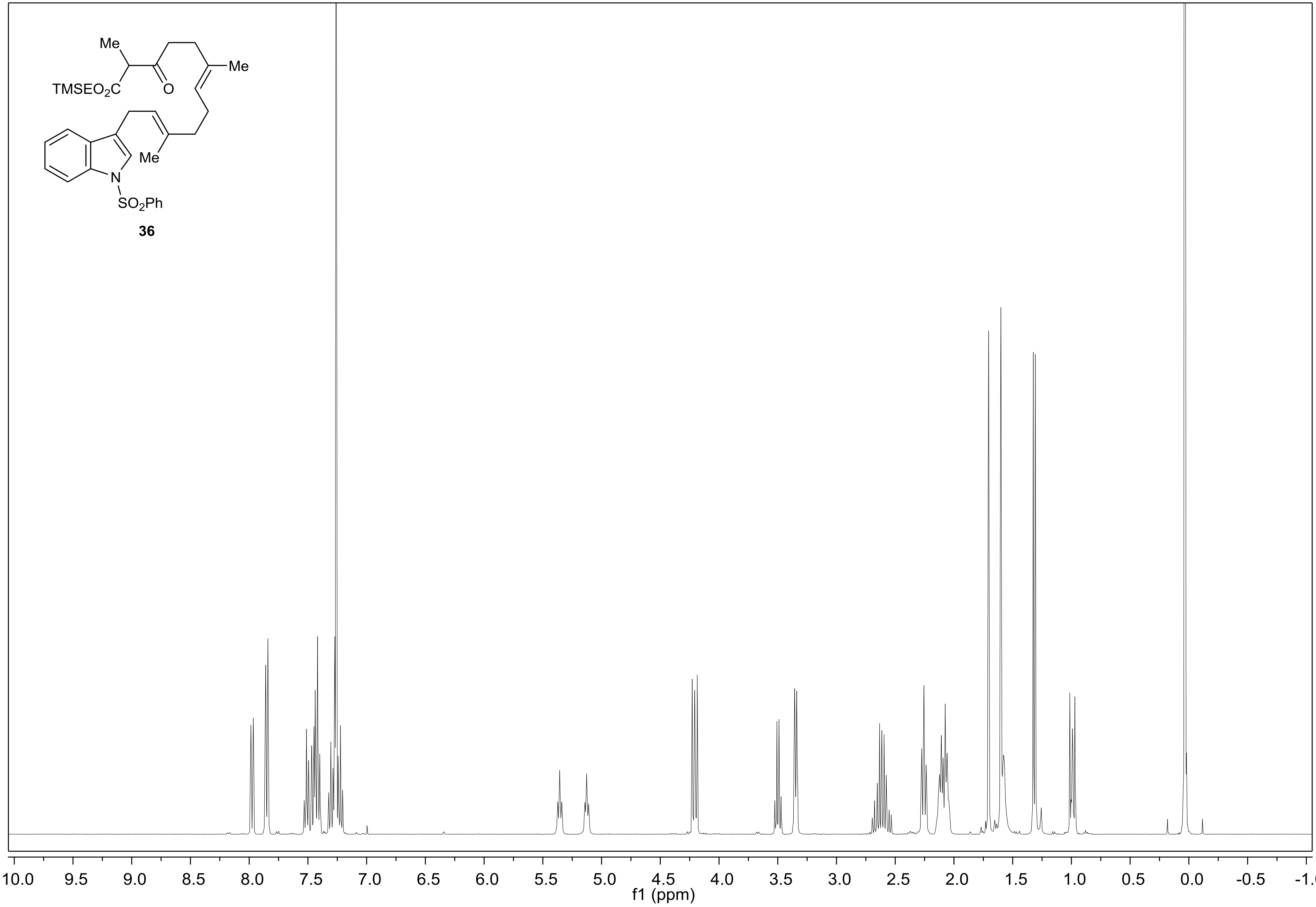

**Supplementary Figure 56. $^{13}$C NMR Spectrum of 36 (101 MHz, $CDCl_3$)**

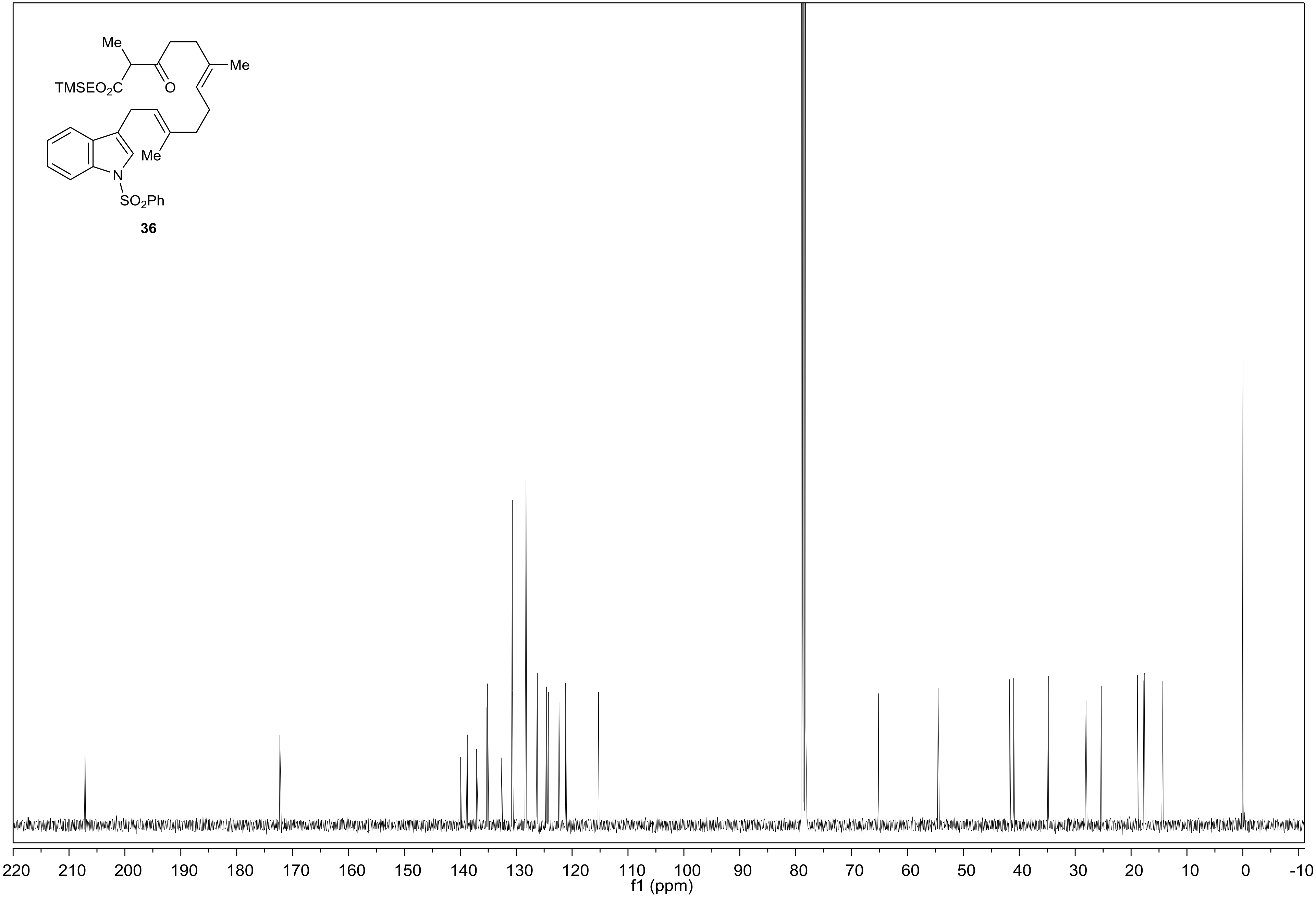

**Supplementary Figure 57. $^1$H NMR Spectrum of 40 (400 MHz, $CDCl_3$)**

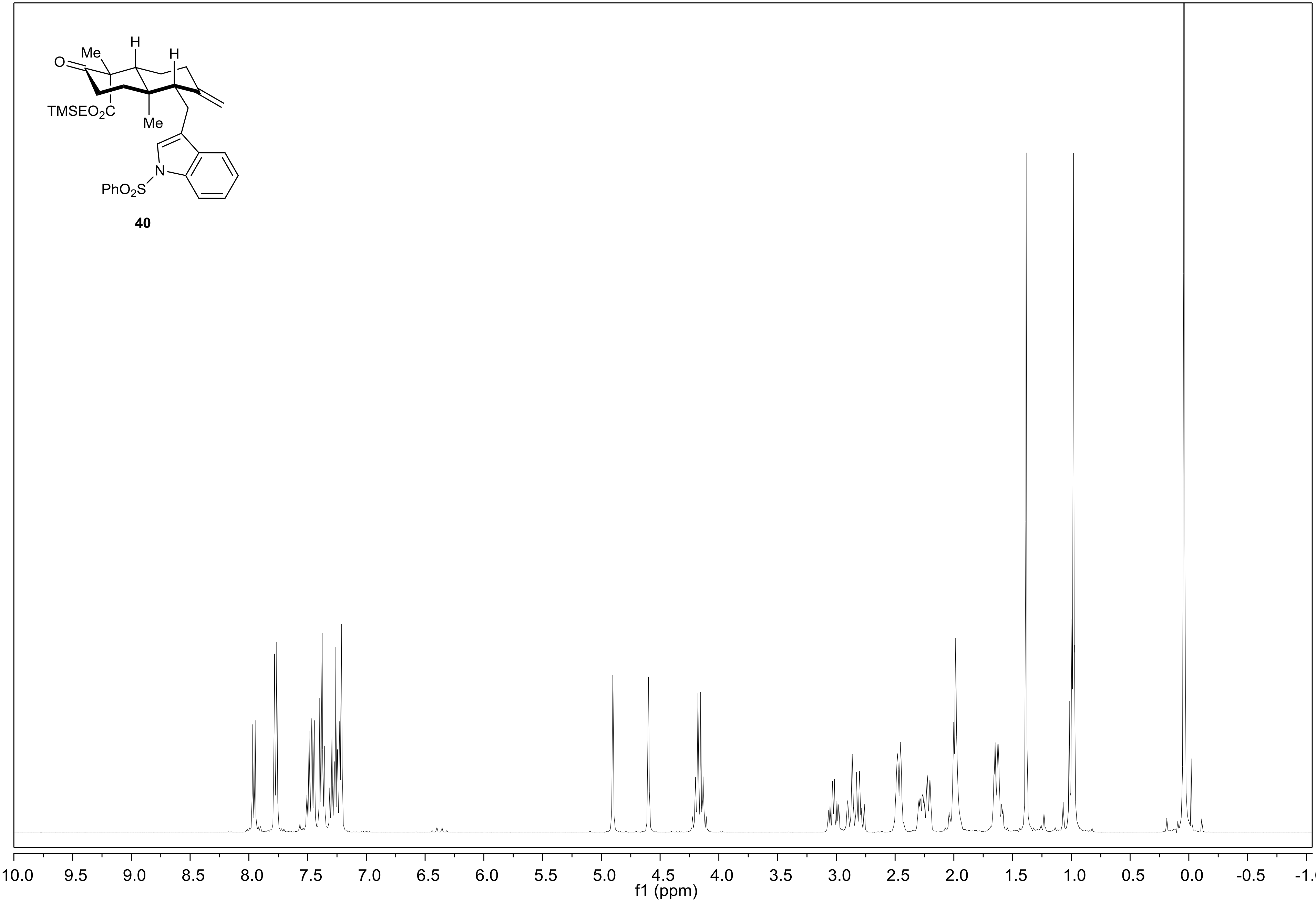

**Supplementary Figure 58.** $^{13}$**C NMR Spectrum of 40 (101 MHz,** $CDCl_3$**)**

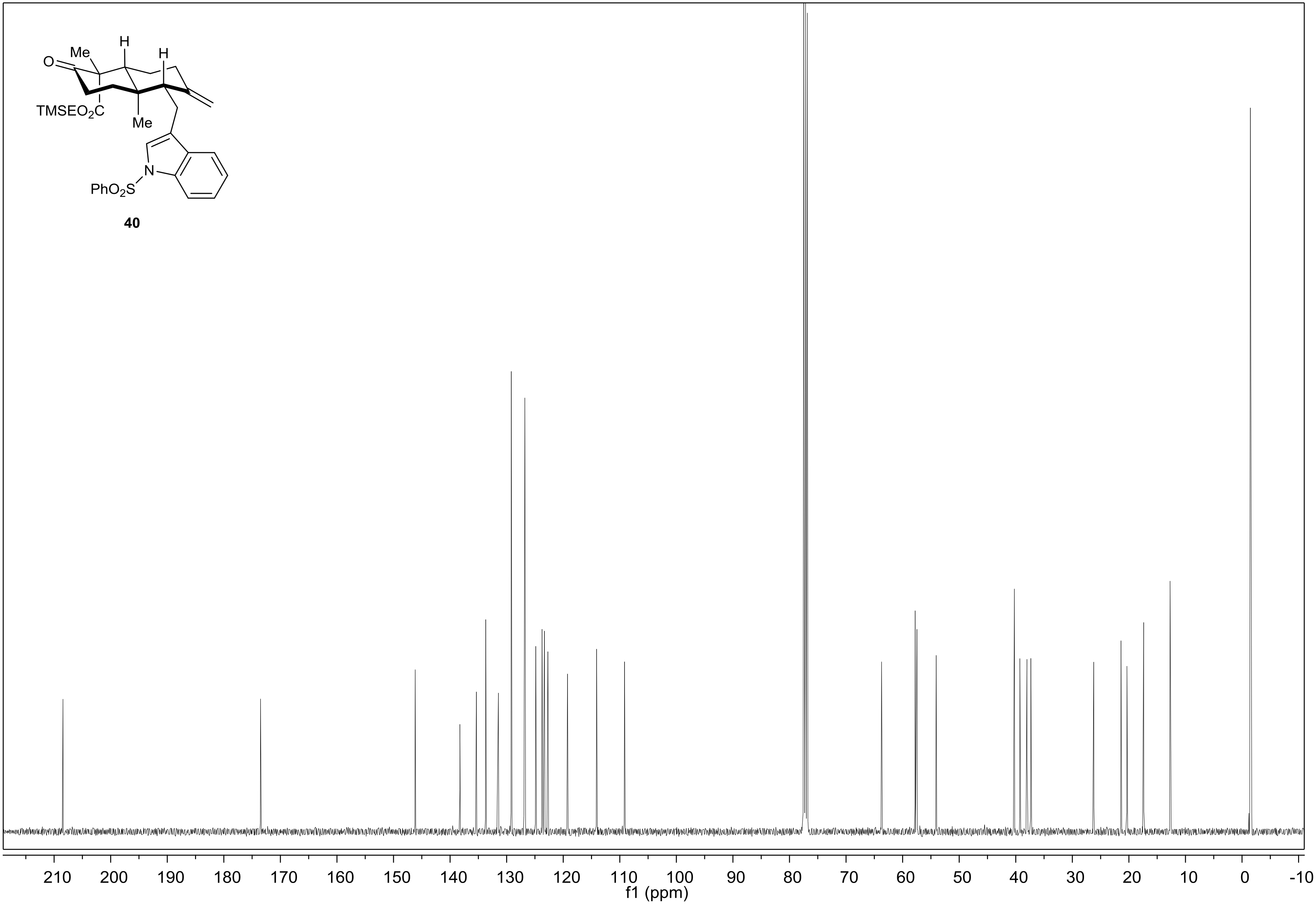

**Supplementary Figure 59. $^{1}$H NMR Spectrum of 40a (400 MHz, $CDCl_3$)**

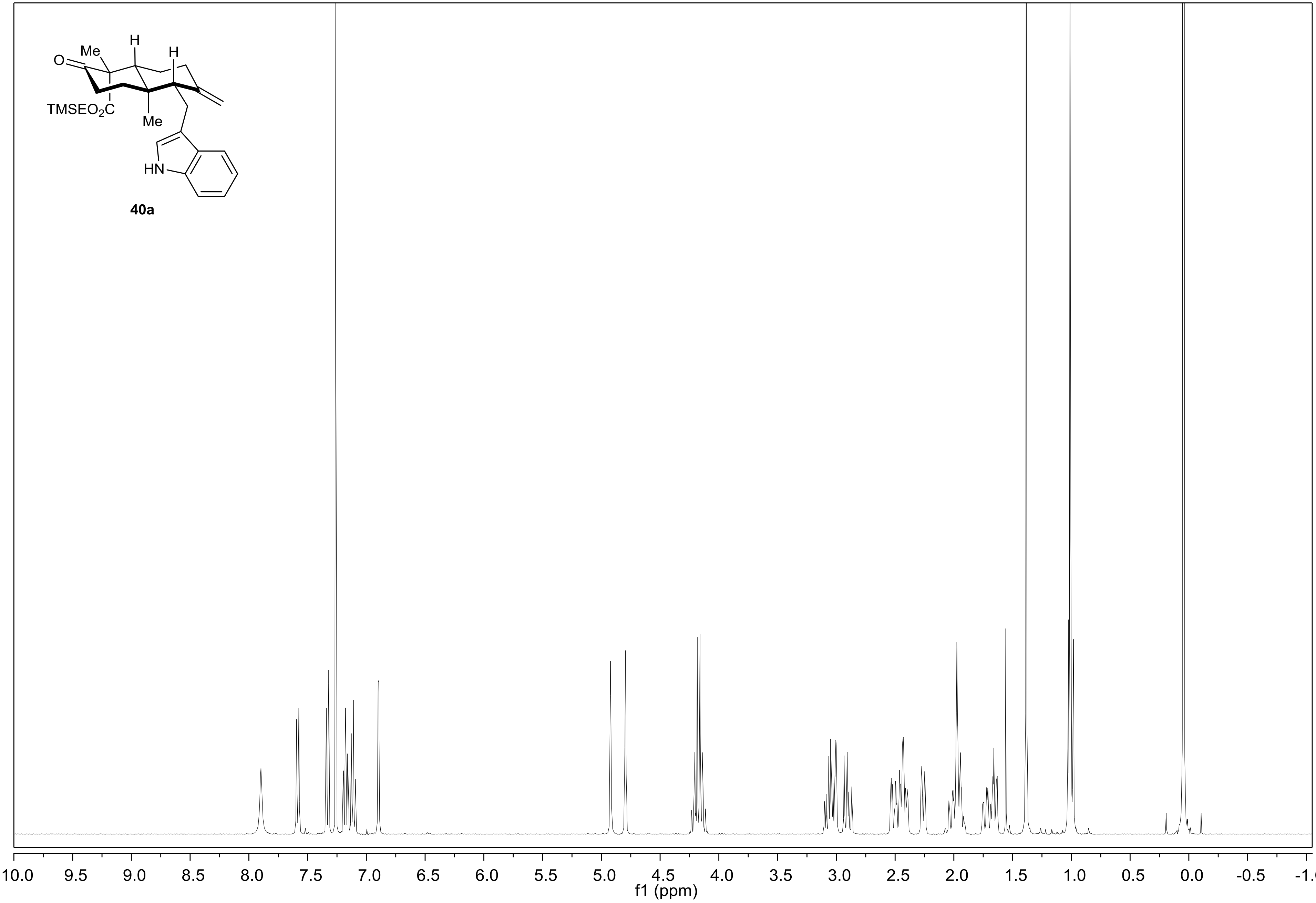

**Supplementary Figure 60. $^{13}C$ NMR Spectrum of 40a (126 MHz, $CDCl_3$)**

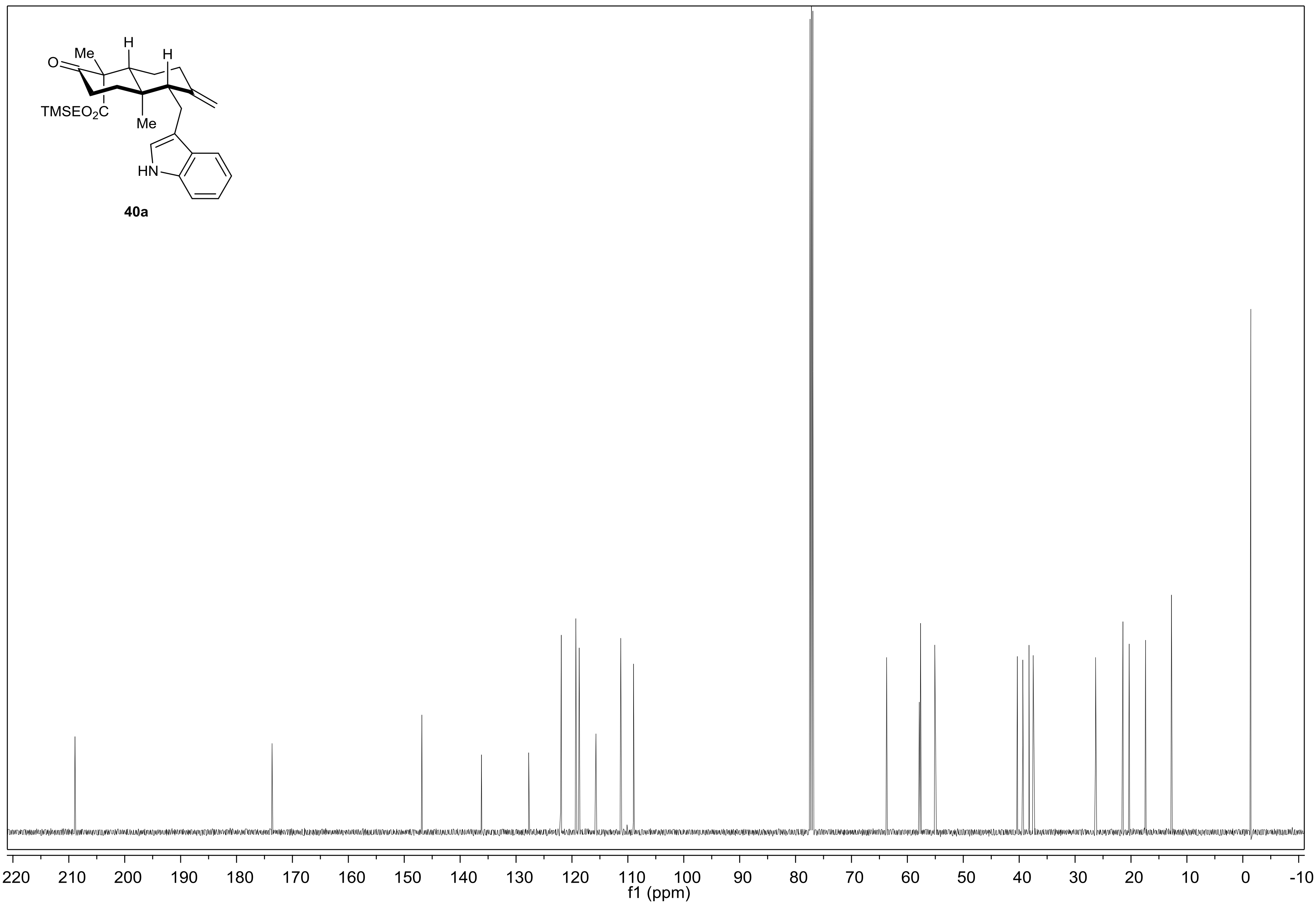

**Supplementary Figure 61. $^1H$ NMR Spectrum of 41 (500 MHz, $CDCl_3$)**

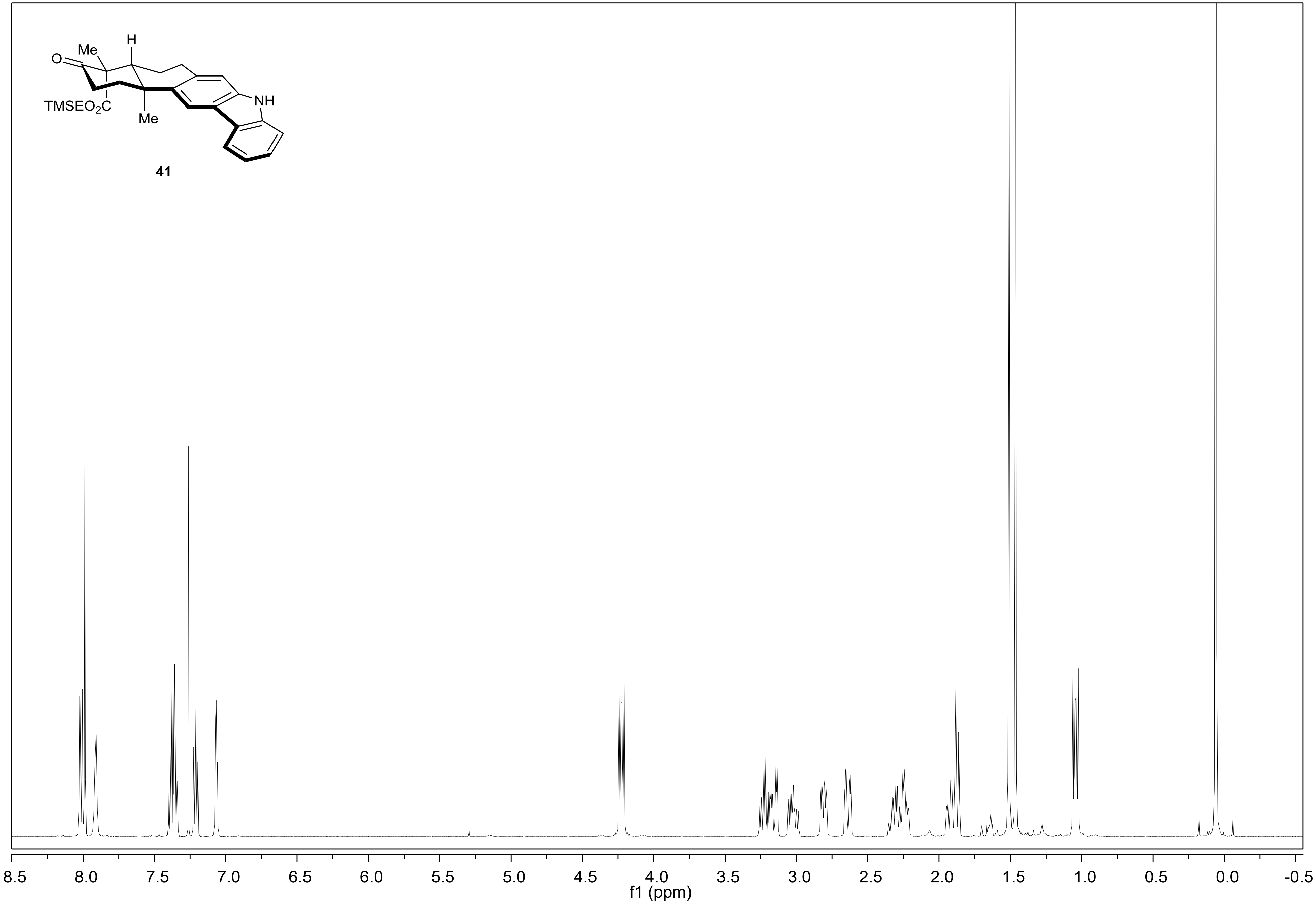

**Supplementary Figure 62. $^{13}$C NMR Spectrum of 41 (126 MHz, $CDCl_3$)**

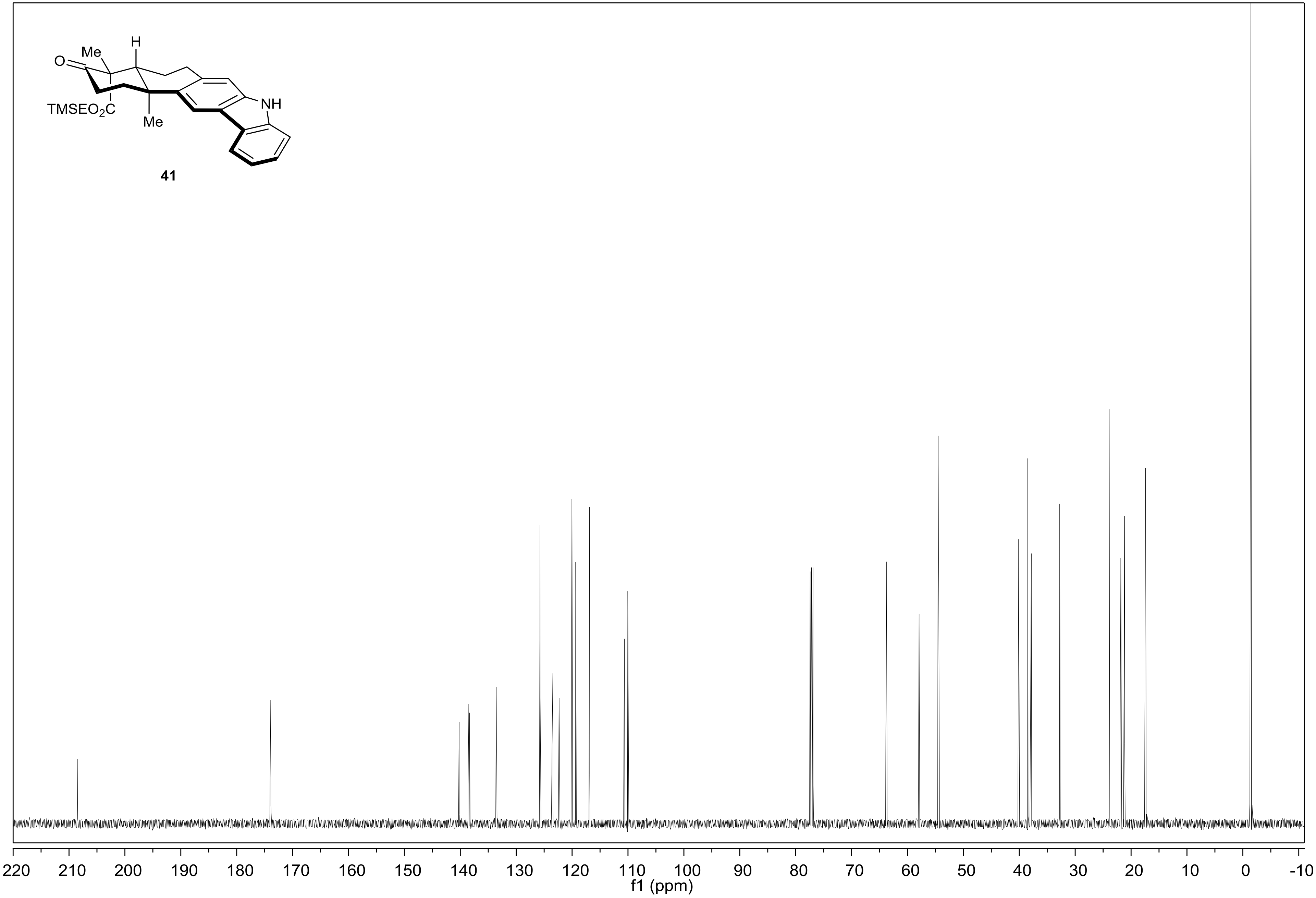

**Supplementary Figure 63. $^1$H NMR Spectrum of 42a (400 MHz, $CDCl_3$)**

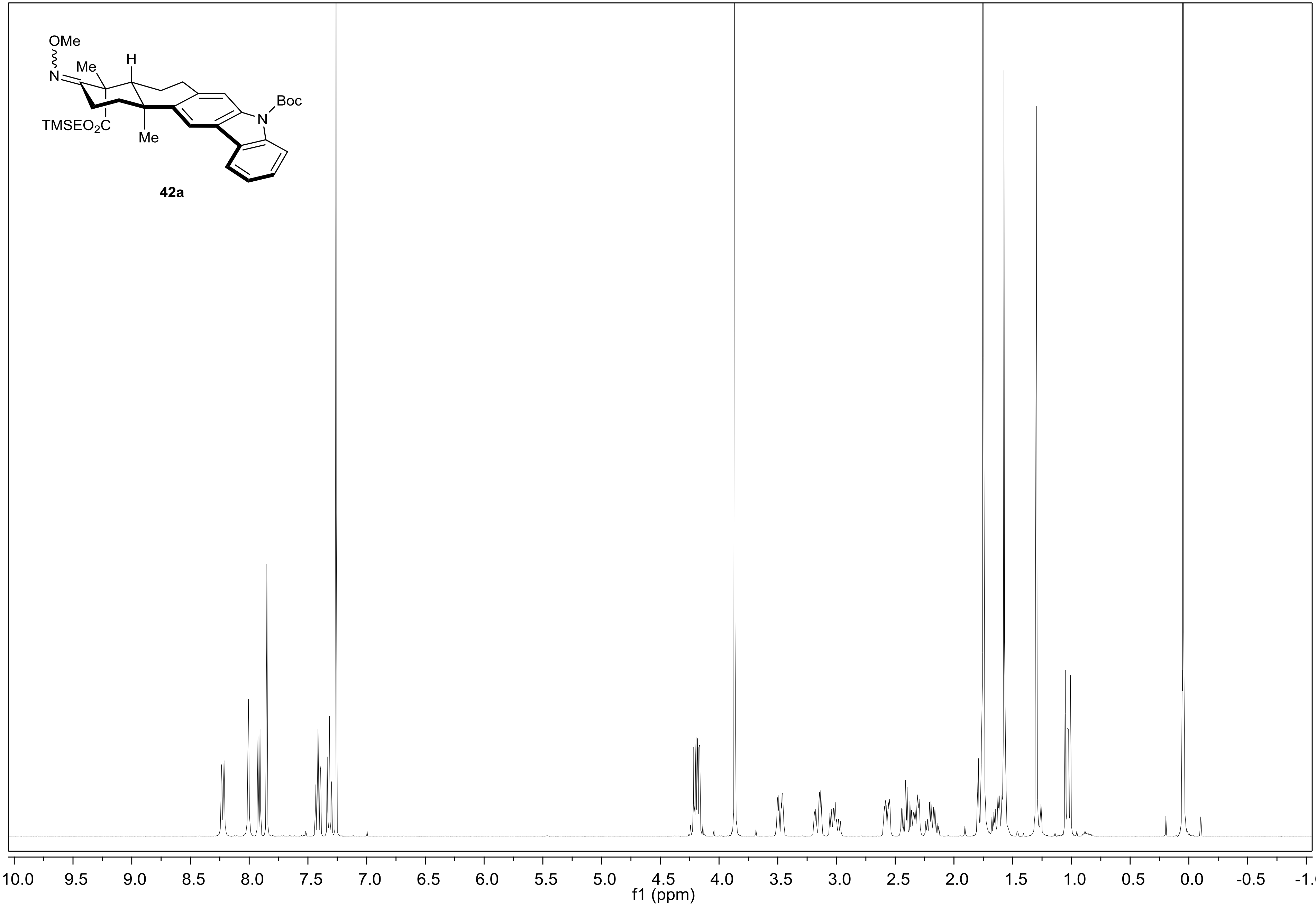

**Supplementary Figure 64.** $^{13}$**C NMR Spectrum of 42a (101 MHz,** $CDCl_3$**)**

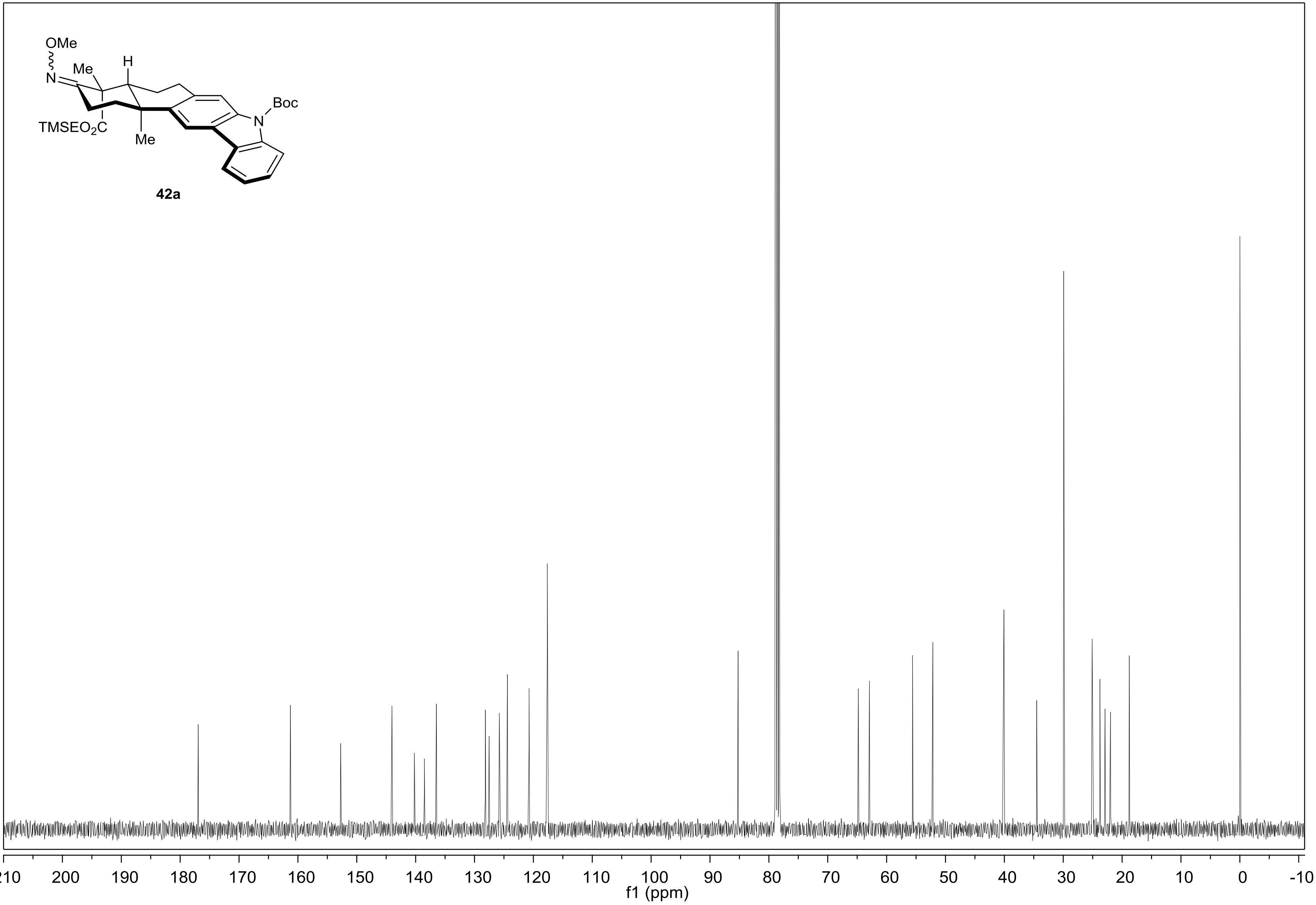

**Supplementary Figure 65. $^{1}$H NMR Spectrum of 42 (400 MHz, $CDCl_3$)**

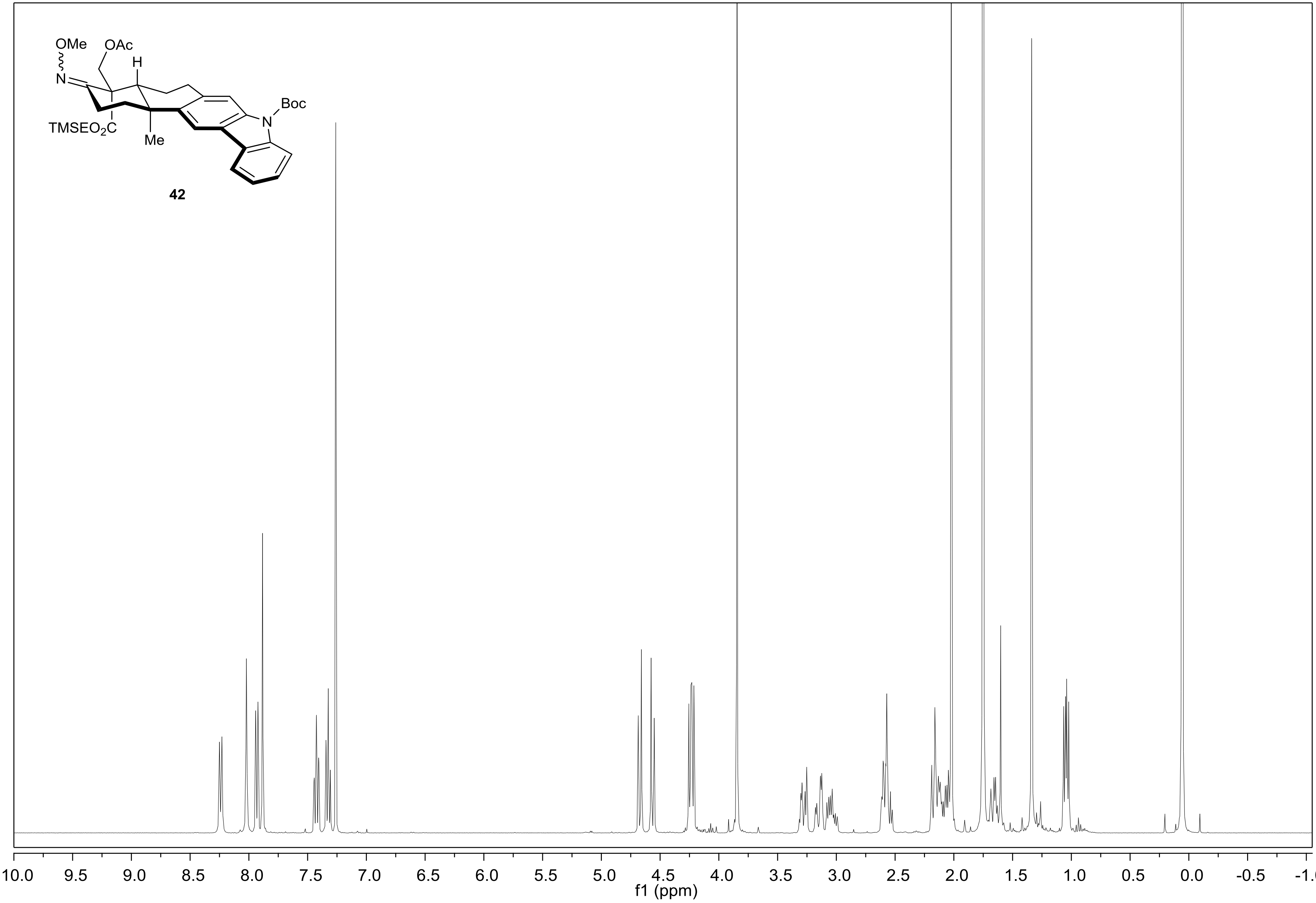

**Supplementary Figure 66.** $^{13}$**C NMR Spectrum of 42 (126 MHz, CDCl$_3$)**

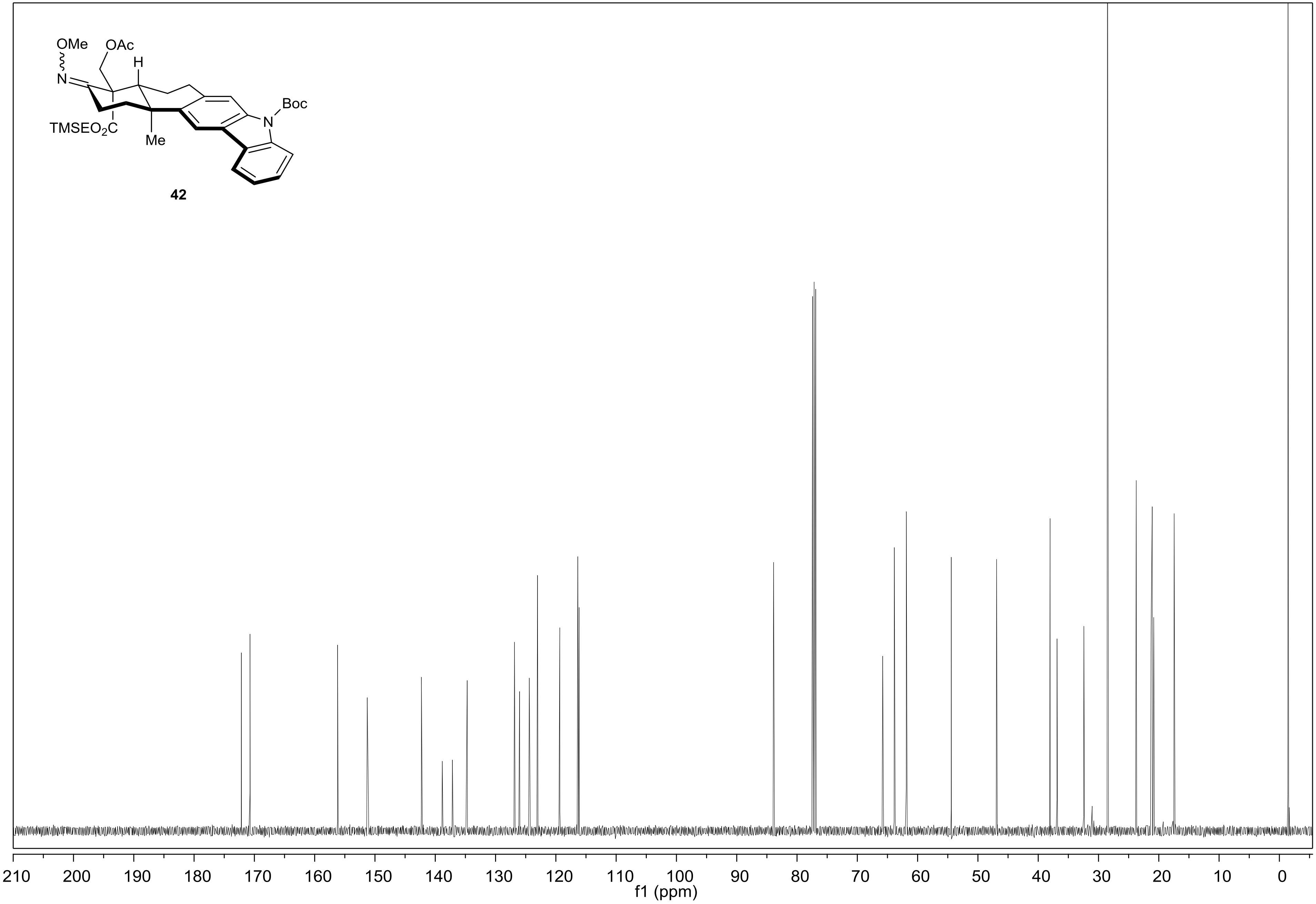

**Supplementary Figure 67. $^{1}$H NMR Spectrum of 45 (600 MHz, $CDCl_3$)**

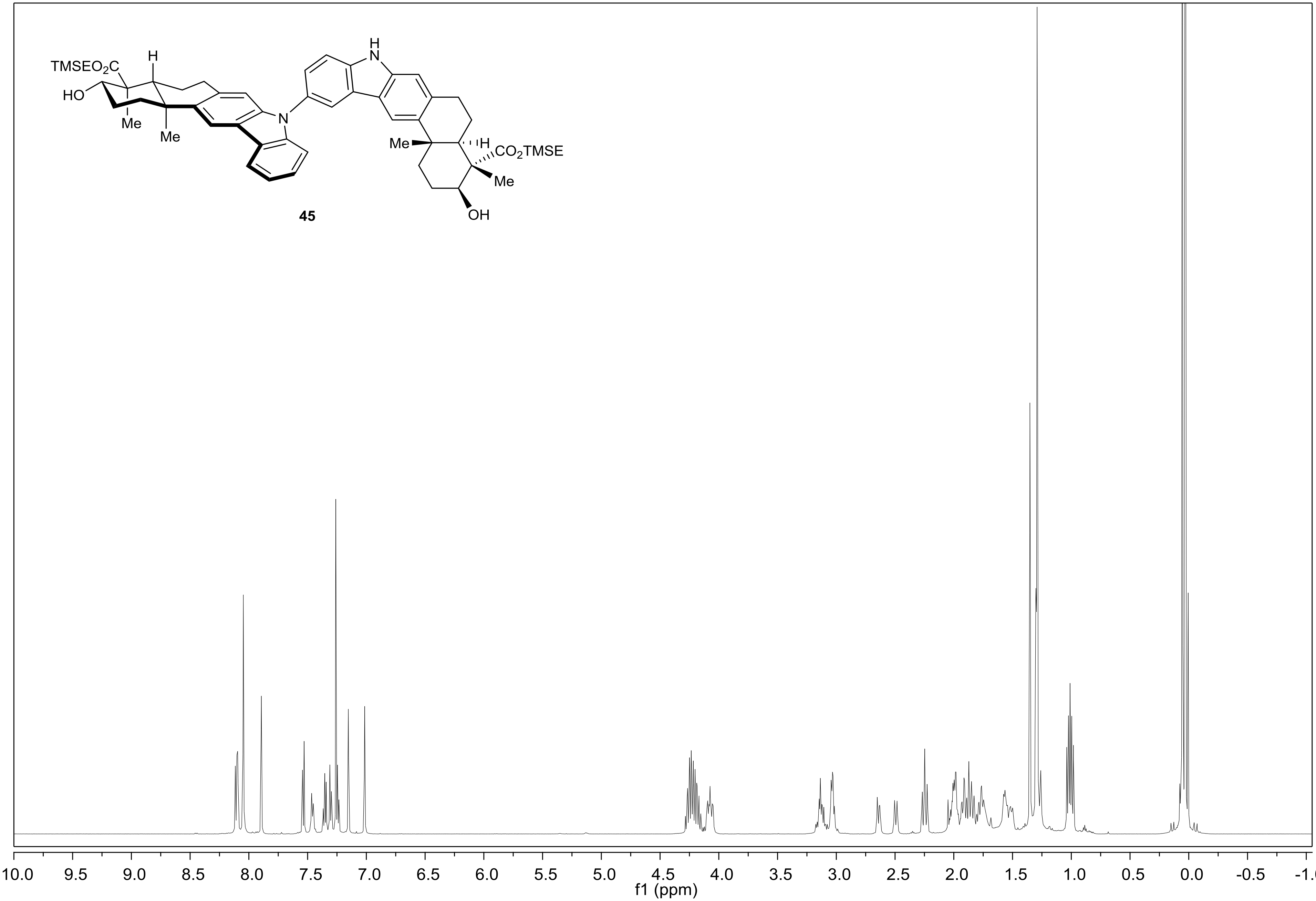

**Supplementary Figure 68.** $^{13}$**C NMR Spectrum of 45 (151 MHz, CDCl$_3$)**

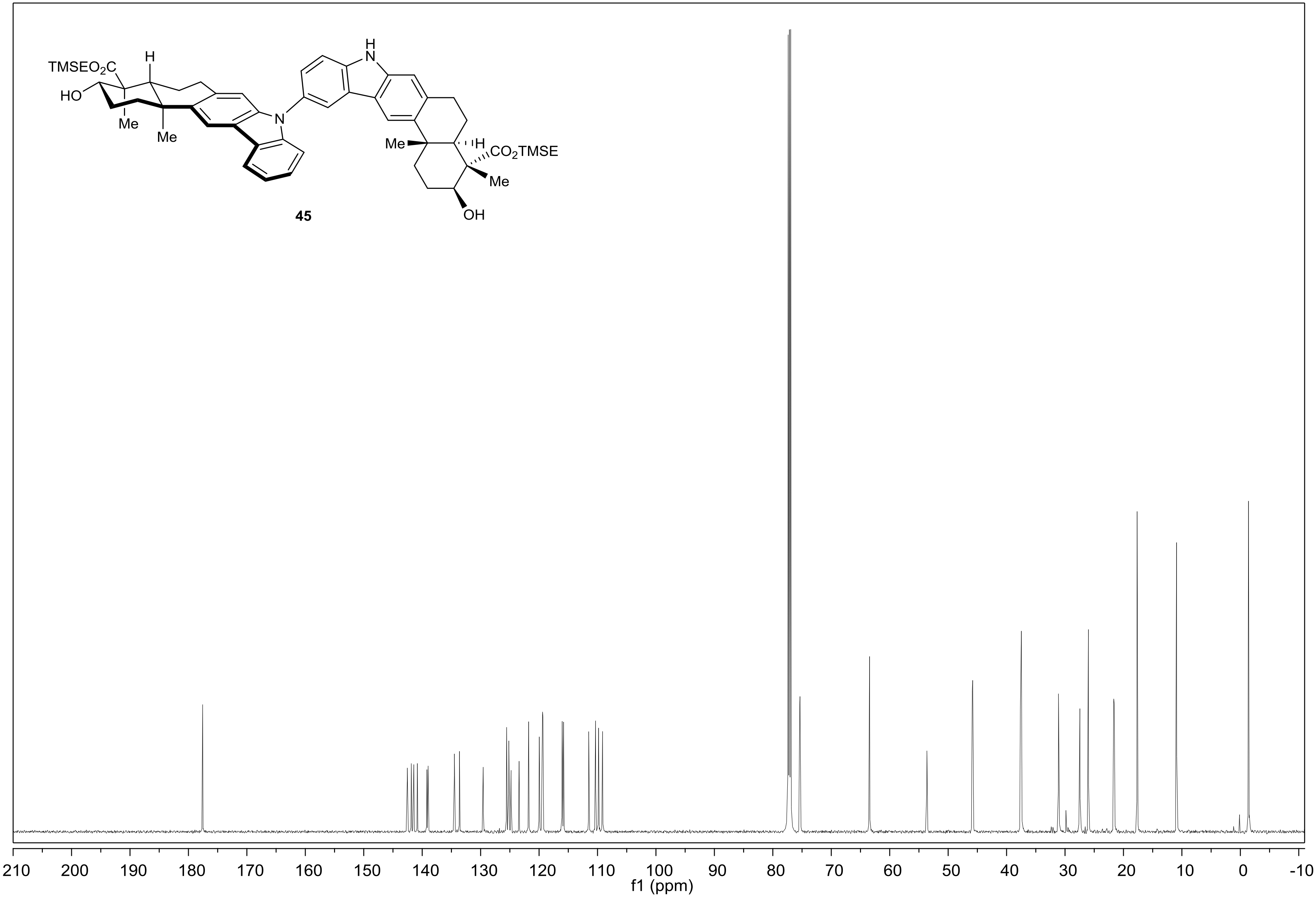

**Supplementary Figure 69. $^{1}$H NMR Spectrum of 4 (600 MHz, methanol-$d_4$)**

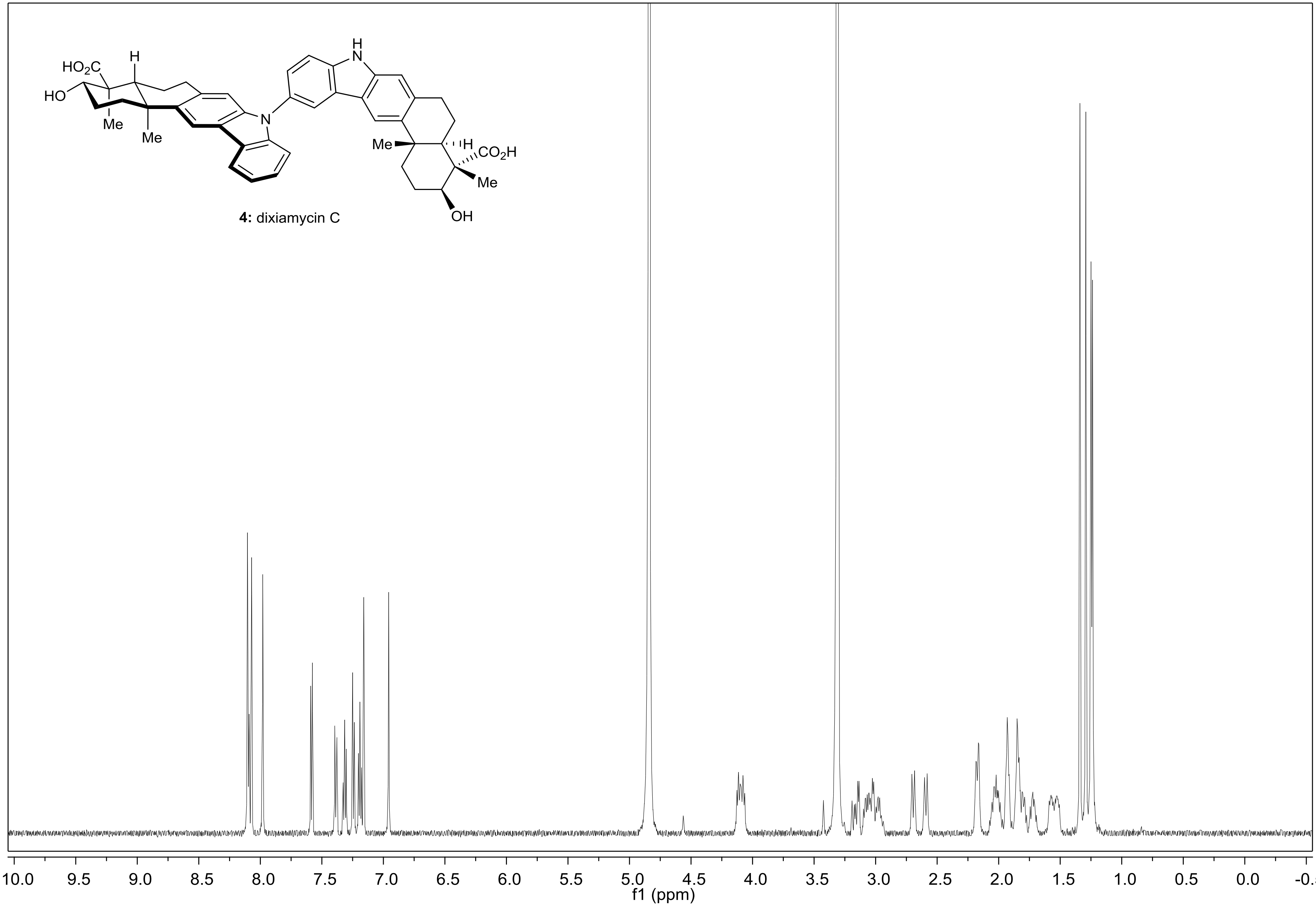

**Supplementary Figure 70.** $^{13}$**C NMR Spectrum of 4 (151 MHz, methanol-d$_4$)**

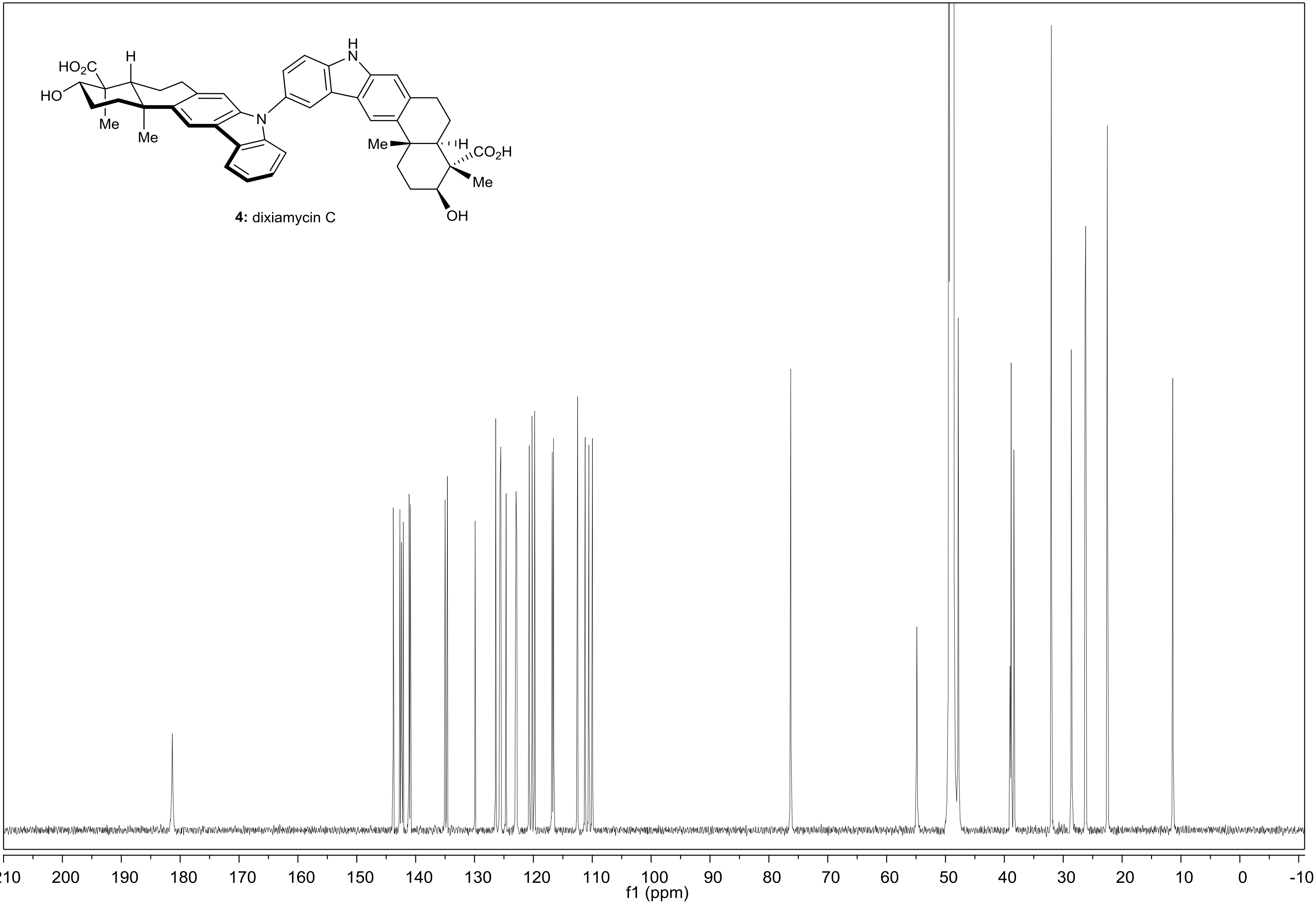

**Supplementary Figure 71. Comparison of the $^1$H NMR Spectra of Natural and Synthetic Indosespene**

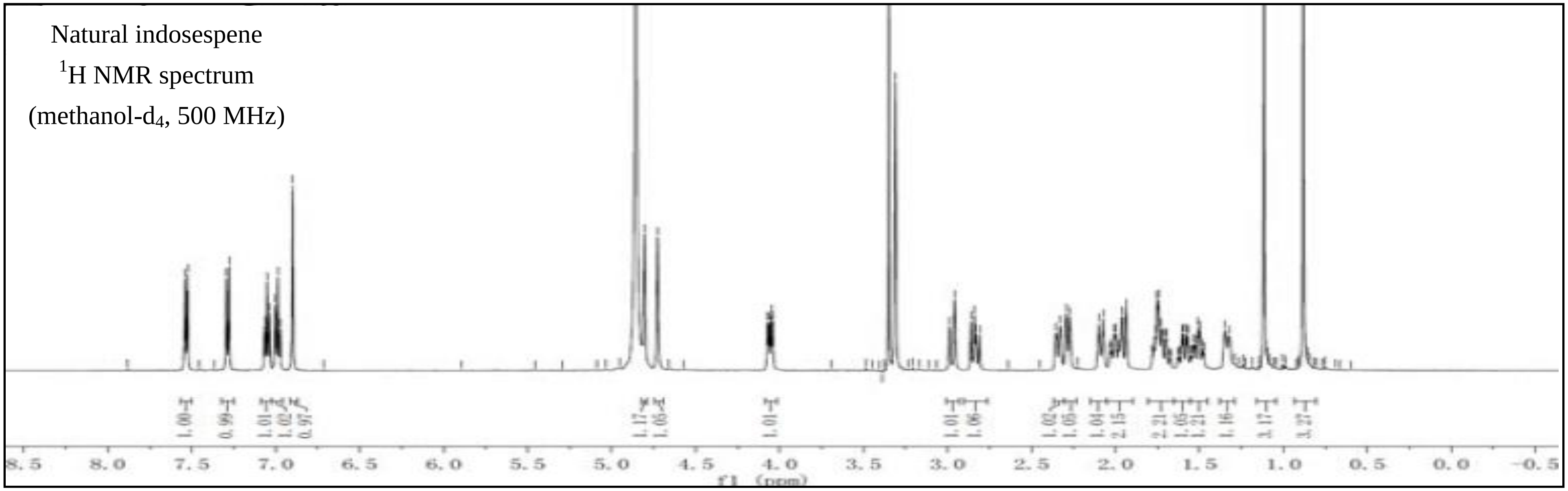


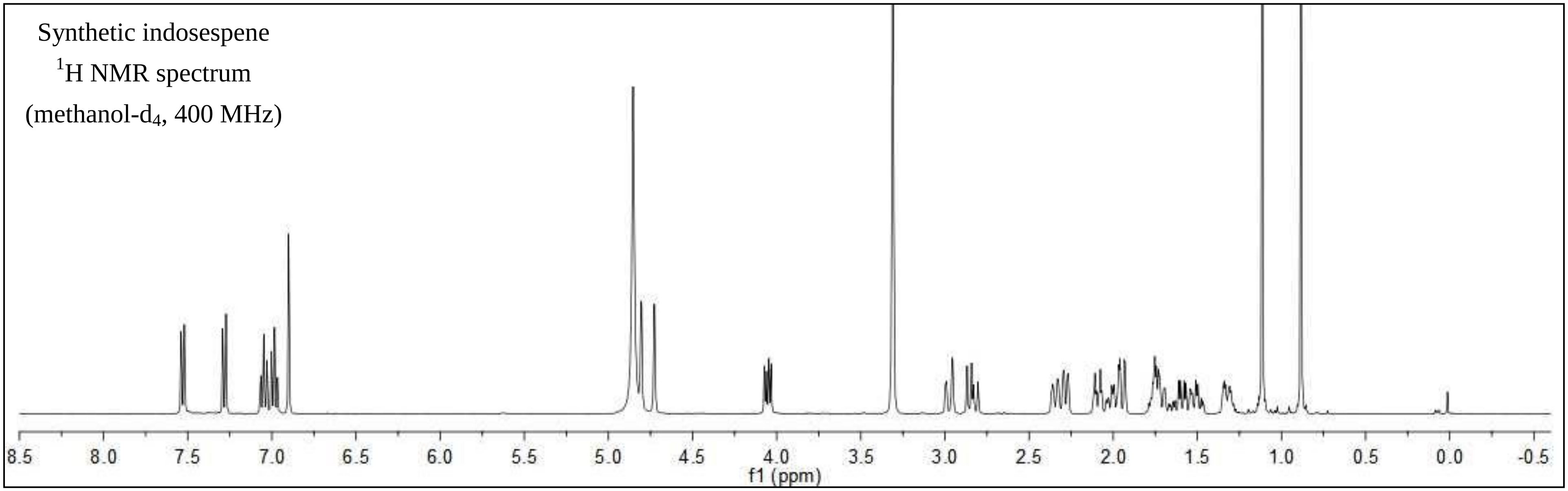

**Supplementary Figure 72. Comparison of the $^{13}$C NMR Spectra of Natural and Synthetic Indosespene**

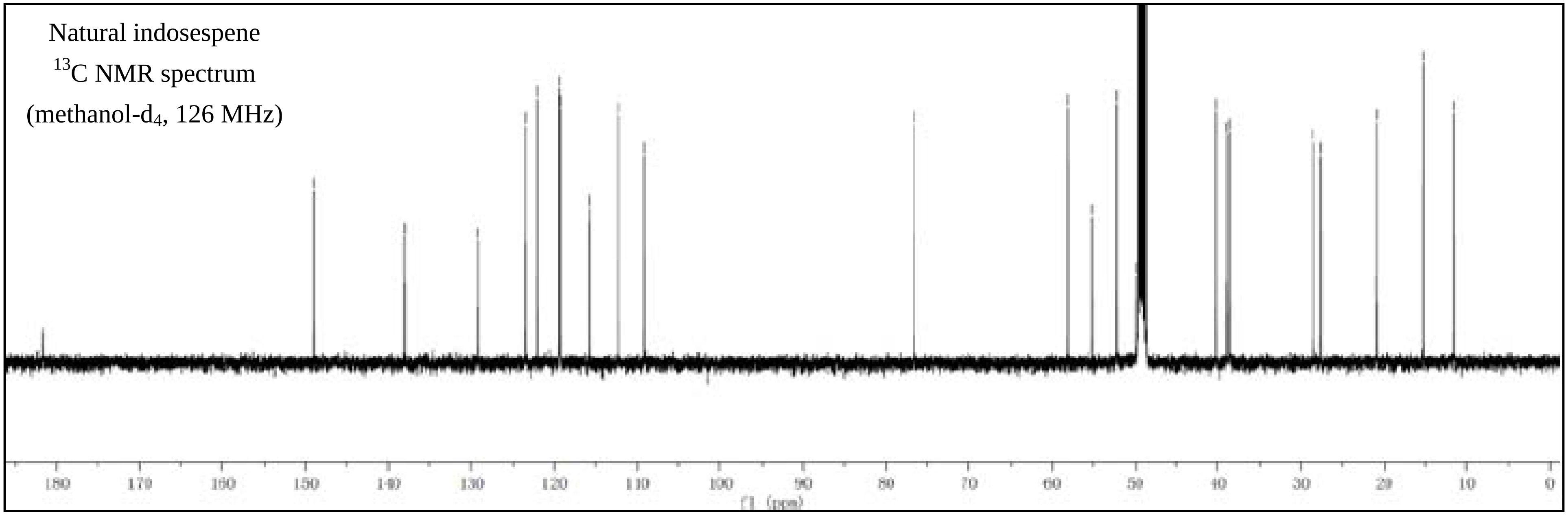


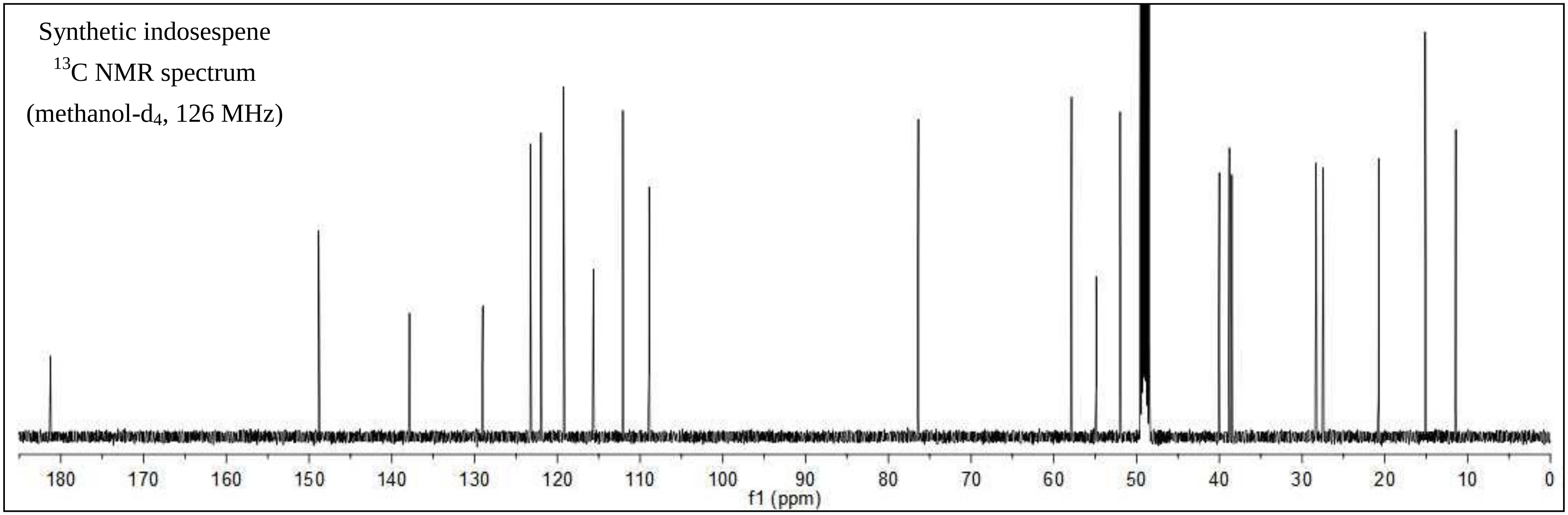

**Supplementary Figure 73. Comparison of the $^{1}$H NMR Spectra of Natural and Synthetic Xiamycin A**

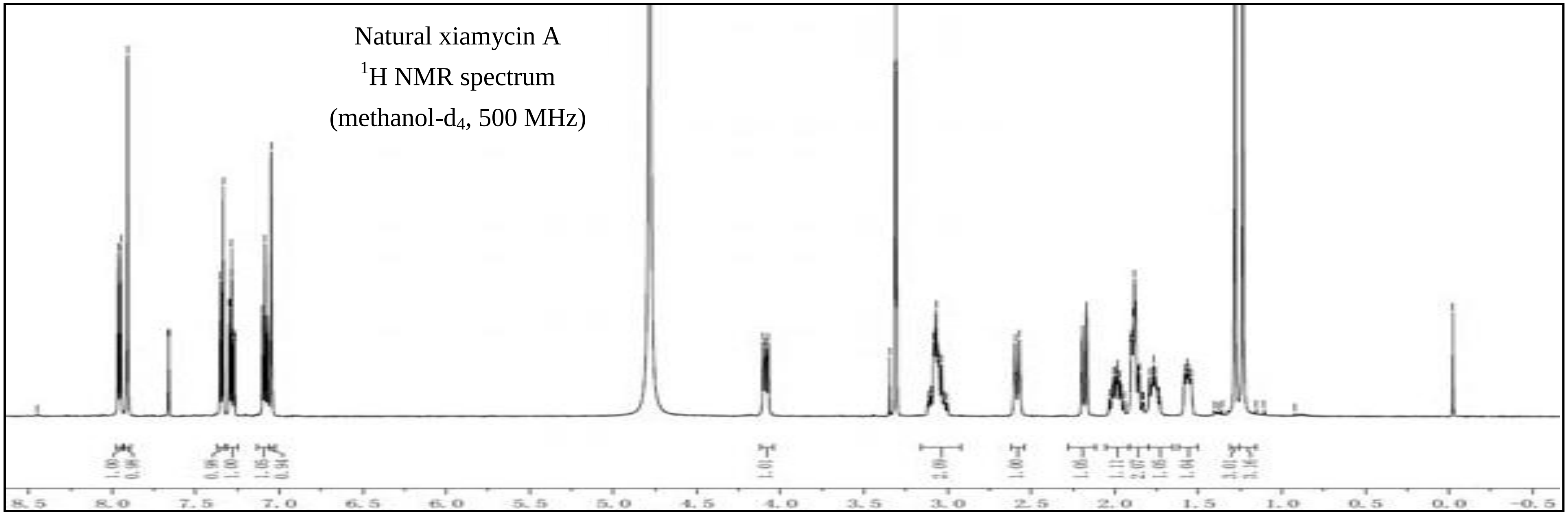


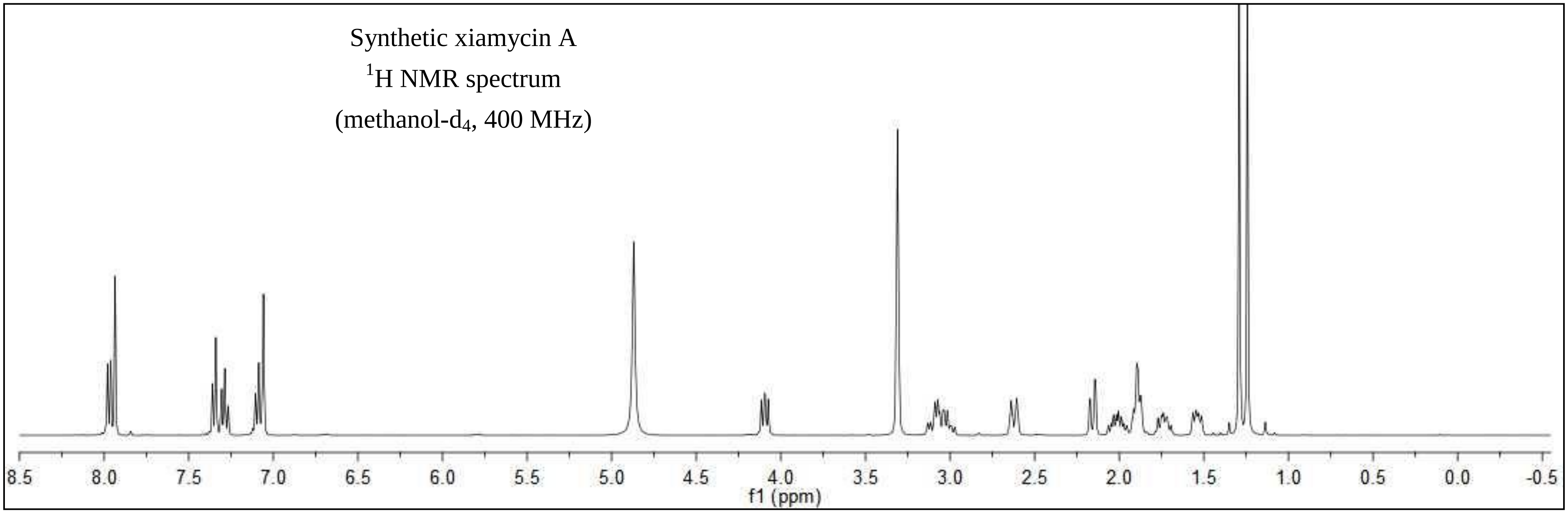

**Supplementary Figure 74. Comparison of the $^{13}C$ NMR Spectra of Natural and Synthetic Xiamycin A**

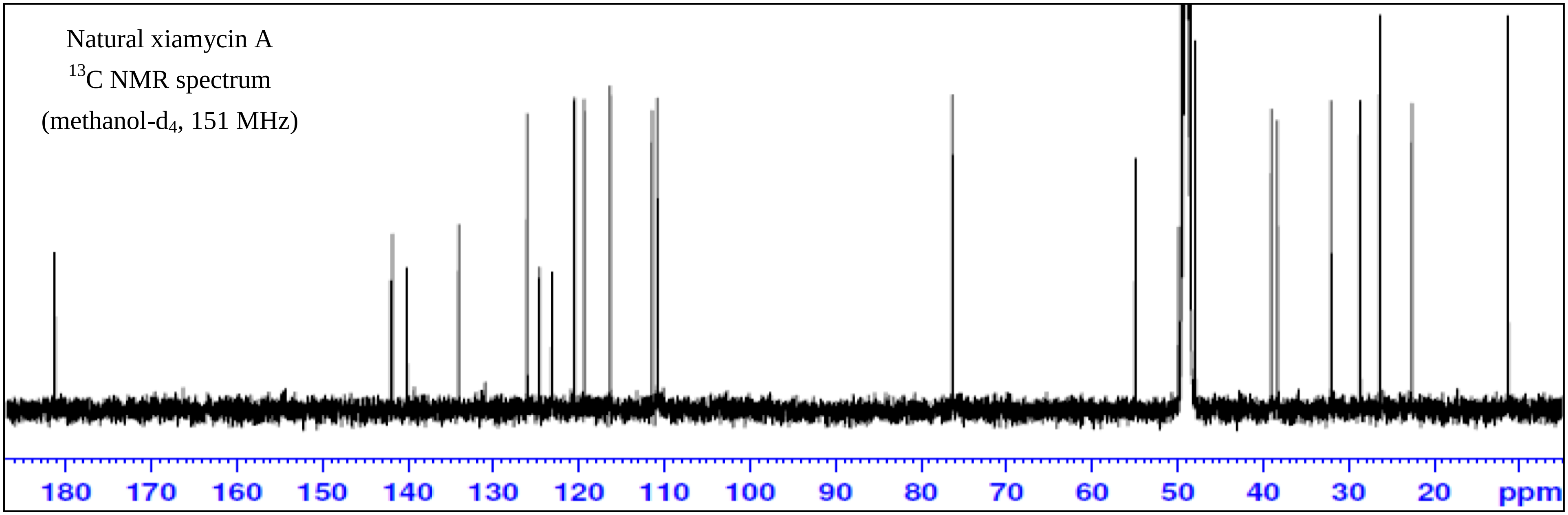


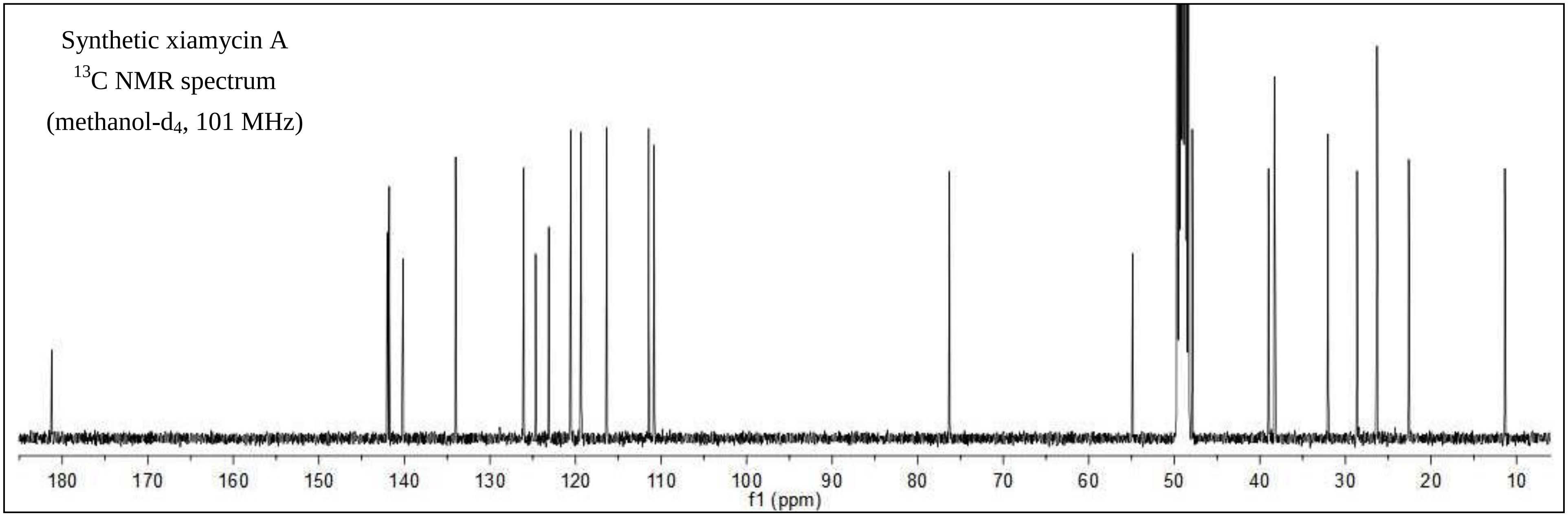

**Supplementary Figure 75. Comparison of the $^1$H NMR Spectra of Natural and Synthetic Dixiamycin C**

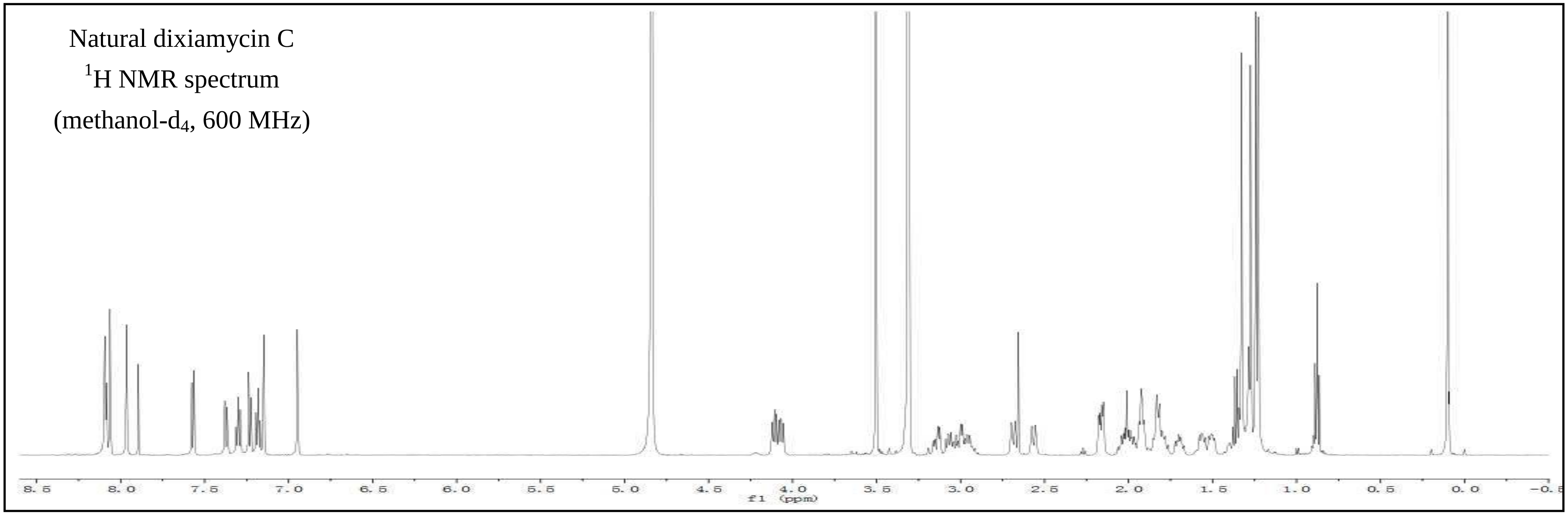


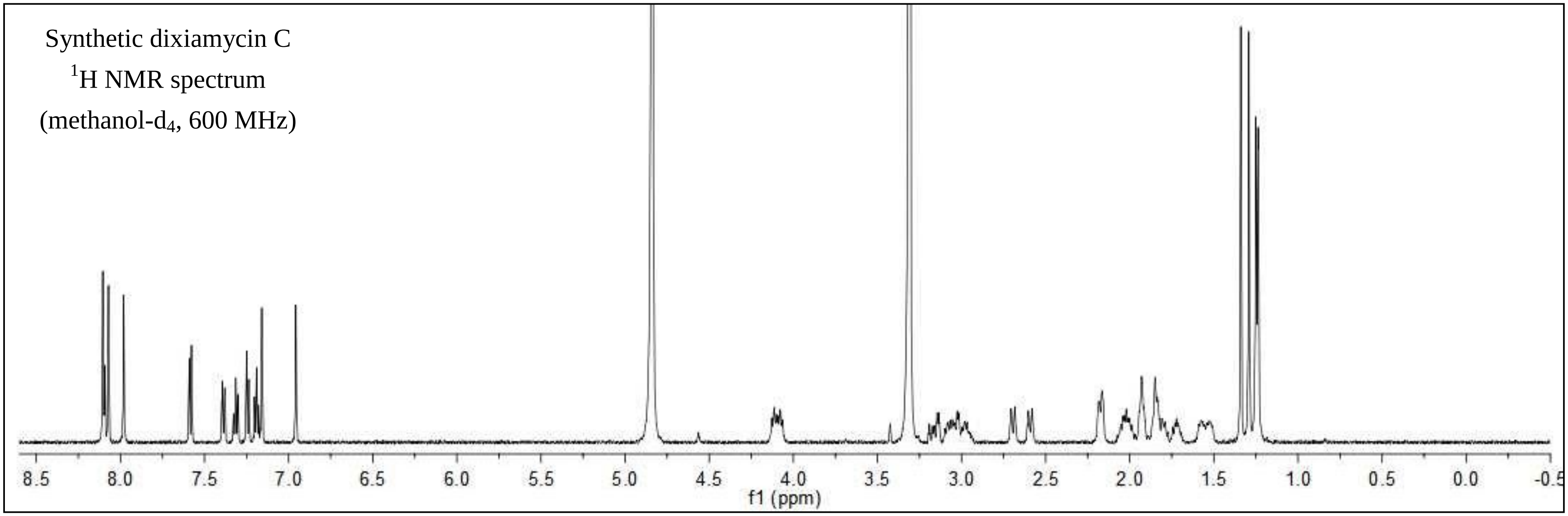

**Supplementary Figure 76. Comparison of the $^{13}$C NMR Spectra of Natural and Synthetic Dixiamycin C**

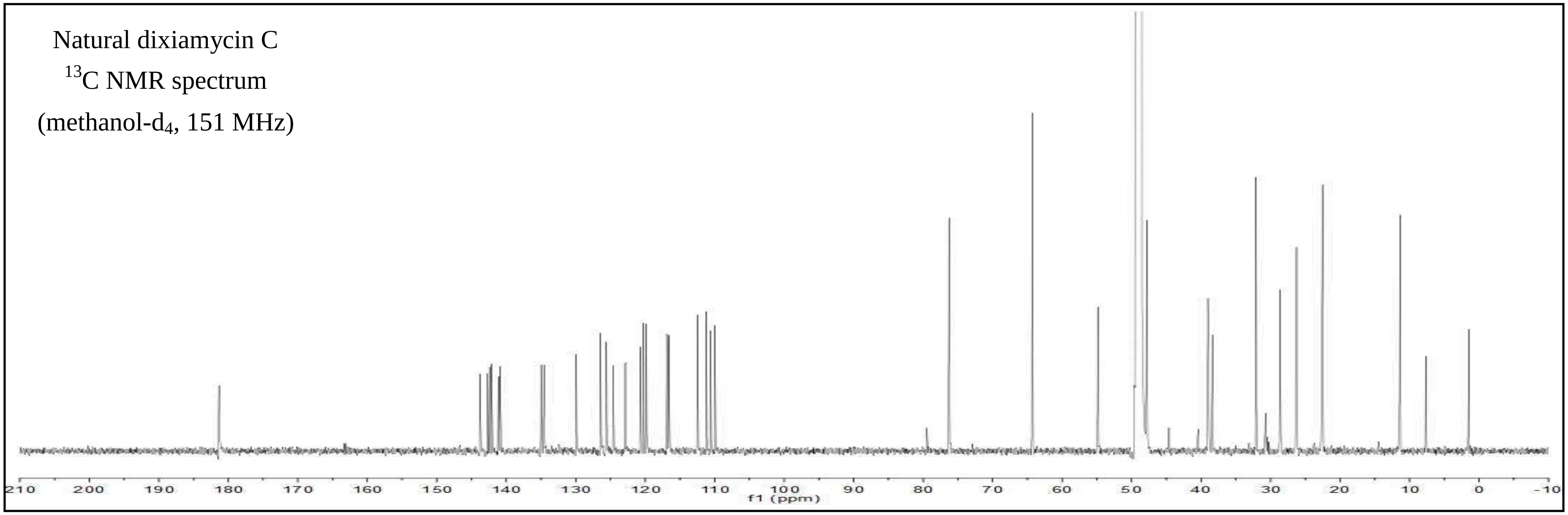


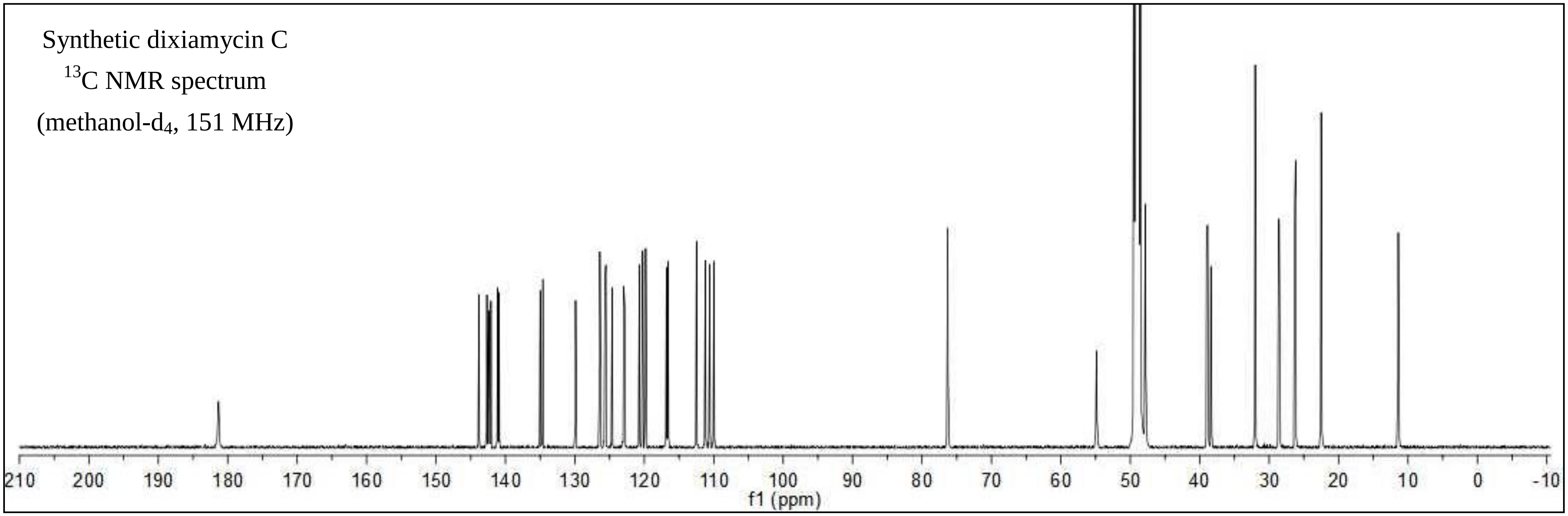

**Supplementary Figure 77. Comparison of the $^{1}$H NMR Spectra of Natural and Synthetic Oridamycin A**

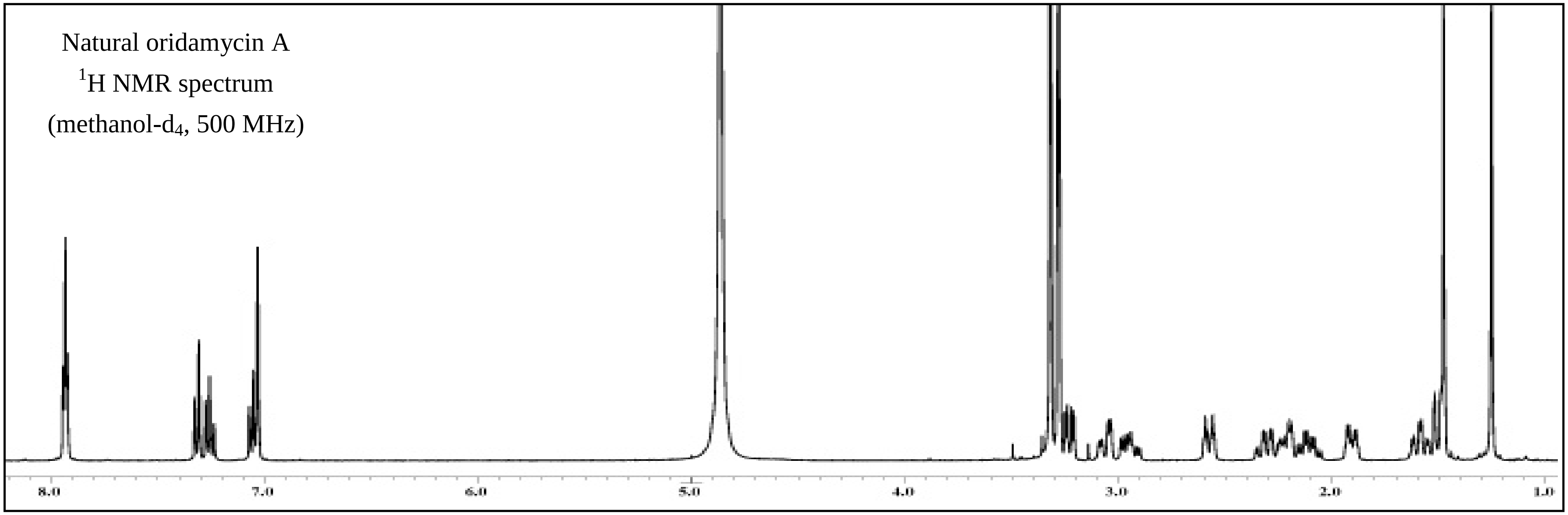


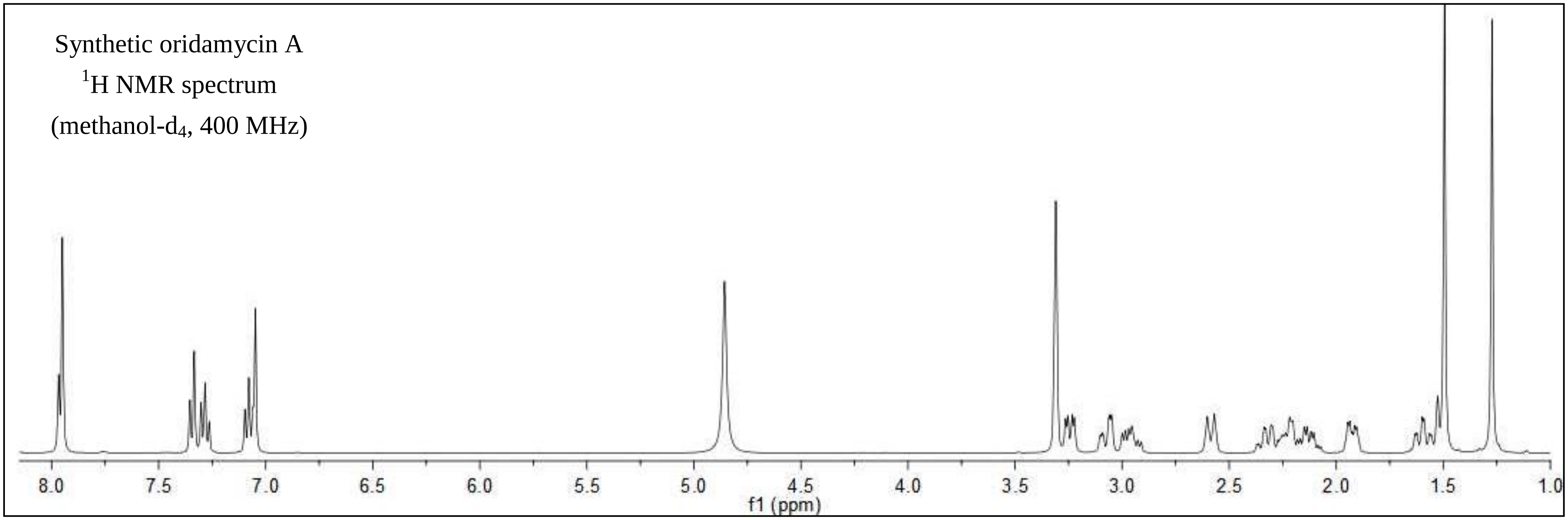

**Supplementary Figure 78. Comparison of the $^{13}$C NMR Spectra of Natural and Synthetic Oridamycin A**

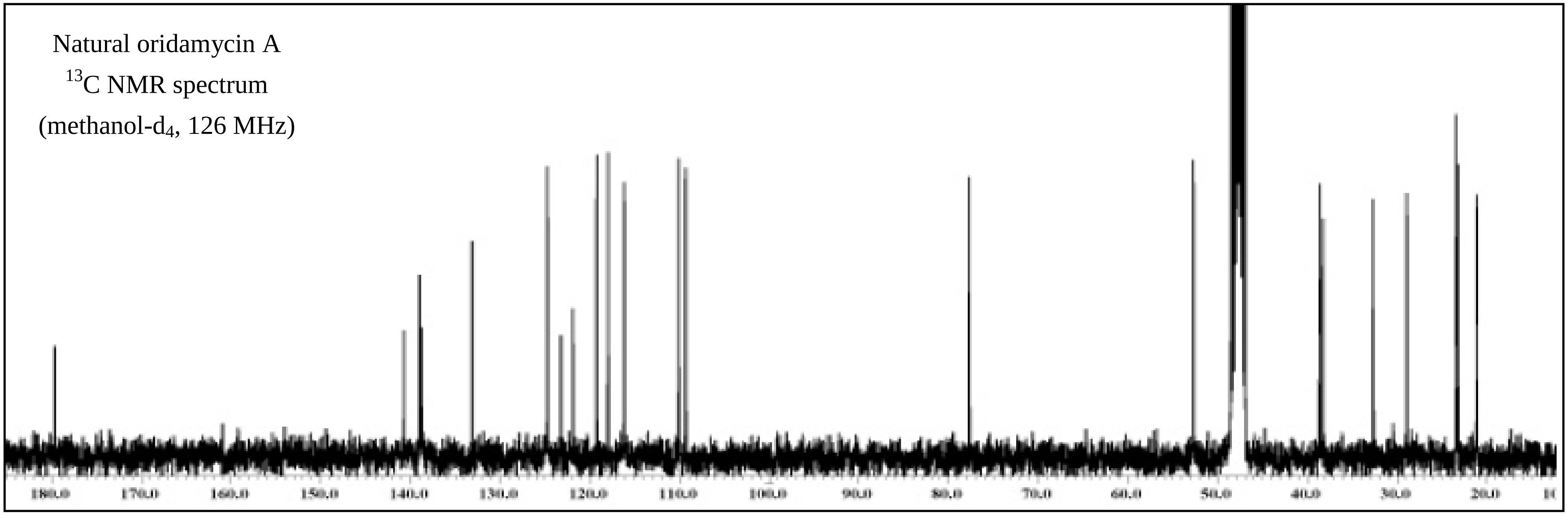


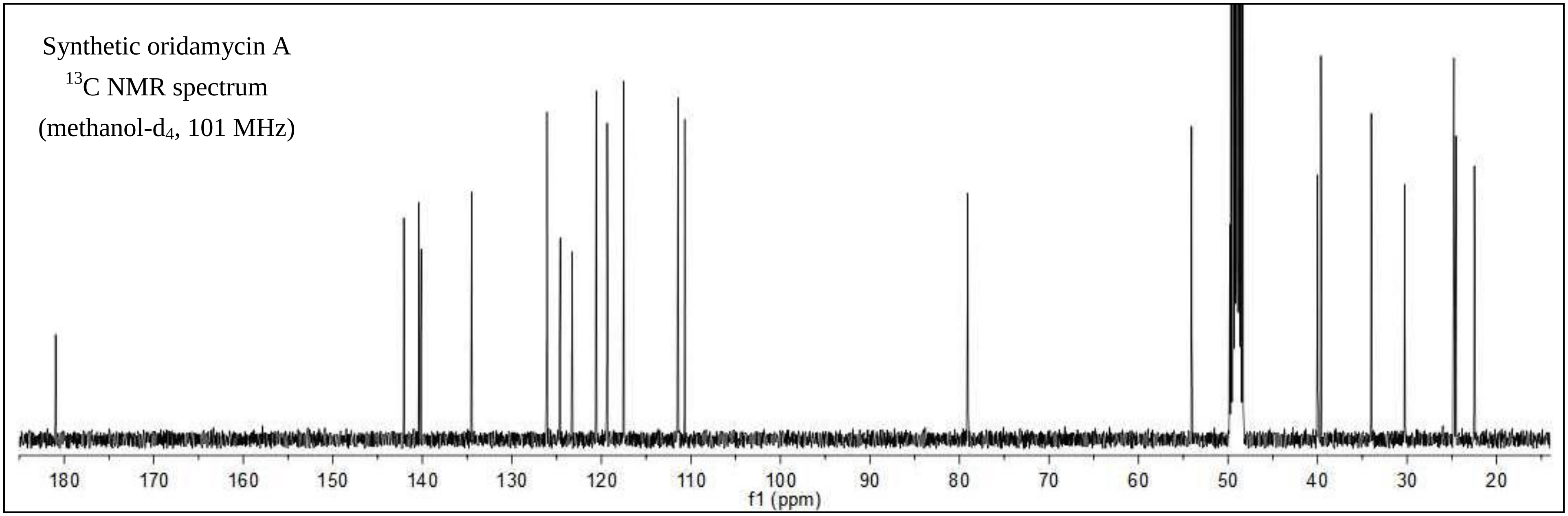

**Supplementary Figure 79. Comparison of the ¹H NMR Spectra of Natural and Synthetic Oridamycin B**

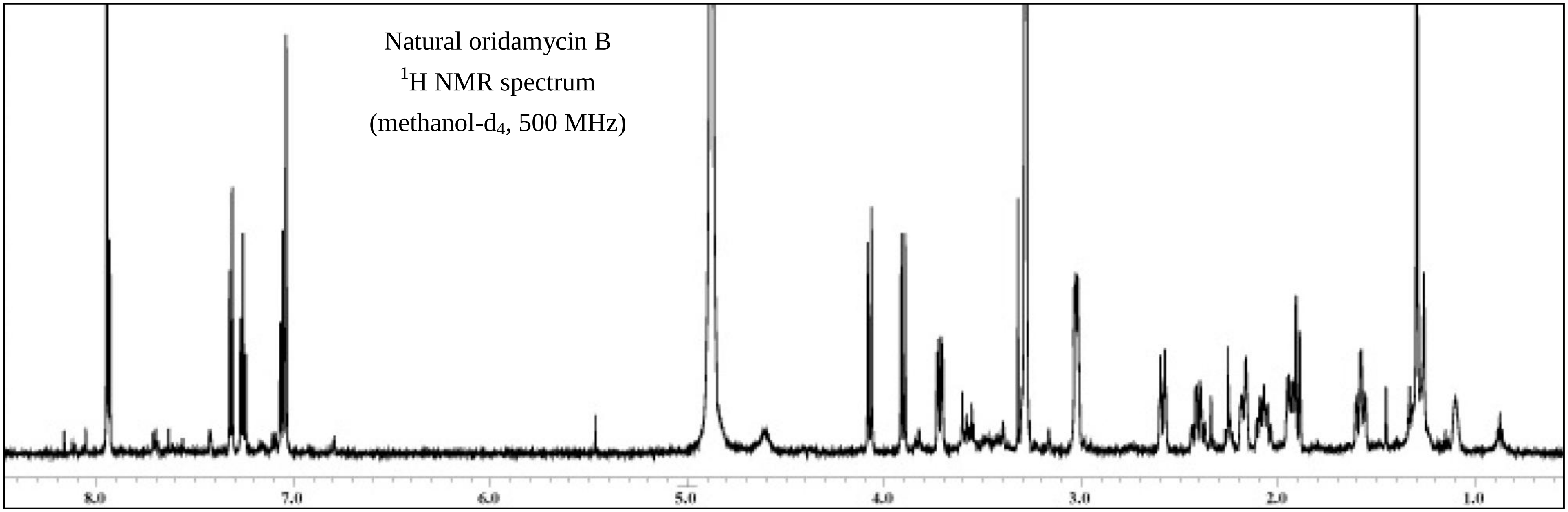


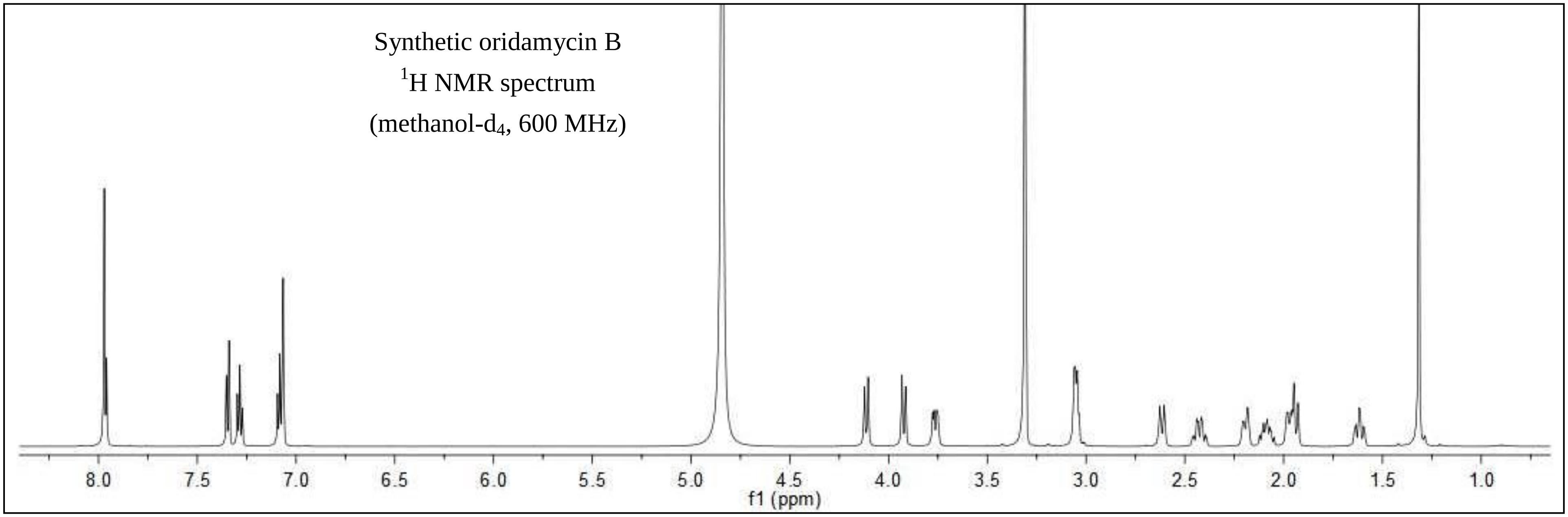

**Supplementary Figure 80. Comparison of the $^{13}$C NMR Spectra of Natural and Synthetic Oridamycin B**

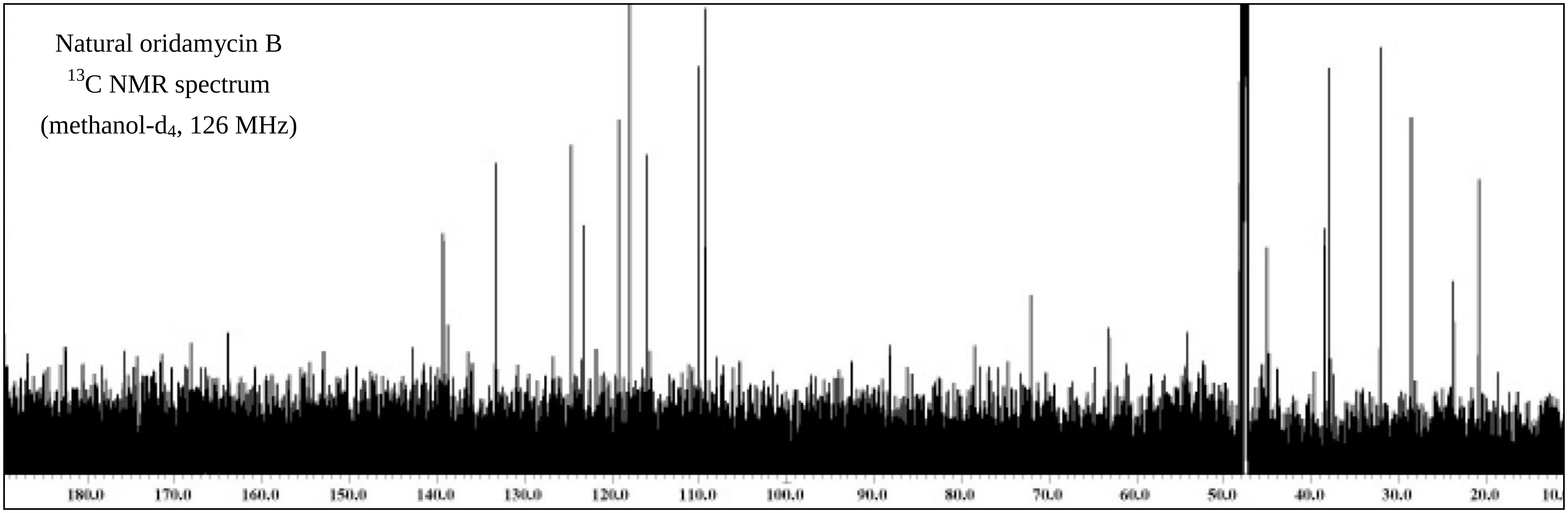


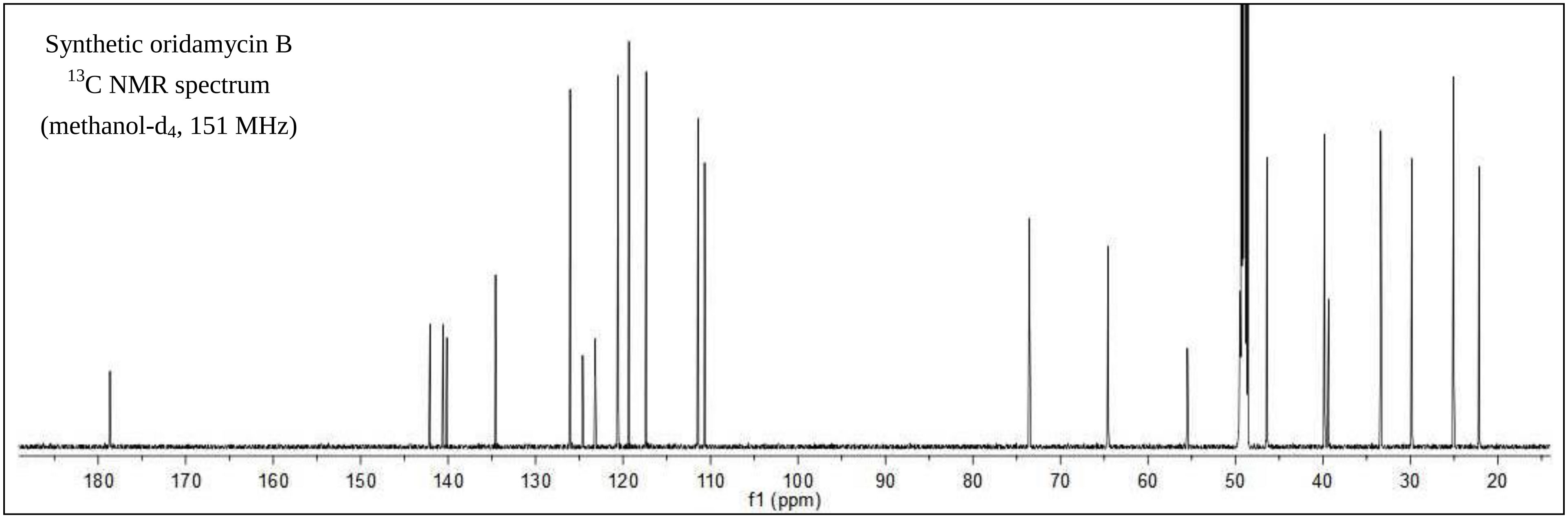

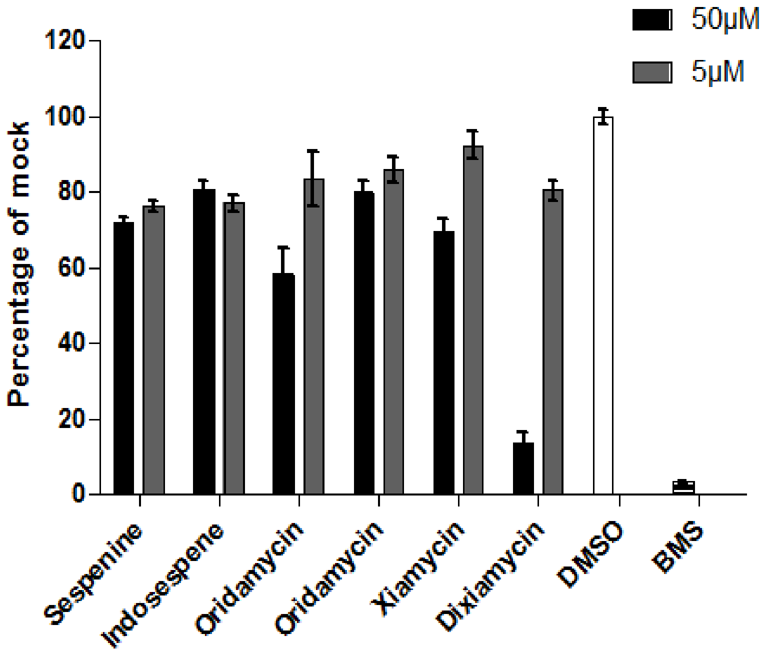


**Supplementary Figure 81. Anti-HCV activity of the compounds.** $1 \times 10^4$ JFH1-NS5A-Rluc/Huh7-Fluc cells containing a subgenomic HCV replicon were seeded into 96 well plates one day before treated with the compounds at indicated concentration for additional 72 hours. Cells were lysed, and Firefly and Renilla luciferase activities were measured by dual luciferase kits. The data was presented as average of duplicated and normalized to DMSO mock treatment. BMS: BMS790052 (Daclatasvir), a compound with potent anti-HCV activity targeting HCV NS5A protein served as positive control at a concentration of 10 nM.

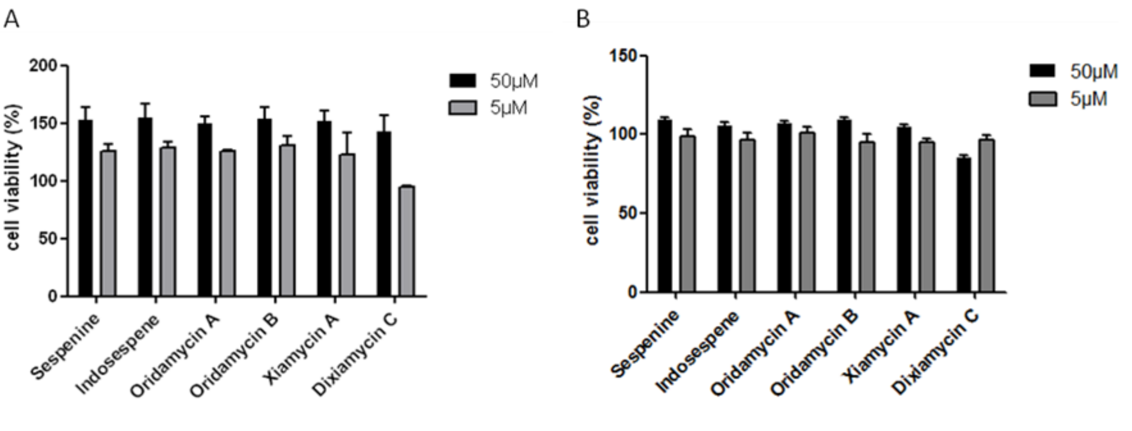


**Supplementary Figure 82. Cytotoxicity of the compounds.** (A) vero cells. (B) Huh7 cells. The cells were treated with 50 or 5.0 $\mu$M of the tested compounds or DMSO control for 24 h. Data were presented as average of triplicates and normalized to the respective DMSO-treated control cells.

**Supplementary Table 1. Comparison of the $^1$H NMR (methnanol-$d_4$) data of natural and synthetic indosespene**

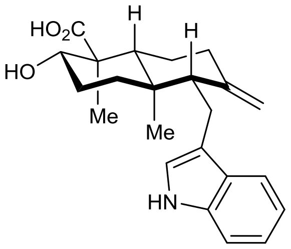


**6:** indosespene

| **Natural** $\delta_H$ [ppm, mult, *J* (Hz)] 500 MHz | | **Synthetic** $\delta_H$ [ppm, mult, *J* (Hz)] 400 MHz | | **Err** (Natural−Synthetic) $\Delta\delta_H$ (ppm) |
|---|---|---|---|---|
| 7.53 | 1 H, d, 8.0 | 7.53 | 1 H, d, 7.8 | 0 |
| 7.28 | 1 H, d, 8.0 | 7.29 | 1 H, d, 8.0 | −0.01 |
| 7.04 | 1 H, t, 7.5 | 7.05 | 1 H, t, 7.5 | −0.01 |
| 6.98 | 1 H, t, 7.3 | 6.99 | 1 H, t, 7.4 | −0.01 |
| 6.89 | 1 H, s | 6.90 | 1 H, s | −0.01 |
| 4.80 | 1 H, s | 4.80 | 1 H, s | 0 |
| 4.73 | 1 H, s | 4.73 | 1 H, s | 0 |
| 4.06 | 1 H, dd, 10.5, 5.0 | 4.06 | 1 H, dd, 11.2, 5.1 | 0 |
| 2.97 | 1 H, d, 15.0 | 2.97 | 1 H, d, 14.1 | 0 |
| 2.83 | 1 H, dd, 15.0, 10.5 | 2.83 | 1 H, dd, 15.2, 10.6 | 0 |
| 2.34 | 1 H, m | 2.37–2.32 | 1 H, m | − |
| 2.27 | 1 H, d, 10.0 | 2.28 | 1 H, d, 10.0 | −0.01 |
| 2.08 | 1 H, d, 13.5 | 2.09 | 1 H, ddd, 13.2, 3.2, 3.2 | −0.01 |
| 1.99 | 1 H, m | 2.05–1.97 | 1 H, m | − |
| 1.95 | 1 H, m | 1.95 | 1 H, dd, 12.7, 2.6 | − |
| 1.75 | 1 H, m | 1.79–1.74 | 1 H, m | − |
| 1.70 | 1 H, m | 1.74–1.68 | 1 H, m | − |
| 1.58 | 1 H, m | 1.65–1.56 | 1 H, m | − |
| 1.50 | 1 H, m | 1.55–1.45 | 1 H, m | − |
| 1.32 | 1 H, m | 1.35–1.29 | 1 H, m | − |
| 1.11 | 3 H, s | 1.11 | 3 H, s | 0 |
| 0.88 | 3 H, s | 0.88 | 3 H, s | 0 |

**Supplementary Table 2. Comparison of the $^{13}$C NMR (methnanol-$d_4$) data of natural and synthetic indosespene**

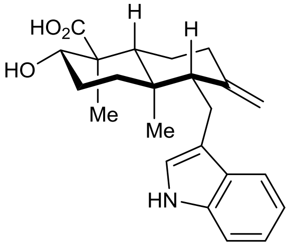

**6:** indosespene

| **Natural**<br>$\delta_C$ (ppm)<br>126 MHz | **Synthetic**<br>$\delta_C$ (ppm)<br>101 MHz | **Err**<br>(Natural−Synthetic)<br>$\Delta\delta_C$ (ppm) |
|---|---|---|
| 181.6 | 181.2 | 0.4 |
| 149.1 | 148.8 | 0.3 |
| 138.0 | 137.9 | 0.1 |
| 129.2 | 129.0 | 0.2 |
| 123.4 | 123.3 | 0.1 |
| 122.1 | 122.0 | 0.1 |
| 119.4 | 119.3 | 0.1 |
| 119.3 | 119.2 | 0.1 |
| 115.8 | 115.7 | 0.1 |
| 112.2 | 112.1 | 0.1 |
| 109.0 | 108.9 | 0.1 |
| 76.6 | 76.4 | 0.2 |
| 58.0 | 57.9 | 0.1 |
| 55.1 | 54.9 | 0.2 |
| 52.1 | 52.0 | 0.1 |
| 40.2 | 40.0 | 0.2 |
| 39.0 | 38.8 | 0.2 |
| 38.6 | 38.5 | 0.1 |
| 28.5 | 28.3 | 0.2 |
| 27.6 | 27.4 | 0.2 |
| 20.9 | 20.8 | 0.1 |
| 15.3 | 15.1 | 0.2 |

| | | |
|---|---|---|
| 11.6 | 11.4 | 0.2 |

**Supplementary Table 3. Comparison of the $^{1}$H NMR (methnanol-$d_4$) data of natural and synthetic xiamycin A**

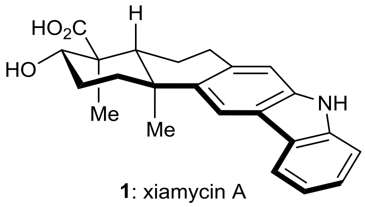


**1**: xiamycin A

| **Natural** $\delta_H$ [ppm, mult, $J$ (Hz)] 500 MHz | | **Synthetic** $\delta_H$ [ppm, mult, $J$ (Hz)] 400 MHz | | **Err** (Natural−Synthetic) $\Delta\delta_H$ (ppm) |
|---|---|---|---|---|
| 7.96 | 1 H, d, 8.0 | 7.97 | 1 H, d, 8.0 | −0.01 |
| 7.91 | 1 H, s | 7.93 | 1 H, s | −0.02 |
| 7.35 | 1 H, d, 8.0 | 7.35 | 1 H, d, 8.0 | 0 |
| 7.28 | 1 H, dt, 7.0, 1.0 | 7.29 | 1 H, t, 7.2 | −0.01 |
| 7.08 | 1 H, dt, 7.0, 1.0 | 7.09 | 1 H, d, 7.2 | −0.01 |
| 7.07 | 1 H, s | 7.06 | 1 H, s | 0.01 |
| 4.09 | 1 H, dd, 10.5, 7.5 | 4.10 | 1 H, dd, 10.4, 7.3 | −0.01 |
| 3.09 | 1 H, m | 3.15–3.01 | 1 H, m | − |
| 3.02 | 1 H, m | 3.11–2.96 | 1 H, m | − |
| 2.58 | 1 H, dt, 13.1, 1.5 | 2.62 | 1 H, dd, 13.2, 3.0 | −0.04 |
| 2.18 | 1 H, dd, 12.6, 2.3 | 2.16 | 1 H, dd, 12.4, 1.8 | 0.02 |
| 2.00 | 1 H, qd, 12.6, 7.3 | 2.08–1.95 | 1 H, m | − |
| 1.90 | 1 H, m | 1.93–1.89 | 1 H, m | − |
| 1.86 | 1 H, qd, 13.1, 2.9 | 1.89–1.85 | 1 H, m | − |
| 1.76 | 1 H, dt, 12.3, 6.7 | 1.78–1.70 | 1 H, m | − |
| 1.56 | 1 H, m | 1.54 | 1 H, dd, 13.0, 7.2 | − |
| 1.28 | 3 H, s | 1.29 | 3 H, s | −0.01 |
| 1.23 | 3 H, s | 1.24 | 3 H, s | −0.01 |

**Supplementary Table 4. Comparison of the $^{13}$C NMR (methnanol-$d_4$) data of natural and synthetic xiamycin A**

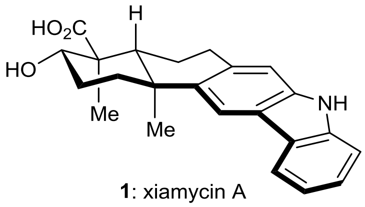


**1**: xiamycin A

| **Natural**<br>$\delta_C$ (ppm)<br>151 MHz | **Synthetic**<br>$\delta_C$ (ppm)<br>101 MHz | **Err**<br>(Natural−Synthetic)<br>$\Delta\delta_C$ (ppm) |
|---|---|---|
| 181.3 | 181.2 | 0.1 |
| 142.0 | 142.0 | 0 |
| 141.8 | 141.8 | 0 |
| 140.1 | 140.1 | 0 |
| 134.0 | 134.0 | 0 |
| 126.0 | 126.0 | 0 |
| 124.7 | 124.7 | 0 |
| 123.1 | 123.1 | 0 |
| 120.5 | 120.6 | −0.1 |
| 119.3 | 119.3 | 0 |
| 116.3 | 116.3 | 0 |
| 111.5 | 111.4 | 0.1 |
| 110.8 | 110.8 | 0 |
| 76.3 | 76.3 | 0 |
| 54.9 | 54.9 | 0 |
| 47.9 | 47.9 | 0 |
| 39.0 | 39.0 | 0 |
| 38.3 | 38.3 | 0 |
| 32.0 | 32.0 | 0 |
| 28.6 | 28.6 | 0 |
| 26.3 | 26.3 | 0 |
| 22.6 | 22.6 | 0 |
| 11.4 | 11.4 | 0 |

**Supplementary Table 5. Comparison of the $^{1}$H NMR (methnanol-d$_4$) data of natural and synthetic dixiamycin C**

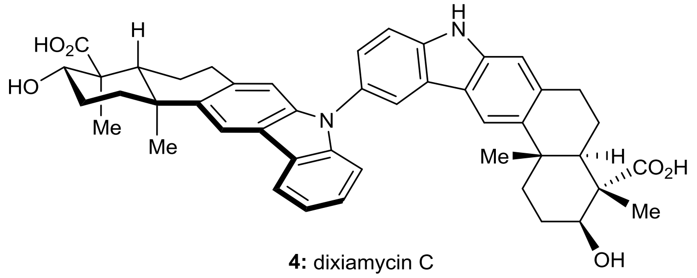


**4:** dixiamycin C

| **Natural**<br>δ$_H$ [ppm, mult, *J* (Hz)]<br>600 MHz | | **Synthetic**<br>δ$_H$ [ppm, mult, *J* (Hz)]<br>600 MHz | | **Err**<br>(Natural−Synthetic)<br>Δδ$_H$ (ppm) |
|---|---|---|---|---|
| 8.09 | 1 H, s | 8.10 | 1 H, s | −0.01 |
| 8.09 | 1 H, d, 4.0 | 8.10 | 1 H, d, 4.5 | −0.01 |
| 8.06 | 1 H, s | 8.07 | 1 H, s | −0.01 |
| 7.96 | 1 H, s | 7.98 | 1 H, s | −0.02 |
| 7.57 | 1 H, d, 8.4 | 7.58 | 1 H, d, 8.4 | −0.01 |
| 7.37 | 1 H, dd, 8.4, 1.8 | 7.39 | 1 H, dd, 8.4, 1.9 | −0.02 |
| 7.29 | 1 H, d, 7.7 | 7.31 | 1 H, t, 7.5 | −0.02 |
| 7.23 | 1 H, d, 8.1 | 7.24 | 1 H, d, 8.1 | −0.01 |
| 7.18 | 1 H, t, 7.4 | 7.19 | 1 H, t, 7.4 | −0.01 |
| 7.15 | 1 H, s | 7.16 | 1 H, s | −0.01 |
| 6.95 | 1 H, s | 6.96 | 1 H, s | −0.01 |
| 4.14–4.05 | 2 H, m | 4.13–4.06 | 2 H, m | − |
| 3.14 | 1 H, dd, 16.7, 6.3 | 3.15 | 1 H, dd, 16.8, 6.2 | −0.01 |
| 3.09–3.04 | 1 H, m | 3.08 | 1 H, dd, 17.5, 9.9 | − |
| 3.01 | 1 H, dd, 16.8, 6.3 | 3.04–3.00 | 1 H, m | − |
| 2.98–2.91 | 1 H, m | 3.00–2.93 | 1 H, m | − |
| 2.69 | 1 H, d, 13.2 | 2.69 | 1 H, d, 13.0 | 0 |
| 2.56 | 1 H, d, 13.2 | 2.59 | 1 H, d, 13.4 | −0.03 |
| 2.16 | 2 H, dd, 12.4, 3.7 | 2.17 | 2 H, dd, 12.5, 3.3 | −0.01 |
| 2.08–1.95 | 2 H, m | 2.07–1.98 | 2 H, m | − |
| 1.95–1.89 | 2 H, m | 1.94–1.91 | 2 H, m | − |
| 1.86–1.81 | 2 H, m | 1.88–1.82 | 2 H, m | − |
| 1.81–1.75 | 1 H, m | 1.82–1.75 | 1 H, m | − |

| | | | | |
|---|---|---|---|---|
| 1.73–1.66 | 1 H, m | 1.75–1.69 | 1 H, m | − |
| 1.59–1.48 | 2 H, m | 1.60–1.49 | 2 H, m | − |
| 1.33 | 3 H, s | 1.34 | 3 H, s | −0.01 |
| 1.27 | 3 H, s | 1.29 | 3 H, s | −0.02 |
| 1.24 | 3 H, s | 1.25 | 3 H, s | −0.01 |
| 1.23 | 3 H, s | 1.24 | 3 H, s | −0.01 |

**Supplementary Table 6. Comparison of the $^{13}$C NMR (methnanol-$d_4$) data of natural and synthetic dixiamycin C**

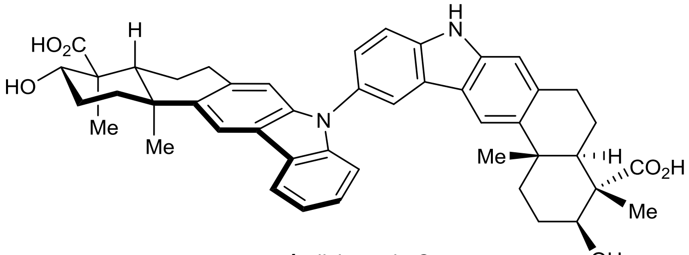


**4:** dixiamycin C

| **Natural**<br>$\delta_C$ (ppm)<br>151 MHz | **Synthetic**<br>$\delta_C$ (ppm)<br>151 MHz | **Err**<br>(Natural−Synthetic)<br>$\Delta\delta_C$ (ppm) |
|---|---|---|
| 181.3 | 181.3 | 0 |
| 143.8 | 143.8 | 0 |
| 142.7 | 142.7 | 0 |
| 142.4 | 142.4 | 0 |
| 142.1 | 142.1 | 0 |
| 141.2 | 141.1 | 0.1 |
| 140.9 | 140.9 | 0 |
| 135.0 | 135.0 | 0 |
| 134.6 | 134.6 | 0 |
| 129.9 | 129.9 | 0 |
| 126.4 | 126.4 | 0 |
| 125.7 | 125.7 | 0 |
| 125.6 | 125.5 | 0.1 |
| 124.6 | 124.6 | 0 |
| 123.0 | 123.0 | 0 |
| 122.8 | 122.8 | 0 |
| 120.7 | 120.7 | 0 |
| 120.2 | 120.2 | 0 |
| 119.8 | 119.8 | 0 |
| 116.9 | 116.8 | 0.1 |
| 116.6 | 116.6 | 0 |
| 112.5 | 112.5 | 0 |
| 111.2 | 111.2 | 0 |

| | | |
|---|---|---|
| 110.6 | 110.6 | 0 |
| 110.0 | 110.0 | 0 |
| 76.3 | 76.3 | 0 |
| 54.9 | 54.9 | 0 |
| 47.9 | 47.8 | 0.1 |
| 47.8 | 47.8 | 0 |
| 39.0 | 39.0 | 0 |
| 38.9 | 38.9 | 0 |
| 38.4 | 38.4 | 0 |
| 38.4 | 38.3 | 0.1 |
| 32.1 | 32.0 | 0.1 |
| 28.7 | 28.6 | 0.1 |
| 28.6 | 28.6 | 0 |
| 26.3 | 26.3 | 0 |
| 26.2 | 26.2 | 0 |
| 22.7 | 22.5 | 0.2 |
| 22.5 | 22.5 | 0 |
| 11.4 | 11.4 | 0 |

**Supplementary Table 7. Comparison of the $^{1}$H NMR (methnanol-$d_4$) data of natural and synthetic oridamycin A**

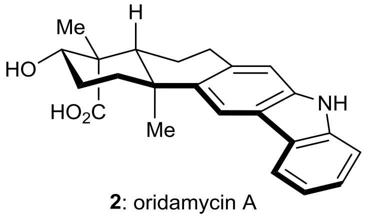


**2**: oridamycin A

| **Natural** $\delta_H$ [ppm, mult, *J* (Hz)] 600 MHz | | **Synthetic** $\delta_H$ [ppm, mult, *J* (Hz)] 400 MHz | | **Err** (Natural−Synthetic) $\Delta\delta_H$ (ppm) |
|---|---|---|---|---|
| 7.93 | 1 H, d, 8.1 | 7.96 | 1 H, d, 8.0 | −0.03 |
| 7.93 | 1 H, s | 7.95 | 1 H, s | −0.02 |
| 7.32 | 1 H, d, 8.1 | 7.34 | 1 H, d, 8.0 | −0.02 |
| 7.25 | 1 H, dt, 8.1, 1.4 | 7.28 | 1 H, t, 7.6 | −0.03 |
| 7.05 | 1 H, dt, 8.1, 1.4 | 7.08 | 1 H, t, 7.6 | −0.03 |
| 7.03 | 1 H, s | 7.05 | 1 H, s | −0.02 |
| 3.22 | 1 H, dd, 12.2, 4.6 | 3.24 | 1 H, dd, 12.1, 4.3 | −0.02 |
| 3.06 | 1 H, ddd, 16.3, 5.4, 2.3 | 3.08 | 1 H, dd, 16.5, 4.0 | −0.02 |
| 2.94 | 1 H, ddd, 16.7, 12.7, 2.3 | 3.01–2.89 | 1 H, m | − |
| 2.57 | 1 H, dt, 13.6, 3.6 | 2.59 | 1 H, dd, 13.2, 2.0 | −0.02 |
| 2.30 | 1 H, dq, 13.6, 3.6 | 2.38–2.26 | 1 H, m | − |
| 2.23 | 1 H, m | 2.25–2.19 | 1 H, m | − |
| 2.09 | 1 H, dt, 12.7, 5.4 | 2.19–2.06 | 1 H, m | − |
| 1.90 | 1 H, dq, 13.6, 3.6 | 1.97–1.89 | 1 H, m | − |
| 1.58 | 1 H, dt, 13.6, 4.1 | 1.59 | 1 H, ddd, 13.6, 13.6, 3.5 | −0.01 |
| 1.51 | 1 H, dd, 12.2, 2.3 | 1.51 | 1 H , d, 12.9 | 0 |
| 1.48 | 3 H, s | 1.49 | 3 H, s | −0.01 |
| 1.26 | 3 H, s | 1.27 | 3 H, s | −0.01 |

**Supplementary Table 8. Comparison of the $^{13}C$ NMR (methnanol-$d_4$) data of natural and synthetic oridamycin A**

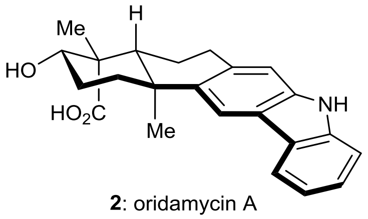


**2**: oridamycin A

| **Natural**<br>$\delta_C$ (ppm)<br>151 MHz | **Synthetic**<br>$\delta_C$ (ppm)<br>101 MHz | **Err**<br>(Natural−Synthetic)<br>$\Delta\delta_C$ (ppm) |
|---|---|---|
| 181.0 | 181.0 | 0 |
| 142.0 | 142.1 | −0.1 |
| 140.3 | 140.4 | −0.1 |
| 140.1 | 140.1 | 0 |
| 134.5 | 134.5 | 0 |
| 126.1 | 126.1 | 0 |
| 124.6 | 124.6 | 0 |
| 123.2 | 123.3 | −0.1 |
| 120.6 | 120.6 | 0 |
| 119.3 | 119.4 | −0.1 |
| 117.5 | 117.5 | 0 |
| 111.4 | 111.4 | 0 |
| 110.7 | 110.7 | 0 |
| 79.1 | 79.1 | 0 |
| 54.1 | 54.1 | 0 |
| 49.8 | 49.8 | 0 |
| 40.0 | 40.0 | 0 |
| 39.6 | 39.6 | 0 |
| 34.0 | 34.0 | 0 |
| 30.3 | 30.3 | 0 |
| 24.8 | 24.8 | 0 |
| 24.6 | 24.6 | 0 |
| 22.5 | 22.5 | 0 |

**Supplementary Table 9. Comparison of the $^{1}$H NMR (methnanol-d$_4$) data of natural and synthetic oridamycin B**

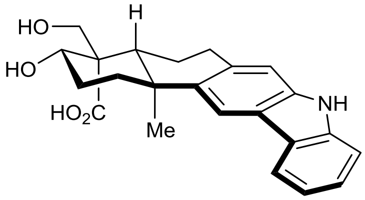


**3**: oridamycin B

| **Natural** $\delta_H$ [ppm, mult, *J* (Hz)] 600 MHz | | **Synthetic** $\delta_H$ [ppm, mult, *J* (Hz)] 400 MHz | | **Err** (Natural−Synthetic) $\Delta\delta_H$ (ppm) |
|---|---|---|---|---|
| 7.95 | 1 H, d, 7.9 | 7.96 | 1 H, d, 7.2 | −0.01 |
| 7.95 | 1 H, s | 7.97 | 1 H, s | −0.02 |
| 7.33 | 1 H, d, 7.9 | 7.35 | 1 H, d, 8.0 | −0.02 |
| 7.27 | 1 H, dt, 7.9, 1.4 | 7.28 | 1 H, t, 7.5 | −0.01 |
| 7.06 | 1 H, dt, 7.9, 1.4 | 7.08 | 1 H, d, 8.0 | −0.02 |
| 7.05 | 1 H, s | 7.07 | 1 H, s | −0.02 |
| 4.09 | 1 H, d, 11.0 | 4.11 | 1 H, d, 10.8 | −0.02 |
| 3.92 | 1 H, d, 11.0 | 3.92 | 1 H, d, 10.8 | 0 |
| 3.73 | 1 H, dd, 12.4, 4.8 | 3.76 | 1 H, dd, 12.2, 4.4 | −0.03 |
| 3.04 | 1 H, dd, 10.3, 4.1 | 3.05 | 1 H, dd, 10.3, 4.5, | −0.01 |
| 2.60 | 1 H, dt, 13.0, 3.5 | 2.62 | 1 H, d, 13.2 | −0.02 |
| 2.41 | 1 H, dq, 13.0, 4.1 | 2.47–2.39 | 1 H, m | − |
| 2.17 | 1 H, m | 2.22–2.17 | 1 H, m | − |
| 2.08 | 1 H, m | 2.13–2.04 | 1 H, m | − |
| 1.95 | 1 H, m | 1.99–1.95 | 1 H, m | − |
| 1.92 | 1 H, dd, 12.2, 2.3 | 1.94 | 1 H, d, 12.2 | −0.02 |
| 1.59 | 1 H, m | 1.61 | 1 H, ddd, 13.5, 13.5, 3.5 | − |
| 1.31 | 3 H, s | 1.32 | 3 H, s | −0.01 |

**Supplementary Table 10. Comparison of the $^{13}C$ NMR (methnanol-d$_4$) data of natural and synthetic oridamycin B**

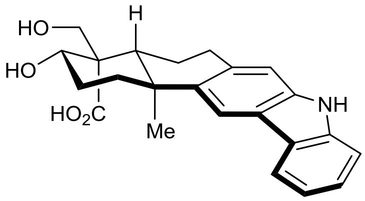


**3**: oridamycin B

| **Natural**<br>$\delta_C$ (ppm)<br>151 MHz | **Synthetic**<br>$\delta_C$ (ppm)<br>151 MHz | **Err**<br>(Natural−Synthetic)<br>$\Delta\delta_C$ (ppm) |
|---|---|---|
| 179.2 | 178.7 | 0.5 |
| 141.9 | 142.1 | −0.2 |
| 140.5 | 140.6 | −0.1 |
| 140.0 | 140.1 | −0.1 |
| 134.5 | 134.6 | −0.1 |
| 125.9 | 126.1 | −0.2 |
| 124.5 | 124.6 | −0.1 |
| 123.2 | 123.2 | 0 |
| 120.4 | 120.6 | −0.2 |
| 119.2 | 119.3 | −0.1 |
| 117.2 | 117.4 | −0.2 |
| 111.3 | 111.4 | −0.1 |
| 110.5 | 110.7 | −0.2 |
| 73.3 | 73.5 | −0.2 |
| 64.4 | 64.6 | −0.2 |
| 55.5 | 55.5 | 0 |
| 46.3 | 46.4 | −0.1 |
| 39.7 | 39.8 | −0.1 |
| 39.2 | 39.4 | −0.2 |
| 33.3 | 33.4 | −0.1 |
| 29.8 | 29.8 | 0 |
| 25.0 | 25.0 | 0 |
| 22.1 | 22.1 | 0 |

**Supplementary Methods**

**I Experimental Procedures and Spectroscopic Data of Compounds**

**II Antiviral Assay**

**III Cytotoxicity Assay**

**IV Crystallographic Data of Compounds 1, 6, and 40**

## I Experimental Procedures and Spectroscopic Data of Compounds

**General Procedures.** All reactions were carried out under an argon atmosphere with dry solvents under anhydrous conditions, unless otherwise noted. 1,4-Dioxane, toluene, and tetrahydrofuran (THF) were distilled immediately before use from sodium-benzophenone ketyl. Methylene chloride ($CH_2Cl_2$), *N*,*N*-dimethylformamide (DMF), dimethyl sulfoxide (DMSO), hexamethylphosphoramide (HMPA), triethylamine ($Et_3N$), *N*,*N*-diisopropylethylamine ($i$$Pr_2NEt$), and pyridine were distilled from calcium hydride and stored under an argon atmosphere. Methanol (MeOH) was distilled from magnesium and stored under an argon atmosphere. Acetone was dried over drierite and distilled before use. Reagents were purchased at the highest commercial quality and used without further purification, unless otherwise stated. Solvents for chromatography were used as supplied by Sinopharm Chemicals. Reactions were monitored by thin layer chromatography (TLC) carried out on S-2 0.25 mm E. Merck silica gel plates (60F-254) using UV light as visualizing agent and aqueous ammonium cerium nitrate/ammonium molybdate or basic aqueous potassium permanganate as developing agent. E. Merck silica gel (60, particle size 0.040–0.063 mm) was used for flash column chromatography. Preparative thin layer chromatography separations were carried out on 0.25 or 0.50 mm E. Merck silica gel plates (60F-254). NMR spectra were recorded on Bruker AV-400, DRX-600, or Agilent 500/54/ASP instrument and calibrated by using residual undeuterated chloroform ($\delta_H$ = 7.26 ppm) and $CDCl_3$ ($\delta_C$ = 77.16 ppm), or undeuterated methanol ($\delta_H$ = 3.31 ppm) and methanol-$d_4$ ($\delta_C$ = 49.00 ppm), as internal references. The following abbreviations are used to designate multiplicities: s = singlet, d = doublet, t = triplet, q = quartet, m = multiplet, quint = quintet, br = broad. IR spectra were recorded on a Thermo Scientific Nicolet 380 FT-IR spectrometer. Melting points (m.p.) are uncorrected and were recorded on a SGW X-4 apparatus. High-resolution mass spectra (HRMS) were recorded on a Bruker APEXIII 7.0 Tesla ESI-FT mass spectrometer at a 4000 V emitter voltage.

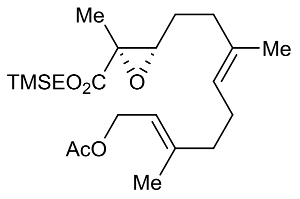

**12**

**Epoxy (trimethylsilyl)ethyl ester 12:** To a stirred solution of epoxy alcohol **11**[1] (10.8 g, 36.4 mmol) in $CH_2Cl_2$ (50 mL) were sequentially added $NaHCO_3$ (7.36 g, 87.6 mmol) and Dess–Martin periodione (18.6 g, 43.8 mmol) at 22 ℃. The reaction mixture was stirred at that temperature for 30 min before it was quenched with saturated aq. $NaHCO_3$ (200 mL). The resulting mixture was extracted with EtOAc (3 × 150 mL), and the combined organic phases were dried over anhydrous $MgSO_4$. After filtration and evaporation of the solvent under vacuum, the residue was purified by flash column chromatography with EtOAc/petroleum ether (1:10) to give the desired aldehyde as a pale yellow oil. This oil was dissolved in *t*BuOH (126 mL). To the resulting solution were sequentially added 2-methyl-2-butene (21.9 g, 33.0 mL, 314 mmol) and an aqueous solution (63.0 mL) of $NaClO_2$ (8.48 g, 94.2 mmol) and $NaH_2PO_4$ (18.8 g, 157 mmol) at 0 ℃. The reaction mixture was stirred at that temperature for 10 min before it was quenched with saturated aq. $NaHSO_3$ (24 mL). After extraction with EtOAc (3 × 200 mL), the combined organic phases were dried over anhydrous $Na_2SO_4$ and filtered. The solvent was evaporated under vacuum, and the residue so obtained was dissolved in $CH_2Cl_2$ (28 mL). To this solution were sequentially added TMSEOH (2.34 g, 2.80 mL, 37.7 mmol), 4-DMAP (3.83 g, 31.4 mmol), and EDC•HCl (6.62 g, 34.5 mmol) at 22 ℃. The reaction mixture was stirred at that temperature for 5 h before it was quenched with water (50 mL). The resulting mixture was extracted with EtOAc (3 × 100 mL), and the combined organic phases were washed with brine (10 mL) and dried over anhydrous $Na_2SO_4$. After filtration and evaporation of the solvent, the residue was subjected to flash column chromatography for purification using EtOAc/petroleum ether (1:15) as eluent to give epoxy (trimethylsilyl)ethyl ester **12** (11.1 g, 74 % for the three steps) as a colorless oil. This compound can also be prepared through a two step sequence as below. To a stirred solution of epoxy alcohol **11** (132 mg, 0.446 mmol) in $CH_2Cl_2$ (1.7 mL) were sequentially added pH 7 phosphate buffer (1.7 mL),

AZADO[2] (3.4 mg, 0.022 mmol), and $PhI(OAc)_2$ (431 mg, 1.34 mmol) at 22 °C. The reaction mixture was stirred at that temperature for 6 h before it was quenched with saturated aq. $NaHSO_3$ (5 mL) and extracted with EtOAc (3 × 10 mL). The combined organic phases were washed with brine (5 mL), dried over anhydrous $Na_2SO_4$, and filtered. The solvent was evaporated under vacuum. The residue so obtained was dissolved in $CH_2Cl_2$ (0.80 mL). To this solution were sequentially added TMSEOH (32.2 mg, 40.0 μL, 0.535 mmol), 4-DMAP (54.4 mg, 0.446 mmol), and EDC•HCl (94.2 mg, 0.491 mmol) at 22 °C. The resultant mixture was stirred at that temperature for 6 h before it was quenched with water (5 mL) and extracted with EtOAc (3 × 10 mL). The combined organic phases were washed with brine (5 mL) and dried over anhydrous $Na_2SO_4$. After filtration and evaporation of the solvent, the residue was purified by flash column chromatography with EtOAc/petroleum ether (1:15) to give **12** (139 mg, 76 % for the two steps) as a colorless oil. **12**: $R_f$ = 0.32 (silica, EtOAc:petroleum ether 1:10); $[\alpha]_D^{26}$ = −8.4 (*c* = 1.1 in $CHCl_3$); IR (film): $\nu_{max}$ = 3386, 2954, 2853, 1730, 1456, 1250, 1288, 1250, 1166, 1095, 858 $cm^{-1}$; $^1H$ NMR (400 MHz, $CDCl_3$): δ = 5.27 (td, *J* = 7.1, 0.9 Hz, 1 H), 5.09 (t, *J* = 6.4 Hz, 1 H), 4.51 (d, *J* = 7.1 Hz, 2 H), 4.20–4.09 (m, 2 H), 3.08 (dd, *J* = 6.2, 6.1 Hz, 1 H), 2.16–2.10 (m, 1 H), 2.09–2.02 (m, 3 H), 2.00–1.96 (m, 2 H), 1.98 (s, 3 H), 1.63 (s, 3 H), 1.63–1.58 (m, 2 H), 1.55 (s, 3 H), 1.44 (s, 3 H), 0.98–0.93 (m, 2 H), −0.02 (s, 9 H) ppm; $^{13}C$ NMR (101 MHz, $CDCl_3$): δ = 171.58, 170.94, 141.85, 133.89, 124.71, 118.44, 63.85, 61.88, 61.26, 57.52, 39.31, 35.96, 26.58, 26.11, 20.99, 17.28, 16.41, 15.95, 13.54, −1.56 ppm; HRMS (*m/z*): $[M + Na]^+$ calcd for $C_{22}H_{38}O_5SiNa^+$ 433.2381, found 433.2388.

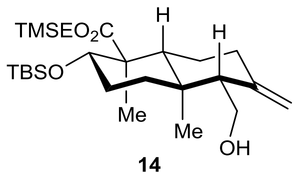

**14**

***trans*-Decalin alcohol 14:** A suspension of $Cp_2TiCl_2$ (14.6 mg, 0.0586 mmol) and manganese dust (129 mg, 2.35 mmol) in THF (7.3 mL) was stirred at 22 °C for 15 min. To the resulting mixture were sequentially added epoxy (trimethylsilyl)ethyl ester **12** (122 mg, 0.297 mmol), *i*$Pr_2$NEt (230 mg, 310 μL, 1.76 mmol), and TMSCl (151 mg, 180 μL, 1.46 mmol). The reaction mixture was stirred at that temperature for 4 h before it was quenched with aq. HCl (3 mL, 2.0 M) and extracted with EtOAc (3 ×

15 mL). The combined organic phases were washed with brine (5 mL) and dried over anhydrous $Mg_2SO_4$. After filtration and evaporation of the volatile under vacuum, the residue was subjected to flash column chromatography using EtOAc/petroleum ether (1:2) as eluent to give *trans*-decalin **13** as a white powder. This powder was dissolved in DMF (0.50 mL). To this solution were sequentially added imidazole (15.6 mg, 0.229 mmol) and TBSCl (26.5 mg, 0.176 mmol) at 22 ℃. The resulting mixture was stirred at that temperature for 10 h before it was quenched with saturated aq. $NaHCO_3$ (3 mL). After extraction with EtOAc (3 × 5 mL), the combined organic phases were washed with brine (2 mL), dried over anhydrous $MgSO_4$, and filtered. The solvent was evaporated under vacuum, and the residue was purified by flash column chromatography with EtOAc/petroleum ether (1:9) as eluent to give the corresponding silyl ether as a pale yellow oil. This oil was dissolved in MeOH (1.0 mL). To this solution was added anhydrous $K_2CO_3$ (20.3 mg, 0.147 mmol) at 22 ℃, and the reaction mixture was stirred at that temperature for 3 h before it was quenched with saturated aq. $NaHCO_3$ (2 mL). The resulting mixture was extracted with EtOAc (3 × 5 mL). The combined organic phases were washed with brine (2 mL) and dried over anhydrous $MgSO_4$. After filtration and evaporation of the solvent under vacuum, the residue was subjected to flash column chromatography for purification using EtOAc/petroleum ether (1:10 → 1:4) as eluent to give *trans*-decalin alcohol **14** (63.5 mg, 44 % for the three steps) as a colorless oil. **14**: $R_f$ = 0.34 (silica, EtOAc:petroleum ether 1:5); $[\alpha]_D^{26}$ = +13.7 (*c* = 0.55 in $CHCl_3$); IR (film): $\nu_{max}$ = 2923, 2868, 2847, 1462, 1388, 1196, 1145, 1082, 936, 602 $cm^{-1}$; $^1H$ NMR (500 MHz, $CDCl_3$): δ = 4.94 (s, 1 H), 4.65 (s, 1 H), 4.19–4.02 (m, 3 H), 3.84–3.76 (m, 2 H), 2.41–2.32 (m, 1 H), 2.05–1.97 (m, 2 H), 1.85 (dd, *J* = 12.7, 2.6 Hz, 1 H), 1.73–1.66 (m, 1 H), 1.64–1.55 (m, 2 H), 1.52 (dd, *J* = 13.1, 4.2 Hz, 1 H), 1.49–1.41 (m, 1 H), 1.35 (dd, *J* = 7.8, 4.0 Hz, 1 H), 1.22–1.17 (m, 1 H), 1.07 (s, 3 H), 0.99–0.94 (m, 2 H), 0.82 (s, 9 H), 0.72 (s, 3 H), 0.05 (s, 9 H), 0.02 (s, 3 H), −0.04 (s, 3 H) ppm; $^{13}C$ NMR (126 MHz, $CDCl_3$): δ = 177.64, 146.95, 107.29, 76.10, 63.02, 58.91, 58.88, 54.45, 50.58, 38.07, 37.42, 36.67, 27.80, 26.17, 25.79, 18.02, 17.37, 15.71, 11.30, −1.31, −3.78, −5.10 ppm; HRMS (*m/z*): $[M + Na]^+$ calcd for $C_{26}H_{50}O_4SiNa^+$ 505.3140, found 505.3138.

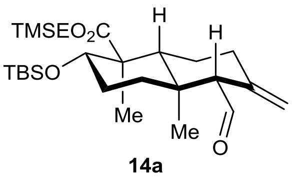


**14a**

***trans*-Decalin aldehyde 14a:** To a stirred solution of *trans*-decalin alcohol **14** (1.21 g, 2.51 mmol) in $CH_2Cl_2$ (6.0 mL) was added Dess−Martin periodinane (1.60 g, 3.77 mmol) at 22 °C. After stirring at that temperature for 30 min, the reaction mixture was quenched with saturated aq. $NaHCO_3$ (50 mL) and extracted with EtOAc (3 × 50 mL). The combined organic phases were dried over anhydrous $MgSO_4$ and filtered. The solvent was evaporated under vacuum, and the residue was purified by flash column chromatography with EtOAc/petroleum ether (1:20) to give *trans*-decalin aldehyde **14a** (1.13 g, 94 %) as a colorless oil. **14a**: $R_f$ = 0.41 (silica, EtOAc:petroleum ether 1:15); $[\alpha]_D^{28}$ = −7.3 (*c* = 0.55 in $CHCl_3$); IR (film): $\nu_{max}$ = 2956, 2855, 1720, 1480, 1259, 1219, 1105, 1033, 837, 772 $cm^{-1}$; $^1H$ NMR (400 MHz, $CDCl_3$): δ = 9.87 (d, *J* = 4.2 Hz, 1 H), 4.94 (s, 1 H), 4.54 (s, 1 H), 4.19–4.05 (m, 2 H), 4.04 (dd, *J* = 10.3, 5.6 Hz, 1 H), 2.48 (d, *J* = 3.6 Hz, 1 H), 2.37 (ddd, *J* = 13.6, 4.3, 2.0 Hz, 1 H), 2.05 (ddd, *J* = 13.6, 12.9, 4.9 Hz, 1 H), 1.75 (dd, *J* = 12.6, 2.5 Hz, 1 H), 1.71–1.66 (m, 1 H), 1.66–1.58 (m, 3 H), 1.27–1.24 (m, 1 H), 1.44–1.35 (m, 1 H), 1.23–1.17 (m, 1 H), 1.13 (s, 6 H), 1.00–0.94 (m, 2 H), 0.82 (s, 9 H), 0.06 (d, *J* = 3.1 Hz, 9 H), 0.02 (s, 3 H), −0.04 (s, 3 H) ppm; $^{13}C$ NMR (101 MHz, $CDCl_3$): δ = 204.41, 177.32, 144.05, 110.23, 75.98, 67.35, 63.14, 54.32, 49.62, 38.13, 36.87, 36.25, 27.35, 25.75, 25.04, 17.98, 17.35, 16.33, 11.40, −1.34, −3.83, −5.18 ppm; HRMS (*m/z*): $[M + Na]^+$ calcd for $C_{26}H_{48}O_4Si_2Na^+$ 503.2983; found 503.2985.

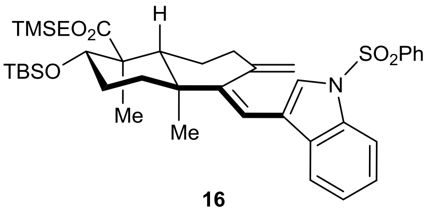


**16**

**Triene 16:** To a stirred solution of *trans*-decalin aldehyde **14a** (421 mg, 0.876 mmol) in THF (8.0 mL) was added a solution of Grignard reagent **15**[3] (6.60 mL, ca. 0.4 M in THF, ca. 2.64 mmol) at 22 °C. The reaction mixture was stirred at that temperature for 40 min before it was quenched with saturated aq. $NaHCO_3$ (15 mL). After extraction with EtOAc (3 × 20 mL), the combined organic phases were washed

with brine (5 mL), dried over anhydrous $Na_2SO_4$, and filtered. The solvent was evaporated under vacuum, and the residue so obtained was purified by flash column chromatography with EtOAc/petroleum ether (1:3) to give the desired secondary alcohol as a white powder. This powder was dissolved in $CH_2Cl_2$ (10 mL). To this solution were sequentially added *i*$Pr_2NEt$ (170 mg, 230 $\mu$L, 1.32 mmol) and MsCl (178 mg, 120 $\mu$L, 1.05 mmol) at 0 ℃. The reaction mixture was stirred at that temperature for 1 h before it was quenched with saturated aq. $NaHCO_3$ (10 mL). The resulting mixture was extracted with EtOAc (3 × 30 mL), and the combined organic phases were dried over anhydrous $Na_2SO_4$. After filtration and removal of the solvent under vacuum, the residue was subjected to flash column chromatography for purification using EtOAc/petroleum ether (1:15) as eluent to give triene **16** (503 mg, 80 % for the two steps) as a white powder. **16**: $R_f$ = 0.34 (silica, EtOAc:petroleum ether 1:15); $[\alpha]_D^{26}$ = +9.4 ($c$ = 1.0 in $CHCl_3$); IR (film): $\nu_{max}$ = 2952, 2928, 2895, 1727, 1447, 1373, 1249, 1176, 976, 837 $cm^{-1}$; $^1H$ NMR (400 MHz, $CDCl_3$): δ = 7.97 (d, $J$ = 8.2 Hz, 1 H), 7.80 (d, $J$ = 7.6 Hz, 2 H), 7.59 (s, 1 H), 7.50 (t, $J$ = 7.4 Hz, 1 H), 7.48 (d, $J$ = 7.6 Hz, 1 H), 7.39 (t, $J$ = 7.8 Hz, 2 H), 7.29 (dd, $J$ = 7.7, 7.3 Hz, 1 H), 7.22 (t, $J$ = 7.4 Hz, 1 H), 6.07 (s, 1 H), 4.87 (s, 1 H), 4.58 (s, 1 H), 4.21–4.05 (m, 3 H), 2.49 (dd, $J$ = 13.0, 2.4 Hz, 1 H), 2.23 (ddd, $J$ = 12.9, 12.8, 5.3 Hz, 1 H), 1.97 (dd, $J$ = 12.6, 2.6 Hz, 1 H), 1.88–1.83 (m, 1 H), 1.81–1.75 (m, 3 H), 1.75–1.67 (m, 1 H), 1.33–1.25 (m, 1 H), 1.17 (s, 3 H), 1.05 (s, 3 H), 1.03–0.96 (m, 2 H), 0.85 (s, 9 H), 0.06 (s, 12 H), −0.03 (s, 3 H) ppm; $^{13}C$ NMR (126 MHz, $CDCl_3$): δ = 177.49, 155.51, 145.62, 138.33, 134.94, 133.74, 131.51, 129.24, 126.82, 124.76, 123.26, 123.22, 120.13, 119.66, 113.86, 113.42, 107.14, 76.29, 63.10, 54.79, 49.16, 40.94, 36.67, 35.08, 28.02, 25.81, 25.61, 20.72, 18.05, 17.44, 11.24, −1.32, −3.77, −5.09 ppm; HRMS ($m/z$): $[M + Na]^+$ calcd for $C_{40}H_{57}N_1O_5SSi_2Na^+$ 742.3388, found 742.3392.

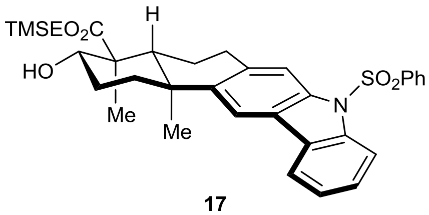


**17**

***N*-Benzenesulfonyl xiamycin A (trimethylsilyl)ethyl ester 17:** Triene **16** (15.4 mg, 0.0214 mmol) was dissolved in DMSO (0.50 mL) in a tube under an air atmosphere. After bubbling air through the solution

for 10 min, the tube was sealed and heated at 120 ℃ under microwave irradiation for 8 × 15 min. The resulting mixture was cooled to 22 ℃ and diluted with water (2 mL). After extraction with EtOAc (5 × 3 mL), the combined organic phases were washed with brine (2 mL), dried over anhydrous $Na_2SO_4$, and filtered. The solvent was removed under vacuum. The residue so obtained was purified by flash column chromatography with EtOAc/petroleum ether (1:6) to give *N*-benzenesulfonylxiamycin A (trimethylsilyl)ethyl ester **17** (10.3 mg, 80 %) as a white powder. **17**: $R_f$ = 0.35 (silica, EtOAc:petroleum ether 1:3); $[\alpha]_D^{27}$ = +79.5 (*c* = 1.05 in $CHCl_3$); IR (film): $\nu_{max}$ = 2950, 2925, 1716, 1465, 1370, 1248, 1090, 1039, 995, 770 $cm^{-1}$; $^1H$ NMR (400 MHz, $CDCl_3$): δ = 8.26 (d, *J* = 8.3 Hz, 1 H), 7.97 (s, 1 H), 7.84 (d, *J* = 7.7 Hz, 1 H), 7.81 (d, *J* = 8.1 Hz, 2 H), 7.75 (s, 1 H), 7.48–7.41 (m, 2 H), 7.37–7.29 (m, 3 H), 4.31–4.19 (m, 2 H), 4.04 (dd, *J* = 10.8, 3.4 Hz, 1 H), 3.21–3.06 (m, 2 H), 2.48 (ddd, *J* = 12.9, 3.1, 2.8 Hz, 1 H), 2.19 (dd, *J* = 12.4, 2.0 Hz, 1 H), 2.05–1.76 (m, 3 H), 1.73 (dd, *J* = 13.1, 3.5 Hz, 1 H), 1.62–1.54 (m, 1 H), 1.29 (s, 3 H), 1.26 (s, 3 H), 1.04 (t, *J* = 10.0 Hz, 2 H), 0.06 (s, 9 H) ppm; $^{13}C$ NMR (126 MHz, $CDCl_3$): δ = 177.48, 145.52, 138.59, 138.21, 136.84, 135.33, 133.80, 129.18, 127.05, 126.78, 126.61, 124.68, 123.94, 119.72, 115.66, 115.14, 114.82, 75.26, 63.61, 53.56, 45.49, 37.40, 37.15, 31.18, 27.32, 25.72, 21.45, 17.69, 10.90, −1.36 ppm; HRMS (*m/z*): $[M + Na]^+$ calcd for $C_{34}H_{41}N_1O_5SSiNa^+$ 626.2367, found 626.2363.

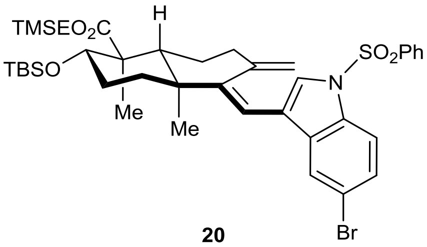


**20**

**Bromotriene 20:** This compound was synthesized from *trans*-decalin aldehyde **14a** (647 mg, 1.35 mmol) and Grignard reagent **18**[3] (10.1 mL, ca. 0.4 M in THF, ca. 4.04 mmol) by using the procedure for the preparation of triene **16**. Bromotriene **20** (812 mg, 76 %) was obtained as a white powder. **20**: $R_f$ = 0.37 (silica, EtOAc:petroleum ether 1:15); $[\alpha]_D^{26}$ = +25.9 (*c* = 0.85 in $CHCl_3$); IR (film): $\nu_{max}$ = 2952, 2855, 1726, 1445, 1375, 1218, 1146, 1122, 973, 773 $cm^{-1}$; $^1H$ NMR (500 MHz, $CDCl_3$): δ = 7.84 (d, *J* = 8.8 Hz, 1 H), 7.77 (d, *J* = 8.3 Hz, 2 H), 7.58 (d, *J* = 1.7 Hz, 1 H), 7.56 (s, 1 H), 7.51 (t, *J* = 7.2 Hz, 1 H),

7.41 (t, $J$ = 7.7 Hz, 2 H), 7.38 (d, $J$ = 8.8 Hz, 1 H), 5.98 (s, 1 H), 4.86 (s, 1 H), 4.53 (s, 1 H), 4.20–4.05 (m, 3 H), 2.49 (dd, $J$ = 13.1, 2.7 Hz, 1 H), 2.21 (ddd, $J$ = 12.9, 12.9, 5.1 Hz, 1 H), 1.96 (dd, $J$ = 12.7, 2.3 Hz, 1 H), 1.88–1.82 (m, 1 H), 1.79–1.74 (m, 3 H), 1.74–1.68 (m, 1 H), 1.31–1.29 (m, 1 H), 1.17 (s, 3 H), 1.03 (s, 3 H), 1.03–0.97 (m, 2 H), 0.85 (s, 9 H), 0.05 (s, 12 H), −0.02 (s, 3 H) ppm; $^{13}$C NMR (126 MHz, $CDCl_3$): δ = 177.42, 156.20, 145.51, 138.00, 134.02, 133.64, 133.22, 129.38, 127.62, 126.78, 124.36, 122.57, 119.60, 116.95, 115.31, 113.55, 106.53, 76.23, 63.11, 54.76, 49.10, 40.98, 36.63, 35.06, 27.95, 25.81, 25.55, 20.67, 18.03, 17.41, 11.24, −1.31, −3.76, −5.10 ppm; HRMS (*m/z*): $[M + Na]^+$ calcd for $C_{40}H_{56}BrN_1O_5SSi_2Na^+$ 820.2493, found 820.2506.

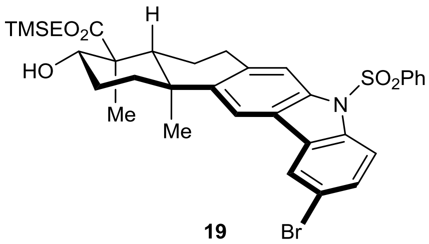


**19**

***N*-Benzenesulfonyl 6-bromoxiamycin A (trimethylsilyl)ethyl ester 19:** This compound was synthesized from bromotriene **20** (8.3 mg, 0.010 mmol) by using the procedure for the preparation of *N*-benzenesulfonylxiamycin A (trimethylsilyl)ethyl ester **17**. *N*-Benzenesulfonyl 6-bromoxiamycin A (trimethylsilyl)ethyl ester **19** (5.4 mg, 76 %) was obtained as a white powder. **19**: $R_f$ = 0.40 (silica, EtOAc:petroleum ether 1:3); $[\alpha]_D^{27}$ = +82.4 ($c$ = 0.85 in $CHCl_3$); IR (film): $\nu_{max}$ = 2927, 2875, 1715, 1462, 1447, 1219, 1174, 1090, 808, 772 cm$^{-1}$; $^{1}$H NMR (400 MHz, $CDCl_3$): δ = 8.13 (d, $J$ = 8.8 Hz, 1 H), 7.97 (s, 1 H), 7.95 (s, 1 H), 7.78 (d, $J$ = 7.9 Hz, 2 H), 7.70 (s, 1 H), 7.52 (d, $J$ = 8.9 Hz, 1 H), 7.49 (t, $J$ = 7.3 Hz, 1 H), 7.35 (t, $J$ = 7.7 Hz, 2 H), 4.31–4.18 (m, 2 H), 4.04 (dd, $J$ = 11.4, 4.3 Hz, 1 H), 3.20–3.05 (m, 2 H), 2.46 (d, $J$ = 12.9 Hz, 1 H), 2.17 (d, $J$ = 12.1 Hz, 1 H), 2.04–1.89 (m, 2 H), 1.84–1.76 (m, 1 H), 1.60–1.53 (m, 1 H), 1.57 (dd, $J$ = 12.8, 7.1 Hz, 1 H), 1.28 (s, 3 H), 1.25 (s, 3 H), 1.04 (t, $J$ = 8.6 Hz, 2 H), 0.06 (s, 9 H) ppm; $^{13}$C NMR (126 MHz, $CDCl_3$): δ = 177.43, 145.93, 137.92, 137.35, 137.15, 136.32, 134.04, 129.72, 129.30, 128.71, 126.60, 123.52, 122.73, 117.33, 116.59, 115.91, 114.91, 75.23, 63.64, 53.53, 45.40, 37.42, 37.14, 31.18, 27.28, 25.71, 21.38, 17.70, 10.89, −1.35 ppm; HRMS (*m/z*): $[M + Na]^+$ calcd for $C_{34}H_{40}BrN_1O_5SSiNa^+$ 704.1472, found 704.1476.

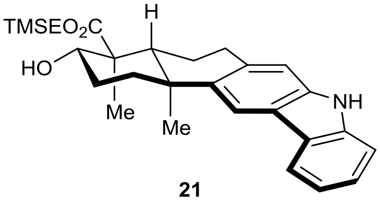


**21**

**Xiamycin A (trimethylsilyl)ethyl ester 21:** To a solution of **17** (165 mg, 0.273 mmol) in MeOH (3.0 mL) was added magnesium powder (13.3 mg, 0.554 mmol) at 22 ℃. The resulting suspension was sonicated at that temperature for 1 h before it was quenched with saturated aq. $NH_4Cl$ (5 mL). The resultant mixture was extracted with EtOAc (3 × 10 mL). The combined organic phases were washed with brine (5 mL) and dried over anhydrous $MgSO_4$. After filtration and evaporation of the solvent under vacuum, the residue was purified by flash column chromatography with EtOAc/petroleum ether (1:5) to give xiamycin A (trimethylsilyl)ethyl ester **21** (124 mg, 98 %) as a white foam. **21**: $R_f$ = 0.29 (silica, EtOAc:petroleum ether 1:4); $[\alpha]_D^{25}$ = +116.5 (*c* = 0.60 in $CHCl_3$); IR (film): $\nu_{max}$ = 3410, 2950, 1708, 1465, 1245, 1067, 1036, 858, 837, 768 $cm^{-1}$; $^1H$ NMR (400 MHz, $CDCl_3$): δ = 8.01 (d, *J* = 7.8 Hz, 1 H), 7.94 (s, 1 H), 7.86 (s, 1 H), 7.39–7.34 (m, 2 H), 7.22–7.17 (m, 1 H), 7.07 (s, 1 H), 4.32–4.15 (m, 2 H), 4.12–4.05 (m, 1 H), 3.17–3.06 (m, 2 H), 2.60–2.56 (m, 1 H), 2.25 (dd, *J* = 12.4, 2.1 Hz, 1 H), 2.06–1.95 (m, 2 H), 1.93–1.85 (m, 1 H), 1.84–1.76 (m, 2 H), 1.58–1.51 (m, 1 H), 1.31 (s, 3 H), 1.30 (s, 3 H), 1.04–0.99 (m, 2 H), 0.05 (s, 9 H) ppm; $^{13}C$ NMR (126 MHz, $CDCl_3$): δ = 177.58, 141.28, 140.15, 138.30, 133.56, 125.56, 123.71, 122.13, 120.03, 119.30, 115.81, 110.59, 109.97, 75.38, 63.47, 53.67, 45.84, 37.54, 37.42, 31.05, 27.53, 26.04, 21.64, 17.68, 10.93, −1.37 ppm; HRMS (*m/z*): $[M + Na]^+$ calcd for $C_{28}H_{37}N_1O_3SiNa^+$ 486.2435, found 486.2441.

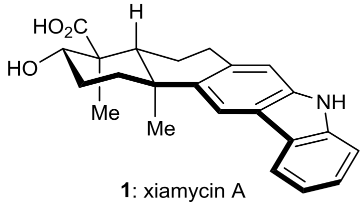


**1**: xiamycin A

**Xiamycin A (1):** To a stirred solution of xiamycin A (trimethylsilyl)ethyl ester **21** (11.8 mg, 0.0254 mmol) in DMF (0.20 mL) was added TASF (14.0 mg, 0.0508 mmol) at 22 ℃. The resulting mixture was warmed to 50 ℃ and stirred at that temperature for 5 h before it was cooled to 22 ℃ and quenched

with aq. HCl (2 mL, 1.0 M). The mixture so obtained was extracted with EtOAc (5 × 3 mL). The combined organic phases were washed with brine (2 mL), dried over anhydrous $Na_2SO_4$, and filtered. The solvent was evaporated under vacuum, and the residue was subjected to HPLC (Waters 2535Q, SunFire Prep C18 OBD column, 19 mm × 250 mm) using acetonitrile/water (40:60 → 80:20, 10 mL/min) as eluent to give xiamycin A (**1**, $t_R$ = 19.88 min). After removal of the solvent under vacuum, **1** (8.8 mg, 95 %) was obtained as a white powder. **1**: $R_f$ = 0.24 (silica, MeOH/$CH_2Cl_2$ 1:15); m.p. 259–260 ℃ (acetone); $[\alpha]_D^{23}$ = +137.6 (*c* = 0.4 in MeOH); IR (film): $\nu_{max}$ = 3705, 2980, 2980, 2966, 2922, 2359, 1694, 1454, 1055, 1032 $cm^{-1}$; $^1H$ NMR (400 MHz, methanol-$d_4$): δ = 7.97 (d, *J* = 8.0 Hz, 1 H), 7.93 (s, 1 H), 7.35 (d, *J* = 8.0 Hz, 1 H), 7.29 (t, *J* = 7.2 Hz, 1 H), 7.09 (d, *J* = 7.2 Hz, 1 H), 7.06 (s, 1 H), 4.10 (dd, *J* = 10.4, 7.3 Hz, 1 H), 3.15–3.01 (m, 1 H), 3.11–2.96 (m, 1 H), 2.62 (dd, *J* = 13.2, 3.0 Hz, 1 H), 2.16 (dd, *J* = 12.4, 1.8 Hz, 1 H), 2.08–1.95 (m, 1 H), 1.93–1.89 (m, 1 H), 1.89–1.85 (m, 1 H), 1.78–1.70 (m, 1 H), 1.54 (dd, *J* = 13.0, 7.2 Hz, 1 H), 1.29 (s, 3 H), 1.24 (s, 3 H) ppm; $^{13}C$ NMR (101 MHz, methanol-$d_4$): δ = 181.24, 142.02, 141.77, 140.14, 134.02, 126.04, 124.65, 123.12, 120.55, 119.34, 116.34, 111.44, 110.80, 76.28, 54.87, 47.90, 39.00, 38.31, 32.03, 28.64, 26.30, 22.59, 11.36 ppm; HRMS (*m/z*): $[M + Na]^+$ calcd for $C_{23}H_{25}N_1O_3Na^+$ 386.1727, found 386.1720. CCDC 948203 contains the supplementary crystallographic data for **1** and is available free of charge from The Cambridge Crystallographic Data Centre via www.ccdc.cam.ac.uk/data_request/cif.

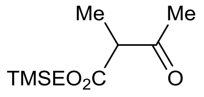


**39**

**(Trimethylsilyl)ethyl 2-methyl-3-oxobutanoate 39:** To a stirred suspension of $K_2CO_3$ (70.2 g, 0.508 mol) in neat (trimethylsilyl)ethyl oxobutanoate[4] (51.4 g, 0.254 mol) was added MeI (63.8 g, 28.0 mL, 0.449 mmol) in two portions at 0 °C. After stirring at that temperature for 12 h, the resulting mixture was diluted with water (500 mL) and extracted with EtOAc (3 × 500 mL). The combined organic phases were washed with brine (2 × 500 mL), dried over anhydrous $Na_2SO_4$, and filtered. The solvent was evaporated under vacuum. The residue so obtained was purified by flash column chromatography with EtOAc/petroleum ether (1:15) to give (trimethylsilyl)ethyl 2-methyl-3-oxobutanoate **39** (50.9 g, 93 %)

as a pale yellow oil. **39**: $R_f$ = 0.31 (silica, EtOAc:petroleum ether 1:15); IR (film): $\nu_{max}$ = 2956, 2900, 1743, 1716, 1456, 1359, 1251, 1154, 1044, 935, 860, 838, 763, 695 cm$^{-1}$; $^{1}$H NMR (400 MHz, $CDCl_3$): δ = 4.26–4.18 (m, 2 H), 3.47 (q, *J* = 7.2 Hz, 1 H), 2.23 (s, 3 H), 1.33 (d, *J* = 7.1 Hz, 3 H), 1.03–0.96 (m, 2 H), 0.04 (s, 9 H) ppm; $^{13}$C NMR (101 MHz, $CDCl_3$): δ = 203.77, 170.75, 63.80, 53.84, 28.53, 17.35, 12.80, −1.47 ppm; HRMS (*m/z*): $[M + Na]^+$ calcd for $C_{10}H_{20}O_3Na^+$ 239.1074, found 239.1077.

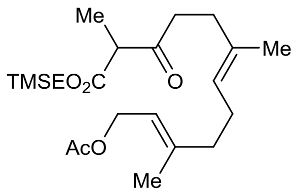


**25**

***β*-Ketoester 25:** This compound was prepared by using a protocol similar to that developed by Yamamoto et al.[5] To a stirred solution of 8-hydroxygeraniol acetate[6] (542 mg, 2.55 mmol) in THF (12 mL) were sequentially added LiBr (1.11g, 12.8 mmol), $Et_3N$ (387 mg, 530 μL, 3.83 mmol), and MsCl (351 mg, 240 μL, 3.06 mmol) at 0 ℃. The resulting mixture was stirred at that temperature for 30 min before it was quenched with saturated aq. $NaHCO_3$ (20 mL) and extracted with diethyl ether (3 × 30 mL). The combined organic phases were sequentially washed with saturated aq. $NaHCO_3$ (10 ml) and saturated aq. NaBr (10 mL) and dried over anhydrous $MgSO_4$. After filtration and evaporation of the solvent under vacuum, the crude bromide was immediately dissolved in THF (5.0 mL) and used for the next step. To a stirred suspension of KH (197 mg, 4.93 mmol) in HMPA/THF (12 mL, 1:5) was added a solution of (trimethylsilyl)ethyl 2-methyl-3-oxobutanoate **39** (827 mg, 3.83 mmol) in THF (10 mL) at 0 ℃. The resulting mixture was stirred at that temperature for 30 min before BuLi (1.90 mL, 2.4 M in hexane, 4.56 mmol) was added. The resultant mixture was stirred at 0 ℃ for 30 min, and the solution of the freshly prepared bromide was added. The reaction mixture was stirred at that temperature for 3 h before it was quenched with saturated aq. $NH_4Cl$ (20 mL) and extracted with EtOAc (3 × 30 mL). The combined organic phases were washed with saturated aq. $NH_4Cl$ (10 mL), dried over anhydrous $Na_2SO_4$, filtered, and concentrated under vacuum. The residue so obtained was dissolved in $CH_2Cl_2$ (5.0 mL). To this solution were sequentially added pyridine (39.3 mg, 40.0 μL, 0.497 mmol), $Ac_2O$ (54.1 mg, 50.0 μL,

0.530 mmol), and 4-DMAP (67.6 mg, 0.553 mmol) at −10 ℃. The reaction mixture was stirred at that temperature for 1 h before it was quenched with saturated aq. $NaHCO_3$ (15 mL). The resulting mixture was extracted with EtOAc (3 × 20 mL), and the combined organic phases were washed with brine (5 mL) and dried over anhydrous $MgSO_4$. After filtration and removal of the solvent under vacuum, the residue was subjected to flash column chromatography for purification using EtOAc/petroleum ether (1:10 → 1:5) as eluent to give *β*-ketoester **25** (607 mg, 58 % for the three steps) as a pale yellow oil. **25**: $R_f$ = 0.28 (silica, EtOAc:petroleum ether 1:10); IR (film): $\nu_{max}$ = 2953, 1738, 1602, 1453, 1380, 1232, 1111, 950, 859, 764, 695 $cm^{-1}$; $^1$H NMR (400 MHz, $CDCl_3$): δ = 5.32 (t, *J* = 7.1 Hz, 1 H), 5.09 (t, *J* = 6.0 Hz, 1 H), 4.57 (d, *J* = 7.1 Hz, 2 H), 4.23–4.17 (m, 2 H), 3.49 (d, *J* = 7.2 Hz, 1 H), 2.70–2.52 (m, 2 H), 2.24 (t, *J* = 7.7 Hz, 2 H), 2.12–2.06 (m, 2 H), 2.04 (s, 3 H), 2.04–1.99 (m, 2 H), 1.68 (s, 3 H), 1.31 (d, *J* = 7.2 Hz, 3 H), 1.01–0.95 (m, 2 H), 0.03 (s, 9 H) ppm; $^{13}$C NMR (101 MHz, $CDCl_3$): δ = 205.62, 171.18, 170.80, 142.07, 133.97, 124.51, 118.49, 63.78, 61.45, 53.09, 40.23, 39.44, 33.32, 26.27, 21.15, 17.41, 16.57, 16.17, 12.88, −1.43 ppm; HRMS (*m/z*): $[M + Na]^+$ calcd for $C_{22}H_{38}O_5SiNa^+$ 433.2381, found 433.2384.

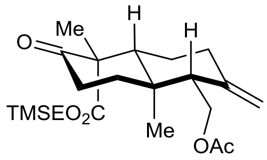


**26**

***trans*-Decalin 26:** To a stirred suspension of $Mn(OAc)_3•2H_2O$ (127 mg, 0.473 mmol) and $Cu(OAc)_2•H_2O$ (47.5 mg, 0.238 mmol) in DMSO (2.0 mL) was added a solution of *β*-ketoester **25** (97.1 mg, 0.237 mmol) in DMSO (0.40 mL) at 22 ℃. The resulting mixture was stirred at that temperature for 12 h before it was diluted with water (5 mL) and extracted with EtOAc (3 × 10 mL). The combined organic phases were washed with brine (2 × 5 mL), dried over anhydrous $Na_2SO_4$, and filtered. The solvent was evaporated under vacuum, and the residue was purified by flash column chromatography with EtOAc/petroleum ether (1:10 → 1:5) to give *trans*-decalin **26** (47.0 mg, 48 %) as a colorless oil. **26**: $R_f$ = 0.17 (silica, EtOAc:petroleum ether 1:10); IR (film): $\nu_{max}$ = 2953, 1737, 1712, 1275, 1259, 1176, 1143, 1091, 764, 750 $cm^{-1}$; $^1$H NMR (400 MHz, $CDCl_3$): δ = 4.95 (s, 1 H), 4.61 (s, 1

H), 4.35–4.22 (m, 2 H), 4.20–4.08 (m, 2 H), 2.97 (ddd, $J$ = 14.8, 14.8, 6.1 Hz, 1 H), 2.50–2.43 (m, 2 H), 2.15–2.05 (m, 2 H), 2.02 (s, 3 H), 2.02–1.91 (m, 3 H), 1.67 (ddd, $J$ = 14.2, 14.1, 4.4 Hz, 1 H), 1.60–1.57 (m, 1 H), 1.36 (s, 3 H), 0.99–0.94 (m, 2 H), 0.91 (s, 3 H), 0.03 (s, 9 H) ppm; $^{13}$C NMR (101 MHz, $CDCl_3$): δ = 208.14, 173.34, 171.25, 145.14, 108.59, 63.73, 61.37, 57.71, 57.24, 53.35, 39.14, 38.78, 37.53, 37.13, 25.75, 21.34, 21.13, 17.31, 13.42, −1.52 ppm; HRMS (*m/z*): $[M + Na]^+$ calcd for $C_{22}H_{36}O_5SiNa^+$ 431.2224, found 431.2221.

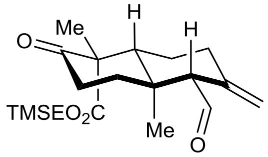


**28**

***trans*-Decalin aldehyde 28:** To a stirred solution of *trans*-decalin **26** (270 mg, 0.658 mmol) in MeOH (3.0 mL) was added anhydrous $K_2CO_3$ (136 mg, 0.987 mmol) at 22 ℃. The reaction mixture was stirred at that temperature for 3 h and then diluted with saturated aq. $NaHCO_3$ (10 mL). The resulting mixture was extracted with EtOAc (3 × 15 mL), and the combined organic phases were washed with brine (2 × 5 mL) and dried over anhydrous $MgSO_4$. After filtration and removal of the solvent under vacuum, the residue was passed through a short plug of silica using EtOAc/petroleum ether (1:5) as eluent to give the corresponding primary alcohol as a colorless oil. This oil was dissolved in $CH_2Cl_2$ (2.0 mL). To the solution was added Dess−Martin periodinane (558 mg, 1.32 mmol) at 22 ℃. After stirring at that temperature for 30 min, the reaction mixture was quenched with saturated aq. $NaHCO_3$ (10 mL). The resultant mixture was extracted with EtOAc (3 × 15 mL). The combined organic phases were dried over anhydrous $MgSO_4$ and filtered. The solvent was evaporated under vacuum, and the residue was purified by flash column chromatography with EtOAc/petroleum ether (1:15 → 1:10) to give *trans*-decalin aldehyde **28** (221 mg, 92 % for the two steps) as a colorless oil. **28**: $R_f$ = 0.31 (silica, EtOAc:petroleum ether 1:10); IR (film): $\nu_{max}$ = 2953, 1719, 1457, 1389, 1275,1221, 1176, 1097, 860, 838 $cm^{-1}$; $^1$H NMR (400 MHz, $CDCl_3$): δ = 9.86 (d, $J$ = 4.0 Hz, 1 H), 5.01 (s, 1 H), 4.57 (s, 1 H), 4.20–4.07 (m, 2 H), 2.96 (ddd, $J$ = 14.9, 14.8, 6.0 Hz, 1 H), 2.50–2.45 (m, 2 H), 2.44–2.38 (m, 1 H), 2.05–2.00 (m, 2 H), 2.00–1.91 (m, 2 H), 1.55 (ddd, $J$ = 14.3, 14.3, 4.4 Hz, 1 H), 1.48–1.43 (m, 1 H), 1.34 (s, 3 H), 1.22 (s, 3 H),

0.99–0.94 (m, 2 H), 0.01 (s, 9 H) ppm; $^{13}C$ NMR (101 MHz, $CDCl_3$): δ = 207.47, 203.23, 173.18, 143.22, 110.70, 66.06, 63.87, 57.71, 56.40, 38.89, 38.70, 36.93, 36.66, 25.10, 21.31, 17.35, 14.11, −1.52 ppm; HRMS (*m/z*): $[M + Na]^+$ calcd for $C_{20}H_{32}O_4SiNa^+$ 387.1962, found 387.1958.

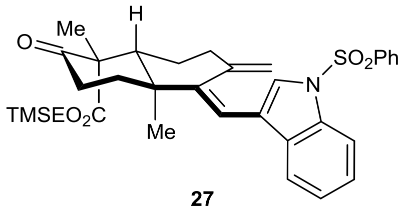


**27**

**Triene 27:** This compound was synthesized from *trans*-decalin aldehyde **28** (89.7 mg, 0.246 mmol) and Grignard reagent **15**[3] (1.85 mL, ca. 0.4 M in THF, ca. 0.740 mmol) by using the procedure for the preparation of triene **16**. Triene **27** (110 mg, 74 %) was obtained as a white powder. **27**: $R_f$ = 0.29 (silica, EtOAc:petroleum ether 1:10); IR (film): $\nu_{max}$ = 2952, 1712, 1477, 1372, 1250, 1176, 1120, 1095, 860, 838 $cm^{-1}$; $^1H$ NMR (400 MHz, $CDCl_3$): δ = 7.96 (d, *J* = 8.2 Hz, 1 H), 7.80 (d, *J* = 7.3 Hz, 2 H), 7.62 (s, 1 H), 7.50 (t, *J* = 7.5 Hz, 1 H), 7.44 (d, *J* = 7.6 Hz, 1 H), 7.40 (t, *J* = 7.7 Hz, 2 H), 7.32–7.27 (m, 1 H), 7.22 (t, *J* = 7.5 Hz, 1 H), 6.12 (s, 1 H), 4.97 (s, 1 H), 4.66 (d, *J* = 1.7 Hz, 1 H), 4.24–4.12 (m, 2 H), 3.13 (ddd, *J* = 14.7, 14.7, 5.9 Hz, 1 H), 2.63–2.57 (m, 2 H), 2.24–2.14 (m, 3 H), 2.09–2.00 (m, 2 H), 1.73–1.68 (m, 1 H), 1.39 (s, 3 H), 1.21 (s, 3 H), 1.09–0.97 (m, 2 H), 0.04 (s, 9 H) ppm; $^{13}C$ NMR (126 MHz, $CDCl_3$): δ = 208.14, 173.23, 153.44, 145.09, 138.23, 134.80, 133.77, 131.24, 129.23, 126.77, 124.83, 123.38, 123.29, 119.61, 119.50, 113.82, 113.80, 109.02, 63.82, 57.94, 56.00, 41.75, 37.42, 37.12, 36.81, 25.49, 21.50, 18.71, 17.36, −1.48 ppm; HRMS (*m/z*): $[M + Na]^+$ calcd for $C_{34}H_{41}N_1O_5SSiNa^+$ 626.2367, found 626.2371.

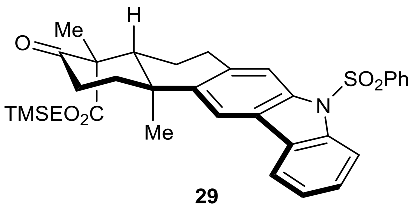


**29**

**Carbazole 29:** This compound was synthesized from triene **27** (17.6 mg, 0.0292 mmol) by using the procedure for the preparation of *N*-benzenesulfonylxiamycin A (trimethylsilyl)ethyl ester **17**. Carbazole **29** (14.6 mg, 83 %) was obtained as a white powder. **29**: $R_f$ = 0.36 (silica, EtOAc:petroleum ether 1:5);

IR (film): $\nu_{max}$ = 2954, 1712, 1275, 1148, 1118, 1092, 913, 749, 686, 595 $cm^{-1}$; $^1H$ NMR (500 MHz, $CDCl_3$): δ = 8.26 (d, *J* = 8.3 Hz, 1 H), 8.00 (s, 1 H), 7.84 (d, *J* = 8.3 Hz, 1 H), 7.83 (s, 1 H), 7.80 (d, *J* = 7.1 Hz, 2 H), 7.49–7.42 (m, 2 H), 7.36–7.30 (m, 3 H), 4.25–4.14 (m, 2 H), 3.26–3.14 (m, 2 H), 3.10–3.00 (m, 1 H), 2.71 (dd, *J* = 13.6, 4.9 Hz, 1 H), 2.64–2.57 (m, 1 H), 2.34–2.23 (m, 2 H), 1.90–1.82 (m, 2 H), 1.48 (s, 3 H), 1.41 (s, 3 H), 1.06–0.98 (m, 2 H), 0.05 (s, 9 H) ppm; $^{13}C$ NMR (101 MHz, $CDCl_3$): δ = 208.00, 173.80, 142.80, 138.68, 138.24, 137.09, 135.44, 133.87, 129.22, 127.26, 126.65, 126.56, 124.95, 124.01, 119.77, 116.75, 115.18, 114.96, 63.87, 57.88, 54.00, 39.74, 38.50, 37.64, 32.90, 23.66, 21.68, 21.09, 17.47, −1.44 ppm; HRMS (*m/z*): $[M + Na]^+$ calcd for $C_{34}H_{39}N_1O_5SSiNa^+$ 624.2210, found 624.2206.

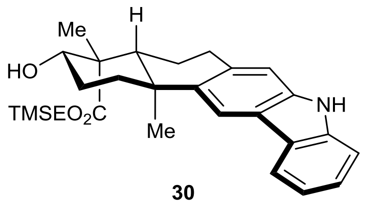


**30**

**Oridamycin A (trimethylsilyl)ethyl ester 30:** To a stirred solution of **29** (13.6 mg, 0.0226 mmol) in MeOH (0.50 mL) was added $NaBH_4$ (2.6 mg, 0.068 mmol) at 0 ℃. The reaction mixture was stirred at that temperature for 30 min before it was quenched with aq. HCl (1 mL, 1.0 M). The resulting mixture was extracted with EtOAc (3 × 5 mL), and the combined organic phases were washed with brine (5 mL) and dried over anhydrous $Na_2SO_4$. After filtration and removal of the solvent under vacuum, the residue was passed through a short plug of silica using EtOAc/petroleum (1:5) as eluent to give the corresponding secondary alcohol a colorless oil. This oil was dissolved in MeOH (0.50 mL). To the solution was added magnesium powder (4.4 mg, 0.18 mmol) at 22 ℃. The resulting mixture was sonicated at that temperature for 1 h before it was quenched with saturated aq. $NH_4Cl$ (4 mL). The mixture so obtained was extracted with EtOAc (3 × 5 mL). The combined organic phases were washed with brine (5 mL), dried over anhydrous $MgSO_4$, and filtered. The solvent was evaporated under vacuum, and the residue was purified by flash column chromatography with EtOAc/petroleum ether (1:4) to give oridamycin A (trimethylsilyl)ethyl ester **30** (8.7 mg, 83 % for the two steps) as a white foam. **30**: $R_f$ = 0.39 (silica, EtOAc:petroleum ether 1:5); IR (film): $\nu_{max}$ = 3411, 2953, 1695, 1608, 1467,

1320, 1250, 1241, 1179, 1034, 940, 858, 838, 748, 735 cm$^{-1}$; $^{1}$H NMR (500 MHz, $CDCl_3$): δ = 8.01 (d, *J* = 7.8 Hz, 1 H), 7.98 (s, 1 H), 7.86 (s, 1 H), 7.42–7.32 (m, 2 H), 7.20 (t, *J* = 7.0 Hz, 1 H), 7.05 (s, 1 H), 4.26–4.14 (m, 2 H), 3.74–3.67 (m, 1 H), 3.22 (ddd, *J* = 12.0, 12.0, 4.3 Hz, 1 H), 3.11 (dd, *J* = 16.7, 4.0 Hz, 1 H), 3.04–2.97 (m, 1 H), 2.57 (ddd, *J* = 13.2, 3.4, 3.4 Hz, 1 H), 2.32–2.24 (m, 2 H), 2.17–2.01 (m, 2 H), 1.71–1.61 (m, 1 H), 1.55 (s, 3 H), 1.22 (s, 3 H), 1.12–0.98 (m, 2 H), 0.06 (s, 9 H) ppm; $^{13}$C NMR (126 MHz, $CDCl_3$): δ = 178.42, 140.20, 139.69, 138.29, 133.80, 125.60, 123.60, 122.36, 120.07, 119.30, 117.22, 110.56, 109.76, 78.41, 62.95, 53.09, 49.12, 39.14, 38.64, 33.30, 29.51, 24.27, 23.91, 21.60, 17.54, −1.43 ppm; HRMS (*m/z*): $[M + Na]^+$ calcd for $C_{28}H_{37}N_1O_3SiNa^+$ 486.2435, found 486.2439.

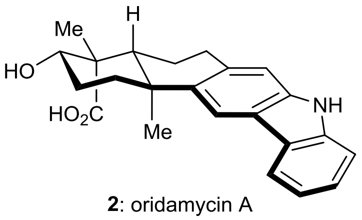


**2**: oridamycin A

**Oridamycin A:** This compound was synthesized from oridamycin A (trimethylsilyl)ethyl ester **30** (12.8 mg, 0.0276 mmol) by using the procedure for the preparation of xiamycin A (**1**). The crude product was subjected to HPLC (Waters 2535Q, SunFire Prep C18 OBD column, 19 mm × 250 mm) using acetonitrile/water (40:60 → 80:20, 10 mL/min) as eluent to give oridamycin A (**2**, $t_R$ = 17.80 min). After removal of the solvent under vacuum, **2** (9.2 mg, 92 %) was obtained as a white powder. **2**: $R_f$ = 0.35 (silica, MeOH/$CH_2Cl_2$ 1:15); IR (film): $\nu_{max}$ = 3410, 2923, 2852, 2533, 1654, 1638, 1560, 1466, 1437, 1075, 1031, 751, 669 cm$^{-1}$; $^{1}$H NMR (400 MHz, methanol-$d_4$): δ = 7.96 (d, *J* = 8.0 Hz, 1 H), 7.95 (s, 1 H), 7.34 (d, *J* = 8.0 Hz, 1 H), 7.28 (t, *J* = 7.6 Hz, 1 H), 7.08 (t, *J* = 7.6 Hz, 1 H), 7.05 (s, 1 H), 3.24 (dd, *J* = 12.1, 4.3 Hz, 1 H), 3.08 (dd, *J* = 16.5, 4.0 Hz, 1 H), 3.01–2.89 (m, 1 H), 2.59 (dd, *J* = 13.2, 2.0 Hz, 1 H), 2.38–2.26 (m, 1 H), 2.25–2.19 (m, 1 H), 2.19–2.06 (m, 1 H), 1.97–1.89 (m, 1 H), 1.59 (ddd, *J* = 13.6, 13.6, 3.5 Hz, 1 H), 1.51 (d, *J* = 12.9 Hz, 1 H), 1.49 (s, 3 H), 1.27 (s, 3 H) ppm; $^{13}$C NMR (101 MHz, methanol-$d_4$): δ = 180.96, 142.07, 140.37, 140.13, 134.49, 126.08, 124.60, 123.26, 120.58, 119.35, 117.49, 111.44, 110.69, 79.06, 54.07, 49.80, 39.99, 39.63, 34.00, 30.27, 24.76, 24.56, 22.48 ppm; HRMS (*m/z*): $[M]^+$ calcd for $C_{23}H_{24}N_1O_3^+$ 362.1762, found 362.1763.

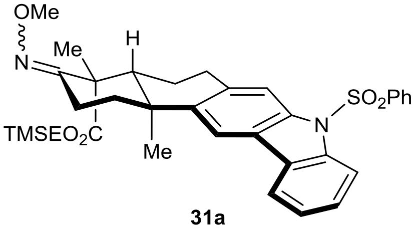


**31a**

***O*-Methyloxime 31a:** To a stirred solution of carbazole **29** (45.1 mg, 0.0750 mmol) in MeOH/pyridine (2.0 mL, 5:1) was added $NH_2OMe$•HCl (12.5 mg, 0.151 mmol) at 22 ℃. The reaction mixture was stirred at that temperature for 2 h before it was quenched with saturated aq. $NaHCO_3$ (5 mL). The resulting mixture was extracted with EtOAc (3 × 5 mL). The combined organic phases were washed with brine (2 × 5 mL) and dried over anhydrous $MgSO_4$. After filtration and evaporation of the solvent, the residue was subjected to flash column chromatography for purification using EtOAc/petroleum ether (1:15) as eluent to give *O*-methyloxime **31a** (44.4 mg, 94 %) as a white powder. **31a**: $R_f$ = 0.42 (silica, EtOAc:petroleum ether 1:15); IR (film): $\nu_{max}$ = 2925, 1721, 1275, 1260, 1176, 1051, 913, 748, 596, 477 $cm^{-1}$; $^1$H NMR (400 MHz, $CDCl_3$): δ = 8.25 (d, *J* = 8.3 Hz, 1 H), 7.97 (s, 1 H), 7.83 (d, *J* = 8.2 Hz, 1 H), 7.82–7.80 (m, 2 H), 7.77 (s, 1 H), 7.48–7.40 (m, 2 H), 7.36–7.30 (m, 3 H), 4.24–4.13 (m, 2 H), 3.85 (s, 3 H), 3.45 (ddd, *J* = 14.9, 4.4, 2.3 Hz, 1 H), 3.19 (dd, *J* = 17.0, 4.1 Hz, 1 H), 3.08–2.98 (m, 1 H), 2.50 (ddd, *J* = 12.9, 5.0, 2.3 Hz, 1 H), 2.43–2.31 (m, 2 H), 2.25–2.11 (m, 1 H), 1.77 (dd, *J* = 12.0, 1.6 Hz, 1 H), 1.62–1.53 (m, 1 H), 1.57 (s, 3 H), 1.27 (s, 3 H), 1.06–1.00 (m, 2 H), 0.05 (s, 9 H) ppm; $^{13}$C NMR (126 MHz, $CDCl_3$): δ = 175.47, 159.72, 143.66, 138.62, 138.20, 136.88, 135.71, 133.81, 129.17, 127.10, 126.71, 126.62, 124.82, 123.96, 119.75, 116.80, 115.15, 114.80, 63.44, 61.53, 53.95, 50.73, 38.73, 33.04, 23.67, 22.34, 21.40, 20.51, 17.40, −1.40 ppm; HRMS (*m/z*): $[M + Na]^+$ calcd for $C_{35}H_{42}N_2O_5SSiNa^+$ 653.2476, found 653.2478.

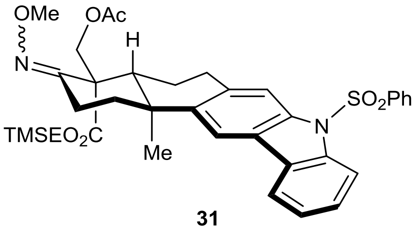


**31**

**Acetoxy *O*-methyloxime 31:** To a stirred solution of *O*-methyloxime **31a** (40.4 mg, 0.0641 mmol) in $Ac_2O$/AcOH (0.60 mL, 1:1) were sequentially added $Pd(OAc)_2$ (2.9 mg, 0.013 mmol) and $PhI(OAc)_2$

(20.6 mg, 0.0641 mmol) at 22 °C. The resulting mixture was heated to 110 °C and stirred at that temperature for 1 h before it was cooled to 22 °C and quenched with saturated aq. $NaHCO_3$ (10 mL). The mixture so obtained was extracted with EtOAc (3 × 10 mL), and the combined organic phases were dried over anhydrous $MgSO_4$ and filtered. The solvent was evaporated under vacuum, and the residue was subjected to flash column chromatography for purification using EtOAc/petroleum ether (1:10→1:5) as eluent to give acetoxy *O*-methyloxime **31** (36.3 mg, 82 %) as a white powder. **31**: $R_f$ = 0.29 (silica, EtOAc:petroleum ether 1:9); IR (film): $\nu_{max}$ = 2952, 1681, 1488, 1467, 1423, 1373, 1246, 1176, 1050, 749 $cm^{-1}$; $^1H$ NMR (400 MHz, $CDCl_3$): δ = 8.26 (d, *J* = 8.3 Hz, 1 H), 7.98 (s, 1 H), 7.85 (d, *J* = 7.5 Hz, 2 H), 7.83–7.80 (m, 2 H), 7.50–7.41 (m, 2 H), 7.38–7.31 (m, 3 H), 4.66 (d, *J* = 10.7 Hz, 1 H), 4.54 (d, *J* = 10.7 Hz, 1 H), 4.25–4.19 (m, 2 H), 3.83 (s, 3 H), 3.29–3.13 (m, 2 H), 3.11–2.99 (m, 1 H), 2.60–2.48 (m, 2 H), 2.17–2.12 (m, 2 H), 2.10–2.05 (m, 1 H), 2.03 (s, 3 H), 1.66–1.60 (m, 1 H), 1.30 (s, 3 H), 1.06–1.01 (m, 2 H), 0.05 (s, 9 H) ppm; $^{13}C$ NMR (126 MHz, $CDCl_3$): δ = 172.12, 170.74, 156.10, 143.27, 138.63, 138.29, 136.93, 135.35, 133.85, 129.22, 127.20, 126.65, 126.57, 124.80, 123.96, 119.78, 116.70, 115.10, 114.87, 65.87, 63.92, 61.90, 54.42, 46.83, 38.13, 36.80, 32.41, 23.79, 21.22, 21.13, 20.78, 17.48, –1.42 ppm; HRMS (*m/z*): $[M + Na]^+$ calcd for $C_{37}H_{44}N_2O_7SSiNa^+$ 711.2531, found 711.2539.

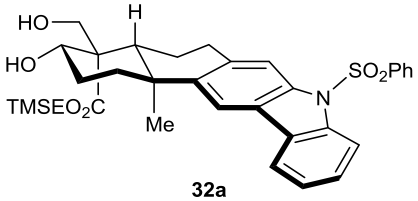

**32a**

***N*-Benzenesulfonyl Oridamycin B (trimethylsilyl)ethyl ester 32a:** To a stirred solution of acetoxy *O*-methyloxime **31** (9.7 mg, 0.014 mmol) in acetone (0.30 mL) was added aq. $HClO_4$ (0.10 mL, 6.0 M) at 22 °C. The resultant mixture was vigorously stirred at that temperature for 12 h before it was quenched with saturated aq. $NaHCO_3$ (5 mL). The resulting mixture was extracted with $CH_2Cl_2$ (5 × 5 mL). The combined organic phases were sequentially washed with saturated aq. $NaHCO_3$ (3 mL) and brine (3 mL), and dried over anhydrous $Na_2SO_4$. After filtration and evaporation under vacuum, the residue was dissolved in MeOH (0.30 mL). To this solution was added $NaBH_4$ (1.6 mg, 0.042 mmol) at 0 °C. After stirring at that temperature for 30 min, the reaction mixture was quenched with aq. HCl (1 mL, 1.0 M),

diluted with brine (4 mL), and extracted with EtOAc (5 × 3 mL). The combined organic phases were sequentially washed with saturated aq. $NaHCO_3$ (2 mL) and brine (2 mL), dried over anhydrous $Na_2SO_4$, filtered, and concentrated under vacuum. The residue so obtained was purified by flash column chromatography with EtOAc/petroleum ether (1:2) to give *N*-benzenesulfonyl oridamycin B (trimethylsilyl)ethyl ester **32a** (5.4 mg, 62 % for the two steps) as a white foam. **32a**: $R_f$ = 0.33 (silica, EtOAc:petroleum ether 1:2); IR (film): $\nu_{max}$ = 3648, 2922, 2864, 1770, 1246, 1055, 1013, 749, 669, 474 $cm^{-1}$; $^1H$ NMR (400 MHz, $CDCl_3$): δ = 8.25 (d, *J* = 8.3 Hz, 1 H), 7.96 (s, 1 H), 7.85–7.79 (m, 3 H), 7.77 (s, 1 H), 7.49–7.41 (m, 2 H), 7.36–7.30 (m, 3 H), 4.37 (d, *J* = 9.4 Hz, 1 H), 4.33–4.21 (m, 2 H), 3.94–3.87 (m, 1 H), 3.81 (d, *J* = 10.5 Hz, 1 H), 3.69–3.61 (m, 1 H), 3.15 (dd, *J* = 17.1, 3.8 Hz, 1 H), 3.06–2.95 (m, 1 H), 2.48 (ddd, *J* = 13.2, 3.3, 3.3 Hz, 1 H), 2.40–2.27 (m, 1 H), 2.18 (dd, *J* = 13.9, 6.1 Hz, 1 H), 2.12–1.97 (m, 2 H), 1.64–1.55 (m, 3 H), 1.21 (s, 3 H), 1.16–1.01 (m, 2 H), 0.06 (s, 9 H) ppm; $^{13}C$ NMR (126 MHz, $CDCl_3$): δ = 176.01, 143.67, 138.65, 138.25, 136.88, 135.29, 133.83, 129.20, 127.20, 126.64, 124.90, 124.00, 119.78, 116.93, 115.16, 114.72, 79.03, 71.50, 63.64, 53.19, 47.01, 38.46, 38.41, 32.93, 29.28, 24.24, 21.20, 17.64, −1.44 ppm; HRMS (*m/z*): $[M + Na]^+$ calcd for $C_{34}H_{41}N_1O_6SSiNa^+$ 642.2316, found 642.2313.

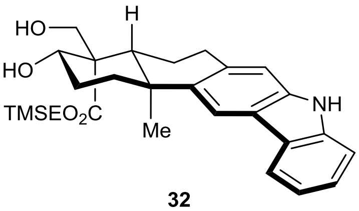


**32**

**Oridamycin B (trimethylsilyl)ethyl ester 32:** This compound was synthesized from *N*-benzenesulfonyl oridamycin B (trimethylsilyl)ethyl ester **32a** (10.4 mg, 0.0168 mmol) by using the procedure for the preparation of xiamycin A (trimethylsilyl)ethyl ester **21**. Oridamycin B (trimethylsilyl)ethyl ester **32** (7.2 mg, 90 %) was obtained as a white foam. **32**: $R_f$ = 0.42 (silica, EtOAc:petroleum ether 2:3); IR (film): $\nu_{max}$ = 3414, 2963, 2912, 2849, 1698, 1652, 1616, 1575, 1464, 1261, 1093, 1028, 800, 692, 669 $cm^{-1}$; $^1H$ NMR (400 MHz, $CDCl_3$): δ = 8.00 (d, *J* = 7.7 Hz, 1 H), 7.96 (s, 1 H), 7.84 (s, 1 H), 7.37 (d, *J* = 3.7 Hz, 2 H), 7.23–7.16 (m, 1 H), 7.07 (s, 1 H), 4.39 (dd, *J* = 10.5, 10.4 Hz, 1 H), 4.30–4.25 (m, 2 H), 3.99 (d, *J* = 10.5 Hz, 1 H), 3.93 (d, *J* = 11.9 Hz, 1 H), 3.81 (d, *J* =

10.4 Hz, 1 H), 3.67 (ddd, $J$ = 12.0, 11.9, 4.5 Hz, 1 H), 3.14–3.05 (m, 1 H), 3.04–2.93 (m, 1 H), 2.59 (ddd, $J$ = 13.3, 3.4, 3.4 Hz, 1 H), 2.42–2.30 (m, 1 H), 2.19–2.12 (m, 1 H), 2.09–2.00 (m, 1 H), 1.70–1.62 (m, 2 H), 1.26 (s, 3 H), 1.14–1.04 (m, 2 H), 0.06 (s, 9 H) ppm; $^{13}$C NMR (126 MHz, $CDCl_3$): δ = 176.12, 140.21, 139.28, 138.32, 133.51, 125.71, 123.58, 122.40, 120.08, 119.39, 117.12, 110.60, 109.77, 79.48,71.95, 63.55, 53.20, 47.61, 38.87, 38.43, 32.85, 29.49, 24.54, 21.39, 17.63, −1.43 ppm; HRMS (*m/z*): $[M + Na]^+$ calcd for $C_{28}H_{37}N_1O_4Na^+$ 502.2384, found 502.2388.

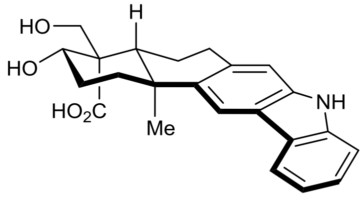


**3**: oridamycin B

**Oridamycin B (3)**: This compound was synthesized from oridamycin B (trimethylsilyl)ethyl ester **32** (6.8 mg, 0.014 mmol) by using the procedure for the preparation of xiamycin A (**1**). The crude product was subjected to HPLC (Waters 2535Q, SunFire Prep C18 OBD column, 19 mm × 250 mm) using acetonitrile/water (40:60 → 80:20, 10 mL/min) as eluent to give oridamycin B (**3**, $t_R$ = 14.53 min). After removal of the solvent under vacuum, **3** (4.9 mg, 91 %) was obtained as a white powder. **3**: $R_f$ = 0.48 (silica, MeOH/$CH_2Cl_2$ 1:6); IR (film): $\nu_{max}$ = 3409, 2924, 2856, 2540, 1703, 1680, 1464, 1339, 1045, 862, 751 cm$^{-1}$; $^1$H NMR (600 MHz, methanol-d$_4$): δ = 7.97 (s, 1 H), 7.96 (d, $J$ = 7.2 Hz, 1 H), 7.35 (d, $J$ = 8.0 Hz, 1 H), 7.28 (t, $J$ = 7.5 Hz, 1 H), 7.08 (d, $J$ = 8.0 Hz, 1 H), 7.07 (s, 1 H), 4.11 (d, $J$ = 10.8 Hz, 1 H), 3.92 (d, $J$ = 10.8 Hz, 1 H), 3.76 (dd, $J$ = 12.2, 4.4 Hz, 1 H), 3.05 (dd, $J$ = 10.3, 4.5 Hz, 1 H), 2.62 (d, $J$ = 13.2 Hz, 1 H), 2.47–2.39 (m, 1 H), 2.22–2.17 (m, 1 H), 2.13–2.04 (m, 1 H), 1.99–1.95 (m, 1 H), 1.94 (d, $J$ = 12.2 Hz, 1 H), 1.61 (ddd, $J$ = 13.5, 13.5, 3.5 Hz, 1 H), 1.32 (s, 3 H) ppm; $^{13}$C NMR (151 MHz, methanol-d$_4$): δ = 178.66, 142.06, 140.59, 140.14, 134.57, 126.05, 124.61, 123.16, 120.56, 119.33, 117.36, 111.42, 110.67, 73.53, 64.55, 55.49, 46.39, 39.79, 39.35, 33.38, 29.82, 25.04, 22.14 ppm; HRMS (*m/z*): $[M]^+$ calcd for $C_{23}H_{24}N_1O_4^+$ 378.1711, found 378.1717.

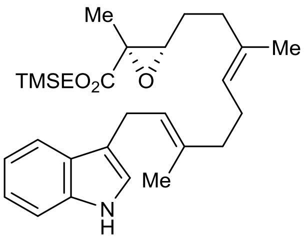


**34**

**Indolyl epoxy (trimethylsilyl)ethyl ester 34:** To a stirred solution of epoxy (trimethylsilyl)ethyl ester **12** (108 mg, 0.263 mmol) and *N*-Boc-3-(tributylstannyl)indole **33**[7] (289 mg, 0.526 mmol) in DMF (3.0 mL) were sequentially added anhydrous LiCl (33.5 mg, 0.789 mmol) and $Pd_2(dba)_3$ (12.1 mg, 0.0132 mmol) at 22 °C. The resulting mixture was heated to 80 °C and stirred at that temperature for 2 h before it was cooled to 22 °C and quenched with saturated aq. $NaHCO_3$ (5 mL). After extraction with EtOAc (3 × 10 mL), the combined organic phases were washed with brine (10 mL), dried over anhydrous $MgSO_4$, and filtered. The solvent was removed under vacuum, and the residue was passed through a short plug of silica using EtOAc/petroleum ether (1:9) as eluent to give the desired coupling product as a pale yellow oil. This oil was dissolved in DMSO (3.0 ml). The resulting mixture was heated to 150 °C and stirred at that temperature for 30 min before it was cooled to 22 °C. The mixture so obtained was subjected to flash column chromatography for purification using EtOAc/petroleum ether (1:10) as eluent to give indolyl epoxy (trimethylsilyl)ethyl ester **34** (81.1 mg, 66 %) as a colorless oil. **34**: $R_f$ = 0.23 (silica, EtOAc:petroleum ether 1:10); $[\alpha]_D^{26}$ = −8.9 (*c* = 0.93 in $CHCl_3$); IR (film): $\nu_{max}$ = 2954, 1740, 1449, 1382, 1277, 1230, 1164, 1038, 949, 771 $cm^{-1}$; $^1H$ NMR (400 MHz, $CDCl_3$): δ = 8.07 (s, 1 H), 7.60 (d, *J* = 7.8 Hz, 1 H), 7.35 (d, *J* = 8.1 Hz, 1 H), 7.19 (dt, *J* = 8.0, 1.1 Hz, 1 H), 7.12 (dt, *J* = 8.0, 1.0 Hz, 1 H), 6.95–6.92 (m, 1 H), 5.47 (td, *J* = 7.1, 1.1 Hz, 1 H), 5.20 (dd, *J* = 6.8, 6.6 Hz, 1 H), 4.29–4.22 (m, 2 H), 3.48 (d, *J* = 7.1 Hz, 2 H), 3.17 (dd, *J* = 6.1, 6.1 Hz, 1 H), 2.20–2.14 (m, 3 H), 2.12–2.07 (m, 3 H), 1.78 (s, 3 H), 1.69–1.65 (m, 2 H), 1.63 (s, 3 H), 1.52 (s, 3 H), 1.06–1.02 (m, 2 H), 0.06 (d, *J* = 3.0 Hz, 9 H) ppm; $^{13}C$ NMR (126 MHz, $CDCl_3$): δ = 171.84, 136.61, 135.29, 133.59, 127.56, 125.34, 123.35, 121.92, 121.35, 119.13, 119.06, 116.02, 111.18, 64.11, 62.19, 57.80, 39.60, 36.10, 26.76, 26.51, 24.07, 17.45, 16.14, 16.12, 13.69, −1.42 ppm; HRMS (*m/z*): $[M + Na]^+$ calcd for $C_{28}H_{41}N_1O_3SiNa^+$ 490.2748, found 490.2748.

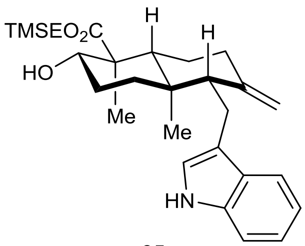


**35**

**Indosespene (trimethylsilyl)ethyl ester 35:** A mixture of $Cp_2TiCl_2$ (14.3 mg, 0.0574 mmol) and Mn dust (127 mg, 2.31 mmol) in THF (7.0 mL) was stirred at 22 ℃ for 15 min. To the resulting mixture were sequentially added indolyl epoxy (trimethylsilyl)ethyl ester **34** (134 mg, 0.287 mmol), $i\text{Pr}_2\text{NEt}$ (222 mg, 300 $\mu$L, 1.72 mmol), and TMSCl (155 mg, 180 $\mu$L, 1.44 mmol). The reaction mixture was stirred at that temperature for 4 h before it was quenched with aq. HCl (6 mL, 2.0 M). The mixture so obtained was extracted with EtOAc (3 × 15 mL), and the combined organic phases were washed with brine (10 mL), dried over anhydrous $Mg_2SO_4$, and filtered. After removal of the solvent under vacuum, the residue was purified by flash column chromatography with EtOAc/petroleum ether (1:5) as eluent to give indosespene (trimethylsilyl)ethyl ester **35** (80.7 mg, 60 %) as a pale yellow foam. **35**: $R_f$ = 0.46 (silica, EtOAc:petroleum ether 1:3); $[\alpha]_D^{24}$ = +40.4 ($c$ = 0.45 in $CHCl_3$); IR (film): $\nu_{max}$ = 3417, 2933, 1709, 1456, 1249, 1153, 1089, 1039, 889, 858 $cm^{-1}$; $^1$H NMR (400 MHz, $CDCl_3$): δ = 7.89 (s, 1 H), 7.62 (d, $J$ = 7.8 Hz, 1 H), 7.33 (d, $J$ = 8.0 Hz, 1 H), 7.18 (t, $J$ = 7.3 Hz, 1 H), 7.12 (t, $J$ = 7.3 Hz, 1 H), 6.90 (s, 1 H), 4.84 (s, 1 H), 4.73 (s, 1 H), 4.27–4.16 (m, 2 H), 4.06 (d, $J$ = 10.1 Hz, 1 H), 2.99 (d, $J$ = 15.1 Hz, 1 H), 2.85 (dd, $J$ = 15.3, 10.6 Hz, 1 H), 2.38–2.27 (m, 2 H), 2.11–2.02 (m, 2 H), 1.96 (dd, $J$ = 12.6, 2.5 Hz, 1 H), 1.86–1.80 (m, 1 H), 1.74–1.67 (m, 1 H), 1.65–1.54 (m, 2 H), 1.54–1.47 (m, 1 H), 1.30–1.26 (m, 1 H), 1.17 (s, 3 H), 1.04–0.98 (m, 2 H), 0.88 (s, 3 H), 0.06 (s, 9 H) ppm; $^{13}$C NMR (126 MHz, $CDCl_3$): δ = 177.68, 147.46, 136.19, 127.86, 121.93, 121.87, 119.19, 118.82, 115.92, 111.23, 108.62, 75.68, 63.44, 56.30, 53.83, 50.40, 39.17, 37.71, 37.08, 27.36, 26.41, 19.81, 17.57, 14.88, 10.90, –1.32 ppm; HRMS ($m/z$): $[M + Na]^+$ calcd for $C_{28}H_{41}N_1O_3SiNa^+$ 490.2748, found 490.2747.

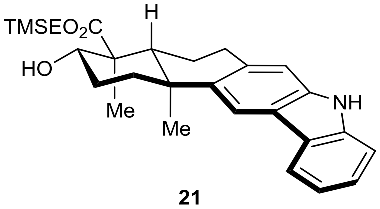


**21**

**Xiamycin A (trimethylsilyl)ethyl ester 21:** To a stirred solution of indosespene (trimethylsilyl)ethyl ester **35** (6.7 mg, 0.014 mmol) in AcOH/toluene (1:4, 0.80 mL) were sequentially added *p*-benzoquinone (3.1 mg, 0.029 mmol) and $Pd(OAc)_2$ (0.7 mg, 0.003 mmol) at 22 ℃. The resulting mixture was warmed to 50 ℃ and stirred at that temperature for 2 h before it was cooled to 22 ℃ and quenched with saturated aq. $NaHCO_3$ (5 mL). After extraction with EtOAc (3 × 5 mL), the combined organic phases were dried over anhydrous $MgSO_4$ and filtered. The solvent was evaporated under vacuum, and the residue was subjected to flash column chromatography for purification using EtOAc/petroleum ether (1:4) as eluent to give xiamycin A (trimethylsilyl)ethyl ester **21** (5.4 mg, 81 % ) as a white foam.

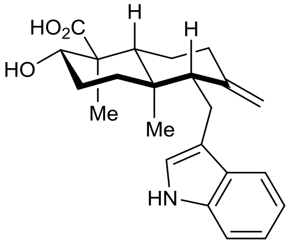


**6:** indosespene

**Indosespene (6):** This compound was synthesized from indosespene (trimethylsilyl)ethyl ester **35** (32.7. mg, 0.0699 mmol) by using the procedure for the preparation of xiamycin A (**1**). The crude product was subjected to HPLC (Waters 2535Q, SunFire Prep C18 OBD column, 19 mm × 250 mm) using acetonitrile/water (40:60 → 80:20, 10 mL/min) as eluent to give indosespene (**6**, $t_R$ = 22.71 min). After removal of the solvent under vacuum, **6** (23.3 mg, 91 %) was obtained as a white powder. **6**: $R_f$ = 0.27 (silica, MeOH:$CH_2Cl_2$ 1:15); m.p. 210–211 ℃ (EtOAc); $[\alpha]_D^{26}$ = +52.4 (*c* = 0.45 in MeOH); IR (film): $\nu_{max}$ = 3706, 2980, 2972, 2922, 2865, 1702, 1454, 1055, 1032, 1012 $cm^{-1}$; $^1H$ NMR (400 MHz, methanol-$d_4$): $\delta$ = 7.53 (d, *J* = 7.8 Hz, 1 H), 7.29 (d, *J* = 8.0 Hz, 1 H), 7.05 (t, *J* = 7.5 Hz, 1 H), 6.99 (t, *J* = 7.4 Hz, 1 H), 6.90 (s, 1 H), 4.80 (s, 1 H), 4.73 (s, 1 H), 4.06 (dd, *J* = 11.2, 5.1 Hz, 1 H), 2.97 (d, *J* =

14.1 Hz, 1 H), 2.83 (dd, $J$ = 15.2, 10.6 Hz, 1 H), 2.37–2.32 (m, 1 H), 2.28 (d, $J$ = 10.0 Hz, 1 H), 2.09 (ddd, $J$ = 13.2, 3.2, 3.2 Hz, 1 H), 2.05–1.97 (m, 1 H), 1.95 (dd, $J$ = 12.7, 2.6 Hz, 1 H), 1.79–1.74 (m, 1 H), 1.74–1.68 (m, 1 H) 1.65–1.56 (m, 1 H), 1.55–1.45 (m, 1 H), 1.35–1.29 (m, 1 H), 1.11 (s, 3 H), 0.88 (s, 3 H) ppm; $^{13}$C NMR (126 MHz, methanol-d$_4$): δ = 181.24, 148.84, 137.87, 129.01, 123.27, 121.97, 119.25, 119.15, 115.65, 112.07, 108.91, 76.38, 57.86, 54.88, 51.98, 39.99, 38.79, 38.48, 28.30, 27.44, 20.75, 15.11, 11.42 ppm; HRMS (*m/z*): $[M + Na]^+$ calcd for $C_{23}H_{29}N_1O_3Na^+$ 390.2040, found 390.2038. CCDC 949054 contains the supplementary crystallographic data for **6** and is available free of charge from The Cambridge Crystallographic Data Centre via www.ccdc.cam.ac.uk/data_request/cif.

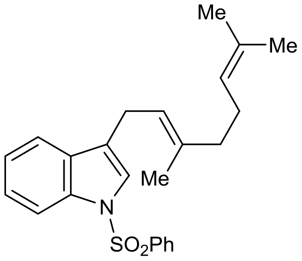

**37a**

***N*-Benzenesulfonyl 3-geranyl indole 37a:** To a stirred solution of 3-geranyl indole **37**[8] (504 mg, 1.99 mmol) in toluene (8.0 mL) were sequentially added $PhSO_2Cl$ (706 mg, 3.98 mmol), aq. NaOH (4.0 mL, 50 wt%), and $Bu_4NBr$ (63.6 mg, 0.198 mmol) at 0 ℃. The resulting mixture was warmed to 22 ℃ and stirred at that temperature for 3 h before it was diluted with water (40 ml) and extracted with EtOAc (3 × 30 mL). The combined organic phases were sequentially washed with saturated aq. $NaHCO_3$ (30 mL) and brine (30 mL) and dried over anhydrous $MgSO_4$. After filtration and evaporation of the solvent under vacuum, the residue was purified with flash column chromatography with EtOAc/petroleum ether (1:20) to give *N*-benzenesulfonyl 3-geranyl indole **37a** (751 mg, 96 %) as a pale yellow oil. **37a**: $R_f$ = 0.36 (silica, EtOAc:petroleum ether 1:20); IR (film): $\nu_{max}$ = 3528, 3062, 2967, 2855, 1729, 1680, 1448, 1375, 1278, 1176, 1120, 974, 746, 723, 594 cm$^{-1}$; $^1$H NMR (400 MHz, $CDCl_3$): δ = 8.01 (d, $J$ = 8.2 Hz, 1 H), 7.88 (d, $J$ = 7.6 Hz, 2 H), 7.56–7.51 (m, 1 H), 7.49 (d, $J$ = 7.8 Hz, 1 H), 7.44 (t, $J$ = 7.8 Hz, 2 H), 7.36–7.30 (m, 2 H), 7.25 (t, $J$ = 7.5 Hz, 1 H), 5.39 (t, $J$ = 7.0 Hz, 1 H), 5.13 (dd, $J$ = 6.3, 6.3 Hz, 1 H), 3.38 (d, $J$ = 7.0 Hz, 2 H), 2.19–2.12 (m, 2 H), 2.12–2.06 (m, 2 H), 1.74 (s, 3 H), 1.71 (s, 3 H), 1.64 (s, 3

H) ppm; $^{13}C$ NMR (101 MHz, $CDCl_3$): δ = 138.41, 137.45, 135.62, 133.70, 131.76, 131.19, 129.25, 126.79, 124.78, 124.15, 123.17, 123.14, 122.78, 120.78, 119.77, 113.83, 39.72, 26.62, 25.83, 23.92, 17.85, 16.26 ppm; HRMS (*m/z*): $[M + Na]^+$ calcd for $C_{24}H_{27}N_1O_2SNa^+$ 416.1655, found 416.1653.

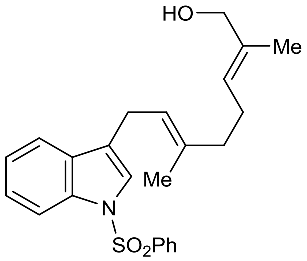


**38**

***N*-Benzenesulfonyl hydroxygeranyl indole 38:** To a stirred suspension of $SeO_2$ (452 mg, 4.08 mmol) in $CH_2Cl_2$ (30 mL) were sequentially added *t*BuOOH (7.40 mL, 5.5 M in decane, 40.7 mmol) and a solution of *N*-benzenesulfonyl 3-geranyl indole **37a** (8.02 g, 20.4 mmol) in $CH_2Cl_2$ (10 mL) at 0 ℃. The reaction mixture was stirred at that temperature for 10 h before it was quenched with saturated aq. $Na_2SO_3$ (200 mL). The resulting mixture was then extracted with EtOAc (4 × 200 mL). The combined organic phases were sequentially washed with aq. KOH (50 mL, 10 wt%) and brine (50 mL), dried over anhydrous $MgSO_4$, and filtered. The solvent was evaporated under vacuum. The residue so obtained was subjected to flash column chromatography for purification using EtOAc/petroleum ether (1:2) as eluent to give *N*-benzenesulfonyl hydroxygeranyl indole **38** (6.82 g, 82 %) as a pale yellow oil. **38**: $R_f$ = 0.43 (silica, EtOAc:petroleum ether 2:3); IR (film): $\nu_{max}$ = 3391, 2916, 2856, 1732, 1665, 1606, 1448, 1369, 1175, 1120, 973.8, 746, 686, 594 $cm^{-1}$; $^1H$ NMR (500 MHz, $CDCl_3$): δ = 7.98 (d, *J* = 8.3 Hz, 1 H), 7.87–7.84 (m, 2 H), 7.51 (t, *J* = 7.5 Hz, 1 H), 7.46 (d, *J* = 8.0 Hz, 1 H), 7.38 (t, *J* = 7.6 Hz, 2 H), 7.33–7.29 (m, 1 H), 7.27 (s, 1 H), 7.25–7.18 (m, 1 H), 5.47–5.32 (m, 2 H), 4.00 (s, 2 H) 3.34 (d, *J* = 7.1 Hz, 2 H), 2.24–2.18 (m, 2 H), 2.16–2.12 (m, 2 H), 1.70 (s, 3 H), 1.65 (s, 3 H) ppm; $^{13}C$ NMR (126 MHz, $CDCl_3$): δ = 138.34, 137.10, 135.61, 135.18, 133.70, 131.13, 129.24, 126.75, 125.33, 124.80, 123.16, 123.07, 122.79, 121.04, 119.69, 113.83, 68.76, 39.24, 25.93, 23.87, 16.17, 13.83 ppm; HRMS (*m/z*): $[M + Na]^+$ calcd for $C_{24}H_{27}N_1O_3SNa^+$ 432.1604, found 432.1609.

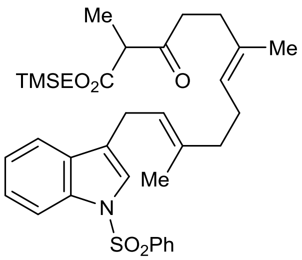

**36**

***N*-Benzenesulfonylindolyl *β*-ketoester 36:** To a stirred solution of *N*-benzenesulfonyl hydroxygeranyl indole **38** (298 mg, 0.728 mmol) in THF (4.0 mL) were sequentially added LiBr (316 mg, 3.64 mmol), $Et_3N$ (109 mg, 150 $\mu$L, 1.09 mmol), and MsCl (104 mg, 70 $\mu$L, 0.87 mmol) at 0 ℃. The resulting mixture was stirred at that temperature for 30 min before it was quenched with saturated aq. $NaHCO_3$ (10 mL) and extracted with diethyl ether (3 × 15 mL). The combined organic phases were sequentially washed with saturated aq. $NaHCO_3$ (5 ml) and saturated aq. NaBr (5 mL) and dried over anhydrous $MgSO_4$. After filtration and evaporation of the solvent under vacuum, the crude bromide was immediately dissolved in THF (5.0 mL) and used for the next step. To a stirred suspension of KH (35.0 mg, 0.874 mmol) in HMPA/THF (10 mL, 1:5) was added a solution of (trimethylsilyl)ethyl 2-methyl-3-oxobutanoate **39** (157 mg, 0.728 mmol) in THF (10.0 mL) at 0 ℃. The resulting mixture was stirred at that temperature for 30 min before BuLi (550 $\mu$L, 1.6 M in hexane, 0.874 mmol) was added. After stirring at 0 ℃ for 30 min, the solution of the freshly prepared bromide was added. The reaction mixture was stirred at that temperature for 3 h before it was quenched with saturated aq. $NH_4Cl$ (15 mL). The resulting mixture was then extracted with EtOAc (3 × 20 mL), and the combined organic phases were washed with saturated aq. $NH_4Cl$ (10 mL) and dried over anhydrous $Na_2SO_4$. After filtration and removal of the solvent under vacuum, the residue was purified by flash column chromatography with diethyl ether/petroleum ether (1:5) to give *N*-benzenesulfonylindolyl *β*-ketoester **36** (353 mg, 80 %) as a pale yellow oil. **36**: $R_f$ = 0.38 (silica, diethyl ether/petroleum ether 2:5); IR (film): $\nu_{max}$ = 3411, 3063, 2953, 1740, 1715, 1448, 1373, 1175, 1119, 859, 838, 746, 723, 686, 594, 572 $cm^{-1}$; $^1$H NMR (400 MHz, $CDCl_3$): δ = 7.98 (d, *J* = 8.2 Hz, 1 H), 7.85 (d, *J* = 7.9 Hz, 2 H), 7.54–7.49 (m, 1 H), 7.46 (d, *J* = 7.8 Hz, 1 H), 7.44–7.39 (m, 2 H), 7.30 (t, *J* = 7.7 Hz, 1 H), 7.27 (s, 1 H), 7.22 (t, *J* = 7.6 Hz, 1 H), 5.36 (t, *J* =

7.0 Hz, 1 H), 5.13 (t, $J$ = 6.7 Hz, 1 H), 4.25–4.17 (m, 2 H), 3.50 (q, $J$ = 7.1 Hz, 1 H), 3.35 (d, $J$ = 7.0 Hz, 2 H), 2.70–2.53 (m, 2 H), 2.26 (t, $J$ = 7.6 Hz, 2 H), 2.15–2.09 (m, 2 H), 2.08–2.02 (m, 2 H), 1.71 (s, 3 H), 1.60 (s, 3 H), 1.32 (d, $J$ = 7.1 Hz, 3 H), 1.01–0.97 (m, 2 H), 0.03 (s, 9 H) ppm; $^{13}$C NMR (101 MHz, $CDCl_3$): δ = 205.70, 170.85, 138.47, 137.31, 135.64, 133.81, 133.71, 131.17, 129.27, 126.81, 124.85, 124.80, 123.17, 123.03, 122.80, 120.91, 119.75, 113.85, 63.79, 53.09, 40.32, 39.59, 33.38, 26.64, 23.93, 17.44, 16.33, 16.23, 12.91, −1.40 ppm; HRMS (*m/z*): $[M + Na]^+$ calcd for $C_{34}H_{45}N_1O_5Na^+$ 630.2680, found 630.2686.

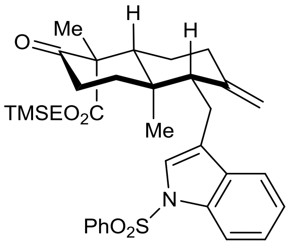


**40**

***N*-Benzenesulfonylindolyl decalin 40:** To a stirred suspension of $Mn(OAc)_3$•$2H_2O$ (457 mg, 1.68 mmol) and $Cu(OAc)_2$•$H_2O$ (169 mg, 0.849 mmol) in DMSO (8.0 mL) was added a solution of ***N***-benzenesulfonylindolyl *β*-ketoester **36** (516 mg, 0.852 mmol) in DMSO (2.0 mL) at 22 ℃. The resulting mixture was stirred at that temperature for 12 h before it was diluted with water (20 mL) and extracted with EtOAc (3 × 50 mL). The combined organic phases were washed with brine (2 × 20 mL) and dried over anhydrous $Na_2SO_4$. After filtration and evaporation of the solvent under vacuum, the residue was purified by flash column chromatography with diethyl ether/petroleum ether (1:10 → 1:5) to give *N*-benzenesulfonylindolyl decalin **40** (266 mg, 52 %) as a white foam. **40**: $R_f$ = 0.43 (silica, diethyl ether/petroleum ether 1:3); m.p. 114–116 ℃ (EtOAc/petroleum ether 1:1); IR (film): $\nu_{max}$ = 2953, 1712, 1448, 1371, 1250, 1176, 1120, 1096, 859, 838, 748, 723, 686, 594 cm$^{-1}$; $^1$H NMR (400 MHz, $CDCl_3$): δ = 7.96 (d, $J$ = 8.1 Hz, 1 H), 7.77 (d, $J$ = 7.5 Hz, 2 H), 7.49 (t, $J$ = 7.5 Hz, 1 H), 7.45 (d, $J$ = 8.3 Hz, 1 H), 7.37 (t, $J$ = 7.9 Hz, 2 H), 7.29 (t, $J$ = 7.7 Hz, 1 H), 7.25–7.20 (m, 1 H), 7.21 (s, 1 H), 4.90 (s, 1 H), 4.60 (s, 1 H), 4.23–4.10 (m, 2 H), 3.03 (ddd, $J$ = 14.7, 14.7, 6.0 Hz, 1 H), 2.88 (d, $J$ = 15.3 Hz, 1 H), 2.80 (dd, $J$ = 15.9, 10.4 Hz, 1 H), 2.50–2.44 (m, 2 H), 2.27 (ddd, $J$ = 13.0, 5.9, 2.4 Hz, 1 H), 2.22 (d, $J$ = 9.7 Hz, 1 H), 2.02–1.95 (m, 3 H), 1.66–1.58 (m, 2 H), 1.39 (s, 3 H), 1.02–0.97 (m, 2 H),

0.99 (s, 3 H), 0.04 (s, 9 H) ppm; $^{13}$C NMR (101 MHz, $CDCl_3$): δ = 208.43, 173.51, 146.17, 138.26, 135.35, 133.70, 131.46, 129.18, 126.77, 124.85, 123.74, 123.33, 122.71, 119.24, 114.10, 109.16, 63.73, 57.79, 57.47, 54.08, 40.24, 39.27, 38.02, 37.33, 26.21, 21.39, 20.35, 17.39, 12.72, −1.47 ppm; HRMS (*m/z*): $[M + Na]^+$ calcd for $C_{34}H_{43}N_1O_5SSiNa^+$ 628.2523, found 628.2525. CCDC 948503 contains the supplementary crystallographic data for **35** and is available free of charge from The Cambridge Crystallographic Data Centre via www.ccdc.cam.ac.uk/data_request/cif.

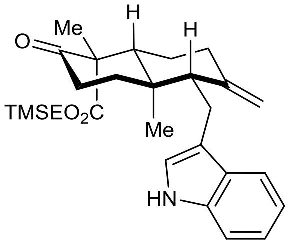


**40a**

**Indolyl decalin 40a:** To a solution of *N*-benzenesulfonylindolyl decalin **40** (81.1 mg, 0.134 mmol) in MeOH (1.0 mL) were sequentially added magnesium powder (9.6 mg, 0.400 mmol) and $NH_4Cl$ (8.6 mg, 0.160 mmol) at 22 °C. The resulting mixture was sonicated at that temperature for 1 h before it was quenched with saturated aq. $NH_4Cl$ (10 mL). After extraction with EtOAc (3 × 10 mL), the combined organic phases were washed with brine (10 mL), dried over anhydrous $MgSO_4$, and filtered. The solvent was evaporated under vacuum, and the residue so obtained was subjected to flash column chromatography using diethyl ether/petroleum ether (1:2) as eluent to give indolyl decalin **40a** (59.6 mg, 96 %) as a white foam. **40a**: $R_f$ = 0.35 (silica, diethyl ether/petroleum ether 1:2); IR (film): $\nu_{max}$ = 3412, 3080, 2952, 2857, 1709, 1457, 1250, 1223, 1175, 1095, 860, 838, 740, 694.5 cm$^{-1}$; $^1$H NMR (400 MHz, $CDCl_3$): δ = 7.90 (s, 1 H), 7.59 (d, *J* = 7.8 Hz, 1 H), 7.33 (d, *J* = 8.0 Hz, 1 H), 7.18 (t, *J* = 7.6 Hz, 1 H), 7.12 (t, *J* = 7.4 Hz, 1 H), 6.90 (s, 1 H), 4.93 (s, 1 H), 4.80 (s, 1 H), 4.25–4.12 (m, 2 H), 3.08 (dd, *J* = 14.7, 6.1 Hz, 1 H), 3.05–3.00 (m, 1 H), 2.91 (dd, *J* = 15.5, 10.5 Hz, 1 H), 2.52 (ddd, *J* = 14.6, 4.3, 2.7 Hz, 1 H), 2.46–2.42 (m, 2 H), 2.26 (d, *J* = 10.0 Hz, 1 H), 2.03–1.92 (m, 2 H), 1.78–1.68 (m, 2 H), 1.66–1.63 (m, 1 H), 1.40 (s, 3 H), 1.02 (s, 3 H), 1.03–0.98 (m, 2 H), 0.05 (s, 9 H) ppm; $^{13}$C NMR (126 MHz, $CDCl_3$): δ = 208.93, 173.63, 146.83, 136.16, 127.71, 121.96, 121.93, 119.24, 118.66, 115.61, 111.28,

108.97, 63.69, 57.84, 57.61, 55.07, 40.29, 39.31, 38.21, 37.47, 26.31, 21.42, 20.30, 17.37, 12.73, −1.46 ppm; HRMS (*m/z*): $[M + Na]^+$ calcd for $C_{28}H_{39}N_1O_3SiNa^+$ 488.2591, found 488.2590.

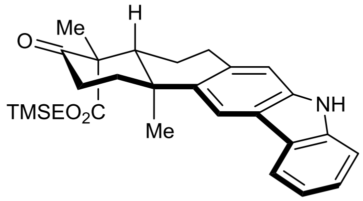


**41**

**Pentacyclic carbazole (trimethylsilyl)ethyl ester 41:** This compound was synthesized from indolyl decalin **40a** (37.6 mg, 0.0807 mmol) by using the procedure for the preparation of xiamycin A (trimethylsilyl)ethyl ester **21**. Pentacyclic carbazole (trimethylsilyl)ethyl ester **41** (24.2 mg, 65 %) was obtained as a white foam. **41**: $R_f$ = 0.39 (silica, diethyl ether/petroleum ether 1:2); IR (film): $\nu_{max}$ = 2955, 1708, 1611, 1466, 1319, 1244, 1098, 910, 858, 838, 734, 589 $cm^{-1}$; $^1H$ NMR (500 MHz, $CDCl_3$): δ = 8.01 (d, *J* = 7.8 Hz, 1 H), 7.99 (s, 1 H), 7.91 (s, 1 H), 7.40–7.33 (m, 2 H), 7.23–7.19 (m, 1 H), 7.07 (s, 1 H), 4.24–4.21 (m, 2 H), 3.22 (ddd, *J* = 14.9, 14.8, 6.3 Hz, 1 H), 3.16 (dd, *J* = 17.0, 4.3 Hz, 1 H), 3.06–2.99 (m, 1 H), 2.81 (ddd, *J* = 13.1, 6.0, 2.2 Hz, 1 H), 2.64 (ddd, *J* = 14.5, 4.2, 2.3 Hz, 1 H), 2.35–2.20 (m, 2 H), 1.95–1.86 (m, 2 H), 1.51 (s, 3 H), 1.46 (s, 3 H), 1.06–1.02 (m, 2 H), 0.06 (s, 9 H) ppm; $^{13}C$ NMR (126 MHz, $CDCl_3$): δ = 208.52, 173.91, 140.21, 138.47, 138.33, 133.57, 125.72, 123.45, 122.33, 120.03, 119.35, 116.87, 110.65, 110.04, 63.78, 57.92, 54.52, 40.11, 38.47, 37.85, 32.76, 23.91, 21.83, 21.15, 17.41, −1.45 ppm; HRMS (*m/z*): $[M + Na]^+$ calcd for $C_{28}H_{35}N_1O_3SiNa^+$ 484.2278, found 484.2272.

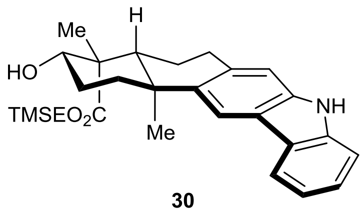


**30**

**Oridamycin A (trimethylsilyl)ethyl ester 30:** To a stirred solution of pentacyclic carbazole (trimethylsilyl)ethyl ester **41** (39.5 mg, 0.0856 mmol) and $CeCl_3$•$7H_2O$ (31.8 mg, 0.0854 mmol) in MeOH (1.5 mL) was added $NaBH_4$ (3.6 mg, 0.095 mmol) at 0 ℃. The reaction mixture was stirred at that temperature for 30 min before it was quenched with aq. HCl (5 mL, 1.0 M). The resulting mixture

was extracted with EtOAc (3 × 5 mL), and the combined organic phases were washed with brine (2 × 5 mL) and dried over anhydrous $Na_2SO_4$. After filtration and removal of the solvent under vacuum, the residue was purified by flash column chromatography with EtOAc/petroleum ether (1:6) to give oridamycin A (trimethylsilyl)ethyl ester **30** (36.4 mg, 92 %) as a white foam.

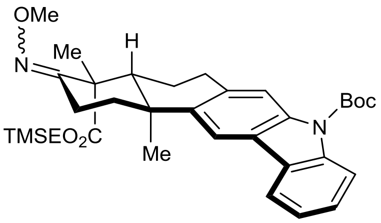


**42a**

***O*-Methyloxime 42a:** To a stirred solution of pentacyclic carbazole (trimethylsilyl)ethyl ester **41** (141 mg, 0.305 mmol) in MeOH/pyridine (3.0 mL, 5:1) was added $NH_2OMe$•HCl (50.5 mg, 0.608 mmol) at 22 ℃. The reaction mixture was stirred at that temperature for 2 h before it was quenched with saturated aq. $NaHCO_3$ (10 mL). The resulting mixture was extracted with EtOAc (3 × 10 mL). The combined organic phases were washed with brine (2 × 5 mL) and dried over anhydrous $MgSO_4$. After filtration and evaporation of the solvent, the residue was passed through a short plug of silica using EtOAc/petroleum ether (1:5) as eluent to give the desired *O*-methyloxime as a colorless oil. This oil was dissolved in THF (2.0 mL). To the solution were sequentially added $Boc_2O$ (133 mg, 0.606 mmol) and 4-DMAP (73.8 mg, 0.606 mmol) at 22 ℃. After stirring at that temperature for 1 h, the reaction mixture was quenched with saturated aq. $NaHCO_3$ (10 mL) and extracted with EtOAc (3 × 10 mL). The combined organic phases were washed with brine (10 mL), dried over anhydrous $MgSO_4$, and filtered. The solvent was evaporated under vacuum. The residue so obtained was purified by flash column chromatography with EtOAc/petroleum ether (1:20) to give *O*-methyloxime **42a** (159 mg, 89 %) as a white powder. **42a**: $R_f$ = 0.45 (silica, EtOAc:petroleum ether 1:20); IR (film): $\nu_{max}$ = 2955, 1726, 1468, 1456, 1358, 1326, 1251, 1227, 1160, 1048, 839, 767, 748 $cm^{-1}$; $^1H$ NMR (400 MHz, $CDCl_3$): δ = 8.24 (d, *J* = 8.3 Hz, 1 H), 8.02 (s, 1 H), 7.92 (d, *J* = 7.1 Hz, 1 H), 7.86 (s, 1 H), 7.44–7.39 (m, 1 H), 7.32 (t, *J* = 7.5 Hz, 1 H), 4.22–4.15 (m, 2 H), 3.88 (s, 3 H), 3.48 (ddd, *J* = 15.0, 4.4, 2.4 Hz, 1 H), 3.16 (dd, *J* = 17.0, 4.0 Hz, 1 H), 3.19–3.13 (m, 1 H), 3.06–2.97 (m, 1 H), 2.57 (ddd, *J* = 12.9, 5.1, 2.3 Hz, 1 H), 2.40

(ddd, $J$ = 14.8, 14.8, 5.3 Hz, 1 H), 2.35–2.28 (m, 1 H), 2.24–2.13 (m, 1 H), 1.79 (d, $J$ = 12.0 Hz, 1 H), 1.76 (s, 9 H), 1.70–1.62 (m, 1 H), 1.59 (s, 3 H), 1.31 (s, 3 H), 1.06–0.99 (m, 2 H), 0.06 (s, 9 H) ppm; $^{13}$C NMR (101 MHz, $CDCl_3$): δ = 175.50, 159.85, 151.31, 142.62, 138.81, 137.11, 135.05, 126.75, 126.12, 124.38, 123.00, 119.32, 116.37, 116.23, 83.79, 63.38, 61.50, 54.19, 50.74, 38.79, 38.66, 33.09, 28.51, 28.03, 23.69, 22.37, 21.51, 20.57, 17.37, −1.41 ppm; HRMS (*m/z*): $[M + Na]^+$ calcd for $C_{34}H_{46}N_2O_5SiNa^+$ 613.3068, found 613.3068.

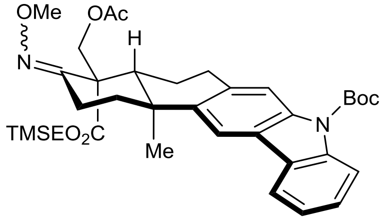


**42**

**Acetoxy *O*-methyloxime 42:** To a stirred solution of *O*-methyloxime **42a** (60.2 mg, 0.102 mmol) in $Ac_2O$/AcOH (1.0 mL, 1:1) were sequentially added $Pd(OAc)_2$ (4.6 mg, 0.020 mmol) and $PhI(OAc)_2$ (32.8 mg, 0.102 mmol) at 22 ℃. The resulting mixture was heated to 110 ℃ and stirred at that temperature for 1 h before it was cooled to 22 ℃ and quenched with saturated aq. $NaHCO_3$ (10 mL). After extraction with EtOAc (3 × 10 mL), the combined organic phases were dried over anhydrous $MgSO_4$ and filtered. The solvent was evaporated under vacuum, and the residue so obtained was subjected to flash column chromatography using EtOAc/petroleum ether (1:8) as eluent to give acetoxy *O*-methyloxime **42** (53.6 mg, 81 %) as a white powder. **42**: $R_f$ = 0.44 (silica, EtOAc:petroleum ether 1:6); IR (film): $\nu_{max}$ = 3431, 2954, 2817, 2253, 1731, 1601, 1469, 910, 839, 768 cm$^{-1}$; $^1$H NMR (400 MHz, $CDCl_3$): δ = 8.24 (d, $J$ = 8.3 Hz, 1 H), 8.02 (s, 1 H), 7.93 (d, $J$ = 7.5 Hz, 1 H), 7.88 (s, 1 H), 7.42 (t, $J$ = 7.3 Hz, 1 H), 7.32 (t, $J$ = 7.5 Hz, 1 H), 4.67 (d, $J$ = 10.7 Hz, 1 H), 4.56 (d, $J$ = 10.7 Hz, 1 H), 4.27–4.20 (m, 2 H), 3.84 (s, 3 H), 3.32–3.24 (m, 1 H), 3.15 (dd, $J$ = 17.0, 4.3 Hz, 1 H), 3.09–2.99 (m, 1 H), 2.61–2.52 (m, 2 H), 2.20–2.11 (m, 2 H), 2.10–2.03 (m, 1 H), 2.02 (s, 3 H), 1.75 (s, 9 H), 1.68–1.63 (m, 1 H), 1.34 (s, 3 H), 1.07–1.02 (m, 2 H), 0.06 (s, 9 H) ppm; $^{13}$C NMR (126 MHz, $CDCl_3$): δ = 172.16, 170.72, 156.20, 151.28, 142.30, 138.84, 137.16, 134.70, 126.85, 126.02, 124.40, 123.04, 119.35, 116.39, 116.37, 116.14, 83.89, 77.16, 65.79, 63.87, 61.88, 54.42, 46.90, 38.03, 36.88, 32.44, 28.51, 23.74, 21.28,

21.09, 20.83, 17.46, –1.43 ppm; HRMS (*m/z*): $[M + Na]^+$ calcd for $C_{36}H_{48}N_2O_7SiNa^+$ 671.3123, found 671.3128.

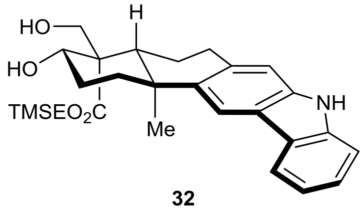


**32**

**Oridamycin B (trimethylsilyl)ethyl ester 32:** To a stirred solution of acetoxy *O*-methyloxime **42** (42.4 mg, 0.0654 mmol) in acetone (0.90 mL) was added aq. $HClO_4$ (0.30 mL, 6.0 M) at 22 °C. The reaction mixture was vigorously stirred at that temperature for 8 h before it was quenched with saturated aq. $NaHCO_3$ (10 mL). The resulting mixture was extracted with $CH_2Cl_2$ (3 × 10 mL). The combined organic phases were sequentially washed with saturated aq. $NaHCO_3$ (10 mL) and brine (10 mL) and dried over anhydrous $Na_2SO_4$. After filtration and evaporation under vacuum, the residue was immediately dissolved in MeOH (0.50 mL). To this solution was added $NaBH_4$ (2.7 mg, 0.071 mmol) at 0 °C. After stirring at that temperature for 30 min, the reaction mixture was quenched with aq. HCl (2 mL, 1.0 M). The resulting mixture was extracted with EtOAc (3 × 5 mL). The combined organic phases were sequentially washed with saturated aq. $NaHCO_3$ (2 mL) and brine (2 mL), dried over anhydrous $Na_2SO_4$, and filtered. The solvent was removed under vacuum, and the residue so obtained was purified by flash column chromatography with EtOAc/petroleum ether (1:2) to give oridamycin B (trimethylsilyl)ethyl ester **32** (16.9 mg, 54 % for the two steps) as a white foam.

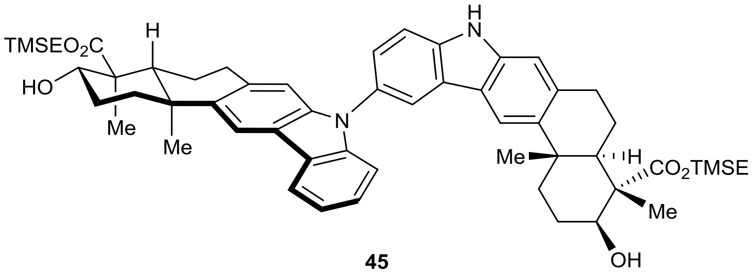


**45**

**Dixiamycin C bis[2-(trimethylsilyl)ethyl] ester 45:** To a solution of *N*-benzenesulfonyl 6-bromoxiamycin A (trimethylsilyl)ethyl ester **19** (29.7 mg, 0.0435 mmol) and xiamycin A (trimethylsilyl)ethyl ester **21** (13.6 mg, 0.0293 mmol) in 1,4-dioxane (0.25 mL) were sequentially added

(±)-*trans*-1,2-diaminocyclohexane **43** (10.1 mg, 0.0879 mmol), $K_3PO_4$ (46.7 mg, 0.219 mmol), and CuI (8.4 mg, 0.044 mmol) at 22 °C. The resulting mixture was heated to 110 °C and stirred at that temperature for 12 h before it was cooled to 22 °C. The volatile was removed under vacuum, and the residue was directly subjected to flash column chromatography for purification using EtOAc/petroleum ether (1:2) as eluent to give the desired coupling product **44** as a pale yellow powder. This powder was dissolved in MeOH (1.0 mL). To this solution was added magnesium powder (4.2 mg, 0.17 mmol) at 22 °C. The resulting mixture was sonicated at that temperature for 1 h before it was quenched with saturated aq. $NH_4Cl$ (3 mL). The resulting mixture was extracted with EtOAc (3 × 5 mL), and the combined organic phases were washed with brine (3 mL) and dried over anhydrous $MgSO_4$. After filtration and evaporation of the solvent under vacuum, the residue was purified by flash column chromatography with EtOAc/petroleum ether (1:2) to give dixiamycin C bis[2-(trimethylsilyl)ethyl] esters **45** (13.8 mg, 51 % for the two steps) as a pale yellow foam. **45**: $R_f$ = 0.28 (silica, EtOAc:petroleum ether 1:2); $[\alpha]_D^{24}$ = +118.5 (*c* = 0.45 in $CHCl_3$); IR (film): $\nu_{max}$ = 3410, 2926, 2853, 1712, 1495, 1467, 1067, 858, 837, 746 $cm^{-1}$; $^1H$ NMR (600 MHz, $CDCl_3$): δ = 8.11 (d, *J* = 7.9 Hz, 1 H), 8.10 (s, 1 H), 8.05 (s, 2 H), 7.89 (s, 1 H), 7.54 (d, *J* = 8.4 Hz, 1 H), 7.46 (d, *J* = 8.1 Hz, 1 H), 7.35 (t, *J* = 7.6 Hz, 1 H), 7.30 (d, *J* = 7.9 Hz, 1 H), 7.24 (d, *J* = 7.1 Hz, 1 H), 7.15 (s, 1 H), 7.01 (s, 1 H), 4.28–4.16 (m, 2 H), 4.12–4.04 (m, 2 H), 3.17–3.11 (m, 2 H), 3.04–3.01 (m, 2 H), 2.64 (d, *J* = 12.2 Hz, 1 H), 2.49 (d, *J* = 12.8 Hz, 1 H), 2.28–2.20 (m, 2 H), 2.06–1.98 (m, 3 H), 1.96–1.85 (m, 4 H), 1.82–1.74 (m, 4 H), 1.58–1.49 (m, 3 H), 1.35 (s, 3 H), 1.30 (s, 3 H), 1.29 (s, 6 H), 1.05–0.97 (m, 4 H), 0.05 (s, 9 H), 0.03 (s, 9 H) ppm; $^{13}C$ NMR (151 MHz, $CDCl_3$): δ = 177.56, 142.56, 141.87, 141.44, 140.83, 139.19, 138.99, 134.49, 133.62, 129.55, 125.56, 125.18, 124.79, 123.42, 121.79, 119.97, 119.41, 119.34, 116.06, 115.81, 111.50, 110.33, 109.82, 109.14, 75.40, 75.35, 63.50, 63.45, 53.66, 53.63, 45.87, 45.80, 37.65, 37.47, 37.45, 31.12, 31.06, 27.56, 27.46, 26.04, 26.00, 21.67, 21.57, 17.67, 10.91, −1.37, −1.39 ppm; HRMS (*m/z*): $[M]^+$ calcd for $C_{56}H_{72}N_2O_6Si_2^+$ 924.4923, found 924.4922.

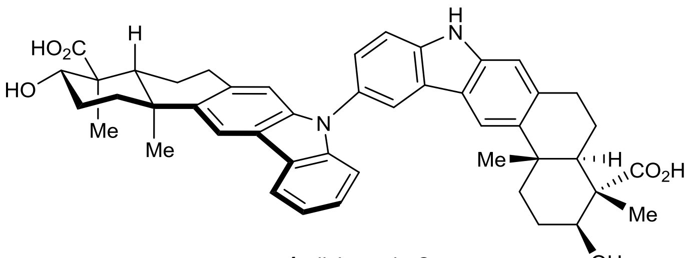


**4:** dixiamycin C

**Dixiamycin C (4):** To a stirred solution of dixiamycin C bis[2-(trimethylsilyl)ethyl] esters **45** (13.3 mg, 0.0144 mmol) in DMF (0.20 mL) was added TASF (15.8 mg, 0.0576 mmol) at 22 ℃. The resulting mixture was warmed to 50 ℃ and stirred at that temperature for 5 h before it was quenched with aq. HCl (2 mL, 1.0 M) and extracted with EtOAc (5 × 3 mL). The combined organic phases were washed with brine (2 mL), dried over anhydrous $Na_2SO_4$, and filtered. The solvent was evaporated under vacuum. The residue so obtained was subjected to HPLC (TCI, Kasiesorb LC ODS 2000 column, 10 mm × 250 mm) using acetonitrile/water (50:50, 5 mL/min) as eluent to give dixiamycin C (**4**, $t_R$ = 21.15 min). After removal of the solvent under vacuum, **4** (10.0 mg, 96 %) was obtained as a white powder. **4**: $R_f$ = 0.45 (silica, MeOH:$CH_2Cl_2$ 1:5); $[\alpha]_D^{25}$ = +121.5 (*c* = 0.05 in MeOH); IR (film): $\nu_{max}$ = 3671, 2966, 2864, 1713, 1455, 1384, 1238, 1056, 1032, 1013 $cm^{-1}$; $^1H$ NMR (600 MHz, methanol-$d_4$): δ = 8.10 (s, 1 H), 8.10 (d, *J* = 4.5 Hz, 1 H), 8.07 (s, 1 H), 7.98 (s, 1 H), 7.58 (d, *J* = 8.4 Hz, 1 H), 7.39 (dd, *J* = 8.4, 1.9 Hz, 1 H), 7.31 (t, *J* = 7.5 Hz, 1 H), 7.24 (d, *J* = 8.1 Hz, 1 H), 7.19 (t, *J* = 7.4 Hz, 1 H), 7.16 (s, 1 H), 6.96 (s, 1 H), 4.13–4.06 (m, 2 H), 3.15 (dd, *J* = 16.8, 6.2 Hz, 1 H), 3.08 (dd, *J* = 17.5, 9.9 Hz, 1 H), 3.04–3.00 (m, 1 H), 3.00–2.93 (m, 1 H), 2.69 (d, *J* = 13.0 Hz, 1 H), 2.59 (d, *J* = 13.4 Hz, 1 H), 2.17 (dd, *J* = 12.5, 3.3 Hz, 2 H), 2.07–1.98 (m, 2 H), 1.94–1.91 (m, 2 H), 1.88–1.82 (m, 2 H), 1.82–1.75 (m, 1 H) 1.75–1.69 (m, 1 H), 1.60–1.49 (m, 2 H) 1.34 (s, 3 H), 1.29 (s, 3 H), 1.25 (s, 3 H), 1.24 (s, 3 H) ppm; $^{13}C$ NMR (151 MHz, methanol-$d_4$): 181.32, 143.80, 142.67, 142.40, 142.09, 141.11, 140.91, 134.99, 134.60, 129.89, 126.40, 125.68, 125.54, 124.63, 122.95, 122.81, 120.70, 120.23, 119.79, 116.81, 116.57, 112.48, 111.20, 110.57, 109.96, 76.29, 54.87, 47.83, 47.81, 39.00, 38.85, 38.38, 38.33, 32.02, 28.63, 28.55, 26.30, 26.19, 22.51, 11.39 ppm; HRMS (*m/z*): $[M + Na]^+$ calcd for $C_{46}H_{48}N_2O_6Na^+$ 747.3405, found 747.3392.

## II Antiviral Assay

For anti-HSV-1 assay, vero cells were cultured in Dulbecco′s modified Eagle′s medium (DMEM, Invitrogen, Carlsbad, CA, USA) supplemented with 10 % fetal bovine serum, 2 mM L-glutamine, 100 U/mL penicillin, and 100 mg/mL streptomycin. The cells were incubated at 37 ℃ with 5 % $CO_2$. The vero cells were seeded into 48-well plates at a density of $3 \times 10^4$ per well. The cells were infected with HSV-1 at a multiplicity of infection of 1 for 4 h. The HSV-1 inocula were then removed, and the cells were replenished with fresh media containing 5.0 $\mu$M and 0.50 $\mu$M of the tested compounds dissolved in DMSO. After 20 h incubation at 37 ℃, the titers of HSV-1 in the culture supernatants were measured by $TCID_{50}$ assay[9] and expressed as a percentage of the mock DMSO treatment.

A dual-luciferase reporter system was used to evaluate the anti-HCV activity. First, an Huh7 cell line harboring JFH1 subgenomic replicon with Renilla reporter gene inserted in the NS5A domain III region was generated as previously reported,[10] named JFH1-NS5A-Rluc cell. The firefly reporter gene was then introduced as internal control by a lenti-virus vector, generating JFH1-NS5A-Rluc/Huh7-luc cell line. To evaluate the anti-HCV activity of the compounds, JFH1-NS5A-Rluc/Huh7-luc cells were seeded into 96-well plate at a density of $1 \times 10^4$ cells per well one day before treated with indicated concentration of tested compounds. Cell culture supernatants were removed 72 hours later, and the cells were washed once with PBS and then lysed with 25 $\mu$L 1X passive lysis assay buffer. Firefly and Renilla luciferase activities were measured using Dual-Luciferase® Reporter Assay system (Promega, E1960) following the manufacturer′s instructions.

## III Cytotoxicity Assay

Vero or Huh-7 cells were seeded into 96-well plate at a density of $1 \times 10^4$ cells per well 24 h before treated with 50 $\mu$M, 5.0 $\mu$M of the tested compounds or DMSO control for additional 24 h. Cell viability was measured by Cell-Titer-Glo® Luminescent Cell Viability assay (Promega, G7572) according to the manufacturer′s instructions. It should be noted that $1 \times 10^4$ cells falls into the linear range of cell viability readout. Data were presented as average of triplicates and normalized to the respective DMSO-treated control cells.

## IV Crystallographic Data of Compounds 1, 6, and 40

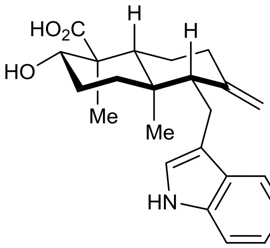


**6:** indosespene

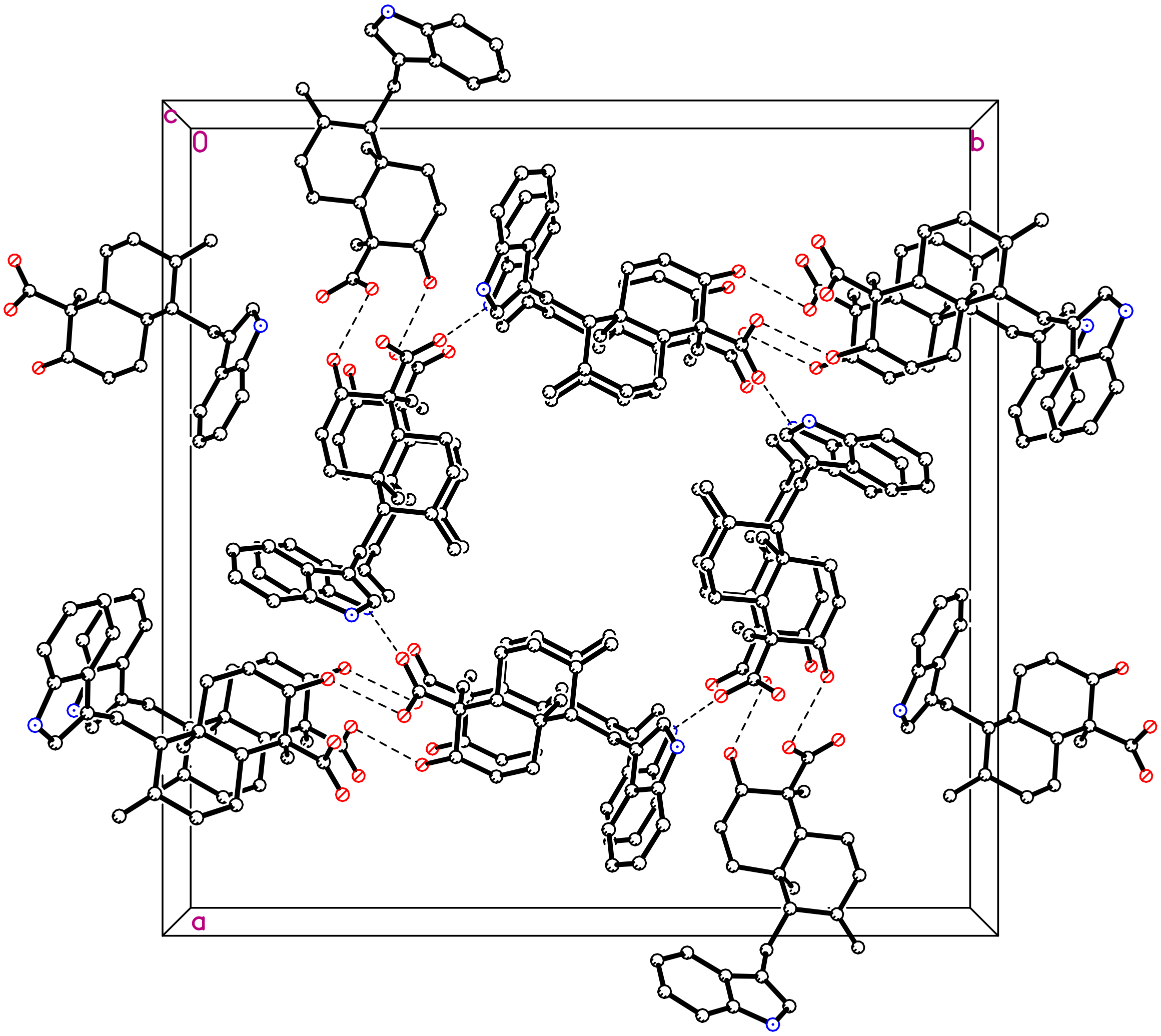

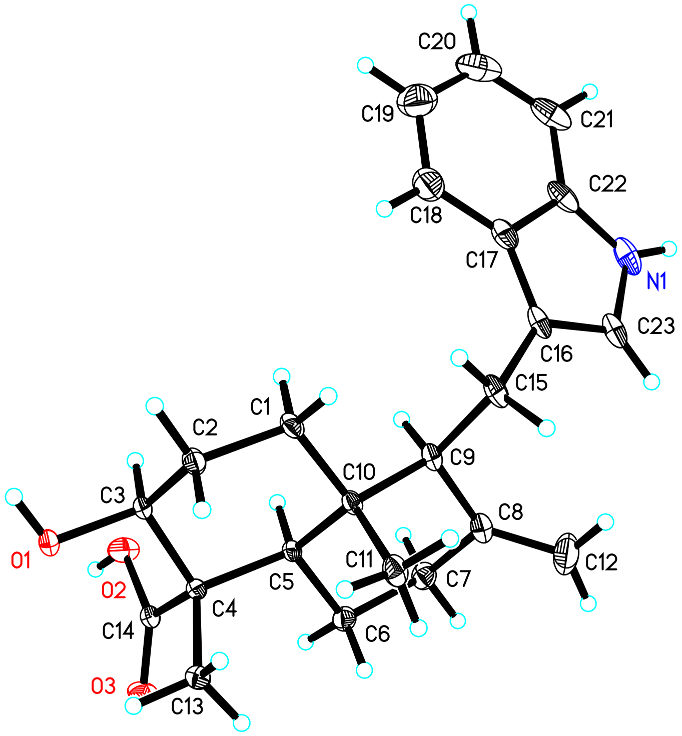


ORTEP of Compound **6**

*Crystal data*

| | |
|---|---|
| $C_{23}H_{29}NO_3$ | $D_x$ = 1.129 Mg m$^{-3}$ |
| $M_r$ = 367.47 | Mo *K*α radiation, λ = 0.71073 Å |
| Tetragonal, *I*4 | Cell parameters from 2624 reflections |
| *a* = 25.728 (6) Å | θ = 2.5–23.0 ° |
| *c* = 6.5313 (16) Å | μ = 0.07 mm$^{-1}$ |
| *V* = 4323 (2) Å$^3$ | *T* = 140 K |
| *Z* = 8 | Prism, colourless |
| *F*(000) = 1584 | 0.35 × 0.06 × 0.05 mm |

*Data collection*

| | |
|---|---|
| Bruker APEX-II CCD diffractometer | $R_{int}$ = 0.073 |
| φ and ω scans | $\theta_{max}$ = 30.5°, $\theta_{min}$ = 1.1 ° |
| 21553 measured reflections | *h* = −32→36 |
| 6551 independent reflections | *k* = −36→36 |
| 4573 reflections with *I* > 2σ(*I*) | *l* = −9→9 |

*Refinement*

| | |
|---|---|
| Refinement on $F^2$ | Hydrogen site location: inferred from neighbouring sites |
| Least-squares matrix: full | H-atom parameters constrained |
| $R[F^2 > 2\sigma(F^2)]$ = 0.054 | $w = 1/[\sigma^2(F_o^2) + (0.0544P)^2]$ |

| | |
|---|---|
| | where $P = (F_o^2 + 2F_c^2)/3$ |
| $wR(F^2) = 0.120$ | $(\Delta/\sigma)_{max} < 0.001$ |
| $S = 0.93$ | $\Delta\rho_{max} = 0.26$ e Å$^{-3}$ |
| 6551 reflections | $\Delta\rho_{min} = -0.25$ e Å$^{-3}$ |
| 248 parameters | Absolute structure: Flack x determined using 1542 quotients [(I+)-(I-)]/[(I+)+(I-)] (Parsons and Flack (2004), Acta Cryst. A60, s61). |
| 1 restraint | Flack parameter: −1.6 (9) |

*Special details*

**Geometry**. All esds (except the esd in the dihedral angle between two l.s. planes) are estimated using the full covariance matrix. The cell esds are taken into account individually in the estimation of esds in distances, angles and torsion angles; correlations between esds in cell parameters are only used when they are defined by crystal symmetry. An approximate (isotropic) treatment of cell esds is used for estimating esds involving l.s. planes.

*Fractional atomic coordinates and isotropic or equivalent isotropic displacement parameters (Å$^2$)*

| | *x* | *y* | *z* | $U_{iso}$*/$U_{eq}$ |
|---|---|---|---|---|
| N1 | 0.61305 (8) | 0.23296 (10) | 1.3420 (4) | 0.0379 (6) |
| H1N | 0.6333 | 0.2468 | 1.4360 | 0.045* |
| O1 | 0.30325 (6) | 0.19315 (6) | 0.4588 (3) | 0.0269 (4) |
| H1 | 0.2978 | 0.1610 | 0.4623 | 0.040* |
| O2 | 0.28710 (6) | 0.26105 (7) | 0.8657 (3) | 0.0321 (5) |
| H2 | 0.2586 | 0.2730 | 0.9067 | 0.048* |
| O3 | 0.28163 (6) | 0.32740 (6) | 0.6498 (3) | 0.0297 (4) |
| C1 | 0.44179 (8) | 0.19337 (9) | 0.6564 (4) | 0.0221 (5) |
| H1A | 0.4737 | 0.1771 | 0.6010 | 0.026* |
| H1B | 0.4353 | 0.1784 | 0.7936 | 0.026* |
| C2 | 0.39603 (9) | 0.17990 (9) | 0.5165 (4) | 0.0235 (5) |
| H2A | 0.4037 | 0.1916 | 0.3753 | 0.028* |
| H2B | 0.3911 | 0.1417 | 0.5137 | 0.028* |
| C3 | 0.34655 (8) | 0.20568 (8) | 0.5906 (4) | 0.0201 (5) |
| H3 | 0.3387 | 0.1928 | 0.7317 | 0.024* |
| C4 | 0.35114 (8) | 0.26497 (8) | 0.5973 (4) | 0.0176 (5) |
| C5 | 0.39832 (8) | 0.27805 (8) | 0.7401 (4) | 0.0171 (5) |
| H5 | 0.3892 | 0.2624 | 0.8757 | 0.020* |
| C6 | 0.40369 (9) | 0.33630 (9) | 0.7813 (4) | 0.0240 (5) |
| H6A | 0.3697 | 0.3505 | 0.8248 | 0.029* |
| H6B | 0.4140 | 0.3542 | 0.6534 | 0.029* |
| C7 | 0.44437 (10) | 0.34722 (10) | 0.9483 (5) | 0.0282 (6) |
| H7A | 0.4503 | 0.3851 | 0.9593 | 0.034* |
| H7B | 0.4314 | 0.3346 | 1.0820 | 0.034* |
| C8 | 0.49474 (9) | 0.32042 (10) | 0.8974 (4) | 0.0286 (6) |

| | | | | |
|---|---|---|---|---|
| C9 | 0.48891 (8) | 0.26258 (9) | 0.8633 (4) | 0.0213 (5) |
| H9 | 0.4711 | 0.2487 | 0.9879 | 0.026* |
| C10 | 0.45102 (8) | 0.25204 (9) | 0.6788 (4) | 0.0185 (5) |
| C11 | 0.47550 (9) | 0.27356 (11) | 0.4806 (4) | 0.0266 (6) |
| H11A | 0.5122 | 0.2634 | 0.4741 | 0.040* |
| H11B | 0.4728 | 0.3116 | 0.4799 | 0.040* |
| H11C | 0.4570 | 0.2594 | 0.3617 | 0.040* |
| C12 | 0.53749 (12) | 0.34756 (14) | 0.8618 (8) | 0.0612 (11) |
| H12A | 0.5366 | 0.3844 | 0.8682 | 0.073* |
| H12B | 0.5690 | 0.3302 | 0.8298 | 0.073* |
| C13 | 0.35350 (9) | 0.28895 (9) | 0.3825 (4) | 0.0236 (5) |
| H13A | 0.3802 | 0.2711 | 0.3015 | 0.035* |
| H13B | 0.3622 | 0.3259 | 0.3933 | 0.035* |
| H13C | 0.3196 | 0.2851 | 0.3154 | 0.035* |
| C14 | 0.30291 (8) | 0.28772 (9) | 0.7033 (4) | 0.0205 (5) |
| C15 | 0.54123 (9) | 0.23333 (11) | 0.8476 (4) | 0.0285 (6) |
| H15A | 0.5353 | 0.1994 | 0.7798 | 0.034* |
| H15B | 0.5652 | 0.2537 | 0.7604 | 0.034* |
| C16 | 0.56655 (9) | 0.22404 (11) | 1.0518 (4) | 0.0277 (6) |
| C17 | 0.56079 (9) | 0.17782 (10) | 1.1727 (5) | 0.0305 (6) |
| C18 | 0.53351 (11) | 0.13103 (12) | 1.1451 (6) | 0.0424 (7) |
| H18 | 0.5128 | 0.1255 | 1.0265 | 0.051* |
| C19 | 0.53745 (15) | 0.09335 (14) | 1.2941 (7) | 0.0578 (10) |
| H19 | 0.5191 | 0.0616 | 1.2779 | 0.069* |
| C20 | 0.56809 (15) | 0.10096 (15) | 1.4699 (6) | 0.0593 (11) |
| H20 | 0.5699 | 0.0742 | 1.5700 | 0.071* |
| C21 | 0.59546 (13) | 0.14614 (14) | 1.5002 (6) | 0.0486 (9) |
| H21 | 0.6166 | 0.1509 | 1.6179 | 0.058* |
| C22 | 0.59091 (10) | 0.18467 (12) | 1.3515 (5) | 0.0346 (7) |
| C23 | 0.59852 (9) | 0.25619 (11) | 1.1616 (5) | 0.0326 (6) |
| H23A | 0.6092 | 0.2899 | 1.1190 | 0.039* |

*Atomic displacement parameters (Å$^2$)*

| | $U^{11}$ | $U^{22}$ | $U^{33}$ | $U^{12}$ | $U^{13}$ | $U^{23}$ |
|---|---|---|---|---|---|---|
| N1 | 0.0232 (11) | 0.0575 (16) | 0.0330 (14) | 0.0067 (10) | −0.0087 (10) | −0.0185 (13) |
| O1 | 0.0194 (8) | 0.0229 (8) | 0.0384 (12) | −0.0036 (6) | −0.0110 (8) | 0.0018 (8) |
| O2 | 0.0224 (8) | 0.0348 (10) | 0.0392 (12) | 0.0086 (7) | 0.0151 (9) | 0.0129 (9) |
| O3 | 0.0255 (9) | 0.0274 (9) | 0.0362 (11) | 0.0111 (7) | 0.0021 (8) | 0.0086 (9) |
| C1 | 0.0155 (10) | 0.0233 (11) | 0.0274 (13) | 0.0053 (8) | −0.0035 (10) | −0.0027 (11) |
| C2 | 0.0196 (11) | 0.0206 (11) | 0.0305 (15) | 0.0018 (9) | −0.0064 (10) | −0.0052 (10) |
| C3 | 0.0133 (10) | 0.0194 (10) | 0.0277 (13) | −0.0014 (8) | −0.0055 (9) | 0.0026 (10) |
| C4 | 0.0136 (10) | 0.0180 (10) | 0.0211 (12) | 0.0006 (8) | −0.0011 (9) | 0.0031 (9) |

| | | | | | | |
|---|---|---|---|---|---|---|
| C5 | 0.0122 (10) | 0.0193 (11) | 0.0196 (12) | 0.0002 (8) | 0.0006 (9) | 0.0024 (9) |
| C6 | 0.0212 (11) | 0.0192 (11) | 0.0317 (15) | 0.0004 (9) | −0.0001 (10) | −0.0013 (10) |
| C7 | 0.0288 (13) | 0.0221 (12) | 0.0337 (16) | −0.0026 (9) | −0.0031 (12) | −0.0058 (11) |
| C8 | 0.0202 (11) | 0.0351 (14) | 0.0305 (15) | −0.0028 (10) | −0.0038 (11) | −0.0021 (12) |
| C9 | 0.0142 (10) | 0.0299 (12) | 0.0197 (12) | −0.0004 (8) | −0.0011 (10) | −0.0024 (10) |
| C10 | 0.0124 (9) | 0.0246 (11) | 0.0186 (12) | 0.0009 (8) | −0.0003 (9) | 0.0008 (10) |
| C11 | 0.0173 (11) | 0.0407 (15) | 0.0219 (14) | −0.0007 (10) | 0.0041 (10) | 0.0005 (12) |
| C12 | 0.0315 (16) | 0.0463 (19) | 0.106 (3) | −0.0129 (13) | −0.002 (2) | −0.001 (2) |
| C13 | 0.0217 (11) | 0.0256 (12) | 0.0234 (14) | −0.0006 (9) | −0.0024 (10) | 0.0048 (11) |
| C14 | 0.0140 (10) | 0.0217 (11) | 0.0257 (14) | −0.0004 (8) | −0.0018 (9) | 0.0026 (10) |
| C15 | 0.0148 (10) | 0.0450 (15) | 0.0257 (14) | 0.0058 (10) | −0.0001 (10) | −0.0050 (12) |
| C16 | 0.0138 (10) | 0.0412 (15) | 0.0282 (15) | 0.0057 (9) | −0.0033 (10) | −0.0094 (12) |
| C17 | 0.0204 (11) | 0.0375 (14) | 0.0336 (15) | 0.0087 (10) | −0.0021 (12) | −0.0078 (13) |
| C18 | 0.0356 (15) | 0.0426 (16) | 0.049 (2) | 0.0032 (12) | −0.0044 (15) | −0.0040 (16) |
| C19 | 0.059 (2) | 0.0427 (19) | 0.072 (3) | 0.0066 (16) | 0.007 (2) | 0.0063 (19) |
| C20 | 0.064 (2) | 0.056 (2) | 0.058 (3) | 0.0278 (18) | 0.009 (2) | 0.019 (2) |
| C21 | 0.0435 (18) | 0.066 (2) | 0.0363 (18) | 0.0311 (16) | −0.0014 (15) | −0.0012 (17) |
| C22 | 0.0237 (12) | 0.0518 (17) | 0.0284 (16) | 0.0180 (12) | −0.0014 (12) | −0.0066 (14) |
| C23 | 0.0170 (11) | 0.0465 (16) | 0.0344 (17) | 0.0053 (10) | −0.0048 (12) | −0.0112 (14) |

*Geometric parameters (Å, º)*

| | | | |
|---|---|---|---|
| N1—C22 | 1.368 (4) | C8—C9 | 1.512 (3) |
| N1—C23 | 1.373 (4) | C9—C15 | 1.546 (3) |
| N1—H1N | 0.8800 | C9—C10 | 1.574 (3) |
| O1—C3 | 1.445 (3) | C9—H9 | 1.0000 |
| O1—H1 | 0.8400 | C10—C11 | 1.542 (3) |
| O2—C14 | 1.327 (3) | C11—H11A | 0.9800 |
| O2—H2 | 0.8400 | C11—H11B | 0.9800 |
| O3—C14 | 1.210 (3) | C11—H11C | 0.9800 |
| C1—C2 | 1.530 (3) | C12—H12A | 0.9500 |
| C1—C10 | 1.535 (3) | C12—H12B | 0.9500 |
| C1—H1A | 0.9900 | C13—H13A | 0.9800 |
| C1—H1B | 0.9900 | C13—H13B | 0.9800 |
| C2—C3 | 1.515 (3) | C13—H13C | 0.9800 |
| C2—H2A | 0.9900 | C15—C16 | 1.503 (4) |
| C2—H2B | 0.9900 | C15—H15A | 0.9900 |
| C3—C4 | 1.531 (3) | C15—H15B | 0.9900 |
| C3—H3 | 1.0000 | C16—C23 | 1.369 (4) |
| C4—C13 | 1.534 (3) | C16—C17 | 1.435 (4) |
| C4—C14 | 1.537 (3) | C17—C18 | 1.405 (4) |
| C4—C5 | 1.567 (3) | C17—C22 | 1.412 (4) |
| C5—C6 | 1.529 (3) | C18—C19 | 1.378 (5) |

| | | | |
|---|---|---|---|
| C5—C10 | 1.564 (3) | C18—H18 | 0.9500 |
| C5—H5 | 1.0000 | C19—C20 | 1.406 (6) |
| C6—C7 | 1.538 (4) | C19—H19 | 0.9500 |
| C6—H6A | 0.9900 | C20—C21 | 1.374 (6) |
| C6—H6B | 0.9900 | C20—H20 | 0.9500 |
| C7—C8 | 1.505 (4) | C21—C22 | 1.393 (5) |
| C7—H7A | 0.9900 | C21—H21 | 0.9500 |
| C7—H7B | 0.9900 | C23—H23A | 0.9500 |
| C8—C12 | 1.323 (4) | | |

| | | | |
|---|---|---|---|
| C22—N1—C23 | 108.7 (2) | C10—C9—H9 | 106.2 |
| C22—N1—H1N | 125.6 | C1—C10—C11 | 109.6 (2) |
| C23—N1—H1N | 125.6 | C1—C10—C5 | 108.12 (17) |
| C3—O1—H1 | 109.5 | C11—C10—C5 | 114.56 (19) |
| C14—O2—H2 | 109.5 | C1—C10—C9 | 109.76 (19) |
| C2—C1—C10 | 113.48 (18) | C11—C10—C9 | 109.15 (18) |
| C2—C1—H1A | 108.9 | C5—C10—C9 | 105.48 (18) |
| C10—C1—H1A | 108.9 | C10—C11—H11A | 109.5 |
| C2—C1—H1B | 108.9 | C10—C11—H11B | 109.5 |
| C10—C1—H1B | 108.9 | H11A—C11—H11B | 109.5 |
| H1A—C1—H1B | 107.7 | C10—C11—H11C | 109.5 |
| C3—C2—C1 | 110.9 (2) | H11A—C11—H11C | 109.5 |
| C3—C2—H2A | 109.5 | H11B—C11—H11C | 109.5 |
| C1—C2—H2A | 109.5 | C8—C12—H12A | 120.0 |
| C3—C2—H2B | 109.5 | C8—C12—H12B | 120.0 |
| C1—C2—H2B | 109.5 | H12A—C12—H12B | 120.0 |
| H2A—C2—H2B | 108.0 | C4—C13—H13A | 109.5 |
| O1—C3—C2 | 111.1 (2) | C4—C13—H13B | 109.5 |
| O1—C3—C4 | 107.39 (17) | H13A—C13—H13B | 109.5 |
| C2—C3—C4 | 112.37 (18) | C4—C13—H13C | 109.5 |
| O1—C3—H3 | 108.6 | H13A—C13—H13C | 109.5 |
| C2—C3—H3 | 108.6 | H13B—C13—H13C | 109.5 |
| C4—C3—H3 | 108.6 | O3—C14—O2 | 121.9 (2) |
| C3—C4—C13 | 112.2 (2) | O3—C14—C4 | 123.8 (2) |
| C3—C4—C14 | 109.27 (17) | O2—C14—C4 | 114.24 (19) |
| C13—C4—C14 | 106.90 (18) | C16—C15—C9 | 113.3 (2) |
| C3—C4—C5 | 106.89 (17) | C16—C15—H15A | 108.9 |
| C13—C4—C5 | 115.31 (18) | C9—C15—H15A | 108.9 |
| C14—C4—C5 | 105.98 (19) | C16—C15—H15B | 108.9 |
| C6—C5—C10 | 112.70 (18) | C9—C15—H15B | 108.9 |
| C6—C5—C4 | 112.65 (18) | H15A—C15—H15B | 107.7 |
| C10—C5—C4 | 115.27 (19) | C23—C16—C17 | 105.9 (3) |
| C6—C5—H5 | 105.0 | C23—C16—C15 | 129.0 (3) |

| | | | |
|---|---|---|---|
| C10—C5—H5 | 105.0 | C17—C16—C15 | 125.1 (2) |
| C4—C5—H5 | 105.0 | C18—C17—C22 | 119.2 (3) |
| C5—C6—C7 | 111.4 (2) | C18—C17—C16 | 133.7 (3) |
| C5—C6—H6A | 109.3 | C22—C17—C16 | 107.1 (2) |
| C7—C6—H6A | 109.3 | C19—C18—C17 | 118.4 (3) |
| C5—C6—H6B | 109.3 | C19—C18—H18 | 120.8 |
| C7—C6—H6B | 109.3 | C17—C18—H18 | 120.8 |
| H6A—C6—H6B | 108.0 | C18—C19—C20 | 121.3 (3) |
| C8—C7—C6 | 110.2 (2) | C18—C19—H19 | 119.3 |
| C8—C7—H7A | 109.6 | C20—C19—H19 | 119.3 |
| C6—C7—H7A | 109.6 | C21—C20—C19 | 121.5 (3) |
| C8—C7—H7B | 109.6 | C21—C20—H20 | 119.2 |
| C6—C7—H7B | 109.6 | C19—C20—H20 | 119.2 |
| H7A—C7—H7B | 108.1 | C20—C21—C22 | 117.3 (3) |
| C12—C8—C7 | 120.8 (3) | C20—C21—H21 | 121.4 |
| C12—C8—C9 | 125.2 (3) | C22—C21—H21 | 121.4 |
| C7—C8—C9 | 113.44 (19) | N1—C22—C21 | 130.0 (3) |
| C8—C9—C15 | 113.74 (19) | N1—C22—C17 | 107.7 (3) |
| C8—C9—C10 | 110.11 (19) | C21—C22—C17 | 122.3 (3) |
| C15—C9—C10 | 113.9 (2) | C16—C23—N1 | 110.5 (3) |
| C8—C9—H9 | 106.2 | C16—C23—H23A | 124.8 |
| C15—C9—H9 | 106.2 | N1—C23—H23A | 124.8 |

| | | | |
|---|---|---|---|
| C10—C1—C2—C3 | −56.5 (3) | C8—C9—C10—C11 | 64.4 (2) |
| C1—C2—C3—O1 | 179.91 (19) | C15—C9—C10—C11 | −64.7 (3) |
| C1—C2—C3—C4 | 59.6 (3) | C8—C9—C10—C5 | −59.1 (2) |
| O1—C3—C4—C13 | −52.5 (2) | C15—C9—C10—C5 | 171.8 (2) |
| C2—C3—C4—C13 | 70.0 (2) | C3—C4—C14—O3 | −141.5 (2) |
| O1—C3—C4—C14 | 65.9 (2) | C13—C4—C14—O3 | −19.9 (3) |
| C2—C3—C4—C14 | −171.6 (2) | C5—C4—C14—O3 | 103.6 (3) |
| O1—C3—C4—C5 | −179.84 (18) | C3—C4—C14—O2 | 40.8 (3) |
| C2—C3—C4—C5 | −57.4 (3) | C13—C4—C14—O2 | 162.4 (2) |
| C3—C4—C5—C6 | −173.3 (2) | C5—C4—C14—O2 | −74.1 (2) |
| C13—C4—C5—C6 | 61.2 (3) | C8—C9—C15—C16 | 77.9 (3) |
| C14—C4—C5—C6 | −56.8 (2) | C10—C9—C15—C16 | −154.9 (2) |
| C3—C4—C5—C10 | 55.5 (2) | C9—C15—C16—C23 | −85.6 (3) |
| C13—C4—C5—C10 | −70.0 (2) | C9—C15—C16—C17 | 93.8 (3) |
| C14—C4—C5—C10 | 171.93 (18) | C23—C16—C17—C18 | −179.8 (3) |
| C10—C5—C6—C7 | −56.7 (3) | C15—C16—C17—C18 | 0.7 (5) |
| C4—C5—C6—C7 | 170.7 (2) | C23—C16—C17—C22 | −0.8 (3) |
| C5—C6—C7—C8 | 52.1 (3) | C15—C16—C17—C22 | 179.7 (2) |
| C6—C7—C8—C12 | 116.6 (3) | C22—C17—C18—C19 | −0.3 (4) |
| C6—C7—C8—C9 | −55.4 (3) | C16—C17—C18—C19 | 178.6 (3) |

| | | | |
|---|---|---|---|
| C12—C8—C9—C15 | 18.3 (4) | C17—C18—C19—C20 | −0.3 (5) |
| C7—C8—C9—C15 | −170.2 (2) | C18—C19—C20—C21 | −0.2 (5) |
| C12—C8—C9—C10 | −110.8 (3) | C19—C20—C21—C22 | 1.2 (5) |
| C7—C8—C9—C10 | 60.7 (3) | C23—N1—C22—C21 | 177.8 (3) |
| C2—C1—C10—C11 | −73.9 (2) | C23—N1—C22—C17 | −1.0 (3) |
| C2—C1—C10—C5 | 51.6 (3) | C20—C21—C22—N1 | 179.5 (3) |
| C2—C1—C10—C9 | 166.2 (2) | C20—C21—C22—C17 | −1.8 (4) |
| C6—C5—C10—C1 | 176.0 (2) | C18—C17—C22—N1 | −179.7 (2) |
| C4—C5—C10—C1 | −52.8 (3) | C16—C17—C22—N1 | 1.1 (3) |
| C6—C5—C10—C11 | −61.4 (3) | C18—C17—C22—C21 | 1.4 (4) |
| C4—C5—C10—C11 | 69.8 (2) | C16—C17—C22—C21 | −177.8 (2) |
| C6—C5—C10—C9 | 58.6 (2) | C17—C16—C23—N1 | 0.2 (3) |
| C4—C5—C10—C9 | −170.13 (18) | C15—C16—C23—N1 | 179.6 (2) |
| C8—C9—C10—C1 | −175.4 (2) | C22—N1—C23—C16 | 0.6 (3) |
| C15—C9—C10—C1 | 55.5 (3) | | |

*Hydrogen-bond geometry (Å, º)*

| *D*—H···*A* | *D*—H | H···*A* | *D*···*A* | *D*—H···*A* |
|---|---|---|---|---|
| N1—H1*N*···O3$^{i}$ | 0.88 | 1.94 | 2.821 (3) | 175 |
| O1—H1···C23$^{ii}$ | 0.84 | 2.49 | 3.166 (3) | 138 |
| O2—H2···O1$^{iii}$ | 0.84 | 1.84 | 2.676 (2) | 170 |
| N1—H1*N*···O3$^{i}$ | 0.88 | 1.94 | 2.821 (3) | 175 |
| O2—H2···O1$^{iii}$ | 0.84 | 1.84 | 2.676 (2) | 170 |

Symmetry codes: (i) −*y*+1, *x*, *z*+1; (ii) −*y*+1/2, *x*−1/2, *z*−1/2; (iii) −*x*+1/2, −*y*+1/2, *z*+1/2.

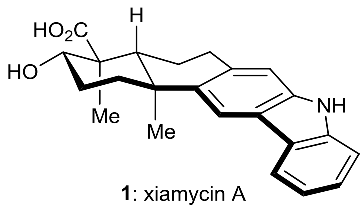


**1**: xiamycin A

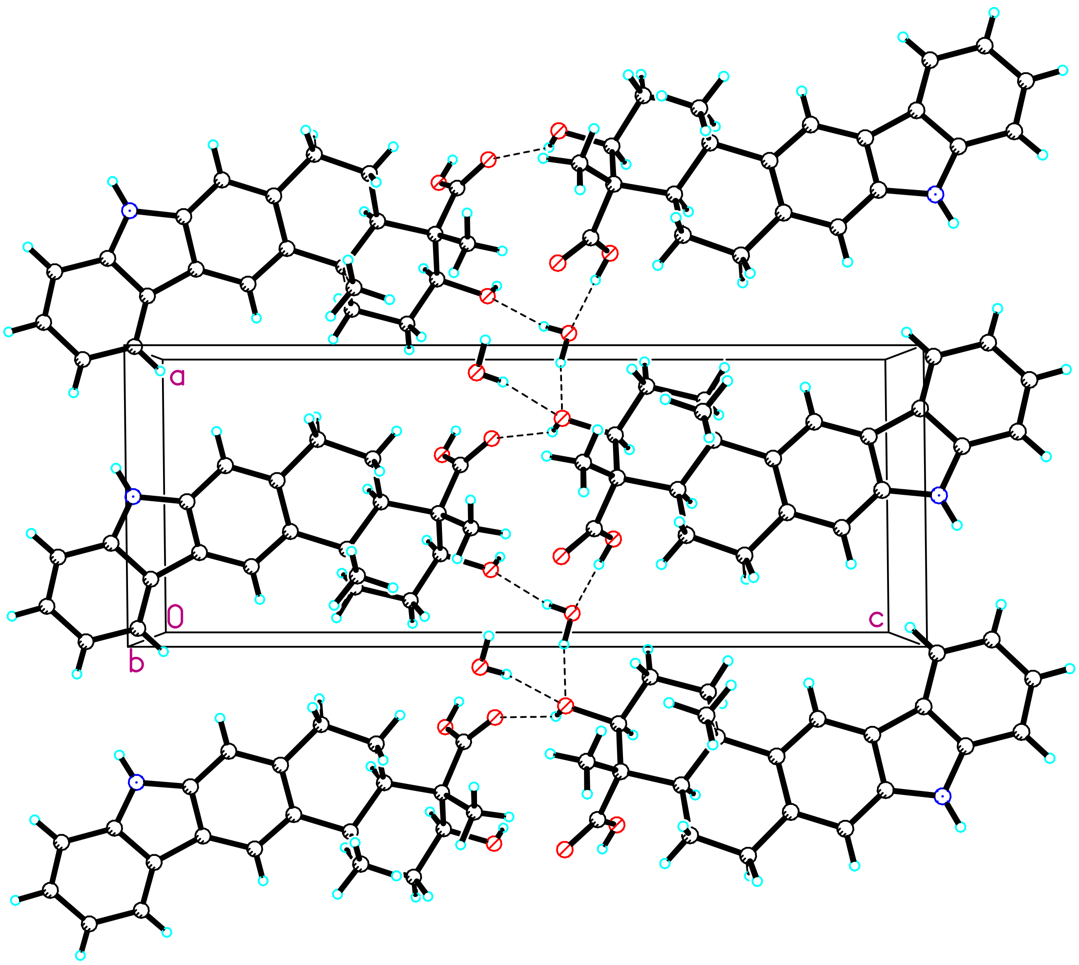

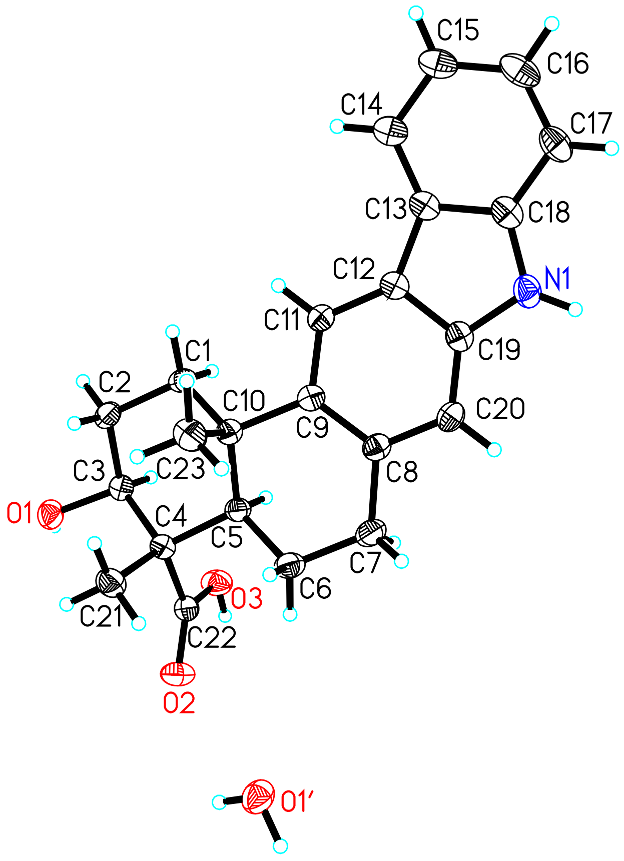


ORTEP of Compound **1**

*Crystal data*

| | |
|---|---|
| $C_{23}H_{25}NO_3 \cdot H_2O$ | $F(000) = 408$ |
| $M_r = 381.46$ | $D_x = 1.316$ Mg m$^{-3}$ |
| Monoclinic, $P2_1$ | Cu *K*α radiation, λ = 1.54178 Å |
| Hall symbol: P 2yb | Cell parameters from 4281 reflections |
| *a* = 7.3863 (15) Å | θ = 4.5–67.0 ° |
| *b* = 6.6732 (13) Å | μ = 0.72 mm$^{-1}$ |
| *c* = 19.530 (4) Å | *T* = 296 K |
| β = 90.68 (3)° | Prismatic, colorless |
| *V* = 962.6 (3) Å$^3$ | 0.26 × 0.15 × 0.10 mm |
| *Z* = 2 | |

*Data collection*

| | |
|---|---|
| Bruker APEX-II CCD diffractometer | 3198 independent reflections |
| Radiation source: fine-focus sealed tube | 3122 reflections with $I > 2\sigma(I)$ |
| graphite | $R_{int} = 0.016$ |
| φ and ω scans | $\theta_{max} = 67.0°$, $\theta_{min} = 2.3°$ |
| Absorption correction: multi-scan *SADABS* | *h* = −8→8 |

| | |
|---|---|
| $T_{min}$ = 0.670, $T_{max}$ = 0.753 | $k = -7 \rightarrow 7$ |
| 6375 measured reflections | $l = -22 \rightarrow 22$ |

*Refinement*

| | |
|---|---|
| Refinement on $F^2$ | Secondary atom site location: difference Fourier map |
| Least-squares matrix: full | Hydrogen site location: inferred from neighbouring sites |
| $R[F^2 > 2\sigma(F^2)] = 0.034$ | H atoms treated by a mixture of independent and constrained refinement |
| $wR(F^2) = 0.088$ | $w = 1/[\sigma^2(F_o^2) + (0.0575P)^2 + 0.1008P]$ where $P = (F_o^2 + 2F_c^2)/3$ |
| $S = 1.06$ | $(\Delta/\sigma)_{max} < 0.001$ |
| 3198 reflections | $\Delta\rho_{max}$ = 0.17 e Å$^{-3}$ |
| 261 parameters | $\Delta\rho_{min}$ = −0.22 e Å$^{-3}$ |
| 1 restraint | Absolute structure: Flack H D (1983), Acta Cryst. A39, 876-881 |
| Primary atom site location: structure-invariant direct methods | Flack parameter: 0.17 (18) |

*Special details*

**Geometry**. All esds (except the esd in the dihedral angle between two l.s. planes) are estimated using the full covariance matrix. The cell esds are taken into account individually in the estimation of esds in distances, angles and torsion angles; correlations between esds in cell parameters are only used when they are defined by crystal symmetry. An approximate (isotropic) treatment of cell esds is used for estimating esds involving l.s. planes.

**Refinement**. Refinement of $F^2$ against ALL reflections. The weighted R-factor wR and goodness of fit S are based on $F^2$, conventional R-factors R are based on F, with F set to zero for negative $F^2$. The threshold expression of $F^2 > 2\text{sigma}(F^2)$ is used only for calculating R-factors(gt) etc. and is not relevant to the choice of reflections for refinement. R-factors based on $F^2$ are statistically about twice as large as those based on F, and R- factors based on ALL data will be even larger.

*Fractional atomic coordinates and isotropic or equivalent isotropic displacement parameters (Å$^2$)*

| | *x* | *y* | *z* | $U_{iso}$*/$U_{eq}$ |
|---|---|---|---|---|
| O1 | 0.23010 (15) | 0.0374 (2) | 0.45074 (6) | 0.0466 (3) |
| H1A | 0.2753 | −0.0687 | 0.4635 | 0.070* |
| O1' | 0.91894 (19) | 0.7705 (2) | 0.43909 (8) | 0.0561 (4) |
| O2 | 0.70589 (17) | 0.2302 (2) | 0.45502 (6) | 0.0527 (3) |
| O3 | 0.65091 (16) | −0.0236 (2) | 0.38474 (6) | 0.0502 (3) |
| H3B | 0.7411 | −0.0697 | 0.4038 | 0.075* |
| N1 | 0.4980 (2) | 0.4781 (2) | −0.01832 (7) | 0.0465 (3) |
| H1B | 0.5964 | 0.5106 | −0.0387 | 0.056* |
| C1 | 0.1714 (2) | 0.1899 (3) | 0.26708 (8) | 0.0412 (4) |
| H1C | 0.0624 | 0.2272 | 0.2420 | 0.049* |

| | | | | |
|---|---|---|---|---|
| H1D | 0.2193 | 0.0696 | 0.2461 | 0.049* |
| C2 | 0.1224 (2) | 0.1436 (3) | 0.34109 (9) | 0.0464 (4) |
| H2A | 0.0688 | 0.2613 | 0.3618 | 0.056* |
| H2B | 0.0336 | 0.0365 | 0.3419 | 0.056* |
| C3 | 0.2885 (2) | 0.0816 (3) | 0.38204 (8) | 0.0390 (4) |
| H3A | 0.3378 | −0.0410 | 0.3619 | 0.047* |
| C4 | 0.4378 (2) | 0.2450 (3) | 0.38198 (8) | 0.0367 (3) |
| C5 | 0.4786 (2) | 0.3002 (2) | 0.30554 (8) | 0.0345 (3) |
| H5A | 0.5252 | 0.1767 | 0.2851 | 0.041* |
| C6 | 0.6304 (2) | 0.4533 (3) | 0.29833 (8) | 0.0447 (4) |
| H6A | 0.5858 | 0.5858 | 0.3096 | 0.054* |
| H6B | 0.7287 | 0.4210 | 0.3298 | 0.054* |
| C7 | 0.6984 (2) | 0.4517 (3) | 0.22563 (9) | 0.0499 (4) |
| H7A | 0.7672 | 0.5734 | 0.2181 | 0.060* |
| H7B | 0.7808 | 0.3396 | 0.2206 | 0.060* |
| C8 | 0.5544 (2) | 0.4366 (3) | 0.17075 (8) | 0.0377 (3) |
| C9 | 0.37441 (19) | 0.3790 (2) | 0.18582 (8) | 0.0346 (3) |
| C10 | 0.3118 (2) | 0.3597 (3) | 0.26054 (8) | 0.0361 (3) |
| C11 | 0.2504 (2) | 0.3574 (2) | 0.13238 (8) | 0.0368 (3) |
| H11A | 0.1323 | 0.3189 | 0.1418 | 0.044* |
| C12 | 0.3001 (2) | 0.3926 (2) | 0.06489 (8) | 0.0372 (3) |
| C13 | 0.2063 (2) | 0.3859 (3) | −0.00079 (8) | 0.0399 (4) |
| C14 | 0.0301 (3) | 0.3368 (3) | −0.02130 (9) | 0.0471 (4) |
| H14A | −0.0546 | 0.2956 | 0.0106 | 0.057* |
| C15 | −0.0163 (3) | 0.3508 (3) | −0.09019 (10) | 0.0545 (5) |
| H15A | −0.1333 | 0.3191 | −0.1046 | 0.065* |
| C16 | 0.1114 (3) | 0.4121 (3) | −0.13797 (10) | 0.0588 (5) |
| H16A | 0.0771 | 0.4213 | −0.1838 | 0.071* |
| C17 | 0.2857 (3) | 0.4593 (3) | −0.11942 (9) | 0.0530 (5) |
| H17A | 0.3693 | 0.4997 | −0.1519 | 0.064* |
| C18 | 0.3341 (3) | 0.4447 (3) | −0.04995 (8) | 0.0442 (4) |
| C19 | 0.4800 (2) | 0.4513 (2) | 0.05165 (8) | 0.0385 (3) |
| C20 | 0.6064 (2) | 0.4718 (3) | 0.10384 (9) | 0.0407 (4) |
| H20A | 0.7248 | 0.5088 | 0.0942 | 0.049* |
| C21 | 0.3879 (2) | 0.4234 (3) | 0.42690 (8) | 0.0485 (4) |
| H21A | 0.3660 | 0.3779 | 0.4727 | 0.073* |
| H21B | 0.2808 | 0.4864 | 0.4088 | 0.073* |
| H21C | 0.4858 | 0.5182 | 0.4275 | 0.073* |
| C22 | 0.6122 (2) | 0.1533 (3) | 0.41153 (8) | 0.0388 (4) |
| C23 | 0.2228 (3) | 0.5610 (3) | 0.27848 (10) | 0.0504 (4) |
| H23A | 0.1218 | 0.5846 | 0.2481 | 0.076* |
| H23B | 0.3096 | 0.6670 | 0.2736 | 0.076* |

| | | | | |
|---|---|---|---|---|
| H23C | 0.1815 | 0.5572 | 0.3249 | 0.076* |
| H1'A | 0.890 (4) | 0.700 (5) | 0.4724 (14) | 0.078 (8)* |
| H1'B | 1.027 (4) | 0.822 (5) | 0.4509 (13) | 0.083 (9)* |

*Atomic displacement parameters (Å$^2$)*

| | $U^{11}$ | $U^{22}$ | $U^{33}$ | $U^{12}$ | $U^{13}$ | $U^{23}$ |
|---|---|---|---|---|---|---|
| O1 | 0.0424 (6) | 0.0570 (8) | 0.0406 (6) | 0.0031 (5) | 0.0037 (5) | 0.0112 (5) |
| O1' | 0.0437 (7) | 0.0684 (9) | 0.0560 (8) | −0.0020 (6) | −0.0018 (6) | 0.0158 (7) |
| O2 | 0.0505 (7) | 0.0571 (8) | 0.0501 (7) | −0.0076 (6) | −0.0169 (6) | −0.0019 (6) |
| O3 | 0.0434 (6) | 0.0557 (8) | 0.0512 (6) | 0.0100 (6) | −0.0106 (5) | −0.0051 (6) |
| N1 | 0.0560 (8) | 0.0420 (9) | 0.0416 (7) | −0.0078 (7) | 0.0072 (6) | 0.0058 (6) |
| C1 | 0.0311 (7) | 0.0520 (10) | 0.0404 (8) | −0.0059 (7) | −0.0059 (6) | 0.0043 (7) |
| C2 | 0.0307 (7) | 0.0642 (12) | 0.0443 (9) | −0.0044 (8) | 0.0007 (6) | 0.0079 (8) |
| C3 | 0.0351 (8) | 0.0455 (10) | 0.0364 (8) | −0.0007 (7) | 0.0019 (6) | 0.0005 (7) |
| C4 | 0.0360 (7) | 0.0404 (9) | 0.0335 (7) | −0.0007 (7) | −0.0007 (6) | −0.0027 (6) |
| C5 | 0.0320 (7) | 0.0367 (8) | 0.0348 (7) | −0.0003 (6) | −0.0024 (6) | −0.0017 (6) |
| C6 | 0.0410 (8) | 0.0475 (10) | 0.0455 (8) | −0.0098 (8) | −0.0072 (7) | 0.0032 (8) |
| C7 | 0.0362 (8) | 0.0616 (12) | 0.0518 (10) | −0.0099 (8) | −0.0057 (7) | 0.0110 (9) |
| C8 | 0.0356 (7) | 0.0340 (9) | 0.0434 (8) | −0.0016 (6) | −0.0016 (6) | 0.0035 (6) |
| C9 | 0.0345 (8) | 0.0303 (8) | 0.0391 (8) | 0.0019 (6) | −0.0014 (6) | 0.0006 (6) |
| C10 | 0.0332 (8) | 0.0381 (9) | 0.0371 (8) | 0.0006 (6) | −0.0008 (6) | −0.0003 (7) |
| C11 | 0.0336 (7) | 0.0363 (8) | 0.0404 (8) | −0.0011 (6) | 0.0007 (6) | 0.0014 (6) |
| C12 | 0.0418 (8) | 0.0314 (9) | 0.0384 (8) | 0.0017 (6) | −0.0015 (6) | 0.0001 (6) |
| C13 | 0.0510 (9) | 0.0303 (8) | 0.0382 (8) | 0.0027 (7) | −0.0038 (6) | 0.0005 (6) |
| C14 | 0.0546 (10) | 0.0389 (10) | 0.0476 (9) | 0.0029 (7) | −0.0078 (8) | −0.0032 (7) |
| C15 | 0.0645 (11) | 0.0456 (11) | 0.0528 (10) | 0.0070 (9) | −0.0166 (9) | −0.0026 (8) |
| C16 | 0.0898 (15) | 0.0419 (12) | 0.0444 (9) | 0.0111 (10) | −0.0150 (9) | 0.0040 (8) |
| C17 | 0.0781 (13) | 0.0412 (10) | 0.0398 (9) | 0.0014 (9) | 0.0006 (8) | 0.0082 (8) |
| C18 | 0.0607 (10) | 0.0306 (9) | 0.0413 (8) | 0.0006 (8) | −0.0011 (7) | 0.0024 (7) |
| C19 | 0.0468 (8) | 0.0292 (8) | 0.0394 (8) | −0.0009 (7) | 0.0028 (6) | 0.0026 (6) |
| C20 | 0.0368 (8) | 0.0357 (9) | 0.0497 (9) | −0.0034 (6) | 0.0041 (6) | 0.0032 (7) |
| C21 | 0.0512 (9) | 0.0519 (11) | 0.0423 (8) | 0.0020 (8) | −0.0006 (7) | −0.0089 (8) |
| C22 | 0.0373 (8) | 0.0458 (10) | 0.0333 (8) | −0.0056 (7) | 0.0010 (6) | 0.0022 (7) |
| C23 | 0.0547 (10) | 0.0488 (11) | 0.0478 (9) | 0.0148 (8) | −0.0029 (8) | −0.0029 (8) |

*Geometric parameters (Å, °)*

| | | | |
|---|---|---|---|
| O1—C3 | 1.4446 (19) | C7—H7A | 0.9700 |
| O1—H1A | 0.8200 | C7—H7B | 0.9700 |
| O1'—H1'A | 0.83 (3) | C8—C20 | 1.386 (2) |
| O1'—H1'B | 0.89 (3) | C8—C9 | 1.418 (2) |
| O2—C22 | 1.204 (2) | C9—C11 | 1.388 (2) |
| O3—C22 | 1.324 (2) | C9—C10 | 1.541 (2) |

| | | | |
|---|---|---|---|
| O3—H3B | 0.8200 | C10—C23 | 1.538 (2) |
| N1—C18 | 1.371 (2) | C11—C12 | 1.392 (2) |
| N1—C19 | 1.386 (2) | C11—H11A | 0.9300 |
| N1—H1B | 0.8600 | C12—C19 | 1.411 (2) |
| C1—C2 | 1.526 (2) | C12—C13 | 1.452 (2) |
| C1—C10 | 1.542 (2) | C13—C14 | 1.396 (3) |
| C1—H1C | 0.9700 | C13—C18 | 1.410 (2) |
| C1—H1D | 0.9700 | C14—C15 | 1.388 (3) |
| C2—C3 | 1.514 (2) | C14—H14A | 0.9300 |
| C2—H2A | 0.9700 | C15—C16 | 1.396 (3) |
| C2—H2B | 0.9700 | C15—H15A | 0.9300 |
| C3—C4 | 1.551 (2) | C16—C17 | 1.370 (3) |
| C3—H3A | 0.9800 | C16—H16A | 0.9300 |
| C4—C21 | 1.526 (2) | C17—C18 | 1.402 (2) |
| C4—C22 | 1.533 (2) | C17—H17A | 0.9300 |
| C4—C5 | 1.570 (2) | C19—C20 | 1.381 (2) |
| C5—C6 | 1.525 (2) | C20—H20A | 0.9300 |
| C5—C10 | 1.557 (2) | C21—H21A | 0.9600 |
| C5—H5A | 0.9800 | C21—H21B | 0.9600 |
| C6—C7 | 1.512 (2) | C21—H21C | 0.9600 |
| C6—H6A | 0.9700 | C23—H23A | 0.9600 |
| C6—H6B | 0.9700 | C23—H23B | 0.9600 |
| C7—C8 | 1.504 (2) | C23—H23C | 0.9600 |

| | | | |
|---|---|---|---|
| C3—O1—H1A | 109.5 | C8—C9—C10 | 120.79 (13) |
| H1'A—O1'—H1'B | 104 (2) | C23—C10—C9 | 106.04 (14) |
| C22—O3—H3B | 109.5 | C23—C10—C1 | 109.50 (13) |
| C18—N1—C19 | 109.18 (14) | C9—C10—C1 | 110.47 (13) |
| C18—N1—H1B | 125.4 | C23—C10—C5 | 115.58 (13) |
| C19—N1—H1B | 125.4 | C9—C10—C5 | 108.13 (12) |
| C2—C1—C10 | 113.25 (13) | C1—C10—C5 | 107.11 (13) |
| C2—C1—H1C | 108.9 | C9—C11—C12 | 120.99 (14) |
| C10—C1—H1C | 108.9 | C9—C11—H11A | 119.5 |
| C2—C1—H1D | 108.9 | C12—C11—H11A | 119.5 |
| C10—C1—H1D | 108.9 | C11—C12—C19 | 118.67 (14) |
| H1C—C1—H1D | 107.7 | C11—C12—C13 | 134.63 (15) |
| C3—C2—C1 | 110.82 (13) | C19—C12—C13 | 106.70 (14) |
| C3—C2—H2A | 109.5 | C14—C13—C18 | 119.96 (16) |
| C1—C2—H2A | 109.5 | C14—C13—C12 | 134.00 (16) |
| C3—C2—H2B | 109.5 | C18—C13—C12 | 106.04 (15) |
| C1—C2—H2B | 109.5 | C15—C14—C13 | 118.76 (19) |
| H2A—C2—H2B | 108.1 | C15—C14—H14A | 120.6 |
| O1—C3—C2 | 107.29 (12) | C13—C14—H14A | 120.6 |

| | | | |
|---|---|---|---|
| O1—C3—C4 | 111.38 (13) | C14—C15—C16 | 120.4 (2) |
| C2—C3—C4 | 112.27 (14) | C14—C15—H15A | 119.8 |
| O1—C3—H3A | 108.6 | C16—C15—H15A | 119.8 |
| C2—C3—H3A | 108.6 | C17—C16—C15 | 122.07 (17) |
| C4—C3—H3A | 108.6 | C17—C16—H16A | 119.0 |
| C21—C4—C22 | 107.60 (13) | C15—C16—H16A | 119.0 |
| C21—C4—C3 | 111.82 (14) | C16—C17—C18 | 117.91 (18) |
| C22—C4—C3 | 108.25 (13) | C16—C17—H17A | 121.0 |
| C21—C4—C5 | 114.46 (13) | C18—C17—H17A | 121.0 |
| C22—C4—C5 | 106.34 (12) | N1—C18—C17 | 129.69 (17) |
| C3—C4—C5 | 108.07 (12) | N1—C18—C13 | 109.47 (14) |
| C6—C5—C10 | 110.78 (13) | C17—C18—C13 | 120.84 (17) |
| C6—C5—C4 | 113.25 (12) | C20—C19—N1 | 130.00 (15) |
| C10—C5—C4 | 115.89 (12) | C20—C19—C12 | 121.45 (14) |
| C6—C5—H5A | 105.3 | N1—C19—C12 | 108.55 (14) |
| C10—C5—H5A | 105.3 | C19—C20—C8 | 119.12 (14) |
| C4—C5—H5A | 105.3 | C19—C20—H20A | 120.4 |
| C7—C6—C5 | 109.55 (14) | C8—C20—H20A | 120.4 |
| C7—C6—H6A | 109.8 | C4—C21—H21A | 109.5 |
| C5—C6—H6A | 109.8 | C4—C21—H21B | 109.5 |
| C7—C6—H6B | 109.8 | H21A—C21—H21B | 109.5 |
| C5—C6—H6B | 109.8 | C4—C21—H21C | 109.5 |
| H6A—C6—H6B | 108.2 | H21A—C21—H21C | 109.5 |
| C8—C7—C6 | 115.45 (13) | H21B—C21—H21C | 109.5 |
| C8—C7—H7A | 108.4 | O2—C22—O3 | 122.28 (16) |
| C6—C7—H7A | 108.4 | O2—C22—C4 | 124.66 (16) |
| C8—C7—H7B | 108.4 | O3—C22—C4 | 113.06 (13) |
| C6—C7—H7B | 108.4 | C10—C23—H23A | 109.5 |
| H7A—C7—H7B | 107.5 | C10—C23—H23B | 109.5 |
| C20—C8—C9 | 120.81 (14) | H23A—C23—H23B | 109.5 |
| C20—C8—C7 | 117.32 (14) | C10—C23—H23C | 109.5 |
| C9—C8—C7 | 121.80 (14) | H23A—C23—H23C | 109.5 |
| C11—C9—C8 | 118.96 (14) | H23B—C23—H23C | 109.5 |
| C11—C9—C10 | 120.01 (13) | | |

| | | | |
|---|---|---|---|
| C10—C1—C2—C3 | −59.4 (2) | C4—C5—C10—C1 | −53.09 (17) |
| C1—C2—C3—O1 | −179.03 (15) | C8—C9—C11—C12 | −0.4 (2) |
| C1—C2—C3—C4 | 58.3 (2) | C10—C9—C11—C12 | 173.97 (15) |
| O1—C3—C4—C21 | −47.26 (18) | C9—C11—C12—C19 | −0.1 (2) |
| C2—C3—C4—C21 | 73.09 (17) | C9—C11—C12—C13 | −179.28 (16) |
| O1—C3—C4—C22 | 71.10 (16) | C11—C12—C13—C14 | −1.7 (3) |
| C2—C3—C4—C22 | −168.55 (13) | C19—C12—C13—C14 | 179.07 (19) |
| O1—C3—C4—C5 | −174.12 (12) | C11—C12—C13—C18 | 178.15 (19) |

| | | | |
|---|---|---|---|
| C2—C3—C4—C5 | −53.77 (17) | C19—C12—C13—C18 | −1.10 (17) |
| C21—C4—C5—C6 | 57.39 (17) | C18—C13—C14—C15 | −1.2 (3) |
| C22—C4—C5—C6 | −61.26 (17) | C12—C13—C14—C15 | 178.67 (18) |
| C3—C4—C5—C6 | −177.29 (14) | C13—C14—C15—C16 | 0.2 (3) |
| C21—C4—C5—C10 | −72.23 (17) | C14—C15—C16—C17 | 0.4 (3) |
| C22—C4—C5—C10 | 169.12 (14) | C15—C16—C17—C18 | −0.1 (3) |
| C3—C4—C5—C10 | 53.08 (17) | C19—N1—C18—C17 | 178.11 (18) |
| C10—C5—C6—C7 | −64.64 (18) | C19—N1—C18—C13 | −2.6 (2) |
| C4—C5—C6—C7 | 163.19 (14) | C16—C17—C18—N1 | 178.34 (19) |
| C5—C6—C7—C8 | 41.5 (2) | C16—C17—C18—C13 | −0.8 (3) |
| C6—C7—C8—C20 | 168.30 (17) | C14—C13—C18—N1 | −177.85 (16) |
| C6—C7—C8—C9 | −14.8 (3) | C12—C13—C18—N1 | 2.29 (19) |
| C20—C8—C9—C11 | 0.3 (2) | C14—C13—C18—C17 | 1.5 (3) |
| C7—C8—C9—C11 | −176.55 (16) | C12—C13—C18—C17 | −178.38 (16) |
| C20—C8—C9—C10 | −174.06 (15) | C18—N1—C19—C20 | −178.78 (17) |
| C7—C8—C9—C10 | 9.1 (2) | C18—N1—C19—C12 | 1.90 (19) |
| C11—C9—C10—C23 | −79.19 (18) | C11—C12—C19—C20 | 0.8 (2) |
| C8—C9—C10—C23 | 95.10 (17) | C13—C12—C19—C20 | −179.84 (15) |
| C11—C9—C10—C1 | 39.4 (2) | C11—C12—C19—N1 | −179.85 (15) |
| C8—C9—C10—C1 | −146.35 (15) | C13—C12—C19—N1 | −0.45 (17) |
| C11—C9—C10—C5 | 156.28 (14) | N1—C19—C20—C8 | 179.86 (17) |
| C8—C9—C10—C5 | −29.4 (2) | C12—C19—C20—C8 | −0.9 (2) |
| C2—C1—C10—C23 | −71.30 (18) | C9—C8—C20—C19 | 0.4 (2) |
| C2—C1—C10—C9 | 172.27 (13) | C7—C8—C20—C19 | 177.34 (16) |
| C2—C1—C10—C5 | 54.72 (17) | C21—C4—C22—O2 | −11.1 (2) |
| C6—C5—C10—C23 | −61.58 (17) | C3—C4—C22—O2 | −132.11 (16) |
| C4—C5—C10—C23 | 69.22 (18) | C5—C4—C22—O2 | 111.98 (17) |
| C6—C5—C10—C9 | 57.05 (17) | C21—C4—C22—O3 | 168.32 (14) |
| C4—C5—C10—C9 | −172.15 (13) | C3—C4—C22—O3 | 47.31 (17) |
| C6—C5—C10—C1 | 176.11 (13) | C5—C4—C22—O3 | −68.61 (16) |

*Hydrogen-bond geometry (Å, º)*

| *D*—H···*A* | *D*—H | H···*A* | *D*···*A* | *D*—H···*A* |
|---|---|---|---|---|
| O1—H1*A*···O2$^{i}$ | 0.82 | 2.09 | 2.7914 (19) | 144 |
| O3—H3*B*···O1′$^{ii}$ | 0.82 | 1.82 | 2.6240 (19) | 166 |
| O1′—H1′*A*···O1$^{iii}$ | 0.83 (3) | 2.06 (3) | 2.884 (2) | 169 (3) |
| O1′—H1′*B*···O1$^{iv}$ | 0.89 (3) | 2.08 (3) | 2.914 (2) | 155 (3) |

Symmetry codes: (i) −*x*+1, *y*−1/2, −*z*+1; (ii) *x*, *y*−1, *z*; (iii) −*x*+1, *y*+1/2, −*z*+1; (iv) *x*+1, *y*+1, *z*.

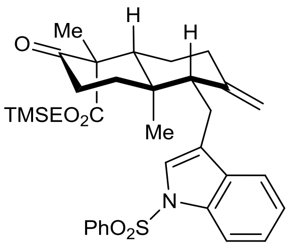


**40**

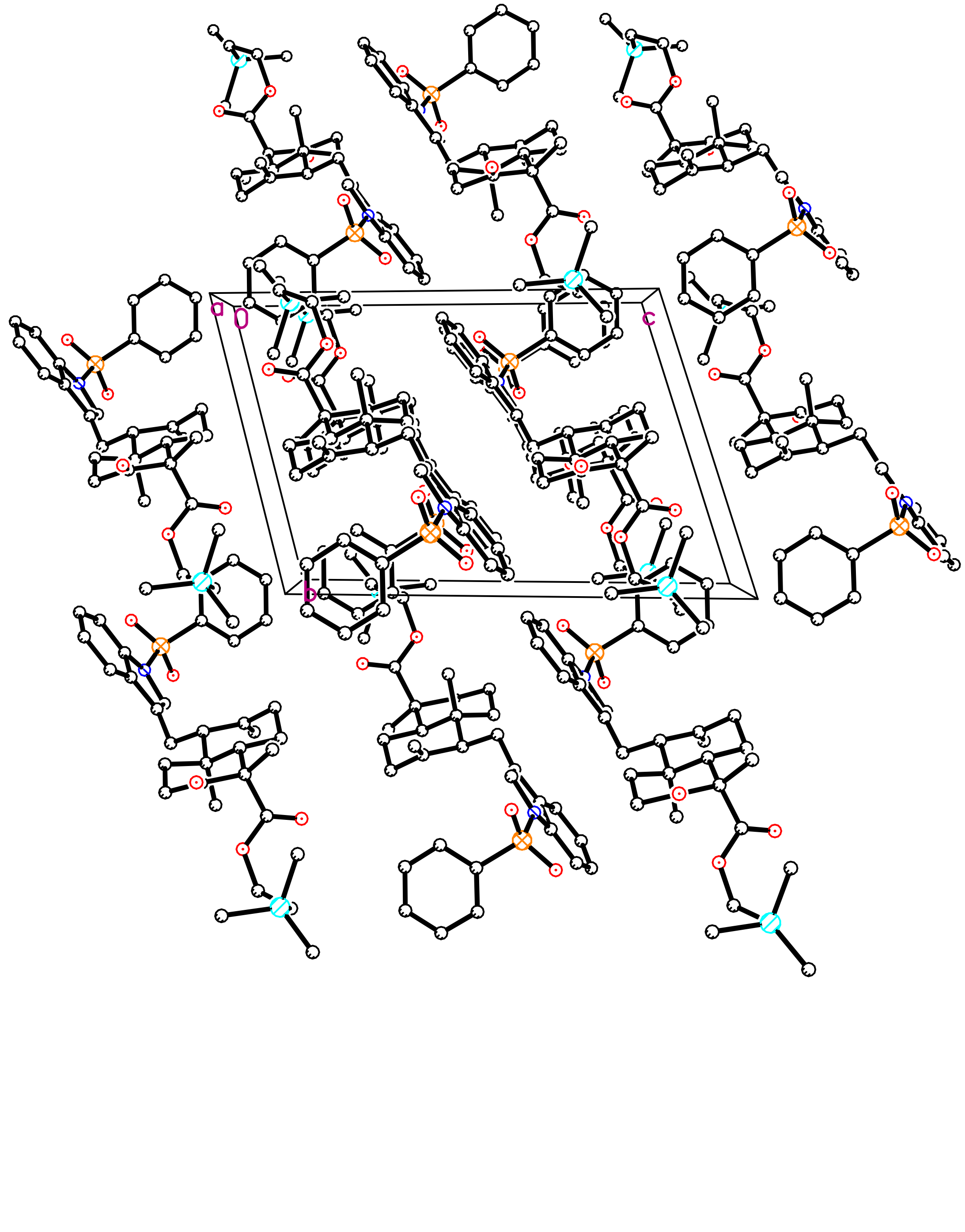

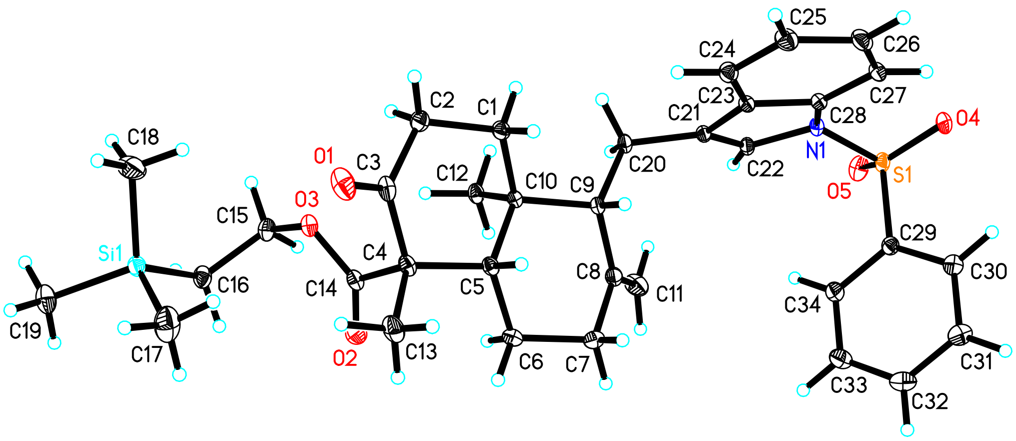


ORTEP of Compound **40**

*Crystal data*

| | |
|---|---|
| $C_{34}H_{43}NO_5SSi$ | $Z = 2$ |
| $M_r = 605.84$ | $F(000) = 648$ |
| Triclinic, $P1$ | $D_x = 1.271$ Mg m$^{-3}$ |
| $a = 10.6277$ (10) Å | Mo $K\alpha$ radiation, $\lambda = 0.71073$ Å |
| $b = 11.3209$ (11) Å | Cell parameters from 8555 reflections |
| $c = 15.3086$ (15) Å | $\theta = 2.3–30.5$ ° |
| $\alpha = 70.820$ (2)° | $\mu = 0.18$ mm$^{-1}$ |
| $\beta = 77.972$ (2)° | $T = 140$ K |
| $\gamma = 66.025$ (2)° | Block, colourless |
| $V = 1583.5$ (3) Å$^3$ | 0.35 × 0.30 × 0.24 mm |

*Data collection*

| | |
|---|---|
| Bruker APEX-II CCD diffractometer | 8125 reflections with $I > 2\sigma(I)$ |
| $\varphi$ and $\omega$ scans | $R_{int} = 0.021$ |
| Absorption correction: multi-scan *SADABS* | $\theta_{max} = 30.6°$, $\theta_{min} = 1.4$ ° |
| $T_{min} = 0.697$, $T_{max} = 0.746$ | $h = -13 \rightarrow 15$ |
| 15724 measured reflections | $k = -14 \rightarrow 16$ |
| 9565 independent reflections | $l = -21 \rightarrow 21$ |

*Refinement*

| | |
|---|---|
| Refinement on $F^2$ | 0 restraints |
| Least-squares matrix: full | Hydrogen site location: inferred from neighbouring sites |
| $R[F^2 > 2\sigma(F^2)] = 0.040$ | H-atom parameters constrained |
| $wR(F^2) = 0.134$ | $w = 1/[\sigma^2(F_o^2) + (0.0819P)^2 + 0.4595P]$ where $P = (F_o^2 + 2F_c^2)/3$ |
| $S = 1.02$ | $(\Delta/\sigma)_{max} = 0.008$ |

| | |
|---|---|
| 9565 reflections | $\Delta\rho_{max}$ = 0.45 e Å$^{-3}$ |
| 384 parameters | $\Delta\rho_{min}$ = −0.42 e Å$^{-3}$ |

*Special details*

**Geometry**. All esds (except the esd in the dihedral angle between two l.s. planes) are estimated using the full covariance matrix. The cell esds are taken into account individually in the estimation of esds in distances, angles and torsion angles; correlations between esds in cell parameters are only used when they are defined by crystal symmetry. An approximate (isotropic) treatment of cell esds is used for estimating esds involving l.s. planes.

*Fractional atomic coordinates and isotropic or equivalent isotropic displacement parameters (Å$^2$)*

| | *x* | *y* | *z* | $U_{iso}$*/$U_{eq}$ |
|---|---|---|---|---|
| S1 | 0.27052 (3) | 0.22286 (3) | 0.63769 (2) | 0.02055 (8) |
| Si1 | 0.97189 (4) | 0.96538 (4) | 0.82182 (3) | 0.02247 (9) |
| O1 | 1.08221 (11) | 0.58346 (13) | 0.71418 (9) | 0.0379 (3) |
| O2 | 0.71654 (12) | 0.73040 (10) | 0.89775 (7) | 0.0311 (2) |
| O3 | 0.81577 (9) | 0.81619 (9) | 0.75978 (7) | 0.02227 (18) |
| O4 | 0.29465 (11) | 0.13238 (10) | 0.58387 (7) | 0.0284 (2) |
| O5 | 0.14020 (10) | 0.33040 (10) | 0.64058 (8) | 0.0306 (2) |
| N1 | 0.38718 (11) | 0.29436 (10) | 0.59657 (7) | 0.01891 (19) |
| C1 | 0.80531 (13) | 0.56065 (13) | 0.63091 (9) | 0.0214 (2) |
| H1A | 0.8645 | 0.4656 | 0.6347 | 0.026* |
| H1B | 0.7584 | 0.5983 | 0.5730 | 0.026* |
| C2 | 0.89719 (14) | 0.63806 (15) | 0.62575 (10) | 0.0256 (3) |
| H2A | 0.9712 | 0.6223 | 0.5749 | 0.031* |
| H2B | 0.8410 | 0.7354 | 0.6104 | 0.031* |
| C3 | 0.96221 (13) | 0.59861 (13) | 0.71449 (10) | 0.0241 (2) |
| C4 | 0.87165 (13) | 0.57821 (12) | 0.80726 (9) | 0.0205 (2) |
| C5 | 0.77420 (12) | 0.50647 (11) | 0.80365 (8) | 0.0178 (2) |
| H5 | 0.8384 | 0.4142 | 0.8004 | 0.021* |
| C6 | 0.68079 (15) | 0.48337 (14) | 0.89365 (9) | 0.0248 (2) |
| H6A | 0.7371 | 0.4417 | 0.9474 | 0.030* |
| H6B | 0.6109 | 0.5708 | 0.9005 | 0.030* |
| C7 | 0.60771 (15) | 0.39172 (14) | 0.89261 (9) | 0.0268 (3) |
| H7A | 0.6769 | 0.3018 | 0.8907 | 0.032* |
| H7B | 0.5448 | 0.3810 | 0.9498 | 0.032* |
| C8 | 0.52679 (14) | 0.45252 (13) | 0.80899 (9) | 0.0226 (2) |
| C9 | 0.61591 (12) | 0.47275 (11) | 0.71798 (8) | 0.0162 (2) |
| H9 | 0.6882 | 0.3825 | 0.7172 | 0.019* |
| C10 | 0.69498 (12) | 0.56568 (11) | 0.71452 (8) | 0.0158 (2) |
| C11 | 0.38900 (16) | 0.49329 (18) | 0.81819 (11) | 0.0338 (3) |
| H11A | 0.3409 | 0.4840 | 0.8783 | 0.041* |
| H11B | 0.3391 | 0.5316 | 0.7646 | 0.041* |

| | | | | |
|---|---|---|---|---|
| C12 | 0.59014 (13) | 0.70994 (11) | 0.70511 (9) | 0.0203 (2) |
| H12A | 0.6370 | 0.7672 | 0.7079 | 0.030* |
| H12B | 0.5167 | 0.7101 | 0.7559 | 0.030* |
| H12C | 0.5499 | 0.7445 | 0.6455 | 0.030* |
| C13 | 0.96663 (16) | 0.49054 (15) | 0.88660 (11) | 0.0319 (3) |
| H13A | 1.0373 | 0.5273 | 0.8828 | 0.048* |
| H13B | 1.0114 | 0.3984 | 0.8809 | 0.048* |
| H13C | 0.9114 | 0.4902 | 0.9465 | 0.048* |
| C14 | 0.79134 (13) | 0.71520 (12) | 0.82800 (9) | 0.0214 (2) |
| C15 | 0.73248 (14) | 0.95339 (12) | 0.76534 (10) | 0.0241 (2) |
| H15A | 0.6355 | 0.9605 | 0.7854 | 0.029* |
| H15B | 0.7341 | 1.0161 | 0.7027 | 0.029* |
| C16 | 0.78187 (14) | 0.99626 (14) | 0.83175 (10) | 0.0262 (3) |
| H16A | 0.7563 | 0.9495 | 0.8958 | 0.031* |
| H16B | 0.7300 | 1.0937 | 0.8234 | 0.031* |
| C17 | 1.0672 (2) | 0.79300 (17) | 0.89238 (16) | 0.0494 (5) |
| H17A | 1.0724 | 0.7270 | 0.8621 | 0.074* |
| H17B | 1.0186 | 0.7765 | 0.9544 | 0.074* |
| H17C | 1.1609 | 0.7848 | 0.8978 | 0.074* |
| C18 | 1.04328 (19) | 0.9850 (3) | 0.69877 (12) | 0.0500 (5) |
| H18A | 1.1432 | 0.9624 | 0.6949 | 0.075* |
| H18B | 0.9993 | 1.0782 | 0.6627 | 0.075* |
| H18C | 1.0250 | 0.9247 | 0.6737 | 0.075* |
| C19 | 0.98595 (17) | 1.09164 (16) | 0.86881 (13) | 0.0357 (3) |
| H19A | 1.0810 | 1.0630 | 0.8838 | 0.053* |
| H19B | 0.9229 | 1.0992 | 0.9251 | 0.053* |
| H19C | 0.9613 | 1.1791 | 0.8222 | 0.053* |
| C20 | 0.53623 (12) | 0.52101 (11) | 0.63137 (8) | 0.0182 (2) |
| H20A | 0.4489 | 0.5967 | 0.6384 | 0.022* |
| H20B | 0.5921 | 0.5551 | 0.5761 | 0.022* |
| C21 | 0.50297 (12) | 0.41360 (11) | 0.61499 (8) | 0.0170 (2) |
| C22 | 0.37629 (13) | 0.40647 (11) | 0.62411 (8) | 0.0183 (2) |
| H22 | 0.2928 | 0.4674 | 0.6457 | 0.022* |
| C23 | 0.60100 (12) | 0.30198 (11) | 0.58032 (8) | 0.0172 (2) |
| C24 | 0.74155 (13) | 0.26714 (13) | 0.54976 (9) | 0.0228 (2) |
| H24 | 0.7919 | 0.3177 | 0.5538 | 0.027* |
| C25 | 0.80615 (14) | 0.15714 (14) | 0.51343 (10) | 0.0270 (3) |
| H25 | 0.9014 | 0.1328 | 0.4921 | 0.032* |
| C26 | 0.73274 (15) | 0.08163 (14) | 0.50786 (9) | 0.0267 (3) |
| H26 | 0.7798 | 0.0058 | 0.4839 | 0.032* |
| C27 | 0.59267 (14) | 0.11526 (12) | 0.53660 (9) | 0.0226 (2) |
| H27 | 0.5428 | 0.0644 | 0.5324 | 0.027* |

| | | | | |
|---|---|---|---|---|
| C28 | 0.52827 (13) | 0.22703 (12) | 0.57187 (8) | 0.0180 (2) |
| C29 | 0.31199 (13) | 0.12964 (13) | 0.75206 (9) | 0.0208 (2) |
| C30 | 0.35639 (13) | −0.00956 (13) | 0.77583 (9) | 0.0234 (2) |
| H30 | 0.3646 | −0.0544 | 0.7311 | 0.028* |
| C31 | 0.38871 (14) | −0.08228 (14) | 0.86676 (10) | 0.0286 (3) |
| H31 | 0.4177 | −0.1775 | 0.8846 | 0.034* |
| C32 | 0.37861 (17) | −0.01606 (17) | 0.93108 (10) | 0.0339 (3) |
| H32 | 0.4017 | −0.0661 | 0.9927 | 0.041* |
| C33 | 0.33474 (19) | 0.12343 (18) | 0.90591 (11) | 0.0364 (3) |
| H33 | 0.3287 | 0.1679 | 0.9504 | 0.044* |
| C34 | 0.29977 (17) | 0.19792 (15) | 0.81665 (10) | 0.0301 (3) |
| H34 | 0.2682 | 0.2932 | 0.7996 | 0.036* |

*Atomic displacement parameters (Å$^2$)*

| | $U^{11}$ | $U^{22}$ | $U^{33}$ | $U^{12}$ | $U^{13}$ | $U^{23}$ |
|---|---|---|---|---|---|---|
| S1 | 0.02058 (14) | 0.02066 (14) | 0.02514 (15) | −0.01137 (11) | −0.00477 (11) | −0.00560 (11) |
| Si1 | 0.02354 (17) | 0.02317 (17) | 0.02378 (17) | −0.01002 (13) | −0.00128 (13) | −0.00885 (13) |
| O1 | 0.0174 (4) | 0.0524 (7) | 0.0549 (7) | −0.0120 (4) | 0.0006 (4) | −0.0317 (6) |
| O2 | 0.0416 (6) | 0.0278 (5) | 0.0272 (5) | −0.0148 (4) | 0.0035 (4) | −0.0131 (4) |
| O3 | 0.0218 (4) | 0.0200 (4) | 0.0282 (5) | −0.0081 (3) | −0.0030 (3) | −0.0095 (3) |
| O4 | 0.0382 (5) | 0.0303 (5) | 0.0289 (5) | −0.0218 (4) | −0.0062 (4) | −0.0096 (4) |
| O5 | 0.0196 (4) | 0.0269 (5) | 0.0454 (6) | −0.0088 (4) | −0.0081 (4) | −0.0065 (4) |
| N1 | 0.0206 (5) | 0.0185 (4) | 0.0216 (5) | −0.0101 (4) | −0.0021 (4) | −0.0065 (4) |
| C1 | 0.0203 (5) | 0.0277 (6) | 0.0211 (5) | −0.0125 (5) | 0.0007 (4) | −0.0096 (4) |
| C2 | 0.0237 (6) | 0.0331 (7) | 0.0262 (6) | −0.0169 (5) | 0.0016 (5) | −0.0099 (5) |
| C3 | 0.0185 (5) | 0.0240 (6) | 0.0355 (7) | −0.0079 (4) | −0.0018 (5) | −0.0153 (5) |
| C4 | 0.0193 (5) | 0.0189 (5) | 0.0255 (6) | −0.0045 (4) | −0.0077 (4) | −0.0085 (4) |
| C5 | 0.0180 (5) | 0.0158 (5) | 0.0201 (5) | −0.0038 (4) | −0.0051 (4) | −0.0063 (4) |
| C6 | 0.0307 (6) | 0.0286 (6) | 0.0177 (5) | −0.0128 (5) | −0.0046 (5) | −0.0054 (4) |
| C7 | 0.0331 (7) | 0.0292 (6) | 0.0192 (6) | −0.0159 (5) | −0.0016 (5) | −0.0025 (5) |
| C8 | 0.0252 (6) | 0.0248 (6) | 0.0226 (6) | −0.0132 (5) | 0.0014 (4) | −0.0095 (4) |
| C9 | 0.0171 (5) | 0.0152 (5) | 0.0183 (5) | −0.0063 (4) | −0.0021 (4) | −0.0063 (4) |
| C10 | 0.0162 (5) | 0.0154 (4) | 0.0174 (5) | −0.0056 (4) | −0.0029 (4) | −0.0059 (4) |
| C11 | 0.0257 (7) | 0.0512 (9) | 0.0318 (7) | −0.0177 (6) | 0.0053 (5) | −0.0207 (7) |
| C12 | 0.0202 (5) | 0.0155 (5) | 0.0260 (6) | −0.0047 (4) | −0.0071 (4) | −0.0060 (4) |
| C13 | 0.0310 (7) | 0.0276 (6) | 0.0394 (8) | −0.0066 (5) | −0.0207 (6) | −0.0066 (6) |
| C14 | 0.0223 (5) | 0.0217 (5) | 0.0244 (6) | −0.0081 (4) | −0.0058 (4) | −0.0091 (4) |
| C15 | 0.0217 (6) | 0.0190 (5) | 0.0326 (7) | −0.0071 (4) | −0.0040 (5) | −0.0074 (5) |
| C16 | 0.0262 (6) | 0.0248 (6) | 0.0323 (7) | −0.0116 (5) | 0.0039 (5) | −0.0147 (5) |
| C17 | 0.0483 (10) | 0.0265 (7) | 0.0727 (13) | −0.0094 (7) | −0.0247 (10) | −0.0060 (8) |
| C18 | 0.0362 (9) | 0.0978 (16) | 0.0326 (8) | −0.0384 (10) | 0.0078 (7) | −0.0274 (9) |
| C19 | 0.0327 (7) | 0.0326 (7) | 0.0500 (9) | −0.0128 (6) | −0.0037 (7) | −0.0207 (7) |

| | | | | | | |
|---|---|---|---|---|---|---|
| C20 | 0.0201 (5) | 0.0156 (5) | 0.0222 (5) | −0.0074 (4) | −0.0053 (4) | −0.0060 (4) |
| C21 | 0.0190 (5) | 0.0163 (5) | 0.0176 (5) | −0.0074 (4) | −0.0029 (4) | −0.0052 (4) |
| C22 | 0.0208 (5) | 0.0163 (5) | 0.0207 (5) | −0.0082 (4) | −0.0025 (4) | −0.0062 (4) |
| C23 | 0.0197 (5) | 0.0179 (5) | 0.0162 (5) | −0.0078 (4) | −0.0028 (4) | −0.0054 (4) |
| C24 | 0.0215 (6) | 0.0253 (6) | 0.0247 (6) | −0.0105 (5) | 0.0003 (4) | −0.0095 (5) |
| C25 | 0.0230 (6) | 0.0296 (6) | 0.0282 (6) | −0.0073 (5) | 0.0025 (5) | −0.0138 (5) |
| C26 | 0.0310 (7) | 0.0244 (6) | 0.0257 (6) | −0.0068 (5) | −0.0002 (5) | −0.0136 (5) |
| C27 | 0.0301 (6) | 0.0209 (5) | 0.0209 (5) | −0.0105 (5) | −0.0031 (5) | −0.0088 (4) |
| C28 | 0.0220 (5) | 0.0184 (5) | 0.0161 (5) | −0.0094 (4) | −0.0024 (4) | −0.0048 (4) |
| C29 | 0.0199 (5) | 0.0234 (5) | 0.0222 (5) | −0.0113 (4) | 0.0012 (4) | −0.0077 (4) |
| C30 | 0.0194 (5) | 0.0233 (6) | 0.0264 (6) | −0.0064 (4) | −0.0010 (4) | −0.0078 (5) |
| C31 | 0.0237 (6) | 0.0267 (6) | 0.0274 (6) | −0.0044 (5) | 0.0002 (5) | −0.0050 (5) |
| C32 | 0.0344 (7) | 0.0406 (8) | 0.0224 (6) | −0.0124 (6) | −0.0003 (5) | −0.0063 (6) |
| C33 | 0.0487 (9) | 0.0446 (8) | 0.0243 (7) | −0.0245 (7) | 0.0020 (6) | −0.0136 (6) |
| C34 | 0.0409 (8) | 0.0305 (7) | 0.0255 (6) | −0.0197 (6) | 0.0041 (5) | −0.0119 (5) |

*Geometric parameters (Å, º)*

| | | | |
|---|---|---|---|
| S1—O5 | 1.4290 (10) | C13—H13A | 0.9800 |
| S1—O4 | 1.4306 (10) | C13—H13B | 0.9800 |
| S1—N1 | 1.6566 (11) | C13—H13C | 0.9800 |
| S1—C29 | 1.7616 (13) | C15—C16 | 1.5160 (19) |
| Si1—C18 | 1.8547 (18) | C15—H15A | 0.9900 |
| Si1—C17 | 1.8618 (18) | C15—H15B | 0.9900 |
| Si1—C19 | 1.8646 (15) | C16—H16A | 0.9900 |
| Si1—C16 | 1.8860 (15) | C16—H16B | 0.9900 |
| O1—C3 | 1.2149 (16) | C17—H17A | 0.9800 |
| O2—C14 | 1.2072 (17) | C17—H17B | 0.9800 |
| O3—C14 | 1.3450 (16) | C17—H17C | 0.9800 |
| O3—C15 | 1.4633 (15) | C18—H18A | 0.9800 |
| N1—C28 | 1.4130 (16) | C18—H18B | 0.9800 |
| N1—C22 | 1.4191 (14) | C18—H18C | 0.9800 |
| C1—C2 | 1.5316 (18) | C19—H19A | 0.9800 |
| C1—C10 | 1.5441 (17) | C19—H19B | 0.9800 |
| C1—H1A | 0.9900 | C19—H19C | 0.9800 |
| C1—H1B | 0.9900 | C20—C21 | 1.5009 (15) |
| C2—C3 | 1.5048 (19) | C20—H20A | 0.9900 |
| C2—H2A | 0.9900 | C20—H20B | 0.9900 |
| C2—H2B | 0.9900 | C21—C22 | 1.3569 (17) |
| C3—C4 | 1.5466 (19) | C21—C23 | 1.4506 (16) |
| C4—C14 | 1.5392 (17) | C22—H22 | 0.9500 |
| C4—C13 | 1.5440 (18) | C23—C24 | 1.3989 (17) |
| C4—C5 | 1.5723 (17) | C23—C28 | 1.4074 (16) |

| | | | |
|---|---|---|---|
| C5—C6 | 1.5350 (18) | C24—C25 | 1.3902 (18) |
| C5—C10 | 1.5619 (15) | C24—H24 | 0.9500 |
| C5—H5 | 1.0000 | C25—C26 | 1.401 (2) |
| C6—C7 | 1.5333 (19) | C25—H25 | 0.9500 |
| C6—H6A | 0.9900 | C26—C27 | 1.390 (2) |
| C6—H6B | 0.9900 | C26—H26 | 0.9500 |
| C7—C8 | 1.5003 (19) | C27—C28 | 1.3971 (16) |
| C7—H7A | 0.9900 | C27—H27 | 0.9500 |
| C7—H7B | 0.9900 | C29—C30 | 1.3889 (18) |
| C8—C11 | 1.336 (2) | C29—C34 | 1.3996 (18) |
| C8—C9 | 1.5190 (17) | C30—C31 | 1.3963 (19) |
| C9—C20 | 1.5458 (16) | C30—H30 | 0.9500 |
| C9—C10 | 1.5746 (16) | C31—C32 | 1.385 (2) |
| C9—H9 | 1.0000 | C31—H31 | 0.9500 |
| C10—C12 | 1.5372 (15) | C32—C33 | 1.391 (2) |
| C11—H11A | 0.9500 | C32—H32 | 0.9500 |
| C11—H11B | 0.9500 | C33—C34 | 1.384 (2) |
| C12—H12A | 0.9800 | C33—H33 | 0.9500 |
| C12—H12B | 0.9800 | C34—H34 | 0.9500 |
| C12—H12C | 0.9800 | | |
| O5—S1—O4 | 120.57 (6) | H13A—C13—H13C | 109.5 |
| O5—S1—N1 | 105.90 (6) | H13B—C13—H13C | 109.5 |
| O4—S1—N1 | 106.62 (6) | O2—C14—O3 | 123.79 (12) |
| O5—S1—C29 | 109.06 (6) | O2—C14—C4 | 124.15 (12) |
| O4—S1—C29 | 109.17 (6) | O3—C14—C4 | 112.05 (11) |
| N1—S1—C29 | 104.24 (6) | O3—C15—C16 | 113.56 (11) |
| C18—Si1—C17 | 109.45 (11) | O3—C15—H15A | 108.9 |
| C18—Si1—C19 | 111.14 (9) | C16—C15—H15A | 108.9 |
| C17—Si1—C19 | 109.51 (9) | O3—C15—H15B | 108.9 |
| C18—Si1—C16 | 110.13 (7) | C16—C15—H15B | 108.9 |
| C17—Si1—C16 | 110.25 (8) | H15A—C15—H15B | 107.7 |
| C19—Si1—C16 | 106.33 (7) | C15—C16—Si1 | 117.72 (9) |
| C14—O3—C15 | 117.41 (10) | C15—C16—H16A | 107.9 |
| C28—N1—C22 | 107.82 (10) | Si1—C16—H16A | 107.9 |
| C28—N1—S1 | 125.28 (8) | C15—C16—H16B | 107.9 |
| C22—N1—S1 | 120.51 (9) | Si1—C16—H16B | 107.9 |
| C2—C1—C10 | 113.54 (10) | H16A—C16—H16B | 107.2 |
| C2—C1—H1A | 108.9 | Si1—C17—H17A | 109.5 |
| C10—C1—H1A | 108.9 | Si1—C17—H17B | 109.5 |
| C2—C1—H1B | 108.9 | H17A—C17—H17B | 109.5 |
| C10—C1—H1B | 108.9 | Si1—C17—H17C | 109.5 |
| H1A—C1—H1B | 107.7 | H17A—C17—H17C | 109.5 |

| | | | |
|---|---|---|---|
| C3—C2—C1 | 113.33 (11) | H17B—C17—H17C | 109.5 |
| C3—C2—H2A | 108.9 | Si1—C18—H18A | 109.5 |
| C1—C2—H2A | 108.9 | Si1—C18—H18B | 109.5 |
| C3—C2—H2B | 108.9 | H18A—C18—H18B | 109.5 |
| C1—C2—H2B | 108.9 | Si1—C18—H18C | 109.5 |
| H2A—C2—H2B | 107.7 | H18A—C18—H18C | 109.5 |
| O1—C3—C2 | 121.54 (13) | H18B—C18—H18C | 109.5 |
| O1—C3—C4 | 120.32 (13) | Si1—C19—H19A | 109.5 |
| C2—C3—C4 | 118.15 (11) | Si1—C19—H19B | 109.5 |
| C14—C4—C13 | 106.34 (10) | H19A—C19—H19B | 109.5 |
| C14—C4—C3 | 108.90 (10) | Si1—C19—H19C | 109.5 |
| C13—C4—C3 | 108.96 (11) | H19A—C19—H19C | 109.5 |
| C14—C4—C5 | 112.74 (10) | H19B—C19—H19C | 109.5 |
| C13—C4—C5 | 109.28 (11) | C21—C20—C9 | 113.74 (9) |
| C3—C4—C5 | 110.47 (10) | C21—C20—H20A | 108.8 |
| C6—C5—C10 | 113.06 (10) | C9—C20—H20A | 108.8 |
| C6—C5—C4 | 113.27 (10) | C21—C20—H20B | 108.8 |
| C10—C5—C4 | 115.44 (10) | C9—C20—H20B | 108.8 |
| C6—C5—H5 | 104.5 | H20A—C20—H20B | 107.7 |
| C10—C5—H5 | 104.5 | C22—C21—C23 | 107.21 (10) |
| C4—C5—H5 | 104.5 | C22—C21—C20 | 127.04 (11) |
| C7—C6—C5 | 110.74 (10) | C23—C21—C20 | 125.65 (11) |
| C7—C6—H6A | 109.5 | C21—C22—N1 | 109.82 (10) |
| C5—C6—H6A | 109.5 | C21—C22—H22 | 125.1 |
| C7—C6—H6B | 109.5 | N1—C22—H22 | 125.1 |
| C5—C6—H6B | 109.5 | C24—C23—C28 | 119.46 (11) |
| H6A—C6—H6B | 108.1 | C24—C23—C21 | 132.27 (11) |
| C8—C7—C6 | 109.35 (11) | C28—C23—C21 | 108.04 (10) |
| C8—C7—H7A | 109.8 | C25—C24—C23 | 118.71 (12) |
| C6—C7—H7A | 109.8 | C25—C24—H24 | 120.6 |
| C8—C7—H7B | 109.8 | C23—C24—H24 | 120.6 |
| C6—C7—H7B | 109.8 | C24—C25—C26 | 120.95 (13) |
| H7A—C7—H7B | 108.3 | C24—C25—H25 | 119.5 |
| C11—C8—C7 | 120.89 (13) | C26—C25—H25 | 119.5 |
| C11—C8—C9 | 125.69 (13) | C27—C26—C25 | 121.42 (12) |
| C7—C8—C9 | 113.19 (11) | C27—C26—H26 | 119.3 |
| C8—C9—C20 | 113.50 (10) | C25—C26—H26 | 119.3 |
| C8—C9—C10 | 110.91 (9) | C26—C27—C28 | 117.19 (12) |
| C20—C9—C10 | 112.53 (9) | C26—C27—H27 | 121.4 |
| C8—C9—H9 | 106.4 | C28—C27—H27 | 121.4 |
| C20—C9—H9 | 106.4 | C27—C28—C23 | 122.22 (11) |
| C10—C9—H9 | 106.4 | C27—C28—N1 | 130.49 (11) |

| | | | |
|---|---|---|---|
| C12—C10—C1 | 110.55 (10) | C23—C28—N1 | 107.03 (10) |
| C12—C10—C5 | 113.19 (9) | C30—C29—C34 | 121.66 (12) |
| C1—C10—C5 | 106.74 (9) | C30—C29—S1 | 119.28 (10) |
| C12—C10—C9 | 108.79 (9) | C34—C29—S1 | 119.06 (10) |
| C1—C10—C9 | 109.12 (9) | C29—C30—C31 | 118.72 (12) |
| C5—C10—C9 | 108.35 (9) | C29—C30—H30 | 120.6 |
| C8—C11—H11A | 120.0 | C31—C30—H30 | 120.6 |
| C8—C11—H11B | 120.0 | C32—C31—C30 | 120.20 (13) |
| H11A—C11—H11B | 120.0 | C32—C31—H31 | 119.9 |
| C10—C12—H12A | 109.5 | C30—C31—H31 | 119.9 |
| C10—C12—H12B | 109.5 | C31—C32—C33 | 120.30 (14) |
| H12A—C12—H12B | 109.5 | C31—C32—H32 | 119.8 |
| C10—C12—H12C | 109.5 | C33—C32—H32 | 119.8 |
| H12A—C12—H12C | 109.5 | C34—C33—C32 | 120.59 (14) |
| H12B—C12—H12C | 109.5 | C34—C33—H33 | 119.7 |
| C4—C13—H13A | 109.5 | C32—C33—H33 | 119.7 |
| C4—C13—H13B | 109.5 | C33—C34—C29 | 118.51 (14) |
| H13A—C13—H13B | 109.5 | C33—C34—H34 | 120.7 |
| C4—C13—H13C | 109.5 | C29—C34—H34 | 120.7 |
| O5—S1—N1—C28 | −172.20 (10) | C13—C4—C14—O3 | 119.69 (12) |
| O4—S1—N1—C28 | −42.64 (11) | C3—C4—C14—O3 | 2.41 (14) |
| C29—S1—N1—C28 | 72.79 (11) | C5—C4—C14—O3 | −120.57 (11) |
| O5—S1—N1—C22 | 39.35 (11) | C14—O3—C15—C16 | 79.77 (14) |
| O4—S1—N1—C22 | 168.92 (9) | O3—C15—C16—Si1 | 48.65 (15) |
| C29—S1—N1—C22 | −75.65 (10) | C18—Si1—C16—C15 | 34.16 (14) |
| C10—C1—C2—C3 | 52.56 (15) | C17—Si1—C16—C15 | −86.71 (13) |
| C1—C2—C3—O1 | 137.70 (13) | C19—Si1—C16—C15 | 154.67 (11) |
| C1—C2—C3—C4 | −42.61 (16) | C8—C9—C20—C21 | −73.62 (13) |
| O1—C3—C4—C14 | 94.59 (14) | C10—C9—C20—C21 | 159.39 (10) |
| C2—C3—C4—C14 | −85.11 (13) | C9—C20—C21—C22 | 110.58 (13) |
| O1—C3—C4—C13 | −21.01 (17) | C9—C20—C21—C23 | −73.71 (15) |
| C2—C3—C4—C13 | 159.29 (11) | C23—C21—C22—N1 | −0.10 (13) |
| O1—C3—C4—C5 | −141.08 (12) | C20—C21—C22—N1 | 176.25 (11) |
| C2—C3—C4—C5 | 39.23 (15) | C28—N1—C22—C21 | 1.77 (13) |
| C14—C4—C5—C6 | −57.72 (13) | S1—N1—C22—C21 | 155.10 (9) |
| C13—C4—C5—C6 | 60.31 (14) | C22—C21—C23—C24 | 172.82 (13) |
| C3—C4—C5—C6 | −179.82 (10) | C20—C21—C23—C24 | −3.6 (2) |
| C14—C4—C5—C10 | 74.87 (13) | C22—C21—C23—C28 | −1.62 (13) |
| C13—C4—C5—C10 | −167.11 (11) | C20—C21—C23—C28 | −178.03 (11) |
| C3—C4—C5—C10 | −47.23 (13) | C28—C23—C24—C25 | −1.26 (18) |
| C10—C5—C6—C7 | 56.37 (13) | C21—C23—C24—C25 | −175.18 (13) |
| C4—C5—C6—C7 | −169.90 (10) | C23—C24—C25—C26 | −0.4 (2) |

| | | | |
|---|---|---|---|
| C5—C6—C7—C8 | −56.99 (14) | C24—C25—C26—C27 | 1.3 (2) |
| C6—C7—C8—C11 | −115.39 (15) | C25—C26—C27—C28 | −0.41 (19) |
| C6—C7—C8—C9 | 59.33 (14) | C26—C27—C28—C23 | −1.31 (18) |
| C11—C8—C9—C20 | −11.77 (18) | C26—C27—C28—N1 | 172.12 (12) |
| C7—C8—C9—C20 | 173.81 (10) | C24—C23—C28—C27 | 2.18 (18) |
| C11—C8—C9—C10 | 116.07 (15) | C21—C23—C28—C27 | 177.45 (11) |
| C7—C8—C9—C10 | −58.35 (13) | C24—C23—C28—N1 | −172.60 (11) |
| C2—C1—C10—C12 | 65.41 (13) | C21—C23—C28—N1 | 2.67 (13) |
| C2—C1—C10—C5 | −58.09 (13) | C22—N1—C28—C27 | −176.92 (12) |
| C2—C1—C10—C9 | −174.98 (10) | S1—N1—C28—C27 | 31.35 (18) |
| C6—C5—C10—C12 | 67.44 (13) | C22—N1—C28—C23 | −2.73 (13) |
| C4—C5—C10—C12 | −65.24 (13) | S1—N1—C28—C23 | −154.46 (9) |
| C6—C5—C10—C1 | −170.71 (10) | O5—S1—C29—C30 | 132.09 (11) |
| C4—C5—C10—C1 | 56.61 (12) | O4—S1—C29—C30 | −1.53 (12) |
| C6—C5—C10—C9 | −53.31 (12) | N1—S1—C29—C30 | −115.15 (11) |
| C4—C5—C10—C9 | 174.01 (9) | O5—S1—C29—C34 | −48.13 (13) |
| C8—C9—C10—C12 | −70.55 (12) | O4—S1—C29—C34 | 178.26 (11) |
| C20—C9—C10—C12 | 57.81 (12) | N1—S1—C29—C34 | 64.63 (12) |
| C8—C9—C10—C1 | 168.75 (10) | C34—C29—C30—C31 | 0.3 (2) |
| C20—C9—C10—C1 | −62.88 (12) | S1—C29—C30—C31 | −179.88 (10) |
| C8—C9—C10—C5 | 52.89 (12) | C29—C30—C31—C32 | −1.1 (2) |
| C20—C9—C10—C5 | −178.75 (9) | C30—C31—C32—C33 | 0.7 (2) |
| C15—O3—C14—O2 | −8.45 (18) | C31—C32—C33—C34 | 0.4 (3) |
| C15—O3—C14—C4 | 172.45 (10) | C32—C33—C34—C29 | −1.2 (2) |
| C13—C4—C14—O2 | −59.41 (17) | C30—C29—C34—C33 | 0.8 (2) |
| C3—C4—C14—O2 | −176.69 (12) | S1—C29—C34—C33 | −179.00 (12) |
| C5—C4—C14—O2 | 60.33 (16) | | |

*Hydrogen-bond geometry (Å, °)*

| *D*—H···*A* | *D*—H | H···*A* | *D*···*A* | *D*—H···*A* |
|---|---|---|---|---|
| C22—H22···O1$^{i}$ | 0.95 | 2.32 | 3.2579 (16) | 170 |
| C27—H27···O4 | 0.95 | 2.46 | 3.0382 (18) | 119 |

Symmetry code: (i) *x*−1, *y*, *z*.

**Supplementary References**

1. Lan, J.; Liu, Z.; Yuan, H.; Peng, L.; Li, W.-D. Z.; Li, Y.; Li, Y. & Chan, A. S. C. First total synthesis and absolute configuration of marine cembrane diterpenoid (+)-11,12-epoxysarcophytol A. *Tetrahedron Lett.* **41**, 2181–2184 (2000).
2. Shibuya, M.; Tomizawa, M.; Suzuki, I. & Iwabuchi, Y. 2-Azaadamantane *N*-oxyl (AZADO) and 1-Me-AZADO: highly efficient organocatalysts for oxidation of alcohols. *J. Am. Chem. Soc.* **128**, 8412–8413 (2006).
3. Bian, M.; Wang, Z.; Xiong, X.; Sun, Y.; Matera, C.; Nicolaou, K. C. & Li, A. Total syntheses of anominine and tubingensin A. *J. Am. Chem. Soc.* **134**, 8078–8081 (2012).
4. Ueda, Y.; Roberge, G. & Vinet, V. A simple method of preparing trimethylsilyl- and *tert*-butyldimethylsilyl-enol ethers of *α*-diazoacetoacetates and their use in the synthesis of a chiral precursor to thienamycin analogs. *Can. J. Chem.* **62**, 2936–2940 (1984).
5. Kitagawa, Y.; Itoh, A.; Hashimoto, S.; Yamamoto, H. & Nozaki, H. Total synthesis of humulene. A stereoselective approach. *J. Am. Chem. Soc.* **99**, 3864–3867 (1977).
6. Umbreit, M. A. & Sharpless, K. B. Allylic oxidation of olefins by catalytic and stoichiometric selenium dioxide with *tert*-butyl hydroperoxide. *J. Am. Chem. Soc.* **99**, 5526–5528, (1977).
7. McNeil, D. W.; Rose, J. M.; David, E.; Shih, C. K.; Grob, P. M. & Kelly, T. A. Novel non-nucleoside inhibitors of human immunodeficiency virus type 1 reverse transcriptase. 6. 2-indol-3-yl- and 2-azaindol-3-yl- dipyridodiazepinones. *J. Med. Chem.* **40**, 2430–2433 (1997).
8. Westermaier, M. & Mayr, H. Electrophilic allylations and benzylations of indoles in neutral aqueous or alcoholic solutions. *Org. Lett.* **8**, 4791–4794 (2006).
9. Reed, L. J. & Muench, H. A simple method of estimating fifty percent endpoints. *The American Journal of Hygiene* **27**, 493–497 (1938).
10. Moradpour, D. Evans, M. J.; Gosert, R.; Yuan, Z.; Blum, H. E.; Goff, S. P.; Lindenbach, B. D. & Rice, C. M. Insertion of green fluorescent protein into nonstructural protein 5A allows direct visualization of functional hepatitis C virus replication complexes. *J. Virol.* **78**, 7400–7409 (2004).
